# Improving communication of prognostic uncertainty in advanced illness: a realist review

**DOI:** 10.1136/bmjopen-2025-115385

**Published:** 2026-07-01

**Authors:** Simon N Etkind, Katharine Weetman, Katy Hyams, Tanvi Acharya, Farhad Shokraneh, John I MacArtney

**Affiliations:** 1 Primary Care Unit, University of Cambridge, Cambridge, UK; 2 Cambridge University Hospitals NHS Foundation Trust, Cambridge, UK; 3 School of Medical Sciences, College of Medicine & Health, University of Birmingham, Birmingham, UK; 4 Warwick Medical School, University of Warwick, Coventry, UK

**Keywords:** INTERNAL MEDICINE, PALLIATIVE CARE, GERIATRIC MEDICINE, Prognosis, Systematic Review

## Abstract

**Objectives:**

Prognostic uncertainty is common in advanced illness, driven by multimorbidity, treatment complexities and unpredictable disease trajectories. When inadequately acknowledged or discussed, it can lead to patient and family distress and inhibit decision-making.

We aimed to build a programme theory of how, why and for whom communication of prognostic uncertainty ‘works’ (or not) in advanced illness and palliative care contexts.

**Design:**

Realist systematic review, reported according to RAMESES criteria.

**Data sources:**

Embase, MEDLINE, CINAHL, Cochrane Database of Systematic Reviews, SCI via Web of Science, PsycINFO, SSCI and multiple grey literature sources. We included evidence examining communication of prognostic uncertainty in adults with advanced illnesses.

**Data extraction and synthesis:**

We coded papers qualitatively to identify Context–Mechanism–Outcome Configurations (CMOCs) relevant to prognostic uncertainty communication. Supported by clinical and lay stakeholder consultation, we refined these into a programme theory.

**Results:**

From 4634 search results, we included 205 studies with 46 579 participants across 11 countries. The final programme theory identified contexts of communication: socio-cultural norms and values, patient and family preferences, communication models and interventions, clinician uncertainty tolerance, training and experience. Mechanisms of communication were mediated by the care relationship, the timing and initiation of discussion, the content of communication and explicit versus implicit communication. Relevant outcomes were emotions, hope, distress, decision making and care planning. There was no single model of effective communication as this varied based on individual characteristics and preferences, but openness was usually viewed positively.

**Conclusion:**

This programme theory provides a new understanding of the contexts and mechanisms, such as explicitness of discussion, that support effective communication of prognostic uncertainty across advanced illness contexts to reduce distress and aid decision making. Sociocultural factors are important, but they have the most limited supporting evidence, especially in low- and middle-income countries. The model presented here may support professionals in individualised and compassionate communication of prognostic uncertainty

**STRENGTHS AND LIMITATIONS OF THIS STUDY**

- This review used an extensive search designed by an information specialist, which enabled synthesis of a large and diverse literature across multiple illness contexts.
- The realist design enabled theory development to explain how communication of prognostic uncertainty works, for whom and in what contexts.
- Stakeholder involvement ensured relevance to the population of interest.
- While there were no language limitations in our search, we searched primarily English language databases, which may have limited our ability to access literature in other languages.
- In line with the realist approach, we aimed to synthesise sufficient literature to develop a programme theory rather than finding all existing relevant literature.

## Background

People with advanced illnesses commonly experience uncertainty in physical, social, practical, temporal and existential domains.^1–3^ While some illness uncertainties can be resolved, eg, through diagnostic testing or information provision, many cannot.^4 5^ How uncertainty is managed and communicated is therefore increasingly important for those with serious illness and those caring for them,^6^ and it is a recognised research priority.^7^

Prognostic uncertainty is a specific subset of illness uncertainty that is increasing due to population changes and increasing illness complexity.^8 9^ It can affect healthcare delivery in multiple settings by inhibiting timely end of life discussions and identification of needs,^10^ and it can be a barrier for accessing services and benefits (eg, palliative care, special rules access to benefits) that are gated by prognosis.^11 12^ Consequently, poor communication of prognostic uncertainty may worsen inequities in the context of rising palliative care need.^13 14^ For this reason, the importance of communicating prognostic uncertainty is increasingly recognised.^7 15 16^

Most patients prefer open discussions of prognostic uncertainty, but such communication is often avoided or implicit,^17–19^ which may partly be attributed to health professional intolerance of uncertainty, perceptions that expressing uncertainty is a sign of failure, or clinical inexperience.^17 18^ If poorly communicated or unshared, prognostic uncertainty can be distressing for patients, carers, and health professionals alike, inhibiting decision making.^3^

Communication of prognosis itself is well-researched.^15 20^ However, there is no evidence synthesis to inform how prognostic *uncertainty* can be best communicated to improve patient outcomes, and existing research has not explicitly focused on the mechanisms of communication. Many patients, health systems and wider factors are likely to affect optimal communication,^21 22^ so an explanatory approach is needed to inform preferred approaches to prognostic uncertainty communication, while accounting for these contextual factors.

We aimed to identify, understand and explain current practice of communicating prognostic uncertainty in advanced illness contexts, including how and why different outcomes are achieved through verbal and written communications between healthcare professionals, patients and their relatives and/or carers.

## Methods

### Review question

How, why and for whom does the communication of prognostic uncertainty ‘work’ (or not) in serious illness contexts potentially relevant to palliative and end-of-life care?

### Design

Stakeholder informed realist systematic review and synthesis, reported according to the RAMESES standards (online supplemental appendix 1).^23^ We chose this theory-driven approach to generate explanations of how communicating prognostic uncertainty may be theorised to ‘work’ (or not), and if so, under what contexts, for whom, why and to what extent.^24 25^ Realist methodology is particularly suited to investigate complex interventions and sociological issues, such as that under scrutiny here.^9 24 26^ The review protocol is registered on Prospero (CRD42024563546). This was a literature review, therefore ethical approval was not required

10.1136/bmjopen-2025-115385.supp1 Supplementary data

### Patient and public involvement

The review was supported by an advisory group of two clinicians (one GP and one palliative care consultant) and two lay advisors with relevant lived experience. The group comprised advisors from across the UK and were from a range of cultural backgrounds.

We consulted the advisory group throughout the review to ensure the question and programme theory fit with lived experience. Stakeholders contributed to developing the initial and final programme theories and refined the CMOC list.

### Inclusion and exclusion criteria

We included evidence relating to verbal or written communication of prognostic uncertainty in advanced and life-limiting illness contexts relevant to palliative care. We included communication between healthcare professionals AND patients and/or relatives and/or carers and/or other healthcare professionals, in any healthcare setting. We included all study designs, as long as they contained content that could be scrutinised against the initial programme theory and contained at least one statement regarding how or for whom communication of prognostic uncertainty works and why.^24 25^ We included published peer reviewed papers and grey literature such as reports, theses and guidelines.^25 27^

Exclusion criteria: Prognostic uncertainty for patients without advanced or life-limiting illness; evidence specifically related to patients<18 years old, communication of prognosis without specific consideration of uncertainty.

### Initial programme theory

On 29 April 2024, we conducted scoping searches in PubMed and Google Scholar using a title search for terms prognosis, palliative, end-of-life, communication and uncertainty and identified 54 highly relevant papers.^28 29^ These papers were reviewed by four authors (SE, KW, FS, JM) who each produced summary points and independently developed ideas for an initial programme theory. The initial theory was then refined through repeated discussion of these ideas within the research team and patient and clinician advisory group.^24 25^ We produced a visual ‘map’ of the initial theory (online supplemental appendix 2) in collaboration with the advisory group. At each review of the theory, we monitored its iterative development and considered it sufficiently developed when no major conceptual changes were identified. The initial theory formed the starting point for our searches and analysis (online supplemental appendix 2).

10.1136/bmjopen-2025-115385.supp2 Supplementary data

### Searches

Following realist review guidelines,^28 30 31^ we searched the following sources with no limitations to time, language, document type or publication status on 18 August 2024^32^: Embase, MEDLINE, CINAHL, Cochrane Database of Systematic Reviews, Science Citation Index-Expanded via Web of Science, PsycINFO and the Social Science Citation Index. To identify grey literature, we searched the following websites and repositories: NHS Digital, NHS Improvement, Royal College of Physicians, General Medical Council, NICE Guidelines, Marie Curie, Google Scholar, ProQuest Dissertations & Theses Global, Trip Database and the Department of Health and Social Care.

The search strategy was designed with an information specialist (FS) and included terms relating to prognosis, uncertainty and palliative care/advanced illness; search terminology was informed by papers from the scoping search (online supplemental appendix 3).

10.1136/bmjopen-2025-115385.supp3 Supplementary data

### Screening and data extraction

A single reviewer screened search results for eligibility, with 10% s screening at title/abstract stage, as per previous realist reviews.^27 33^ We assessed inter-rater agreement on these 10% and considered a score of k>0.8 to reflect sufficient agreement. 30% of results were double-screened at the full text stage. Disagreements were resolved by discussion within the research team (SE, TA, KW & FS) with reference to the inclusion/exclusion criteria. These discussions enabled a consistent interpretation of the inclusion criteria in subsequent screening decisions.

We used a bespoke data extraction form to extract relevant information from each article, populated by one researcher, with 10% double data extraction (online supplemental appendix 4).

10.1136/bmjopen-2025-115385.supp4 Supplementary data

To extract data relevant to ‘context, mechanism, outcome’ configurations (CMOCs), we imported the full text of included articles into NVivo (version 14 with collaboration cloud, QSR international). TA, FS, KW and SE coded included articles to identify text relevant to the programme theory. We coded generally for areas of ‘context’, ‘mechanism’ and ‘outcome’, and specifically for areas such as ‘content of communication’ that could contribute to CMOCs. The coding framework was regularly reviewed and updated by the research team during data extraction.

### Synthesis

Based on the coding of ‘contexts, mechanisms and outcomes’, we developed a series of CMOCs related to the review question, that is, statements relating to communicating prognostic uncertainty where a context could be linked to a mechanism and outcome. These were tabulated during coding and each new CMOC was checked against the existing list to avoid repetition. The full list was subsequently checked after analysis and four overlapping CMOCs were combined.

The research team reviewed the CMOC list in relation to the domains of the initial programme theory and generated thematic areas which CMOCs related to. Additional domains which extended the initial programme theory were also identified during coding through reflexive consideration of the CMOCs coded.

The final programme theory was developed by considering the relationships of the thematic areas (and the CMOCs within them) to each other. We did not attempt to categorise thematic areas themselves as ‘contexts’, ‘mechanisms’ or ‘outcomes’ because most themes bridged across these categories, but we did consider interactions between thematic areas in the context of prognostic uncertainty communication to build a visual model of the programme theory.

The research team then considered whether and how each CMOC fit within the theory. Where needed, we adjusted the configuration of the theory to ensure all CMOCs ‘fit’. We also conducted one round of written feedback and one meeting with the advisory group to support theory development

### Rigour and relevance assessments

We made a judgement of low, moderate or high rigour based on an evaluation of the research methods in relation to the question being asked by each manuscript.^34^ Papers collecting primary data were rated as ‘high’ rigour where methods were appropriate for the question, and there were no flaws identified that affected trustworthiness of the findings. If minor biases or issues were identified, we rated as moderate. If serious concerns limited interpretation, we rated as low rigour. Literature reviews were rated as low rigour if there was no description of a search strategy. Systematic reviews were rated as moderate or high rigour. Rigour was assessed by KH with 25% checked by SE. Relevance was assessed by KH with 50% checked by SE.

We also assessed relevance for all included papers.^34^ Relevance was defined as the alignment of each paper to the review question and initial programme theory, and papers were rated as high, moderate or low relevance based on the degree of focus on our research question.

Rigour and relevance scores were available to the research team at the point of theory development, which enabled us to adjust the weight given to CMOCs depending on the evidence supporting them; however, we were mindful that in some cases, useful concepts can be identified in ‘bad’ research.^35^

## Results

From 4634 search results, we included 205 articles (figure 1). (Cohen’s K 0.88 for agreement in double-screened articles). Articles were mostly from the USA (71), UK (61) and continental Europe (27). Other countries were Canada (14), Australia (20), New Zealand (2), Ghana (1), Hong Kong (1), India (1) and China (1).

**Figure 1:**
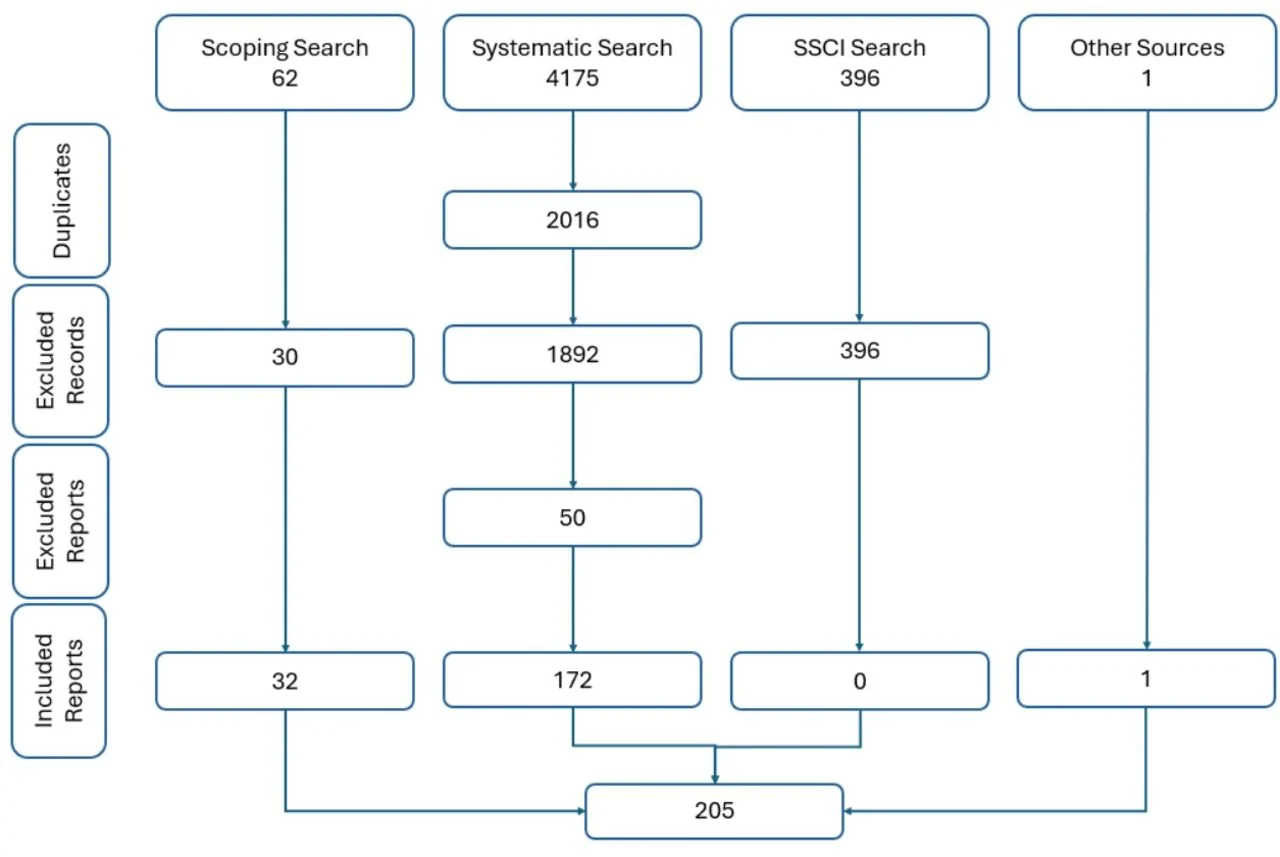
Preferred Reporting Items for Systematic reviews and Meta-Analyses (PRISMA) chart.

Most articles were qualitative analyses (79) or systematic/narrative literature reviews (71). We also included observational quantitative or mixed-methods studies, theses, policy documents, opinion or discussion articles and book chapters. The 103 studies involving primary data collection had 46 579 unique participants, including 39 448 patients, 3959 healthcare professionals and 2003 carers. 82 articles covered all or mixed settings, with 75 in secondary care, 20 in primary or community care and 11 in hospice settings. 41 focused on HCPs only, 14 on patients, 15 on carers and 102 on combinations of HCPs, patients and/or carers. The most common patient populations were cancer, heart failure, general palliative care and intensive care.

82 articles were of high rigour, 76 moderate and 47 low. Most low rigour articles did not involve primary data collection. 63 articles were highly relevant to the programme theory, 91 moderately relevant and 51 were of low relevance (online supplemental appendix 5).

10.1136/bmjopen-2025-115385.supp5 Supplementary data

### Context–mechanism–outcome configurations

We identified 52 context-mechanism-outcome configurations (CMOC) within eight thematic areas. 1. Patient and family preferences; 2. clinician training and experience; 3. prognostic communication models and interventions; 4. timing and initiation of prognostic dialogue; 5. content of communication; 6. explicit disclosure of uncertainty and open communication; 7. implicit or avoided disclosure of information and uncertainty; 8. emotions, hope and distress. We elaborate on each of these below; examples of how CMOCs relate to the findings are given in the text. The full list of CMOCs is included in online supplemental appendix 6.

10.1136/bmjopen-2025-115385.supp6 Supplementary data

Based on discussion of these areas with stakeholder and PPI contributors, we developed a final programme theory (figure 2). References for all included articles are available in online supplemental appendix 7.

10.1136/bmjopen-2025-115385.supp7 Supplementary data

**Figure 2:**
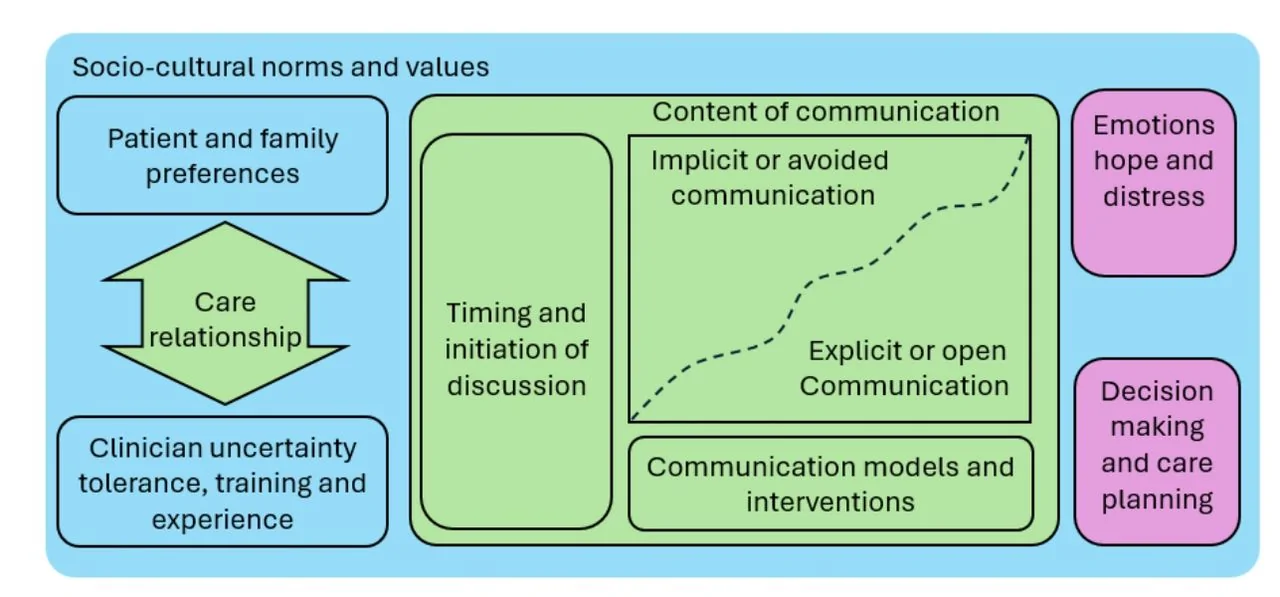
Final programme theory of how communication of prognostic uncertainty ‘works’, for whom and in what contexts.

#### Patient and family preferences

The inherent uncertainty in life-limiting illness cannot be eliminated^1 36 37^ (eg, difficulty of frailty prognostication,^38^ rare diseases,^39^ ICU contexts)^40–42^ and patients and carers may expect a degree of prognostic uncertainty within life-limiting illness contexts.^43^ Most included studies identified that patients usually want sensitive and honest communication by clinicians when prognosis is uncertain^44–50^; paired with empathy (CMOC9,11^51–53^) and a sense of optimism^16 49^ or hopefulness^53–55^ (but not false hope^19 41 56–58^ (CMOC13)). In order for patients and families to plan ahead in physical, financial, social and emotional domains^39 42 59–62^ (CMOC6) and make informed decisions at the end of their life^60 63^ (eg, regarding place of death), they need to be given the option of knowing prognostic information alongside realistic expectations and timeframes.^64 65^ Unrealistic expectations can lead to overly aggressive treatment plans (CMOC35, 36, 41).^54 66–69^

Internationally, although many people desire prognostic estimates and to be fully informed of prognostic uncertainty (CMOC5),^16 19 54 59 62 66 68 70–81^ information needs vary^39 52 60 72 74–76 82–86^; not all patients want to know (CMOC7).^39 44 54 60 78 82 87–90^ In some situations, patients or families may be understood by healthcare professionals as being ‘in denial’,^54 64 75 85^ and if so, prognostic disclosure may be perceived to cause distress^55^ or challenge a positive outlook.^39 76 91 92^ Therefore, providing patients with informational choice and ascertaining individual preferences^6 46 67 70 82 93 94^ is an inclusive approach, alongside consideration of sociocultural factors (CMOC1, 10).^52 53 56 68 75 81 95 96^ Respecting cultural or social norms and religious or spiritual needs is important (CMOC47, 48^56 58 62 63 96 97^) and may influence requests or preferences for prognostic information (CMOC46).^53 54 56 63 96 98^

A few studies suggested some patient groups are less likely to want prognostic information such as older patients, those with lower education, higher anxiety, shorter life-expectancy, and non-Northern European cultures.^53 75 86^ However, assumptions and stereotyping should be avoided,^46 86^ particularly as carer and clinician perceptions of patient preference may be inaccurate.^53 79 94^ A problematic situation can occur where patient and family/carer needs differ (CMOC39),^59 79 83 94^ for example, where families do not want patients to know the prognosis^57 58 75 79 88 96 99^ to protect them or sustain hope (CMOC10, 49, 50, 51).^75 79 96 99^ Such requests for concealment may challenge norms of autonomy and lead to professionals feeling conflicted.^57 88^ A few studies suggested that separate conversations with patients and families may be required (CMOC52),^53 83^ but erred towards respect for patient needs, consent, and confidentiality.^53 75 98^

#### Clinician training and experience

A key factor affecting whether discussions of prognostic uncertainty occur is the baseline clinician tolerance of uncertainty (CMOC 22, 26, 27, 29).^1 6 55 69 100 101^ This was influenced by the nature of the relevant illness or condition and clinicians’ experience.^102–105^ Many studies concur that clinicians may feel underprepared or lack training in how to approach and navigate prognostic discussions.^55 74 78 80 93 96 106–111^ As such, a lack of training and low confidence in communicating uncertainty were key issues in preventing timely and effective prognostic discussions from taking place (CMOC18, 20, 25).^44 50 67 89 112–116^ One of the specific training needs identified was skill in prognostication and addressing the tendency of clinicians to overestimate prognosis,^57 63 83 110 117^ whether or not they acknowledge uncertainty. A further frequently cited training need was communication skills training.^39 64 83 88 93 94 96 118 119^ Some studies report that clinicians with more experience may be more likely to initiate and engage in prognostic discussions,^57 114 120 121^ although evidence on this is conflicting.^114^ Nonetheless, training is needed to enable optimal uncertainty communication practices.^1 55^

#### Prognostic communication models and interventions

We identified many models to facilitate description of prognosis and interventions to facilitate its communication.^1 15 43 55 68 73 78 94 99 115 122–127^ These approaches often covered framing of prognostic uncertainty, for example, through categorical timeframe predictions,^90 117^ probabilities of risk,^123^ probabilistic estimates^43^ or likelihoods of death within specified timeframes,^90^ for example, ‘likelihood a patient will die in the next week or the next month’,^117^ and individualised survival estimates.^128^ Such tools may increase confidence in the degree of uncertainty surrounding a prognosis and so improve disclosure of prognostic information (CMOC17, 40);^115^ however, they cannot eliminate uncertainty inherent to prognostic estimates.^16 123^ Notably, prognostication and use and accuracy of tools may be affected by clinician experience,^117 129^ uncertainty tolerance (CMOC29),^69^ familiarity with the patient^115^ and speciality^117^; clinicians commonly overestimate prognosis,^52 117 130^ so tools such as the ‘surprise’ question^113^ tend to show limited accuracy, although they can promote a holistic approach.^113^

Communication models and interventions specifically addressing communication of uncertain prognosis included the best / worst case framework (CMOC6),^97 131 132^ question prompt lists or sheets,^46 90^ Serious Illness Conversation Guide,^68^ Psychosocial Assessment and Communication Evaluation (PACE),^1 40^ and checking understanding,^86 122^ and more specifically the ‘ask tell ask’ approach, whereby information known is elicited followed by information provision and checking understanding.^44 133^ This is pertinent as discrepancies between doctor’s perception of patient’s understanding and the patient’s perception of understanding are commonly identified.^46 53 79 90^ Gauging and clarifying a desire for information was also described as a ‘drip-feed’ communication strategy.^55^ Other communication strategies included combining verbal and written information^16 60^ (eg, numerical versus word-based estimates,^58 134 135^ breaking bad news strategies such as SPIKES (Setting, Perception, Invitation, Knowledge, Empathy and Strategy),^68 93 122^ balancing hoping for the best against worry^37^ or preparing for the worst.^47 76 109 136^ Colour-coded pictographs^48^ and graphics to represent risks were suggested as useful,^123^ though one study found that patients preferred words to diagrams.^58^

#### Timing and initiation of prognostic dialogue

Timing of prognostic conversations was widely referenced,^46 50 60 98 111 137–139^ with some healthcare professionals delaying prognostic discussions until the patient was closer to the end of their life and uncertainty was reduced (CMOC26).^39 63 66 80 89 109 124 140^ A patient appearing ‘well’ may increase clinician reluctance to initiate prognostic discussions.^38 39 44^ A person’s needs and preferences may change over time,^52 53 60 114^ so multiple opportunities for conversations and continuous communication is key^39 44 50 63 114 141 142^; the content and timing of the discussion may need negotiating,^46 55^ and the ‘right time’^64^ may vary between individuals.^39 64 108 143^ The suggested time point for prognostic conversations was often early in the course of illness^54 55 77 80 107 114 118 129 141 144^ i.e. at the time of or shortly after diagnosis^16 53 55 60 85 86^ so that patients are well enough to be active in conversation.^44^ Several studies cited patients, families and specialist palliative clinicians, who felt that prognostic uncertainty was raised too late^19 72 89 128 145–148^; this missed opportunity can cause regret for caregivers and may lead to unresolved bereavement narratives (CMOC7, 21).^72 146^

Some studies noted ethical and legal principles affecting initiation of discussions. In addition to promotion of autonomy, truth-telling and informed consent,^17 46 87^ it is a legal requirement in many countries that discussions about prognosis take place^46^ and are documented in the dying person’s care record.^56 119 149^ However, despite requirements for disclosure and documentation, prognostic discussions do not necessarily occur in practice, and the effect of prognostic uncertainty on discussions in this context is not known.^55 65 96 117 140^ There is a further risk, or perception of risk, that appearing unsure, or providing an inaccurate prognosis could cause patient or reputational harm.^130 150^ The ethical duty of preventing harm can therefore increase reluctance to initiate prognostic uncertainty discussions.^77 96^

Important considerations for initiating conversations about prognosis include establishing readiness,^55 134 135 151 152^ for example, recognising cues,^85^ as well as current understanding (CMOC1)^56 86 140 151 153 154^ and preferences,^72 94 109^ consent to disclose^64^ and asking how much they would like to know.^49 56 134 135 155 156^ The physical setting can also be pertinent (CMOC45).^86^ It must be safe, confidential and have appropriate people present.^55 56 62 75 86 157^ However, in practice such conversations can be either planned or spontaneous.^158^ In person was usually deemed most appropriate, 93not soon after surgery^60^ or by phone.^88^

Prognostic uncertainty conversations should ideally be led by clinicians with a good relationship and knowledge of the patient (CMOC8).^55 62 75 126 130 159^ Disclosure of prognostic uncertainty in this context may strengthen trust (CMOC12).^39 51 58 61 74 126^ In some families and cultures (eg, Chinese^79^), it may be normalised or preferred that patients are informed of their prognosis via family members^62 96^ and/or that the family decide what information is shared with the patient.^75 79 96^ Nonetheless, many studies argued that healthcare professionals are ultimately responsible for raising prognostic uncertainty.^46 66 74 85 89 129 142 160^ Professionals may, however, feel that another clinician or specialty may be better placed to lead the conversation.^77 89 156 161^ Ambiguity as to who should initiate a discussion can lead to hesitation and avoidance,^65 76 80 109 110 114 118 137 146^ especially in situations of poorly coordinated care. Healthcare professional attitudes and behaviours may vary by region or country,^75 96^ with some cultures deeming prognostic disclosure as prejudicial.^96^ Other factors affecting timely conversations were as follows: resource constraints, such as limited time (CMOC43),^63 96 106 113 115 162^ particularly during appointments^47 80 85 89 96 112^; healthcare professional reluctance (CMOC44)^46 65 71 140 163^; paternalistic medical culture^46^; fear of causing depression and distress^61 81 112^ or patient harm,^96 110^ for example, through taking away hope^78 81 106 115^; lack of private or suitable space for the discussion^116^; language barriers^57 62^; lack of healthcare communication skills and confidence in communicating uncertainty (CMOC18, 20)^44 50 89 112–115^; lack of training^55 78 106 107^; fear of damaging the clinician-patient relationship^120 134 135^ and notably uncertainty itself as a ‘barrier’ (CMOC27).^41 51 63 69 106 109 110 113 114 125 142 148 164 165^

The mechanism of healthcare professionals having difficulty in ‘managing their own emotions’ as a professional,^137^ as well as the impact of uncertainty on their professional identity and sense of competence and knowledge, could lead to the outcome of failing to raise prognostic uncertainty.^102 126^ One study suggested that perception of time taken to explain prognostic uncertainty may be greater than the actual time required.^106^ However, these discussions need to go ‘at the patient’s own pace’.^44^ There is a balance in a healthcare professional raising the topic of prognosis and uncertainty while retaining honesty and fostering hope^89 152^; for this reason, some healthcare professionals waited to discuss prognosis until asked by the patient (CMOC4).^39 44 50 76 85 89 96 113 145^ Discussions may also be indirectly initiated by healthcare professionals and then ‘made explicit by patients’^122^ or vice versa.^76 83 130^ On the contrary, some patients preferred, waited for, or expected clinicians to initiate,^39 44 50 64 74 89 109 156^ while other patients initiated and/or controlled such discussions.^39 75 76 130 152^ Moreover, there is evidence of relatives or companions requesting prognostic information or ‘time estimate requests’ to initiate the dialogue with the healthcare professional (CMOC4).^73 76 130 146 153^ There is a risk that if all parties wait for others to initiate, along with the other issues identified above, a conversation does not take place.^44 107 119 121^ Open and timely negotiation of who is responsible for initiating prognostic conversations is required.^121^

#### Content of communication

Tailoring the language used to the recipient and illness context with compassion is essential when discussing an uncertain prognosis.^64 85^ Some patients may prefer sensitivity and others may prioritise directness.^64^ Medical jargon without explanation, ambiguous language, and euphemisms should be avoided (CMOC32)^19 53 86 119 151 153^; health professionals are directed to use specific terms such as ‘death’ and ‘dying’,^86 153^ although language should be individualised, and in high context cultures, such directness may be less appropriate.^122^

These conversations also provide patients and their families opportunities to ask questions and talk about their fears.^56^ There was a tendency for patients and families to prefer clinicians to be honest about their prognosis and the uncertainty of this^43–46 106 166^; they often value clear, timely and consistent prognostic information about what is likely to happen and the expected disease progression and life expectancy.^62 94 108 114 130^ Although many patients may prefer explicit communication of statistics and survival likelihoods,^16 74–76 87^ others may prefer vagueness or ambiguity to minimise shock and distress, so an individualised or personalised approach is important.^46 70 73 79^

Closing the conversation should include summarisation of the discussion alongside signposting to resources and support,^167^ including planning for the future^55 130^ and symptom control,^168^ and any follow up.^93^ Some clinicians and patients or families prefer to end the discussion on ‘certainty’ or controllable topics such as pain management and reassurance about prioritisation of the patient’s comfort (CMOC23)^168^; this is sometimes referred to as emphasising the positive.^74 83 133 168^

#### Explicit disclosure of uncertainty and honest communication

A patient’s prognosis and individual disease progression is inherently uncertain^42 106 151 153 155^ and therefore clinicians tolerating and normalising^146^ this uncertainty is important for a patient-centred and family-centred approach^37 52^ and improving palliative and end-of-life care (CMOC3).^42 169^ Research evidence^37 55 75 96 98 112 151 153 170^ and guidance^82 166^ acknowledges the potential benefits of open, sensitive, and honest communication regarding a patient’s prognosis (eg, patient and family satisfaction,^19 52 58 73 78^ enhanced wellbeing,^61^ increased trust^19 43 52 61^ and understanding,^68 114^ including explicitly disclosing the uncertainty in predicting their prognosis).^171^ Honest and explicit communication is evidenced in a range of disease contexts such as heart failure,^89 166^ respiratory disease,^37^ advanced kidney disease,^109^ COPD,^107^ cancer (CMOC16),^16 172^ and stroke (CMOC19).^36 173^ Many, but not all,^58 171^ patients and families appreciate clear, truthful, and compassionate communication of prognosis, including discussion of uncertainty^43 151 153 173–175^ as this can be pivotal to inform decision making (CMOC2, 6, 14)^46 58 73^ in respect of a patient’s preferences, values, and end of life wishes.^36 55 56 63 105 110 142 165^

The uncertainty involved in prognostication, and risk of being wrong, may prompt health professional avoidance,^64 125 175^ but often patients do not expect health professionals to know exactly how long someone has left to live.^64 176^ Therefore, language recommended for acknowledging uncertainty, while being honest and informative, is to consider a range of possible scenarios (CMOC6), or to use indicative timeframes, eg, days, weeks, months or years^55 64 83 130 151 153 168 177^ rather than exact timeframes.^58 62 96 115 117 155^ Honesty about uncertainty in prognostic information does not mean clinicians necessarily declaring they ‘don’t know’,^151 153^ which may be detrimental to patient trust/confidence.^6 51^ Rather, healthcare professionals can highlight the irreducible nature of prognostic uncertainty alongside a clear and tailored explanation (CMOC37),^168^ while framing information with phrases such as ‘I think’^101 151 153 178^ and qualifiers/hedging ‘could’ ‘probably’.^122 153^ It is often helpful for patients and families to understand how and why prognosis is uncertain and why it may be estimated as ‘months rather than years’^64 130^ based on evidence such as explaining their signs, symptoms, and deterioration in an accessible manner^62 151 153 168^ without overestimation.^58 110 117^ For example, the phrase ‘sick enough to die’ is cited as an example of how to communicate death as a possible or probable outcome.^19 82^ Such communication and strategies should allow a clinician to explicitly state that a prognosis is uncertain without undermining their experience, training or role in the relationship (CMOC3).^42 153^ However, explicit disclosure of prognostic uncertainty in isolation is not enough;^6 55^ this must be coupled with skilled delivery,^61^ sensitivity^52 54 82 94 122^ and appropriate content of information^37^ in order not to trigger negative effects such as false hope or damaging the relationship.^62 64^ Clinicians should try to emphasise the value of supportive care available and non-abandonment,^55 76 83 179^ if possible, alongside acknowledging prognostic uncertainty and addressing the patient’s and families’ emotions,^146^ to make it known that such care and support can be continued (CMOC9, CMOC11).^52 83^

#### Implicit or avoided disclosure of information and uncertainty

Clinicians may not know what to say^121 164^ and may therefore avoid disclosing prognostic information to patients and carers (CMOC21).^43 63 64 108 125 140 152 174 175^ Two of the main reasons cited in the literature for such avoidance were prognostic uncertainty itself (CMOC27, 28)^109 113 114 121 161^ and fear of taking away hope.^1 63 78 81 106 115 152 171 174 180^ This healthcare professional avoidance or tacit communication may take several forms in practice including expressing uncertainty as certainty (CMOC22, 31, 38)^115 126^ (eg, to influence decision making),^101 110^ denial,^98^ indirect or vague terms,^74 126 181^ excessive optimism^74 83 110^ or avoidance of giving bad news (CMOC33),^44 80 88^ providing a lack of information (both quantity and quality)^144^ or lack of clarity (CMOC30),^75 144^ focusing on now or treatments^41^ rather than the future,^44 75 80^ delaying the disclosure^39 63 80 89 109 140^ or complete avoidance of the conversation altogether.^126 181^ Unless such avoidance was motivated by patients’ and carers’ preferences, it tended to trigger negative outcomes^96^ for patients and/or families such as feeling ill prepared for the future^71 144^/not knowing what to expect,^164^ lack of advance care planning (CMOC34, 42),^39 65^ frustration^60 138 144^ and anger,^64^ diminishing trust of clinicians,^64 140^ poorer satisfaction^53^ and isolation.^59 144^ Notably, clinician avoidance of talking about prognostic uncertainty may be driven by their own emotional discomfort or fear of causing distress to patients.^50 53 61 74 81 108–110 112 126 146 182^

#### Emotions, hope and distress

Hope was a central motivator affecting whether communication of prognostic uncertainty occurred and could have multiple and variable effects. A common contributing factor to clinician reluctance to disclose prognosis and prognostic uncertainty is the desire to preserve hope, and fear of causing harm, anxiety, upset or distress.^43 53 61 81 96 108 110 112 114 146^ Conversely, some evidence^65 85^ suggests that avoiding discussion of uncertainty can lead to false hope (CMOC 38), and also that patients *without* clear prognostic information and awareness may be ‘more likely to experience anxiety’^79^ and prognostic outcomes are not necessarily distressing^81 118^ or depressing^78 79 114^; however, there is mixed and contrasting evidence on these outcomes, particularly around distress.^63^ Furthermore, patients may be more harmed by the dishonesty of clinicians delaying, not asking or sharing their prognosis with them (CMOC24)^53 65 78 98^; this can lead to mistrust in healthcare professionals^72^ and depression, as well as unresolved bereavement narratives.^43 72 146^ Relatedly, another determinant of prognostic conversations is often clinician’s concern of ‘a loss of hope’,^44 62 69 78 81 93 106 115^ which can lead to introduction of prognostic uncertainty to promote (false) hope (CMOC33).^58^ So long as patients want to engage in prognostic discussions, there is limited evidence that communicating uncertainty decreases hope^73 114^ and indeed some may view uncertainty itself as ‘a source of hope’^43 51 100^ which may reduce anxiety^47^; therefore, clinician’s fears of taking away hope may often be unfounded (CMOC13).^73 96 114 174^ Despite this, there exists a paradox of patients and carers wanting to be informed, all the while fearing the future^1^ and the emotional impact of “knowing”^130^; this is complex for clinicians to navigate and requires clarity, skill and compassion.^72 78 100^ For example in Cagle et al. (2016),^62^ one carer expressed the sentiment that, *“regardless of the potential to take away hope, she would want to know”* (p.7).^62^

Figure 3 presents a more detailed description of the theory, illustrating the links between individual CMOCs and thematic areas.

**Figure 3:**
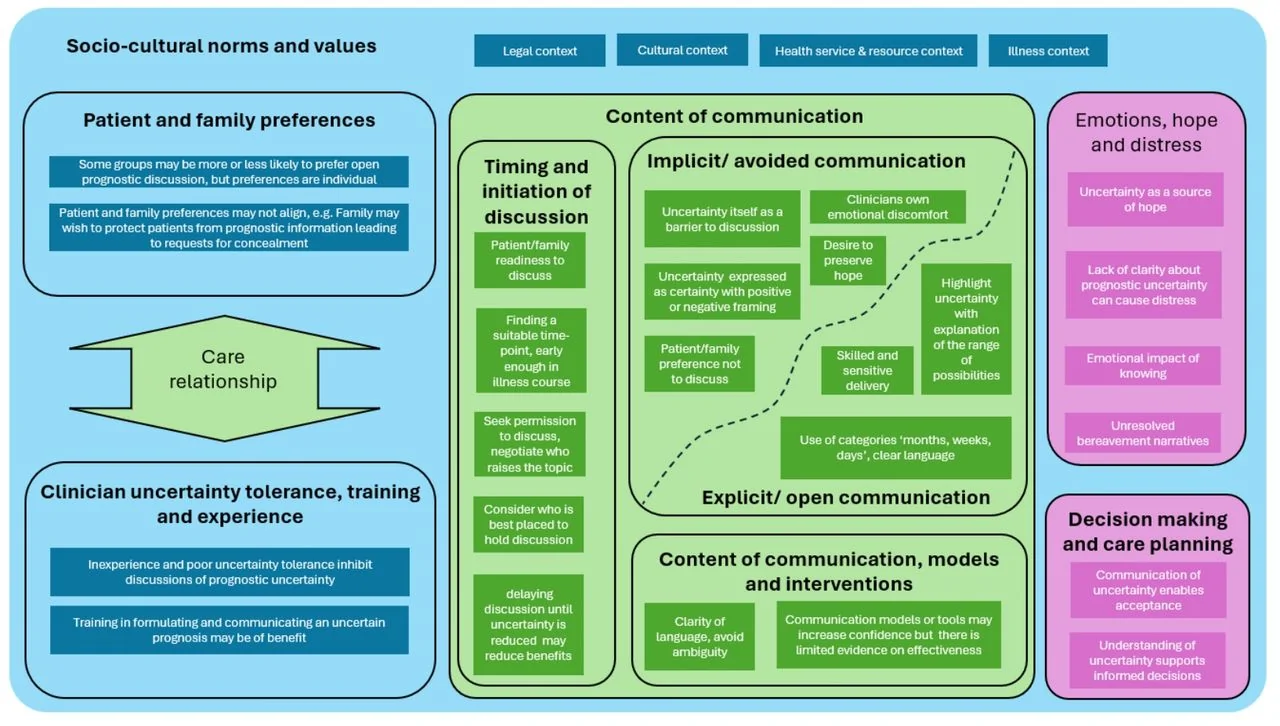
Detailed programme theory.

## Discussion

### Statement of principle findings

The programme theory developed in this review characterises contexts and mechanisms that influence whether and how prognostic uncertainty is communicated between healthcare professionals, patients and carers, and the outcomes affected by such communication. We found that when communicating an uncertain prognosis, the degree of explicitness appears to influence shared understanding and trust between clinicians and patients/carers, impacting experiences of distress, acceptance and future care planning. Key contextual factors including sociocultural norms and values, variation in individual communication preferences and clinician tolerance of uncertainty shape how communication occurs. Although widely recognised as important, there was limited evidence on how various sociocultural factors affect communication.

### Strengths and limitations

We reviewed extensive evidence including academic papers, guidelines and theses. We were therefore able to consider a range of advanced illness contexts: while many papers focused on malignancy, a variety of other illness and care contexts were included. The interdisciplinary research team and regular stakeholder input increased trustworthiness of the findings.

The stakeholder-informed realist approach involved patient and clinician perspectives throughout, alongside in-depth evidence synthesis. Stakeholders particularly contributed to generation of the programme theory and to refining the CMOCs. Communication, prognosis and uncertainty are complex and nuanced concepts; interrogating them through realist synthesis enabled us to go beyond description to develop an explanatory theory.^24^

In line with the realist approach, we aimed to synthesise sufficient literature to develop a programme theory rather than finding all existing relevant literature. The review was therefore not exhaustive. However, we are confident that our searches identified adequate literature to inform a robust theory. Despite the breadth of our search, which was not limited by language, most included articles originated from the USA, Europe and Australia, so perspectives from elsewhere, particularly from lower- and middle-income countries, could be under-represented. We were not able to search repositories of literature in other languages. We were not able to conduct full double screening of search results, but there was good agreement on the proportion that were double screened. It should also be noted that the included literature was in part of moderate or low quality, which limits the conclusions that can be drawn, and further empirical work to validate this theory may help to identify the most useful approaches to communication in specific contexts

### Comparison with existing literature

This is, to our knowledge, the first realist synthesis of evidence regarding communication of prognostic uncertainty. There is however an overlapping body of evidence on communication of prognosis, which identifies prognostic uncertainty as a problem in communication and care delivery.^96 183^ We extend the evidence base by interrogating prognostic uncertainty itself and unveiling the underlying mechanisms affecting its communication.

Literature reviews on prognostic communication in cancer support our findings, including the need for knowledge and expertise about the patient, to individually tailor disclosure and establish individual, nuanced patient preferences.^16^ While the complex interplay between honesty, hope and uncertainty is known,^16 75 184^ the theory presented here adds to existing evidence by explaining that relationship in the context of prognostic communication. Honesty and explicit yet sensitive disclosure of prognostic uncertainty may foster patient-clinician trust and, contrary to some clinicians’ fears, leaves space for hope. Findings by Clayton et al (2008) that hope can be derived from many different sources in advanced illness contexts indicate that accurate prognostic predictions are not patients’ sole sources of hope.^185^ Disclosure of prognostic uncertainty could therefore be reframed in a more nuanced way.

Work on prognostic disclosure indicates that sociocultural factors, such as cultural tendencies towards implicit disclosure and euphemism,^186^ (mis)perceptions that discussing death is (socially) a ‘taboo’,^187^ and paradigms of cultural collectivism^127^ influence preferences and attitudes towards prognostic information.^16 96 127^ The finding presented here adds to knowledge by highlighting the particular importance of sociocultural factors in shaping attitudes to communication of prognostic *uncertainty*, although with limited supporting empirical evidence. The implication is that assumptions or generalisations based on perceived cultural background should be avoided.

### Meaning and implications

This programme theory provides a novel understanding of the contexts and mechanisms that support effective communication of prognostic uncertainty in serious illness and palliative care, demonstrating the complexity and nuance behind such conversations.

The theory presented here can support professionals in individualised and compassionate communication of prognostic uncertainty to reduce distress, enhance trust, and enable shared care planning. While prognostic discussions are sometimes avoided or delayed until uncertainty is reduced, earlier and/or more transparent communication could improve decision making and timely access to services. Confidence can be a key limiting factor, so approaches that support healthcare professionals to develop confidence to engage with prognostic uncertainty by reflecting on their own experience with and tolerance of uncertainty will likely be of benefit. Supporting clinicians to understand and develop their tolerance of uncertainty may influence the occurrence and helpfulness of prognostic conversations. A standardised approach is unhelpful; flexibility and reflexivity could help professionals to communicate with sensitivity and respect for the individual and their sociocultural background.

### Unanswered questions and future research

Future research should focus on translation of these insights into clinician training and practice guidelines, exploring how this programme theory may inform the design and evaluation of approaches to care that integrate prognostic uncertainty, such as parallel planning for multiple possible health outcomes. We found little evidence regarding written communication of prognostic uncertainty and further research here is needed.

To help clinicians develop individualised and culturally appropriate approaches to prognostic communication, future implementation-focused empirical work should also explore how this theory applies in practice. In particular, we should explore how sociocultural factors affect communication of prognostic uncertainty in different geographical, cultural, and health system contexts, and how communication can be sensitively approached.

## Supplementary Material

Reviewer comments

Author's manuscript

## Data Availability

Data sharing not applicable as no datasets generated and/or analysed for this study. This was a synthesis of previously published work which is available online and referenced in the submission.
