# Supplementary material for: Improving communication of prognostic uncertainty in advanced illness: a realist review
Source: BMJ Open. 2026 Jul 1;16(7):e115385. doi: 10.1136/bmjopen-2025-115385 (PMC13663710; doi:10.1136/bmjopen-2025-115385)

Appendix 2. Initial programme theory

- Initial iterations of the draft theory (figure a) focused mainly on the clinical interaction itself and its outcomes.
- Discussions within the team and with the advisory group led to a broader lens, with addition of more contextual factors, particularly around family experiences, clinician factors, and the wider social context (figure b)

Figure a) early draft of initial theory

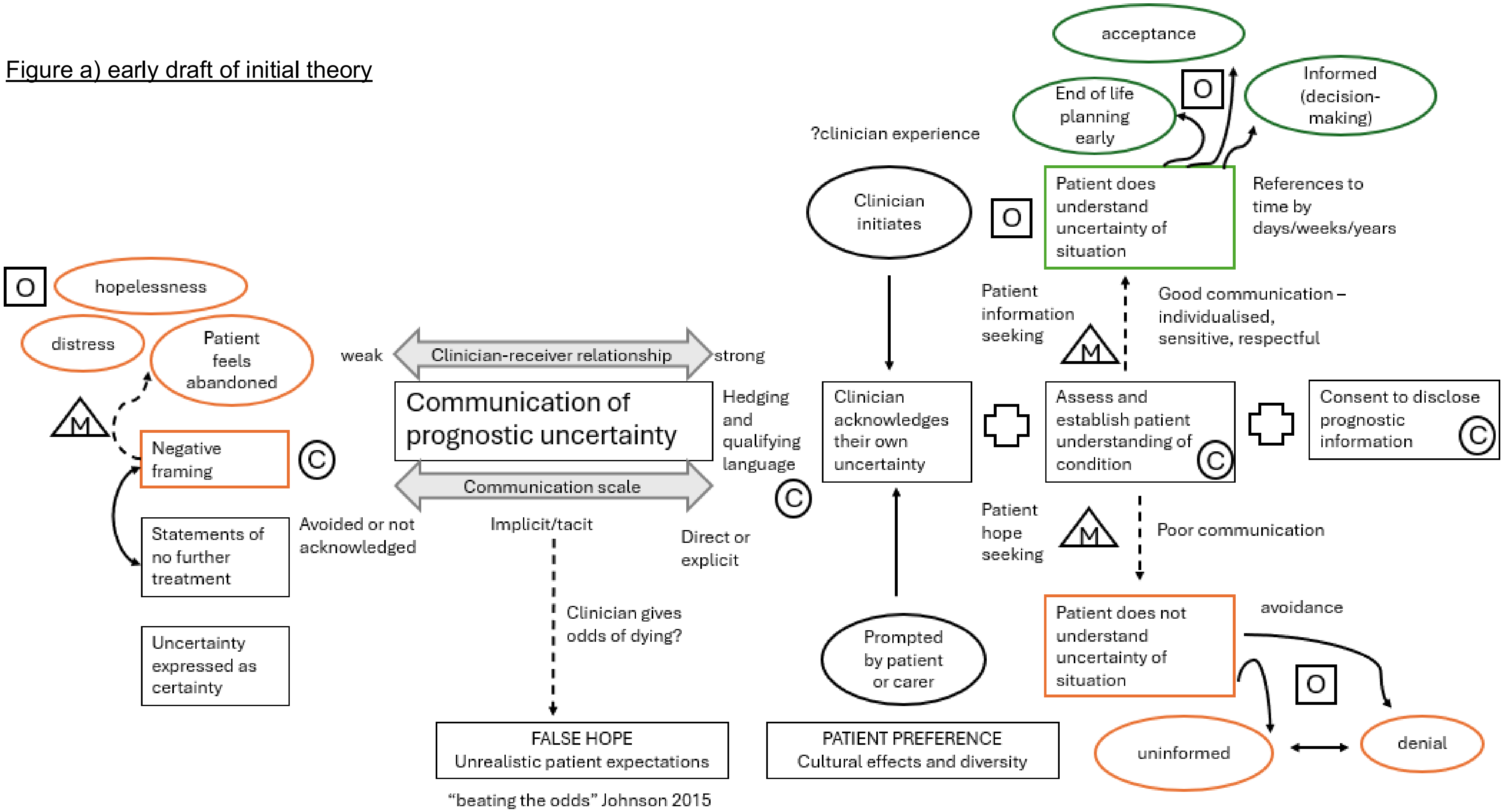

Figure b) agreed initial programme theory

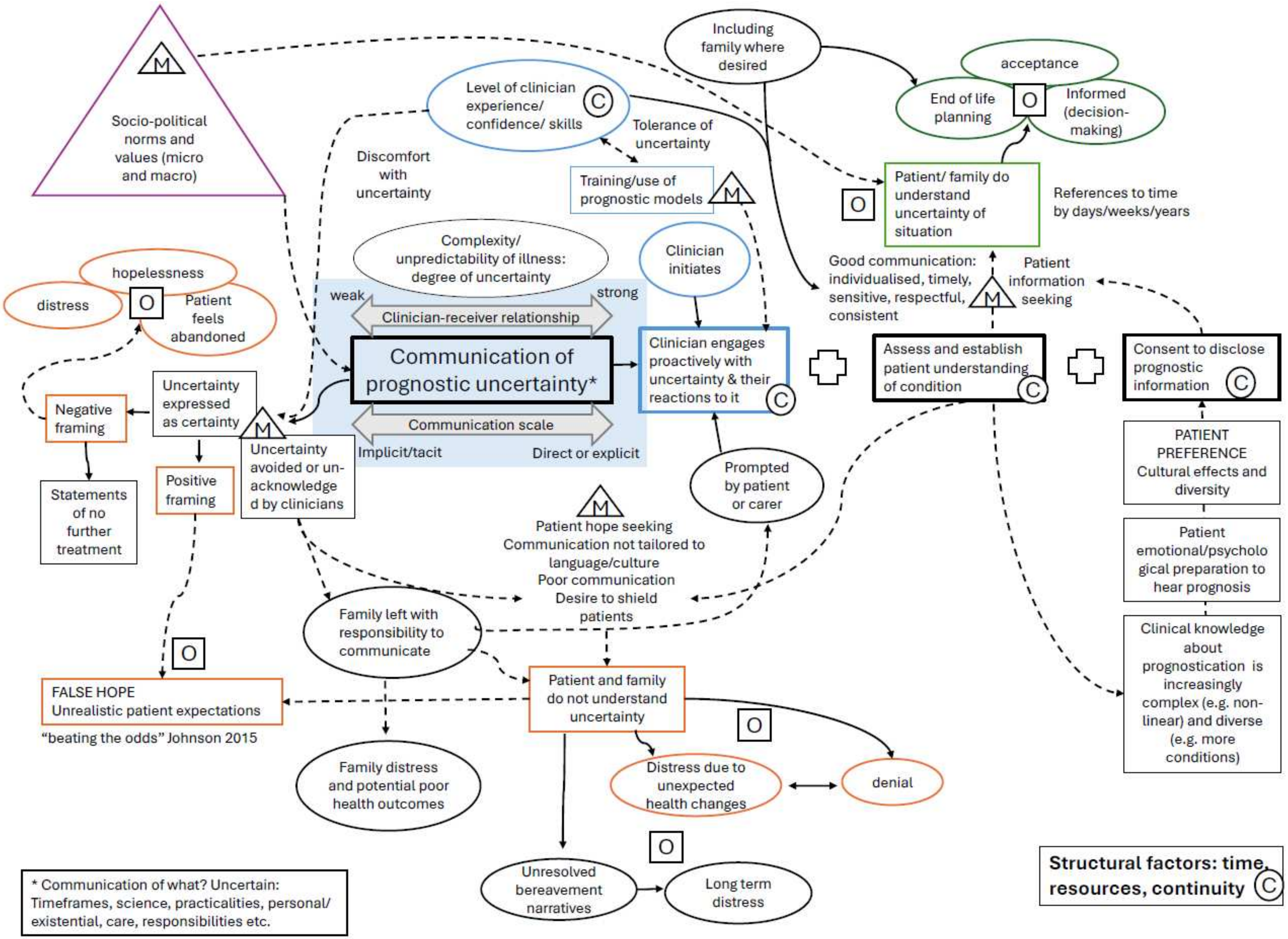

Supplement: Supplementary file 2 [file bmjopen-16-7-s002.pdf]
