# Supplementary material for: Improving communication of prognostic uncertainty in advanced illness: a realist review
Source: BMJ Open. 2026 Jul 1;16(7):e115385. doi: 10.1136/bmjopen-2025-115385 (PMC13663710; doi:10.1136/bmjopen-2025-115385)
Supplement: Supplementary file 3 [file bmjopen-16-7-s003.pdf]

MEDLINE via Ovid SP

Database: Ovid MEDLINE(R) ALL <1946 to August 15, 2024>

1 Communication Barriers/ or Health Communication/ or Nonverbal Communication/ or Teach-Back Communication/ or Communication/ or Disclosure/ or Truth Disclosure/ or Interpersonal Relations/ or Professional-Patient Relations/ or Nurse-Patient Relations/ or Physician-Patient Relations/ or Patient Discharge Summaries/ or (Communicat* or Conversation* or Discussing or Discussed or Discuss or Discusses or Disclos* or Dialog* or Nurse-Patient Relation* or Patient-Nurse Relation* or Doctor-Patient Relation* or Physician-Patient Relation* or Patient-Doctor Relation* or Patient-Physician Relation* or Patient-Health Care Professional Relation* or Patient-Health Professional Relation* or Patient-Health Professional Relation* or Patient-Healthcare Professional Relation* or Patient-Professional Relation* or Professional-Patient Relation* or Interpersonal or Patient Discharge Summar* or Patient Discharge Letter*).ti,ab. (2281379)

2 Prognosis/ or Life Expectancy/ or Disease Progression/ or Clinical Deterioration/ or (Prognos* or Predict* or Forecast* or Life Expectanc* or Disease Progress* or Disease Exacerbat* or Deteriorat*).ti,ab. (3456789)

3 Uncertainty/ or (Uncertain* or Indetermin* or Ambigu* or Unpredictab* or Vague* or Doubt*).ti,ab. (395891)

4 Palliative Care/ or Palliative Medicine/ or Hospice Care/ or Hospices/ or "Hospice and Palliative Care Nursing"/ or Terminal Care/ or Death/ or Bereavement/ or (Palliat* or Hospice* or Bereav* or Terminal* or End-of-Life or Death* or Advanced Illness* or Advanced Disease* or Life-Limiting or Die or Dying or Bereav*).ti,ab. (1810528)

5 and/1-4 (920)

Embase via Ovid SP

Database: Embase <1974 to 2024 August 16>

1 Interpersonal Communication/ or Communication Skill/ or Communication Barrier/ or Nonverbal Communication/ or Verbal Communication/ or Conversation/ or Written Communication/ or Professional-Patient Relationship/ or Nurse Patient Relationship/ or Doctor Patient Relationship/ or Patient Centered Communication/ or (Communicat* or Conversation* or Discussing or Discussed or Discuss or Discusses or Disclos* or Dialog* or Nurse-Patient Relation* or Patient-Nurse Relation* or Doctor-Patient Relation* or Physician-Patient Relation* or Patient-Doctor Relation* or Patient-Physician Relation* or Patient-Health Care Professional Relation* or Patient-Health Professional Relation* or Patient-Health Professional Relation* or Patient-Healthcare Professional Relation* or Patient-Professional Relation* or Professional-Patient Relation* or Interpersonal or Patient Discharge Summar* or Patient Discharge Letter*).ti,ab. (2667971)

2 Prognosis/ or Life Expectancy/ or Disease Exacerbation/ or Deterioration/ or (Prognos* or Predict* or Forecast* or Life Expectanc* or Disease Progress* or Disease Exacerbat* or Deteriorat* or Disease Aggravat*).ti,ab. (4642834)

3 Uncertainty/ or Intolerance of Uncertainty Scale/ or Mishel Uncertainty in Illness Scale/ or Mishel Uncertainty in Illness Theory/ or (Uncertain* or Indetermin* or Ambigu* or Unpredictab* or Vague* or Doubt* or Mishel*).ti,ab. (519031)

4 exp Palliative Therapy/ or Palliative Nursing/ or Hospice Care/ or Hospice/ or Hospice Patient/ or Hospice Nursing/ or Terminal Care/ or Terminally Ill Patient/ or exp Terminal Disease/ or Death/ or Dying/ or Bereavement/ or (Palliat* or Hospice* or Bereav* or Terminal* or End-of-Life or Death* or Advanced Illness* or Advanced Disease* or Life-Limiting or Die or Dying or Bereav*).ti,ab. (2504665)

5 and/1-4 (1753)

6 limit 5 to embase (764)

PsycINFO via Ovid SP

Database: APA PsycInfo <1806 to August 2024 Week 3>

1 Communication/ or Communication Barriers/ or Nonverbal Communication/ or Verbal Communication/ or Conversation/ or Written Communication/ or Communication Skills/ or Communication Skills Training/ or Interpersonal Interaction/ or Interpersonal Communication/ or (Communicat* or Conversation* or Discussing or Discussed or Discuss or Discusses or Disclos* or Dialog* or Nurse-Patient Relation* or Patient-Nurse Relation* or Doctor-Patient Relation* or Physician-Patient Relation* or Patient-Doctor Relation* or Patient-Physician Relation* or Patient-Health Care Professional Relation* or Patient-Health Professional Relation* or Patient-Health Professional Relation* or Patient-Healthcare Professional Relation* or Patient-Professional Relation* or Professional-Patient Relation* or Interpersonal or Patient Discharge Summar* or Patient Discharge Letter*).ti,ab. (1205127)

2 Prognosis/ or Life Expectancy/ or Disease Progression/ or (Prognos* or Predict* or Forecast* or Life Expectanc* or Disease Progress* or Disease Exacerbat* or Deteriorat*).ti,ab. (619978)

3 exp Uncertainty/ or "Ambiguity (Non-Stimulus)"/ or Doubt/ or (Uncertain* or Indetermin* or Ambigu* or Unpredictab* or Vague* or Doubt*).ti,ab. (130464)

4 Palliative Care/ or Hospice/ or Terminally Ill Patients/ or Terminal Cancer/ or "Death and Dying"/ or Bereavement/ or (Palliat* or Hospice* or Bereav* or Terminal* or End-of-Life or Death* or Advanced Illness* or Advanced Disease* or Life-Limiting or Die or Dying or Bereav*).ti,ab. (186761)

5 and/1-4 (261)

CINAHL via EBSCOhost

Limiters - Exclude MEDLINE records

(((MH "Communication") OR (MH "Communication Barriers") OR (MH "Communication Skills") OR (MH "Communication Skills Training") OR (MH "Conversation") OR (MH "Nonverbal Communication") OR (MH "Truth Disclosure") OR (MH "Professional-Patient Relations") OR (MH "Physician-Patient Relations") OR (MH "Nurse-Patient Relations") OR (MH "Interpersonal Relations") OR (MH "Patient Discharge Summaries")) OR TI (Communicat* OR Conversation* OR Discussing OR Discussed OR Discuss OR Discusses OR Disclos* OR Dialog* OR Nurse-Patient Relation* OR Patient-Nurse Relation* OR Doctor-Patient Relation* OR Physician-Patient Relation* OR Patient-Doctor Relation* OR Patient-Physician Relation* OR Patient-Health Care Professional Relation* OR Patient-Health Professional Relation* OR Patient-Health Professional Relation* OR Patient-Healthcare Professional Relation* OR Patient-Professional Relation* OR Professional-Patient Relation* OR Interpersonal OR Patient Discharge Summar* OR Patient Discharge Letter*) OR AB (

Communicat* OR Conversation* OR Discussing OR Discussed OR Discuss OR Discusses OR Disclos* OR Dialog* OR Nurse-Patient Relation* OR Patient-Nurse Relation* OR Doctor-Patient Relation* OR Physician-Patient Relation* OR Patient-Doctor Relation* OR Patient-Physician Relation* OR Patient-Health Care Professional Relation* OR Patient-Health Professional Relation* OR Patient-Health Professional Relation* OR Patient-Healthcare Professional Relation* OR Patient-Professional Relation* OR Professional-Patient Relation* OR Interpersonal OR Patient Discharge Summar* OR Patient Discharge Letter*)) AND (((MH "Prognosis") OR (MH "Life Expectancy") OR (MH "Disease Progression") OR (MH "Clinical Deterioration")) OR TI (Prognos* OR Predict* OR Forecast* OR Life Expectanc* OR Disease Progress* OR Disease Exacerbat* OR Deteriorat*) OR AB (Prognos* OR Predict* OR Forecast* OR Life Expectanc* OR Disease Progress* OR Disease Exacerbat* OR Deteriorat*)) AND (((MH "Uncertainty") OR (MH "Mishel Uncertainty in Illness Theory") OR (MH "Mishel Uncertainty in Illness Scale")) OR TI (Uncertain* OR Indetermin* OR Ambigu* OR Unpredictab* OR Vague* OR Doubt*) OR AB (Uncertain* OR Indetermin* OR Ambigu* OR Unpredictab* OR Vague* OR Doubt*)) AND (((MH "Palliative Care") OR (MH "Palliative Care Nurses") OR (MH "Palliative Care Nursing") OR (MH "Palliative Medicine") OR (MH "Hospice Nurses") OR (MH "Hospice Patients") OR (MH "Hospice Nursing") OR (MH "Hospice Care") OR (MH "Hospices") OR (MH "National Association for Home Care & Hospice") OR (MH "Hospice and Palliative Nurses Association") OR (MH "Hospice (Saba CCC)") OR (MH "Terminal Care") OR (MH "Terminal Care (Saba CCC)") OR (MH "Terminally Ill Patients") OR (MH "Death") OR (MH "Bereavement")) OR TI (Palliat* OR Hospice* OR Bereav* OR Terminal* OR End-of-Life OR Death* OR Advanced Illness* OR Advanced Disease* OR Life-Limiting OR Die OR Dying OR Bereav*) OR AB (Palliat* OR Hospice* OR Bereav* OR Terminal* OR End-of-Life OR Death* OR Advanced Illness* OR Advanced Disease* OR Life-Limiting OR Die OR Dying OR Bereav*))

214

Cochrane Database of Systematic Reviews

Issue 8 of 12, August 2024

Date Run: 18/08/2024 22:03:17

([mh "Communication Barriers"] or [mh "Health Communication"] or [mh "Nonverbal Communication"] or [mh "Teach-Back Communication"] or [mh Communication] or [mh Disclosure] or [mh "Truth Disclosure"] or [mh "Interpersonal Relations"] or [mh "Professional-Patient Relations"] or [mh "Nurse-Patient Relations"] or [mh "Physician-Patient Relations"] or [mh "Patient Discharge Summaries"] or (Communicat* or Conversation* or Discussing or Discussed or Discuss or Discusses or Disclos* or Dialog* or Nurse-Patient Relation* or Patient-Nurse Relation* or Doctor-Patient Relation* or Physician-Patient Relation* or Patient-Doctor Relation* or Patient-Physician Relation* or Patient-Health Care Professional Relation* or Patient-Health Professional Relation* or Patient-Health Professional Relation* or Patient-Healthcare Professional Relation* or Patient-Professional Relation* or Professional-Patient Relation* or Interpersonal or Patient Discharge Summar* or Patient Discharge Letter*):ti,ab) AND ([mh Prognosis] or [mh "Life Expectancy"] or [mh "Disease Progression"] or [mh "Clinical Deterioration"] or (Prognos* or Predict* or Forecast* or Life Expectanc* or Disease Progress* or Disease Exacerbat* or Deteriorat*):ti,ab) AND ([mh Uncertainty] or (Uncertain* or Indetermin* or Ambigu* or Unpredictab* or Vague* or Doubt*):ti,ab) AND ([mh "Palliative Care"] or [mh "Palliative Medicine"] or [mh "Hospice Care"] or [mh Hospices] or [mh "Hospice and Palliative Care Nursing"] or [mh "Terminal Care"] or [mh Death] or [mh Bereavement] or (Palliat* or Hospice* or Bereav* or Terminal* or End-of-Life or Death* or Advanced Illness* or Advanced Disease* or Life-Limiting or Die or Dying or Bereav*):ti,ab)

25

ProQuest Dissertations & Theses Global

18 August 2024 15:09

S1 title(Communicat* or Conversation* or Discussing or Discussed or Discuss or Discusses or Disclos* or Dialog* or Nurse-Patient Relation* or Patient-Nurse Relation* or Doctor-Patient Relation* or Physician-Patient Relation* or Patient-Doctor Relation* or Patient-Physician Relation* or Patient-Health Care Professional Relation* or Patient-Health Professional Relation* or Patient-Health Professional Relation* or Patient-Healthcare Professional Relation* or Patient-Professional Relation* or Professional-Patient Relation* or Interpersonal or Patient Discharge Summar* or Patient Discharge Letter*) OR abstract(Communicat* or Conversation* or Discussing or Discussed or Discuss or Discusses or Disclos* or Dialog* or Nurse-Patient Relation* or Patient-Nurse Relation* or Doctor-Patient Relation* or Physician-Patient Relation* or Patient-Doctor Relation* or Patient-Physician Relation* or Patient-Health Care Professional Relation* or Patient-Health Professional Relation* or Patient-Health Professional Relation* or Patient-Healthcare Professional Relation* or Patient-Professional Relation* or Professional-Patient Relation* or Interpersonal or Patient Discharge Summar* or Patient Discharge Letter*) 11884219

S2 title(Prognos* or Predict* or Forecast* or Life Expectanc* or Disease Progress* or Disease Exacerbat* or Deteriorat*) OR abstract(Prognos* or Predict* or Forecast* or Life Expectanc* or Disease Progress* or Disease Exacerbat* or Deteriorat*) 7103093

S3 title(Uncertain* or Indetermin* or Ambigu* or Unpredictab* or Vague* or Doubt*) OR abstract(Uncertain* or Indetermin* or Ambigu* or Unpredictab* or Vague* or Doubt*) 2471013

S4 title(Palliat* or Hospice* or Bereav* or Terminal* or End-of-Life or Death* or Advanced Illness* or Advanced Disease* or Life-Limiting or Die or Dying or Bereav*) OR abstract(Palliat* or Hospice* or Bereav* or Terminal* or End-of-Life or Death* or Advanced Illness* or Advanced Disease* or Life-Limiting or Die or Dying or Bereav*) 14366671

S5 [S1] AND [S2] AND [S3] AND [S4] 319

Science Citation Index-Expanded via Web of Science

Web of Science Search Strategy (v0.1)

1: Communicat* or Conversation* or Discussing or Discussed or Discuss or Discusses or Disclos* or Dialog* or Nurse-Patient Relation* or Patient-Nurse Relation* or Doctor-Patient Relation* or Physician-Patient Relation* or Patient-Doctor Relation* or Patient-Physician Relation* or Patient-Health Care Professional Relation* or Patient-Health Professional Relation* or Patient-Health Professional Relation* or Patient-Healthcare Professional Relation* or Patient-Professional Relation* or Professional-Patient Relation* or Interpersonal or Patient Discharge Summar* or Patient Discharge Letter* (Title) OR Communicat* or Conversation* or Discussing or Discussed or Discuss or Discusses or Disclos* or Dialog* or Nurse-Patient Relation* or Patient-Nurse Relation* or Doctor-Patient Relation* or Physician-Patient Relation* or Patient-Doctor Relation* or Patient-Physician Relation* or Patient-Health Care Professional Relation* or Patient-Health Professional Relation* or Patient-Health Professional Relation* or Patient-Healthcare Professional Relation* or Patient-Professional Relation* or Professional-Patient Relation* or Interpersonal or Patient Discharge Summar* or Patient Discharge Letter* (Abstract) Editions: WOS.SCI Date Run: Sun Aug 18 2024 16:16:01 GMT+0100 (British Summer Time) Results: 3442984

2: Prognos* or Predict* or Forecast* or Life Expectanc* or Disease Progress* or Disease Exacerbat* or Deteriorat* (Title) OR Prognos* or Predict* or Forecast* or Life Expectanc* or Disease Progress* or Disease Exacerbat* or Deteriorat* (Abstract) Editions: WOS.SCI Date Run: Sun Aug 18 2024 16:16:28 GMT+0100 (British Summer Time) Results: 4892944

3: Uncertain* or Indetermin* or Ambigu* or Unpredictab* or Vague* or Doubt* (Title) OR Uncertain* or Indetermin* or Ambigu* or Unpredictab* or Vague* or Doubt* (Abstract) Editions: WOS.SCI Date Run: Sun Aug 18 2024 16:17:05 GMT+0100 (British Summer Time) Results: 792328

4: Palliat* or Hospice* or Bereav* or Terminal* or End-of-Life or Death* or Advanced Illness* or Advanced Disease* or Life-Limiting or Die or Dying or Bereav* (Title) OR Palliat* or Hospice* or Bereav* or Terminal* or End-of-Life or Death* or Advanced Illness* or Advanced Disease* or Life-Limiting or Die or Dying or Bereav* (Abstract) Editions: WOS.SCI Date Run: Sun Aug 18 2024 16:17:26 GMT+0100 (British Summer Time) Results: 2533135

5: #4 AND #3 AND #2 AND #1 Editions: WOS.SCI Date Run: Sun Aug 18 2024 16:17:46 GMT+0100 (British Summer Time) Results: 1077

Google Scholar

#1 allintitle:Communicating|Communication Prognosis|Prognostic Uncertainty 7

#2 allintitle:Communicating|Communication Uncertainty Palliative|Hospice|"End of Life"|"Life Limiting" 6

7 + 6 = 12 (+ 1 duplicate)

Trip Database

PICO Search

Population: palliative OR "end of life"

Intervention: communicating OR communication

Outcome: (prognosis OR prognostic) AND uncertainty

8 results

NICE Guidelines

(Communicating OR Communication) AND (Prognosis OR Prognostic) Uncertainty AND (Palliative OR Hospice OR "End of Life" OR "Life Limiting")

20

Marie Curie via Google

(Prognosis OR Prognostic) Uncertainty site:mariecurie.org.uk

110

NHS Digital via Google

(Prognosis OR Prognostic) Uncertainty site:digital.nhs.uk

6

NHS Improvement via Google

(Prognosis OR Prognostic) Uncertainty site:improvement.nhs.uk

0

Royal College of Physicians via Google

(Prognosis OR Prognostic) Uncertainty site:rcp.ac.uk

19

General Medical Council via Google

(Communicating OR Communication) (Prognosis OR Prognostic) Uncertainty (Palliative OR "End of Life") site:gmc-uk.org

146

Department of Health via Google

intitle:(Communicating OR Communication) (Prognosis OR Prognostic) Uncertainty (Palliative OR "End of Life") site:gov.uk

273
