# Supplementary material for: Improving communication of prognostic uncertainty in advanced illness: a realist review
Source: BMJ Open. 2026 Jul 1;16(7):e115385. doi: 10.1136/bmjopen-2025-115385 (PMC13663710; doi:10.1136/bmjopen-2025-115385)
Supplement: Supplementary file 4 [file bmjopen-16-7-s004.pdf]

List of fields for data extraction:

1st Author

Year

Country

Title

Aim

Study design (primary data collection (qualitative, quantitative, mixed), literature review (systematic or narrative), policy document, guideline, thesis, book chapter other)

Health professionals, Patients, Carers? (describe if combination)

Patient population & diagnoses (details of study populatino)

Setting (inpatient, outpatient, community, primary care, details of specialty if given)

type of communication described(verbal, written, patient/carer-professional, interprofessional, mixed or multiple types)

communication intervention (describe if relevant)

number of participants (primary data collection only)

Rigour assessment score (high, moderate, low)

Relevance score (high, moderate, low)

Additional details (free text)
