# Supplementary material for: Improving communication of prognostic uncertainty in advanced illness: a realist review
Source: BMJ Open. 2026 Jul 1;16(7):e115385. doi: 10.1136/bmjopen-2025-115385 (PMC13663710; doi:10.1136/bmjopen-2025-115385)
Supplement: Supplementary file 5 [file bmjopen-16-7-s005.pdf]

Appendix 5. Details of included articles.

1st Author	Year	Country	Aim	Study design	HCP, Patients or Carers?	Patient population & diagnoses	Setting	Rigour	Relevance
						e.g. stroke, multimorbidity, frailty,			
Marie Curie	2014	UK	gain a deeper insight into the needs of terminally ill people and their non-professional carers, and understand experiences across the journey from diagnosis to death	Primary data collection - qualitative	Patients and carers	General Palliative care	Community	Moderate	Moderate
Marie Curie (Anderson)	2020	UK	to find the communication techniques that worked best at building awareness, helping decision making and closing the conversation.	Other	HCP + Patients + Carers	General medicine	All or mixed settings	Low	High
NICE	2015	UK	to improve end of life care for people in their last days of life by communicating respectfully and involving them, and the people important to them, in decisions and by maintaining their comfort and dignity	Policy document/guidance	N/A	last days of life	All or mixed settings	Moderate	Moderate
NICE	2018	UK	to improve diagnosis and treatment to increase the length and quality of life for people with heart failure	Policy document/guidance	N/A	heart failure	All or mixed settings	Moderate	Low
Alwidyan	2023	UK	to investigate healthcare professionals' barriers to and enablers of deprescribing in older hospice patients at the end of life and prioritise relevant theoretical domains for behaviour change to be incorporated into future interventions to facilitate deprescribing	Primary data collection - qualitative	HCP only	General palliative care - older patients	Hospice	High	Low
Anderson	2020	UK	To understand the practices used to accomplish prognostication and other relevant activities in conversations/communication between HCPs and relatives/friends of patients who are imminently dying	Thesis	HCP + Carers	General palliative care	Hospice	High	Moderate
Anderson	2020	UK	To understand how clinicians and the relatives/friends of patients at the very end of life manage uncertainty and reference time in prognostic conversations	Primary data collection - qualitative	HCP + Carers	General palliative care	Hospice	High	High
Anderson	2021	UK	To examine transitions out of prognostic talk in interactions between clinicians and the relatives and friends of imminently dying hospice patients.	Primary data collection - qualitative	Other	General palliative care	Hospice	High	Low

Anstey	2012	UK	To explore the personal and professional experience of consultant cardiologists, oncologists and old age psychiatrists and the consequent impact on referral practices to specialist palliative care services	Primary data collection - qualitative	HCP only	Cardiology, Oncology, Old-Age Psychiatry	Secondary care	Moderate	Low
Bailey	2018	UK	To offer advice and support to doctors to develop and improve in confidence when discussing their patients' preferences and values as they approach end of life	Policy document/guidance	HCP + Patients	Mixed - general medicine and palliative care	All or mixed settings	Moderate	Moderate
Barclay	2011	UK	To review the literature concerning conversations about EOLC between patients with heart failure and healthcare professionals, with respect to the prevalence of conversations; patients' and practitioners' preferences for their timing and content; and the facilitators and blockers to conversations.	Literature review	HCP + Patients + Carers	Heart failure	All or mixed settings	High	High
Baum	2020	USA	To provide commentary on a study (Seow et al) regarding potential utility of a patient-oriented prognostic tool	Literature review	N/A	Cancer	All or mixed settings	Low	Low
Bayuo	2021	Ghana	To explore the end of life care experiences of burn care staff and ascertain how their experiences can facilitate the development of clinical guidelines.	Primary data collection - qualitative	HCP only	Severe burns	Secondary care	High	Low
Becker	2020	Switzerland	To discuss communication challenges in EOL decision making and outline strategies from an area of growing interest and research	Literature review	Other	General palliative care	Other	Low	High
BGS	2020	UK	to support clinicians and others to consider the needs of frail older people as they move towards the end of their lives and help them to provide high quality care	Policy document/guidance	N/A	Frailty	Other	Low	Moderate
Bloomer	2018	Australia	to explore how end-of-life care goals and decision-making are communicated in a geriatric inpatient rehabilitation setting.	Primary data collection - qualitative	HCP only	Geriatrics, frailty	Community	Moderate	Moderate
Bloomer	2019	Australia	To investigate end-of-life care provision for older people in subacute care.	Primary data collection - quantitative observational	HCP + Patients + Carers	Geriatrics	Other	Moderate	Low
Borbasi	2021	Australia	To describe the experience and perspectives of residential aged care managers on providing end of life care for residents living with dementia.	Primary data collection - qualitative	HCP only	Dementia	Care Home	High	Moderate

Bristowe	2015	UK	To examine the experience of care supported by the AMBER care bundle compared to standard care in the context of clinical uncertainty, deterioration and limited reversibility.	Primary data collection - qualitative	Patients and carers	General medicine	Secondary care	Moderate	High
Broom	2014	Australia	To explore a range of medical specialists' experiences of referral to palliative care.	Primary data collection - qualitative	HCP only	General Palliative care	Community	Moderate	Low
Bruun	2024 a	UK	To explore how hospice multidisciplinary team (MDT) members oriented to the delicacy of prognostication during their meetings.	Primary data collection - qualitative	HCP only	General palliative care	Hospice	High	Moderate
Bruun	2024 b	UK	To explore how temporality is conveyed in relation to patients' prognoses during hospice multidisciplinary team meetings.	Primary data collection - qualitative	HCP only	General palliative care	Hospice	High	High
Burgers	2022	Netherlands	To understand the challenges in daily clinical practice experienced by HCPs from different disciplines who provide palliative as well as general care to AYAs (adolescents and young adults) with a UPCP (uncertain and/or poor cancer prognosis).	Primary data collection - qualitative	HCP only	Young adults with advanced cancer	All or mixed settings	High	Moderate
Cagle	2016	USA	To respond to the need for a greater understanding of caregiver preferences and attitudes toward being included in discussions about life expectancy	Primary data collection - qualitative	Carers only	Adults with disability age 65+	Community	High	High
Carlini	2022	Australia	To synthesise and critically analyse the points of discordance and concordance between consumers and clinicians about quality end-of-life care.	Literature review	HCP + Patients + Carers	Older patients aged >60	All or mixed settings	Moderate	Low
Cauley	2016	USA	To describe how surgeons approach treatment decisions to avoid nonbeneficial surgery, or engage in preoperative conversations about end-of-life (EOL) care, and to identify modifiable factors to reduce nonbeneficial surgery near the EOL.	Primary data collection - qualitative	HCP only	Older patients with surgical emergencies	Secondary care	Moderate	Low
Cavanagh	2020	USA	We review the literature concerning patient, caregiver, and clinician perspectives on prognosis communication in heart failure	Literature review	HCP + Patients + Carers	Heart failure	Other	Moderate	Low
Chan	2011	Hong Kong	To explore the relationships among the patient's awareness of the prognosis, the family's awareness of the prognosis, the patient's anxiety, and difficulty in communicating with family members.	Primary data collection - quantitative observational	Patients and carers	General palliative care	Secondary care	Low	Moderate

Chang	2014	USA	To examine prognosis discussions held with hospitalized patients for whom palliative care consultations were requested, and if prognosis discussions did not occur, to explore why not.	Primary data collection - qualitative + quantitative	HCP only	General palliative care	Secondary care	Moderate	Moderate
Chen	2018	UK	To assess the adequacy of community-based services available in Northern Ireland (NI) and to meet the multidimensional needs of patients living with heart failure (HF), as experienced and perceived by general practitioners (GP).	Primary data collection - qualitative	HCP only	Advanced heart failure	Primary care	Moderate	Low
Chen	2022	USA	To explore patients' perceptions of and preferences for communication surrounding goals of palliative RT and cancer prognosis.	Primary data collection - qualitative	Patients only	Oncology - bone or lung metastases	Secondary care	High	Low
Chou	2017	USA	Describes oncologists' language during discussions of prognosis and treatment goals in clinical interactions with African American patients diagnosed with cancer.	Primary data collection - qualitative	HCP + Patients	African Americans with stage III or IV cancer	Secondary care	High	High
Chow	2018	Canada	To identify the main challenges to the access and delivery of palliative care in patients with advanced congestive heart failure, and to summarize recommendations for clinical practice based on the available literature.	Literature review	HCP + Patients + Carers	Advanced heart failure	N/A	Moderate	Moderate
Christakis	1999	USA	To understand how and why physicians do and do not prognosticate	Literature review	HCP + Patients	Mixed	N/A	Moderate	Moderate
Christakis	1995	USA	To address the questions: what is the role of prognosis in medical thought and practice? Why is prognosis not as explicit a part of medicine as diagnosis and therapy?	Thesis	HCP + Patients + Carers	Mixed	Other	High	High
Chu	2019	UK	Narrative review discussing prognostication in Palliative Care	Literature review	HCP + Patients + Carers	General palliative care	All or mixed settings	Low	High
Chu	2020	UK	To evaluate the recent literature on prognostication for patients with advanced cancer and to suggest ways in which physicians can improve their own clinical practice in formulating and communicating prognostic estimates.	Literature review	HCP + Patients + Carers	Advanced cancer	All or mixed settings	Low	High

Clarke	2018	UK	To investigate how patients and their family members make decisions about their future care as their condition deteriorates, with a particular focus on mealtimes, eating and drinking	Primary data collection - qualitative	HCP + Patients + Carers	Progressive neurological disease	Community	High	Moderate
Cohen	2016	USA	To assess the effectiveness of an educational quality improvement intervention designed to enhance fellows' communication with patients who have advanced kidney disease.	Primary data collection - qualitative + quantitative	HCP only	Advanced kidney disease	Secondary care	Moderate	Moderate
Combes	2021	UK	To develop and refine an intervention to increase advance care planning engagement with older people living with frailty.	Thesis	HCP + Patients	Older people with frailty	Community	High	Low
Connolly	2021	USA	to conduct a qualitative metasynthesis to answer the following research question:What are the post-stroke PC/EOL experiences from the perspectives of patients, their families and healthcare professionals (HCPs)	Literature review	HCP + Patients + Carers	Stroke	All or mixed settings	High	Moderate
Cormican	2022	Ireland	To synthesize qualitative research evidence on the experiences of informal carers caring for a patient with a chronic haematological malignancy	Literature review	Carers only	Chronic haematological malignancy	All or mixed settings	High	Low
Cottrell	2020	UK	To develop and refine an initial theory on engagement in ACP for people with MS and to identify ways to improve its uptake for those who desire it.	Literature review	HCP + Patients + Carers	Multiple Sclerosis	All or mixed settings	High	Low
Creutzfeldt	2016	USA	To present current knowledge and recommendations regarding communication tasks and practice approaches for neurologists as they practice primary palliative care, including discussing serious news, managing symptoms, aligning treatment with patient preferences, introducing hospice/terminal care, and using the multiprofessional approach.	Literature review	HCP + Patients + Carers	Neurology	N/A	Low	Moderate
Crowther	2011	UK	To explore needs of people with dementia and their informal carers in the last year of life and surrounding death	Thesis	HCP + Patients + Carers	Dementia	Community	High	Moderate
Dias	2022	Portugal	To explore physicians' barriers in performing advanced care planning in patients with cancer; to propose a guide for discussion of advanced care planning for Brazilian physicians	Thesis	HCP + Patients	Oncology	Secondary care	High	Low

Dickerson	2024	USA	To characterize surgeon communication of pancreatic cancer prognosis using VitalTalk's ADAPT framework	Primary data collection - qualitative	HCP + Patients	Oncology - pancreatic cancer	Secondary care	Moderate	Moderate
Diggle	2022	UK	To present and synthesise the current evidence around bereaved caregivers' experiences of end of life care for people with heart failure	Literature review	Carers only	Heart failure	All or mixed settings	High	Low
Disler	2022	Australia, New Zealand	To explore attitudes and perceived barriers to ACP in self-identified palliative care nurses engaged and interested in care for patients with COPD.	Primary data collection - qualitative + quantitative	HCP only	COPD	All or mixed settings	Moderate	Low
Downer	2022	USA	Narrative review discussing communication about time-limited trials in the intensive care unit	Literature review	N/A	Intensive Care	Secondary care	Low	Moderate
Ekberg	2021	UK	Informs evidence-based communication about illness progression and end of life through a rapid review of studies that directly observe how experienced clinicians manage such discussions.	Literature review	HCP + Patients + Carers	General palliative care	All or mixed settings	Moderate	High
Epstein	2021	USA	Explore typical reactions to epistemic and complex uncertainty and offer preliminary proposals for how mindfulness and shared mind might help clinicians and patients navigate more effectively towards clear, health-promoting and values-concordant responses.	Literature review	HCP + Patients + Carers	Serious illness	All or mixed settings	Low	High
Etkind	2016	UK	To examine in detail the concept of uncertainty in illness and its impact on patients and professionals; to appraise current practice and evidence in addressing uncertainty in life-limiting illness	Literature review	HCP + Patients + Carers	Life-limiting illness	All or mixed settings	Moderate	High
Etkind	2025	UK	To co-design clinical practice recommendations for communication of prognostic uncertainty in older people with advanced multimorbidity, based on the lived experience and expertise of patients, carers, and healthcare professionals.	Primary data collection - qualitative	HCP + Patients + Carers	Advanced multimorbidity	Other	High	High
Evans	2009	USA	To understand surrogate decision makers' views regarding whether physicians should discuss prognosis in the face of uncertainty.	Primary data collection - qualitative	Carers only	Intensive Care	Secondary care	High	High
Fallowfield	2002	UK	Narrative review discussing communication of truth vs deceit in palliative care	Literature review	HCP + Patients + Carers	General palliative care	All or mixed settings	Low	Moderate

Fenton	2023	USA	To investigate cancer caregivers' communication experiences and potential impact on patient and caregiver outcomes.	Primary data collection - qualitative	Carers only	Advanced cancer	Secondary care	High	Moderate
Fisher	2012	Australia	Narrative review discussing uncertainty in end-of-life care and shared decision making	Literature review	HCP + Patients + Carers	Intensive Care	Secondary care	Low	High
Fitzsimons	2019	UK and Ireland	To explore caregivers" experience when caring for a loved one with advanced heart failure at the end of life and to identify any unmet psychosocial needs.	Primary data collection - qualitative	Carers only	Heart failure	Secondary care	High	Low
Formagini	2022	USA	To explore key factors that affect the strength of the stigma around palliative care referrals and its association with end of life in cancer care.	Primary data collection - qualitative	HCP + Patients + Carers	Oncology	Secondary care	Moderate	Low
Gadoud	2013	UK	To explore aspects of a palliative care approach for people with advanced HF: recognition of need, transitions in care and impact on patients, family carers and clinicians	Thesis	HCP + Patients + Carers	Heart failure	All or mixed settings	High	Moderate
Gallagher	2015	Brazil, England, Germany, Ireland, Palestine	To understand nurses' EoL decision-making practices in ICUs in different cultural contexts	Primary data collection - qualitative	HCP only	Intensive Care	Secondary care	High	Low
Garland	2013	Canada	To review the existing literature to identify barriers that inhibit end-of-life communication in the heart failure population.	Literature review	HCP + Patients + Carers	Heart failure	All or mixed settings	High	Moderate
Geerse	2019	USA	To characterize the content of serious illness conversations and identify opportunities for improvement	Primary data collection - qualitative	HCP + Patients	Oncology	Secondary care	High	Moderate
Gerlach	2019	Germany	To explore experiences, views and perceptions of haemato-oncologists on the use of the 'Surprise'-Question in the haemato-oncology outpatients clinics of a university hospital in Germany.	Primary data collection - qualitative	HCP only	Haematological malignancy	Secondary care	Moderate	Moderate
Gofton	2018	Canada	To develop a conceptual understanding of the specific characteristics of palliative care in neurology and the challenges of providing palliative care in the setting of neurological illness.	Primary data collection - qualitative	HCP + Patients + Carers	Neurological disease	Secondary care	Moderate	Low

Gonella	2022	Italy	To investigate how end-of-life communication may contribute to palliative-oriented care at the end-of-life in nursing homes according to both families' and nurses' perspective. Secondly, to identify the contextual factors internal to the nursing home that may influence the timing and quality of communication. Thirdly, to confirm the foundations for a first theory of end-of-life communication	Primary data collection - qualitative	HCP + Carers	Palliative care in care homes	Care Home	Moderate	Low
Goodlin	2008	USA	Narrative review applying the current knowledge about discussing prognosis and participatory decision-making to heart failure care.	Literature review	HCP + Patients + Carers	Heart failure	All or mixed settings	Low	High
Goss	2023	USA	To better understand surrogate experiences and needs during the period of active decision-making in SABI (severe acute brain injury), to inform interventions to support SABI patients and families and improve clinician-surrogate communication.	Primary data collection - qualitative	Carers only	Severe acute brain injury	Secondary care	High	High
Gough	2019	UK	To explore patient-centred accounts of the value and timing of prognostic discussions in advanced soft tissue sarcoma	Primary data collection - qualitative	Patients only	Advanced soft tissue sarcoma	Secondary care	Moderate	Moderate
Gramling	2013	USA	To identify and describe major types of prognosis conversations that occur in the natural setting of palliative care consultation.	Primary data collection - qualitative	HCP + Patients + Carers	General palliative care	Secondary care	High	Low
Gramling	2013 a	USA	To describe prognosis communication during PC consultation with seriously ill hospitalized patients.	Primary data collection - qualitative + quantitative	HCP + Patients + Carers	seriously hospitalised patients	Secondary care	Moderate	Moderate
Gray	2021	USA	To describe the current literature on illness and prognostic understanding in patients with haematologic malignancies, to examine the insights gained from literature about illness and prognostic understanding in patients with solid tumors, and to discuss future directions to inform our approach to studying illness and prognostic understanding in haematologic malignancies.	Literature review	HCP + Patients + Carers	Haematological malignancy	All or mixed settings	Moderate	Moderate
Green	2011	UK	To explore the perspectives of health care professionals on the transition of heart failure patients to a palliative care approach.	Primary data collection - qualitative	HCP only	Heart failure	All or mixed settings	Moderate	Moderate

Gutierrez	2010	USA	To explore the communication practices of prognostic information at end of life between and among critical care nurses, physicians, and family members in ICU throughout patients' trajectories, and the social, institutional, professional, and political influences which shape these practices	Thesis	HCP + Patients + Carers	Intensive Care	Secondary care	High	High
Gutierrez	2012	USA	Explore prognostic communication in ICU settings	Primary data collection - qualitative	HCP + Carers	Intensive Care	Secondary care	High	Moderate
Gutierrez	2013	USA	To identify the timing of and influences on critical care physician communication of negative prognoses to family members in an ICU.	Primary data collection - qualitative	HCP + Carers	Intensive Care	Secondary care	High	Moderate
Habib	2019	USA	To characterise reported practices and predictors of prognostic discussions for a cohort of hematologic oncologists.	Primary data collection - quantitative observational	HCP only	Haematological malignancy	Secondary care	High	Moderate
Habib	2021	USA	To characterize whether prognostic uncertainty has increased and if tolerance of uncertainty is associated with prognostic discussion practices in thoracic oncology.	Primary data collection - quantitative observational	HCP only	Lung cancer	Secondary care	Moderate	High
Hagerty	2005	Australia	To clarify the current available knowledge of patient preferences, clinician views and current practice regarding the communication of prognosis.	Literature review	HCP + Patients	Cancer	All or mixed settings	Moderate	High
Halkett	2010	Australia	To understand patients' experiences of High Grade Glioma and identify and describe information and support needs along the disease trajectory.	Primary data collection - qualitative	Patients and carers	High grade glioma	Secondary care	High	Moderate
Hallen	2015	USA	To explore physicians' perceptions of the value of Clinician Prediction Models in EOL care.	Primary data collection - qualitative	HCP only	General medicine	Secondary care	Moderate	High
Han	2016 a	USA	Reflective piece discussing prognostic disclosure and uncertainty	Other	N/A	General palliative care	N/A	Low	Moderate
Han	2016 b	USA	To explore the effects of personalized numeric prognostic information on physicians' intentions to communicate prognosis to cancer patients at the end of life, and to identify factors that moderate these effects.	Primary data collection -	HCP only	End-stage cancer	Primary care	Moderate	Moderate

				quantitative observational					
Hancock	2007	Australia	To conduct a systematic review of truth-telling in discussing prognosis with patients with progressive, advanced life-limiting illnesses and their caregivers.	Literature review	HCP + Patients + Carers	Advanced life-limiting illness	All or mixed settings	High	Moderate
Harrison	2019	Canada	To review how web-based prognosis tools for cancer patients and clinicians describe aleatory (risk estimates) and epistemic (imprecision in risk estimates) uncertainties.	Primary data collection - quantitative observational	N/A	Cancer	N/A	Moderate	Moderate
Harrison	2021	UK	To describe the palliative care needs and experiences of people with mesothelioma and their family carers.	Literature review	HCP + Patients + Carers	Mesothelioma	All or mixed settings	High	Low
Hendershot	2023	USA	To investigate the complexities and interplay between the concepts of prognostic uncertainty and patient preferences as they relate to the delivery of goal-concordant care to patients with severe acute brain injuries (SABI) in the (Neuro-ICU).Neurological Intensive Care Unit	Literature review	HCP + Patients + Carers	Severe acute brain injury	Secondary care	Low	High
Henselmans	2017	Netherlands	To examine how communication about life expectancy is initiated in consultations about palliative chemotherapy, and what prognostic information is presented.	Primary data collection - qualitative	HCP + Patients	Advanced cancer	Secondary care	High	High
Heyland	2009	Canada	To enrich our understanding of the relation between satisfaction with endoflife (EOL) care and discussions regarding the patient's prognosis.	Primary data collection - quantitative observational	Patients and carers	Advanced illness	Secondary care	Moderate	Low
Higginson	2013	UK	To develop and evaluate an interventional tool to improve communication and palliative care, using the ICU as an example of where this is difficult.	Other	HCP + Carers	Intensive Care Medicine	Primary care	High	Moderate
Hjelmfors	2014	Sweden	To describe HF nurses' perspectives on, and daily practice regarding, discussing prognosis and end-of-life care with HF patients in outpatient care. It further aims to explore barriers, facilitators and related factors for discussing these issues.	Primary data collection - quantitative observational	HCP only	Heart failure	All or mixed settings	Moderate	Moderate
Hjelmfors	2018	Sweden	To improve communication about the heart failure trajectory in patients, their families, and health care professionals	Thesis	HCP + Patients + Carers	Heart failure	All or mixed settings	High	Low

Hoff	2011	Sweden	To obtain a deeper understanding of how physicians are dealing with decision making and providing information to patients in situations where pertinent scientific knowledge is lacking or uncertain	Primary data collection - qualitative	HCP only	Oncology (Haematological and Lung)	Secondary care	Moderate	Moderate
Hoff	2014	Sweden	To identify inherent challenges in communicating information about imminent death.	Primary data collection - qualitative	HCP + Patients	Oncology (Haematological and Lung)	Secondary care	Moderate	High
Holloway	2013	USA	Narrative review discussing estimating and communicating prognosis in advanced neurologic disease	Literature review	HCP + Patients + Carers	Neurology	All or mixed settings	Low	High
Hudson	2017	UK	To explore the views and experiences of people who are homeless and those supporting them regarding conversations and approaches to palliative care.	Primary data collection - qualitative	HCP + Patients	General palliative care in patients experiencing homelessness	Community	High	Moderate
Hui	2019	USA	To provide an update on prognostication in patients with advanced cancer, and to discuss future directions for research in this field.	Literature review	HCP + Patients + Carers	Advanced cancer	All or mixed settings	Low	Moderate
Hui	2021	USA	Narrative review discussing the impact of prognostication on patient outcomes.	Literature review	HCP + Patients + Carers	Advanced cancer	All or mixed settings	Low	Moderate
Innes	2009	UK	To examine the research literature, to identify preferences for prognostic awareness in advanced cancer patients, to assess how much and what type of prognostic information cancer patients want in advanced disease and to identify any benefits that patients attribute to prognostic awareness.	Literature review	HCP + Patients + Carers	Advanced cancer	All or mixed settings	High	Moderate
Isaac	2017	USA	Editorial on discussing prognosis in the intensive care unit	Other	N/A	Intensive Care Medicine	Secondary care	Low	Moderate
Islam	2023	UK	To address the question: what are the barriers to and enablers of ethnically diverse patients, family caregivers and health-care professionals engaging in end-of-life care planning?	Primary data collection - qualitative	HCP + Patients + Carers	General palliative care	All or mixed settings	High	High
Jackson	2024	USA	Evidence supporting various communication strategies is presented, followed by a review of formal guidelines, when they exist	Literature review	N/A	Serious illness	All or mixed settings	Low	Moderate

Johnson	2012	USA	A case presentation of acute lung injury (ALI) progressing to acute respiratory distress syndrome (ARDS) requiring tracheal intubation and mechanical ventilation (ETMV) and narrative review discussing uncertainty in critical illness.	Literature review	HCP + Patients + Carers	Intensive Care Medicine	Secondary care	Low	High
Johnson	2015	UK	To explore the process of disclosure, patient experience and preferences for information regarding prognosis or risk of recurrence	Literature review	Patients only	Cancer	All or mixed settings	High	High
Jones	1983	USA	To determine the legal, professional, and ethical responsibilities of registered nurses as they make decisions about disclosure of prognostic information to dying adults	Thesis	HCP + Patients	Dying adults	All or mixed settings	Low	Low
Kantabanlan g	2023	UK	To provide an overview of current evidence on the experiences and palliative care needs of patients with hematologic malignancies and their families and the barriers and challenges of integrating palliative care into hematology care in Asia.	Literature review	HCP + Patients + Carers	Haematological malignancy	All or mixed settings	Low	Low
Kapo	2006	USA	Narrative review discussing prognosis in chronic diseases	Literature review	HCP + Patients + Carers	Chronic life-limiting illness	All or mixed settings	Low	Moderate
Kimbell	2014	UK	To broaden our understanding of the experience of living and dying with advanced liver disease, and to examine the utility of a qualitative longitudinal, multi-perspective methodology in end-of-life research.	Thesis	HCP + Patients + Carers	Advanced liver disease	All or mixed settings	High	High
Kimbell	2015	UK	To understand the experiences and support needs of people with advanced liver disease and those of their lay and professional carers to inform improvements in the supportive and palliative care of this rapidly growing but currently neglected patient group.	Primary data collection - qualitative	HCP + Patients + Carers	Advanced liver disease	All or mixed settings	High	Moderate
Ko	2020	USA	To explore decision-making experiences, related to utilisation of hospice care programmes from a retrospective perspective, with family caregivers (FCGs) in a rural US–Mexico border region.	Primary data collection - qualitative	Carers only	General palliative care	Community	Moderate	Low
Krawczyk	2016	Canada	To report on the concept of “communicating prognostic uncertainty” which emerged from a mixed methods survey asking family members to rank their satisfaction in seven domains of hospital end-of-life care.	Primary data collection - qualitative	Carers only	General palliative care	All or mixed settings	Moderate	High
Kupeli	2019	UK	To understand the similarities and differences between healthcare professionals' and family carers' perspectives on optimal end-of-life care,	Primary data collection - qualitative	Carers only	Advanced dementia	Care Home	Moderate	Moderate

			gain insights into how the interdependent roles of family carers and HCPs can be optimised, and make recommendations for policy and practice.						
Langmuir	2023	Canada	To review the literature on the experiences of uncertainty for individuals with advanced or metastatic cancer patients who are receiving immunotherapy or TT.	Literature review	Patients only	Advanced cancer	Secondary care	High	Moderate
LeBlanc	2018	USA	Narrative review discussing communicating prognosis in oncology	Literature review	N/A	Advanced cancer	N/A	Low	High
LeClaire	2005	USA	To determine whether the timing of prognostic information delivery by physicians is associated with caregiver satisfaction with communication or decision making in the ICU.	Primary data collection - quantitative observational	Carers only	Intensive Care	Secondary care	Moderate	Low
Lee	2022	Canada	Narrative review discussing uncertainty in surgical palliative care	Literature review	Other	Surgery	All or mixed settings	Low	Moderate
Low	2017	UK	To identify the limitations in palliative care provision in the last year of life for people with liver cirrhosis and potential barriers to and enablers of palliative care.	Primary data collection - qualitative	HCP + Patients	Advanced chronic liver disease	Secondary care	Moderate	Low
Maguire	1988	UK	Opinion article discussing handling uncertainty, collusion and denial when communicating with cancer patients	Other	N/A	Oncology	N/A	Low	Moderate
Mandel	2017	USA	To identify the barriers to serious illness conversations in the dialysis population, review best practices in and specific approaches to conducting serious illness conversations, and offer solutions to overcome barriers as well as practical advice, including specific language and tools, to implement serious illness conversations in the dialysis population.	Literature review	N/A	End-stage renal disease	All or mixed settings	Low	High
Masterson	2018	USA	To draw on the experiences of clinicians to clarify the concept of prognostic understanding and examine the mechanisms that contribute to patient understanding and uptake of prognostic information.	Primary data collection - qualitative	HCP only	Advanced cancer	Secondary care	Moderate	High
Maxwell	2020	USA	To examine hospitalized older adults' and family caregivers' receptivity to general (vs. individualized) prognostic information about frailty, injury, and one-year outcomes; and to determine information needs based on prognostic information.	Primary data collection - qualitative	Patients and carers	Frailty/older adults	Secondary care	Moderate	Low

McClung	2013	USA	To discuss specific clinical recommendations and an analysis of some of the ethical issues involved in the provision of care to elderly patients in the terminal stages of heart failure.	Literature review	HCP + Patients + Carers	Advanced heart failure	All or mixed settings	Moderate	Low
McDonald	2016	UK	Policy document on improving communication with patients in the National Health Service	Policy document/guidance	N/A	General palliative care	N/A	Moderate	Low
McLennon	2013	USA	To describe the frequency and types of ethical dilemmas experienced by oncology nurses caring for advanced cancer patients and to summarise their written comments about prognosis-related communications.	Primary data collection - qualitative	HCP only	Oncology	Secondary care	Moderate	Moderate
Medendorp	2021	Netherlands	To generate an overview of available recommendations on how to communicate uncertainty with patients during clinical encounters.	Literature review	N/A	General medicine	All or mixed settings	Moderate	High
Michael	2022	UK	This chapter covers the importance of cancer communication and strategies to aid practitioners in discussing recurrence, progression, prognostication, and transition to palliative care.	Book chapter	N/A	Advanced progressive disease	All or mixed settings	Low	Moderate
Moglia	2024	Italy	To investigate neurologists' attitudes toward prognostic communication and their knowledge and utilization of the ENCALS survival model in clinical practice.	Primary data collection - quantitative observational	HCP only	Amyotrophic Lateral Sclerosis	Secondary care	Moderate	High
Molzahn	2020	Canada	To describe how people diagnosed with heart failure and their family members describe uncertainty related to impending death.	Primary data collection - qualitative	Patients and carers	Advanced heart failure	Secondary care	High	High
Momen	2011	UK	To reviews the extent to which there is evidence that conversations with clinicians about end-of-life care occur for heart failure patients in practice.	Literature review	HCP + Patients	Heart failure	All or mixed settings	High	Moderate
Mone	2021	UK	To identify if there is concordance between actual prognosis and accurate prognostic awareness in individuals with advanced cancer, and to identify the rationale for any discordance between the two.	Literature review	N/A	Advanced cancer	N/A	High	Moderate
Mount	1980	Canada	Editorial discussing the psychological impact of urological cancer	Other	N/A	Oncology and general palliative care	Secondary care	Low	Moderate
Nadicksbernd	2023	UK	To explore professionals' perspectives of difficult conversations about life-limiting, deteriorating health and end-of-life.	Thesis	HCP only	General palliative care	Community	High	Moderate

Nagelschmidt	2020	Germany	To identify barriers that hinder or influence the discussion of end- of- life issues in the family context.	Literature review	Patients and carers	terminally ill adults	All or mixed settings	Moderate	Low
Narayan	2017	USA	To characterise heart failure patient preferences for, reactions to, and application of individualized SHFM prognostic information along with individualized SHFM (Seattle Heart Failure Model) prognostic information along with their understanding of uncertainty in population-based estimates.	Primary data collection - qualitative	Patients only	Heart failure	Secondary care	High	High
Ngwenya	2021	UK	To identify factors that impact communication and support and recommend ways to improve patients' understanding of living with life-threatening illness.	Primary data collection - qualitative	HCP + Patients + Carers	Lung cancer and COPD	Primary care	Moderate	High
Noble	2017	UK	To explore clinician views on decisional conflict in patients with end-stage kidney disease.	Primary data collection - qualitative	HCP only	End-stage renal disease	Secondary care	Moderate	Moderate
Olsson	2021	Australia	To explore existing guidelines around palliative care to increase current understanding of end-of-life communication processes applicable to the acute care setting.	Literature review	Other	end of life	Secondary care	Moderate	Low
Owusuaa	2021	Netherlands	To explore physicians' views on identifying and disclosing the last phase of life of patients with different illness trajectories.	Primary data collection - qualitative	HCP only	Cancer, COPD	All or mixed settings	Moderate	Moderate
Pardue	2020	USA	To apply Mishel's (1990) model of uncertainty in chronic illness to heart failure patients as a framework for clarifying the role uncertainty plays in end-of-life communication; to determine whether time perspectives play a role in heart failure patients' engagement in end-of-life communication; to elucidate the complex relationships between time perspective and uncertainty in illness and their association with end-of-life communication.	Primary data collection - quantitative observational	Patients only	Heart failure	All or mixed settings	Moderate	Moderate
Parker	2006	Australia	To conduct a systematic review regarding communication of prognosis and EoL issues with adults, and or their caregivers, with an advanced progressive life limiting illness.	Literature review	HCP + Patients + Carers	Advanced illness	All or mixed settings	High	High
Parker	2007	Australia	to review studies regarding prognostic/endof-life communication with adult patients in the advanced stages of a life-limiting illness and their caregivers	Literature review	HCP + Patients + Carers	Advanced illness	All or mixed settings	High	Moderate

Parry	2024	UK	To systematically collate, examine, and summarize what knowledge and insights have been established by Conversation Analysis research in palliative care and in other healthcare settings where there are interactions about the end of the patient's life	Literature review	N/A	General palliative care	N/A	Low	High
Parvez	2015	USA	To consider the concept of 'uncertainty' in clinical practice for end stage renal disease from physician and patient perspectives.	Literature review	HCP + Patients	End stage renal disease	All or mixed settings	Low	High
Payne	2010	UK	To identify patients' and family members' experiences of acute stroke and their preferences for end-of-life care.	Primary data collection - qualitative	Patients and carers	Acute stroke	Secondary care	Moderate	High
Peoples	2020	USA	To identify factors that influence how prognostic uncertainty is viewed by physicians, as it relates to their communications with families.	Primary data collection - qualitative	HCP only	General medicine/surgery	Secondary care	High	High
Perumalswami	2019	USA	To present an overview of the role of prognostic uncertainty in oncologists' emotions, why the landscape of prognostication for cancer is changing, and how certain medical advances that have the potential to induce a "Lazarus effect" for patients near death present daily ethical challenges for oncologists	Literature review	N/A	Oncology	Secondary care	Low	Moderate
Pfeil	2015	Germany	To explore the factors that hinder communication about the terminal nature of advance cancer, and to reconstruct how physicians and nurses in oncology perceive their roles in preparing patients for end-of-life (EOL) decisions.	Primary data collection - qualitative	HCP only	Advanced cancer	Secondary care	Moderate	Moderate
Piovesan	2024	Canada	To evaluate the usefulness, for patients and families, of knowing the prognosis during the last days of life and to identify its utility in planning the activities that are important to them during their remaining days.	Primary data collection - quantitative observational	Patients and carers	Hospice inpatients	Hospice	Moderate	Moderate
Pontin	2011	UK	To explore hospital specialist palliative care professionals' experience of prognostication.	Primary data collection - qualitative	HCP only	General palliative care	Secondary care	Moderate	High
Pooler	2018	Canada	To explore bereaved caregivers' experiences and perceptions of an early integrated palliative approach implemented at a Multidisciplinary Interstitial Lung Disease Clinic.	Primary data collection - qualitative	Carers only	Idiopathic Pulmonary Fibrosis	Secondary care	Moderate	Low

Powers	2011	USA	To illustrate distinctions and intersections of palliative care and end-of-life services through examples from case-centered data of older adults cared for during a four-year ethnographic study of an acute care hospital palliative care consultation service.	Primary data collection - qualitative	HCP + Patients + Carers	General palliative care	Secondary care	Low	Moderate
Pun	2023	Hong Kong	To synthesise empirical findings on the role of family in end- of- life (EOL) communication and to identify the communicative practices that are essential for EOL decision- making in family- oriented cultures.	Literature review	HCP + Patients + Carers	end of life care	All or mixed settings	Moderate	Moderate
Rafferty	2016	USA	To explore spouses' reasons for seeking and avoiding conversations regarding their partners' EOL care preferences, and examine whether certain demographic factors (eg, income, gender, age) more likely contributed to the initiation of EOL conversations.	Primary data collection - qualitative + quantitative	Carers only	End of life care	All or mixed settings	Moderate	Low
Reid	2015	UK	To explore healthcare professionals' views on delivering end-of-life care within an acute hospital trust	Primary data collection - qualitative	HCP only	General palliative care	Secondary care	Moderate	Moderate
Rhee	2013	Australia	To explore the perspectives of general practitioners (GPs) on factors influencing the implementation of ACPs.	Primary data collection - qualitative	HCP only	General practice	Primary care	High	Low
Richards	2013	UK	To explore if and how information about a transition to a palliative care approach was communicated to patients recently discharged from hospital and who fulfilled standardized criteria for palliative care need.	Primary data collection - qualitative	Patients and carers	General palliative care	Secondary care	Moderate	Moderate
Ridley	2013	Australia	Narrative review discussing uncertainty in end-of-life decision making	Literature review	N/A	Intensive care medicine	Secondary care	Low	High
Risco	2022	USA	To discuss the importance of prognostication and developing a prognostic mindset, as well as providing a framework for formulating prognosis and communicating "what to expect."	Literature review	N/A	Neurology	All or mixed settings	Low	High
Robinson	2021	New Zealand	To explore the impact of uncertainty on how death is remembered by bereaved family members of people with heart disease.	Primary data collection - qualitative	Carers only	Heart disease	All or mixed settings	Moderate	High
Rowe	2021	USA	To qualitatively explore the challenges presented by the COVID-19 pandemic from the perspective of palliative care clinicians.	Primary data collection - qualitative	HCP only	General palliative care	All or mixed settings	High	Low

Salins	2024	India	To address palliative care needs in the ICU by synthesising palliative care practices provided at end-of-life to critically ill patients and their families before, during, and after ICU admission.	Literature review	HCP + Patients + Carers	Intensive care	Secondary care	High	Moderate
Sanders	2023	Canada	To propose three methodological innovations to advance the basic science of serious illness communication.	Literature review	N/A	Serious illness	All or mixed settings	Low	Moderate
Scholz	2020	Australia	To synthesise clinicians' understandings of communication in end-of-life care.	Literature review	HCP only	Intensive care	All or mixed settings	Moderate	Low
Schuster	2012	USA	To evaluate whether and how physicians discuss prognostic uncertainty with surrogate decision makers of patients who are highly likely, but not certain, to die.	Primary data collection - qualitative + quantitative	HCP + Carers	Intensive care	Secondary care	Moderate	High
Schutz	2018	USA	Editorial discussing communication, personhood and uncertainty in neuropalliative care	Literature review	HCP + Carers	Neuropalliative care	N/A	Low	Low
Sedhom	2024	USA	Narrative review discussing person-centred supportive care in precision oncology	Literature review	HCP + Patients + Carers	Oncology	All or mixed settings	Low	High
Selman	2019	UK	To explore views and experiences of communication, information provision and treatment decision-making among older patients receiving conservative care.	Primary data collection - qualitative	Patients only	Older people with end-stage renal disease	Secondary care	High	Moderate
Sikhosana	2023	Australia	To synthesise the best available evidence on the experience of persons living with a life-limiting illness about expressing their voice during communication with healthcare professionals.	Literature review	Patients only	Life-limiting illness	All or mixed settings	High	Moderate
Simpkin	2019	USA	To provide a narrative review of studies relating to the communication of uncertainty, highlighting current strategies together with challenges and barriers, and outline a framework for future research.	Literature review	HCP + Patients + Carers	General	All or mixed settings	Low	High
Smith	2014		To examine ethical issues in disease prognosis and prognostication in palliative care from a clinical perspective.	Book chapter	N/A	General palliative care	All or mixed settings	Moderate	High
Stanek	2020	USA	(a) provide an analysis and clarification of the concept of goals of care, (b) identify the frequency, distribution, and domains of uncertainty expressed by patients with advanced cancer in their communication with	Thesis	Patients only	Advanced cancer	Secondary care	High	High

			the palliative care team using Han's taxonomy of uncertainty, and (c) describe the magnitude and precision of the association between types of expressed patient uncertainty and patients' self-rated prognosis.						
Stiel	2019	Australia	To explore the experiences of and communication techniques used by palliative care physicians as they consider and discuss nursing home placements for their patients.	Primary data collection - qualitative	HCP only	General palliative care	All or mixed settings	High	Low
Stocker	2014	UK	To explore experiences of giving or receiving a prognosis and managing the transition point from diagnosis to palliative and end of life care for those with heart failure.	Thesis	HCP + Patients + Carers	Heart failure	All or mixed settings	High	High
Stocker	2017	UK	To explore experiences of giving or receiving a prognosis and advanced palliative care planning for those with heart failure.	Primary data collection - qualitative	HCP + Patients + Carers	Heart failure	Secondary care	High	Moderate
Sullivan	2007	USA	To describe whether and when physicians report recognizing and communicating the imminence of death and identify potential barriers and facilitators to recognition and communication about dying in the hospital.	Primary data collection - qualitative	HCP only	General/terminal illness	Secondary care	Moderate	Moderate
Szawarski	2016	UK	Editorial discussing uncertainty in critical illness	Literature review	N/A	Critical illness	N/A	Low	High
Tarbi	2021	USA	To describe the existential dimensions of prognosis communication in naturally-occurring palliative care conversations.	Primary data collection - qualitative + quantitative	HCP + Patients + Carers	Advanced cancer	Secondary care	High	Moderate
Tavares	2019	UK	To understand COPD patients' preferences for palliative care discussions; and explore clinicians' opinions and experiences when holding these conversations.	Thesis	HCP + Patients	COPD	All or mixed settings	High	Moderate
Tavares	2020	UK	To understand the preferences of patients with chronic obstructive pulmonary disease for discussions about palliative and advance care planning with clinicians.	Primary data collection - qualitative	Patients only	COPD	All or mixed settings	High	Low
Temel	2016	USA	Narrative review discussing prognostic uncertainty in the modern era of cancer therapeutics	Literature review	N/A	Oncology	All or mixed settings	Low	High

Tetteh	2021	USA	To examine openness and topic avoidance in interpersonal communication about ovarian cancer.	Primary data collection - qualitative	Patients only	Ovarian cancer	All or mixed settings	High	Moderate
Tilburgs	2018	Netherlands	To determine the barriers and facilitators faced by GPs related to ACP with people with dementia.	Literature review	HCP + Patients + Carers	Dementia	Primary care	High	Low
Tolsa	2022	Switzerland	To explore (1) the perspectives and experiences of clinicians regarding the use of predictive scores for LST decision making in severe acute stroke, and (2) clinicians’ awareness of their own cognitive biases in this context.	Primary data collection - qualitative	HCP only	Severe acute stroke	Secondary care	High	Moderate
Tosh	2018	UK	Reflective article discussing conversations about death	Other	N/A	General/end-of-life	N/A	Low	Moderate
Trankle	2020	Australia	To complement our quantitative survey data by providing a greater understanding of the experiences and views of clinicians when engaging in EoL discussions.	Primary data collection - qualitative + quantitative	HCP only	General palliative care	All or mixed settings	High	Moderate
Tuck	2015	USA	To determine preferences of patients with Parkinson's Disease for timing and initiation of discussions regarding treatment, prognosis, advanced care planning, and end-of-life care options such as hospice.	Primary data collection - qualitative + quantitative	Patients only	Parkinson's Disease	Secondary care	High	Low
Udo	2018	Sweden	To explore palliative home care physicians’ experiences regarding end-of-life breakpoint communication	Primary data collection - qualitative	HCP only	General palliative care	Community	High	Moderate
van der Velden	2020	Netherlands	To offer up-to-date, evidence-based guidance on prognostic communication in palliative oncology.	Literature review	Patients only	Oncology	All or mixed settings	Moderate	Moderate
van der Velden	2021	Netherlands	To explore (1) how advanced cancer patients phrase their questions and ideas about prognosis; (2) how consulting oncologists discuss prognosis with advanced cancer patients; and (3) how advanced cancer patients and consulting oncologists discuss prognostic information previously provided by the referring oncologist.	Primary data collection - qualitative	HCP + Patients	Advanced cancer	Secondary care	High	High

van der Velden	2023	Netherlands	(1) describe the extent and direction of prognostic discordance, patients' prognostic information preferences in cases of prognostic discordance, and physicians' awareness of prognostic discordance; and (2) examine which patient, physician, and caregiver factors predict prognostic discordance.	Primary data collection - quantitative observational	HCP + Patients + Carers	Advanced cancer	Secondary care	High	Moderate
van der Velden	2024	Netherlands	To investigate the effects of prognostic communication strategies (prognostic disclosure vs. communication of unpredictability vs. non-disclosure; standard vs.standard and best-case vs.standard, best- and worst-case survival scenarios; numerical vs.word-based estimates) on prognostic perceptions, treatment decision-making and end-of-life anticipation in advanced cancer.	Primary data collection - quantitative observational	Other	Advanced cancer	All or mixed settings	Moderate	Moderate
van der Velden	2024 b	Netherlands	To investigate effects of prognostic communication strategies on emotions, coping, and appreciation of consultations in advanced cancer.	Primary data collection - quantitative observational	Other	Advanced cancer	All or mixed settings	Moderate	Moderate
van Eenennaam	2020	Netherlands	To develop a communication guide to support neurologists and rehabilitation physicians in discussing personalized prognosis, tailored to the individual needs and preferences of people with ALS and their families.	Policy document/guidance	HCP + Patients + Carers	Amyotrophic Lateral Sclerosis	All or mixed settings	Moderate	High
Vlckova	2020	Czech Republic	To synthesise the available literature on factors associated with accurate prognostic awareness in patients with cancer.	Literature review	HCP + Patients	Oncology	All or mixed settings	High	Moderate
Walczak	2013	Australia, USA	To explore patients' perspectives across two cultures (Australia and USA) regarding communication about prognosis and end-of-life care issues and to consider the ways in which these discussions can be optimised.	Primary data collection - qualitative	Patients only	Oncology	Secondary care	High	High
Walczak	2016	Australia	To identify and synthesise evidence for interventions targeting end-of-life communication.	Literature review	HCP + Patients + Carers	End-of-life care	All or mixed settings	High	Low
Waldrop	2012	USA	Guided by concepts from the Sense of Coherence Theory, which illuminates factors that contribute to adaptation in stressful situations, the objective of this study was to explore and describe family caregivers' accounts of the nature and timing of communication they had with a loved one's health care provider(s) during the advanced stages of cancer and before hospice enrollment.	Primary data collection - qualitative	Carers only	Advanced cancer	Hospice	High	Moderate

Wijsenbeek	2022	Netherlands	Consider the difficulties inherent in discussions with patients with IPF and their loved ones, and how clinicians might communicate with patients more effectively, from breaking the news about the diagnosis to providing support throughout the course of the disease.	Literature review	HCP + Patients + Carers	Idiopathic Pulmonary Fibrosis	All or mixed settings	Low	High
Wittenberg	2009	USA	Narrative review discussing estimating prognosis in end-stage renal disease	Literature review	HCP + Patients + Carers	End-stage renal disease	Secondary care	Low	Moderate
Wittenberg-Lyles	2008	USA	To understand the use and effectiveness of current communication protocols in terminal prognosis disclosures.	Primary data collection - qualitative	HCP + Patients + Carers	General palliative care	Secondary care	Low	Low
Wu	2024	China	To synthesise conversation analytic studies examining communication between healthcare professionals (HCPs) and patients/companions in the setting of palliative care.	Literature review	HCP + Patients + Carers	General palliative care	All or mixed settings	Moderate	High
Xie	2023	Canada	Narrative review discussing Artificial Intelligence decision support tools for end-of-life care planning conversations	Literature review	HCP + Patients + Carers	End-of-life care	All or mixed settings	Moderate	Low
Young	2024	New Zealand	To synthesise literature on patients in the last months of life due to a cancer diagnosis who have declined a referral to end-of-life care.	Literature review	Patients and carers	Cancer	All or mixed settings	High	High
Ziegler	2015	Australia	Reflective article discussing risk and prognosis	Other	N/A		N/A	Low	Moderate
