# Supplementary material for: Improving communication of prognostic uncertainty in advanced illness: a realist review
Source: BMJ Open. 2026 Jul 1;16(7):e115385. doi: 10.1136/bmjopen-2025-115385 (PMC13663710; doi:10.1136/bmjopen-2025-115385)
Supplement: Supplementary file 6 [file bmjopen-16-7-s006.pdf]

Appendix 6. Table of CMO constructs

	Context	Mechanism	Outcome
1	well trained, experienced clinician	Assess and establish patient understanding of condition	Good communication: individualised, timely, **sensitive**, respectful, consistent
2	well trained, experienced clinician	explicit/open communication of prognostic uncertainty	shared understanding of situation, sense of meaning, acceptance, facilitates care planning
3	clinician tolerates uncertainty	explicit/open communication of prognostic uncertainty	shared understanding of situation, sense of meaning, acceptance, facilitates care planning
4	patient/family/carer prompt prognostic discussion	Clinician engages proactively with uncertainty & their reactions to it	explicit/open communication of prognostic uncertainty
5	patient too unwell to communicate	honest/open communication of prognostic uncertainty with family	family satisfaction with end of life care
6	uncertain future illness trajectory	communication of a range of possible scenarios/repeated discussions	patient/family can prepare
7	patient or family unwilling to discuss uncertainty, prefer not to know	conversation not initiated	lack of understanding may lead to denial, distress, and even unresolved bereavement narratives for family, but can also enable patient to have hope and 'live in the moment'
8	strong care relationship, e.g. from relational continuity	collaboration/ working together to enable explicit/open/confident communication of prognostic uncertainty	shared understanding of situation, sense of meaning, acceptance, facilitates care planning
9	empathic care relationship	being available and appearing caring	patient and family feel supported despite uncertainty
10	differences in patient vs relative communication needs	degree to which communication is based on assessment of individual needs/preferences	differing communication needs are met
11	empathic care relationship	collaborative approach to understanding prognostic uncertainty	shared understanding of situation, acceptance, facilitates care planning

	Context	Mechanism	Outcome
12	poor previous communication	Acknowledgement of previous error, open acknowledgement of prognostic uncertainty	builds trust, avoids further conflict
13	complex illness situations	case conference with family	helps to explore and manage uncertainty, leaves room for hope, increases trust, supports decision making
14	treatment decision making in context of prognostic uncertainty	using the concept of 'practical certainty' i.e. 'we are as sure as we can be' during communication with patient and/or family	supports decision making whilst acknowledging inherent uncertainty
15	perceived expertise/authority of clinician delivering prognostic information	value that patient/family place on prognostic information	degree to which uncertainty is accepted/integrated
16	Uncertain prognosis in cancer care	having prognostic discussion with treating oncologist with whom patient has a prior relationship	greater prognostic awareness, hearing prognostic uncertainty from a senior clinician is perceived as more acceptable
17	baseline level of clinician expertise	clinician training/use of prognostic models	Clinician engages proactively with uncertainty & their reactions to it
18	knowledge of best practice but absence of training/experience/ strategies	clinicians anxious about holding conversations and avoid them	implicit/tacit/no communication of prognostic uncertainty
19	Acute illness with uncertain recovery, e.g. severe stroke	use prognostic tools as an aid + clinicians to recognise own biases regarding communicating prognostic uncertainty	improves decision making + clinician-patient/family relationship (especially where families prefer explicit communication of uncertainties)
20	inexperienced, poorly trained clinician	conversation not initiated with patient/family	family/informal caregivers may feel pressure to hold or communicate uncertainty themselves, leading to distress, disempowerment and regret
21	weak care relationship	implicit/tacit/no communication of prognostic uncertainty	lack of understanding, loss of trust, unresolved bereavement narratives

	Context	Mechanism	Outcome
22	patient or relative asks about prognosis	uncertainty may or may not be mentioned depending on clinician tolerance/experience/judgement	Variation in degree of understanding of prognostic uncertainty
23	emotional distress from uncertainty	moving from discussion of uncertainty to end on something where there is certainty	orients towards action and certainty, supports planning
24	Desire to shield patients	vague communication; Uncertainty avoided or un-acknowledged by clinicians	Patient and family do not understand prognostic uncertainty, may lead to greater distress
25	clinicians not confident of prognosis	do not engage in discussions about it	patients unaware what to expect, could cause anxiety or distress
26	Timing of discussion - delayed until near death	Clinician delays discussion until near to patient death to increase the certainty of prognostic information and avoid uncertainty	Family and patient informed late of prognosis/minimal time to process and plan due to clinician low tolerance of uncertainty
27	Prognostic uncertainty as barrier to discussion, often because of poor tolerance by clinician	Discussion avoided by healthcare professionals	Barrier to timely and appropriate ACP engagement
28	Uncertainty avoided or un-acknowledged by clinicians	Family left with responsibility to communicate	Family distress and potential poor health outcomes
29	clinician does not tolerate uncertainty	clinician delays conversation until prognostic uncertainty resolved	late communication of prognosis or prognostic uncertainty, low patient understanding, delayed decisions, however may be appropriate if information may be available soon
30	deteriorating patient	use of proxy constructs e.g. offering unscheduled hospital appointment to communicate short prognosis	lack of clarity can lead to misunderstandings, but may also foster hope
31	uncertainty presented as certainty by clinicians- positive framing	expected outcome does not occur	clinician feels distressed

	Context	Mechanism	Outcome
32	use of jargon/poor communication training etc	lack of clarity/prognostic uncertainty not communicated in a way that patients/families can understand	patient and family do not understand prognostic uncertainty
33	death is expected/in terminal phase	clinician introduces optimistic uncertainty to avoid giving bad news	false hope
34	advance care planning (ACP) discussion	Clinician concern that prognostic uncertainty means decision making is inappropriate	uncertainty is a barrier to ACP if using the ‘decision making’ model, but not if outlining future possibilities.
35	particularly unpredictable illness situation	challenging for clinicians to manage expectations in a changing situation	higher risk that patient and family do not understand uncertainty
36	finely balanced decision re aggressiveness of future care (e.g. in heart failure)	tacit/implicit/no communication of prognostic uncertainty	lack of understanding leading to aggressive care not in line with preferences.
37	different illnesses have different levels of uncertainty and reactions to it	tailor prognostic communication to nature of illness & trajectory	better prognostic awareness in illnesses that have more unpredictable trajectories
38	clinician does not tolerate uncertainty	uncertainty presented as certainty – positive or negative framing	false hope feelings of distress, hopelessness, abandonment
39	differences in patient vs relative communication needs	standardised communication	needs not met
40	no recognition of prognostic uncertainty	Communication frameworks and tools including AI nudge re potentially poor prognosis	increased likelihood of communication of prognosis when these tools are used
41	Unclear prognostic information and disclosure of uncertainty without explaining future possibilities	Patient perceives positive prognosis	Patient pursues unnecessarily aggressive treatments
42	ACP discussions	managing prognostic uncertainty by incorporating discussion of 'typical' illness trajectories	supports care planning in context of prognostic uncertainty
43	lack of time/resources, lack of access to clinicians	weak care relationship	implicit/tacit/no communication of prognostic uncertainty

	Context	Mechanism	Outcome
44	poor continuity of care	conflicting information from different providers	uncertainty (and associated distress) magnified, further discussions may be avoided for fear of introducing more conflicting information
45	variation in communication practices dependent on care environment/ culture (e.g. hospice vs hospital etc)	ability to make time for these discussions	explicit/open communication of prognostic uncertainty
46	socio-ethico-political norms and values	ability/willingness of patient/family to engage with prognostic information	level of understanding & ability to plan based on prognostic knowledge
47	patient/family sociocultural background	Communication not tailored to language/cultural preferences	patient and family do not understand prognostic uncertainty
48	cultural barriers to discussing uncertainty e.g. misconceptions that palliative care and dying are synonymous	clinicians less likely to raise uncertainty. focus on disease management rather than wider concerns	lack of communication/discussions delayed until uncertainty resolved
49	culture of avoiding explicit discussion of prognosis or uncertainty (high context communication)	family believe informing patient may harm their wellbeing	a) patient may have poor understanding of prognostic information, potentially causing anxiety and further uncertainty of situation b) cultural preferences are met
50	Patient/family spiritual/sociocultural background and views	Patient blocks or avoids discussion of prognosis and uncertainty "keeping it positive"	Discussion is delayed until patient is nearer end of life/ prognosis is more certain and short, resulting in more limited scope for care planning
51	Patient/family spiritual/sociocultural background and views	Family requests that patient is not given prognostic information within certain cultural contexts	Patient has no understanding or knowledge of prognosis - family may have been told separately
52	patient who lacks capacity to contribute to discussions e.g. advanced dementia	Degree of family awareness of patients wishes	Family awareness of patient wishes affects how easy it is to discuss prognosis or end of life care, which may affect understanding of uncertain illness trajectory
