# Supplementary material for: Improving communication of prognostic uncertainty in advanced illness: a realist review
Source: BMJ Open. 2026 Jul 1;16(7):e115385. doi: 10.1136/bmjopen-2025-115385 (PMC13663710; doi:10.1136/bmjopen-2025-115385)
Supplement: Supplementary file 7 [file bmjopen-16-7-s007.pdf]

References for all included articles

1. Alwidyan T, McCorry NK, Parsons C. Healthcare professionals' perspectives of deprescribing in older patients at the end of life in hospice care: a qualitative study using the Theoretical Domains Framework. Int J Pharm Pract. 2023;31(3):305-13.
2. Anderson RJ. Communication with the Relatives and Friends of Imminently Dying Patients in a Hospice. England: University of London, University College London (United Kingdom); 2020.
3. Anderson RJ, Stone PC, Low JTS, Bloch S. Managing uncertainty and references to time in prognostic conversations with family members at the end of life: A conversation analytic study. Palliat Med. 2020;34(7):896-905.
4. Anderson RJ, Stone PC, Low JTS, Bloch S. Transitioning out of prognostic talk in discussions with families of hospice patients at the end of life: A conversation analytic study. Patient Educ Couns. 2021;104(5):1075-85.
5. Anstey S. Clinical thinking, knowledge and the referral practices of consultant physicians to specialist palliative care services: an interpretative phenomenological analysis. Wales: Cardiff University (United Kingdom); 2012.
6. Bailey SJ, Cogle K. Talking about dying: How to begin honest conversations about what lies ahead: Royal College of Physicians; 2018.
7. Barclay S, Momen N, Case-Upton S, Kuhn I, Smith E. End-of-life care conversations with heart failure patients: a systematic literature review and narrative synthesis. Br J Gen Pract. 2011;61(582):e49-62.
8. Baum LVM, Friedman D. The Uncertain Science of Predicting Death. JAMA Netw Open. 2020;3(4):e201736.
9. Bayuo J, Bristowe K, Harding R, Agbeko AE, Wong FKY, Agyei FB, et al. "Hanging in a balance": A qualitative study exploring clinicians' experiences of providing care at the end of life in the burn unit. Palliat Med. 2021;35(2):417-25.
10. Becker C, Beck K, Vincent A, Hunziker S. Communication challenges in end-of-life decisions. Swiss Med Wkly. 2020;150:w20351.
11. Bgs. End of Life Care in Frailty: Identification and prognostication. CLINICAL GUIDELINES. 2020.
12. Bloomer MJ, Botti M, Runacres F, Poon P, Barnfield J, Hutchinson AM. Communicating end-of-life care goals and decision-making among a multidisciplinary geriatric inpatient rehabilitation team: A qualitative descriptive study. Palliat Med. 2018;32(10):1615-23.
13. Bloomer MJ, Botti M, Runacres F, Poon P, Barnfield J, Hutchinson AM. End-of-life care for older people in subacute care: A retrospective clinical audit. Collegian. 2019;26(1):22-7.
14. Borbasi JAL, Tong A, Ritchie A, Poulos CJ, Clayton JM. "A good death but there was all this tension around"- perspectives of residential managers on the experience of delivering end of life care for people living with dementia. BMC Geriatr. 2021;21(1):306.
15. Bristowe K, Carey I, Hopper A, Shouls S, Prentice W, Caulkin R, et al. Patient and carer experiences of clinical uncertainty and deterioration, in the face of limited reversibility: A comparative observational study of the AMBER care bundle. Palliat Med. 2015;29(9):797-807.
16. Broom A, Kirby E, Good P, Wootton J, Adams J. The troubles of telling: managing communication about the end of life. Qual Health Res. 2014;24(2):151-62.

17. Bruun A, White N, Oostendorp L, Stone P, Bloch S. Time estimates in prognostic discussions: A conversation analytic study of hospice multidisciplinary team meetings. Palliat Med. 2024;38(5):593-601.
18. Bruun A, White N, Oostendorp L, Stone P, Bloch S. Prognostication As an Interactionally Delicate Matter: A Conversation Analytic Study of Hospice Multidisciplinary Team Meetings. Health Commun. 2025;40(6):956-64.
19. Burgers VWG, van den Bent MJ, Darlington AE, Gualtherie van Weezel AE, Compter A, Tromp JM, et al. A qualitative study on the challenges health care professionals face when caring for adolescents and young adults with an uncertain and/or poor cancer prognosis. ESMO Open. 2022;7(3):100476.
20. Cagle JG, McClymont KM, Thai JN, Smith AK. "If You Don't Know, All of a Sudden, They're Gone": Caregiver Perspectives About Prognostic Communication for Disabled Elderly Adults. J Am Geriatr Soc. 2016;64(6):1299-306.
21. Carlini J, Bahudin D, Michaleff ZA, Plunkett E, She EN, Clark J, et al. Discordance and concordance on perception of quality care at end of life between older patients, caregivers and clinicians: a scoping review. Eur Geriatr Med. 2022;13(1):87-99.
22. Cauley CE, Block SD, Koritsanszky LA, Gass JD, Frydman JL, Nurudeen SM, et al. Surgeons' Perspectives on Avoiding Nonbeneficial Treatments in Seriously Ill Older Patients with Surgical Emergencies: A Qualitative Study. J Palliat Med. 2016;19(5):529-37.
23. Cavanagh CE, Rosman L, Spatz ES, Fried T, Gandhi PU, Soucier RJ, et al. Dying to know: prognosis communication in heart failure. ESC Heart Fail. 2020;7(6):3452-63.
24. Chan WC. Being aware of the prognosis: how does it relate to palliative care patients' anxiety and communication difficulty with family members in the Hong Kong Chinese context? J Palliat Med. 2011;14(9):997-1003.
25. Chang A, Datta-Barua I, McLaughlin B, Daly B. A survey of prognosis discussions held by health-care providers who request palliative care consultation. Palliat Med. 2014;28(4):312-7.
26. Chen JJ, Gamble K, Graham-Wisener L, McGlade K, Doherty J, Donnelly P, et al. GP perceptions of the adequacy of community-based care for patients with advanced heart failure in a UK region (NI): a qualitative study. Open Heart. 2018;5(1):e000734.
27. Chen JJ, Roldan CS, Nichipor AN, Balboni TA, Krishnan MS, Revette AC, et al. Prognostic Understanding and Goals of Palliative Radiotherapy: A Qualitative Study. J Pain Symptom Manage. 2022;64(6):567-76.
28. Chou WS, Hamel LM, Thai CL, Debono D, Chapman RA, Albrecht TL, et al. Discussing prognosis and treatment goals with patients with advanced cancer: A qualitative analysis of oncologists' language. Health Expect. 2017;20(5):1073-80.
29. Chow J, Senderovich H. It's Time to Talk: Challenges in Providing Integrated Palliative Care in Advanced Congestive Heart Failure. A Narrative Review. Curr Cardiol Rev. 2018;14(2):128-37.
30. Christakis NA. Prognostication and bioethics. Daedalus. 1999;128(4):197-214.
31. Christakis NA, Fox RC. Prognostication and death in medical thought and practice. United States -- Pennsylvania: University of Pennsylvania; 1995.
32. Chu C, Anderson R, White N, Stone P. Prognosticating for Adult Patients With Advanced Incurable Cancer: a Needed Oncologist Skill. Curr Treat Options Oncol. 2020;21(1):5.

33. Chu C, White N, Stone P. Prognostication in palliative care. Clin Med (Lond). 2019;19(4):306-10.
34. Clarke G, Fistein E, Holland A, Tobin J, Barclay S, Barclay S. Planning for an uncertain future in progressive neurological disease: a qualitative study of patient and family decision-making with a focus on eating and drinking. BMC Neurol. 2018;18(1):115.
35. Cohen RA, Jackson VA, Norwich D, Schell JO, Schaefer K, Ship AN, et al. A Nephrology Fellows' Communication Skills Course: An Educational Quality Improvement Report. Am J Kidney Dis. 2016;68(2):203-11.
36. Combes S. Conversations on Living and Dying: Facilitating Advance Care Planning with Older People Living with Frailty. Developing and Refining an Intervention. England: University of London, King's College (United Kingdom); 2021.
37. Connolly T, Coats H, DeSanto K, Jones J. The experience of uncertainty for patients, families and healthcare providers in post-stroke palliative and end-of-life care: a qualitative meta-synthesis. Age Ageing. 2021;50(2):534-45.
38. Cormican O, Dowling M. Providing Care to People Living with a Chronic Hematological Malignancy: A Qualitative Evidence Synthesis of Informal Carers' Experiences. Semin Oncol Nurs. 2022;38(6):151338.
39. Cottrell L, Economos G, Evans C, Silber E, Burman R, Nicholas R, et al. A realist review of advance care planning for people with multiple sclerosis and their families. PLoS One. 2020;15(10):e0240815.
40. Creutzfeldt CJ, Robinson MT, Holloway RG. Neurologists as primary palliative care providers: Communication and practice approaches. Neurol Clin Pract. 2016;6(1):40-8.
41. Crowther JL. Dementia: What Care Do Patients and Informal Carers Need in the Last Year of Life and Time Surrounding Death? An Exploratory Qualitative Study. England: The University of Liverpool (United Kingdom); 2011.
42. Dias LM, Barra W, Rego F. Advance Care Planning in Oncology: Challenges in Clinical Practice. Portugal: Universidade do Porto (Portugal); 2022.
43. Dickerson LK, Lipson TA, Chauhan SSB, Allen GI, Young B, Park JO, et al. Evaluating surgeon communication of pancreatic cancer prognosis using the VitalTalk ADAPT framework. J Surg Oncol. 2024;130(3):476-84.
44. Diggle MFJ, Schutz S, Butcher D. Bereaved Caregivers' Experiences of End of Life Care For People With Advanced Heart Failure: A Narrative Synthesis. Omega (Westport). 2025;90(4):1581-608.
45. Disler R, Pascoe A, Luckett T, Donesky D, Irving L, Currow DC, et al. Barriers to Palliative Care Referral and Advance Care Planning (ACP) for Patients With COPD: A Cross-Sectional Survey of Palliative Care Nurses. Am J Hosp Palliat Care. 2022;39(2):169-77.
46. Downer K, Gustin J, Lincoln T, Goodman L, Barnett MD. Communicating About Time-Limited Trials. Chest. 2022;161(1):202-7.
47. Ekberg S, Parry R, Land V, Ekberg K, Pino M, Antaki C, et al. Communicating with patients and families about illness progression and end of life: a review of studies using direct observation of clinical practice. BMC Palliat Care. 2021;20(1):186.
48. Epstein RM. Facing epistemic and complex uncertainty in serious illness: The role of mindfulness and shared mind. Patient Educ Couns. 2021;104(11):2635-42.
49. Etkind SN, Fennessy R, Wang J, Simpson RM, O'Neill B, Barclay SIG, et al. Communicating prognostic uncertainties in advanced multimorbidity: a multimethod qualitative study to co-design practice recommendations. Eur Geriatr Med. 2025.

50. Etkind SN, Koffman J. Approaches to managing uncertainty in people with life-limiting conditions: role of communication and palliative care. Postgrad Med J. 2016;92(1089):412-7.
51. Evans LR, Boyd EA, Malvar G, Apatira L, Luce JM, Lo B, et al. Surrogate decision-makers' perspectives on discussing prognosis in the face of uncertainty. Am J Respir Crit Care Med. 2009;179(1):48-53.
52. Fallowfield LJ, Jenkins VA, Beveridge HA. Truth may hurt but deceit hurts more: communication in palliative care. Palliat Med. 2002;16(4):297-303.
53. Fenton A, Fletcher KM, Kizildag D, Borstelmann NA, Kessler D, Cronin C, et al. Cancer Caregivers' Prognostic and End-of-Life Communication Needs and Experiences and their Impact. J Pain Symptom Manage. 2023;65(1):16-25.
54. Fisher M, Ridley S. Uncertainty in end-of-life care and shared decision making. Critical care and resuscitation : journal of the Australasian Academy of Critical Care Medicine. 2012;14(1):81-7.
55. Fitzsimons D, Doherty LC, Murphy M, Dixon L, Donnelly P, McDonald K, et al. Inadequate Communication Exacerbates the Support Needs of Current and Bereaved Caregivers in Advanced Heart Failure and Impedes Shared Decision-making. J Cardiovasc Nurs. 2019;34(1):11-9.
56. Formagini T, Poague C, O'Neal A, Brooks JV. "When I Heard the Word Palliative": Obscuring and Clarifying Factors Affecting the Stigma Around Palliative Care Referral in Oncology. JCO Oncol Pract. 2022;18(1):e72-e9.
57. Gadoud AC. A palliative care approach for people with advanced heart failure: recognition of need, transitions in care and impact on patients, family carers and clinicians. England: University of Hull (United Kingdom); 2013.
58. Garland EL, Bruce A, Stajduhar K. Exposing barriers to end-of-life communication in heart failure: an integrative review. Canadian journal of cardiovascular nursing = Journal canadien en soins infirmiers cardio-vasculaires. 2013;23(1):12-8.
59. Geerse OP, Lamas DJ, Sanders JJ, Paladino J, Kavanagh J, Henrich NJ, et al. A Qualitative Study of Serious Illness Conversations in Patients with Advanced Cancer. J Palliat Med. 2019;22(7):773-81.
60. Gerlach C, Goebel S, Weber S, Weber M, Sleeman KE. Space for intuition - the 'Surprise'-Question in haemato-oncology: Qualitative analysis of experiences and perceptions of haemato-oncologists. Palliat Med. 2019;33(5):531-40.
61. Gofton TE, Chum M, Schulz V, Gofton BT, Sarpal A, Watling C. Challenges facing palliative neurology practice: A qualitative analysis. J Neurol Sci. 2018;385:225-31.
62. Gonella S, Basso I, Dimonte V, Giulio PD. The role of end-of-life communication in contributing to palliative-oriented care at the end-of-life in nursing home. Int J Palliat Nurs. 2022;28(1):16-26.
63. Goodlin SJ, Quill TE, Arnold RM. Communication and decision-making about prognosis in heart failure care. J Card Fail. 2008;14(2):106-13.
64. Goss AL, Voumard RR, Engelberg RA, Curtis JR, Creutzfeldt CJ. Do They Have a Choice? Surrogate Decision-Making After Severe Acute Brain Injury. Crit Care Med. 2023;51(7):924-35.
65. Gough N, Ross JR, Riley J, Judson I, Koffman J. 'When something is this rare ... how do you know bad really is bad...?'-views on prognostic discussions from patients with advanced soft tissue sarcoma. BMJ Support Palliat Care. 2019;9(1):100-7.

66. Gramling R, Norton S, Ladwig S, Winters P, Metzger M, Quill T, et al. Latent classes of prognosis conversations in palliative care: a mixed-methods study. J Palliat Med. 2013;16(6):653-60.
67. Gramling R, Norton SA, Ladwig S, Metzger M, DeLuca J, Gramling D, et al. Direct observation of prognosis communication in palliative care: a descriptive study. J Pain Symptom Manage. 2013;45(2):202-12.
68. Gray TF, Temel JS, El-Jawahri A. Illness and prognostic understanding in patients with hematologic malignancies. Blood Rev. 2021;45:100692.
69. Green E, Gardiner C, Gott M, Ingleton C. Exploring the extent of communication surrounding transitions to palliative care in heart failure: the perspectives of health care professionals. Journal of palliative care. 2011;27(2):107-16.
70. Gutierrez KM. Communication of prognostic information in an ICU at end of life: practices among and between nurses, physicians and family members: University of Minnesota; 2010.
71. Gutierrez KM. Prognostic communication of critical care nurses and physicians at end of life. Dimens Crit Care Nurs. 2012;31(3):170-82.
72. Gutierrez KM. Prognostic categories and timing of negative prognostic communication from critical care physicians to family members at end-of-life in an intensive care unit. Nurs Inq. 2013;20(3):232-44.
73. Habib AR, Chen R, Magnavita ES, Jaung T, Awad MM, Odejide O, et al. Prevalence and Tolerance of Prognostic Uncertainty Among Thoracic Oncologists. Oncologist. 2021;26(8):e1480-e2.
74. Habib AR, Cronin AM, Earle CC, Tulsky JA, Mack JW, Abel GA, et al. How Do Blood Cancer Doctors Discuss Prognosis? Findings from a National Survey of Hematologic Oncologists. J Palliat Med. 2019;22(6):677-84.
75. Hagerty RG, Butow PN, Ellis PM, Dimitry S, Tattersall MH. Communicating prognosis in cancer care: a systematic review of the literature. Ann Oncol. 2005;16(7):1005-53.
76. Halkett GK, Lobb EA, Oldham L, Nowak AK. The information and support needs of patients diagnosed with High Grade Glioma. Patient Educ Couns. 2010;79(1):112-9.
77. Hallen SA, Hootsmans NA, Blaisdell L, Gutheil CM, Han PK. Physicians' perceptions of the value of prognostic models: the benefits and risks of prognostic confidence. Health Expect. 2015;18(6):2266-77.
78. Han PK. The Need for Uncertainty: A Case for Prognostic Silence. Perspect Biol Med. 2016;59(4):567-75.
79. Han PK, Dieckmann NF, Holt C, Gutheil C, Peters E. Factors Affecting Physicians' Intentions to Communicate Personalized Prognostic Information to Cancer Patients at the End of Life: An Experimental Vignette Study. Med Decis Making. 2016;36(6):703-13.
80. Hancock K, Clayton JM, Parker SM, Wal der S, Butow PN, Carrick S, et al. Truth-telling in discussing prognosis in advanced life-limiting illnesses: a systematic review. Palliat Med. 2007;21(6):507-17.
81. Harrison M, Gardiner C, Taylor B, Ejegi-Memeh S, Darlison L. Understanding the palliative care needs and experiences of people with mesothelioma and their family carers: An integrative systematic review. Palliat Med. 2021;35(6):1039-51.
82. Harrison M, Han PKJ, Rabin B, Bell M, Kay H, Spooner L, et al. Communicating uncertainty in cancer prognosis: a review of web-based prognostic tools. Patient education and counseling. 2019;102(5):842-9.

83. Hendershot KA, Elias MN, Taylor BL, Wahlster S, Creutzfeldt CJ. An Update on Palliative Care in Neurocritical Care: Providing Goal-Concordant Care in the Face of Prognostic Uncertainty. Curr Treat Options Neurol. 2023;25(12):517-29.
84. Henselmans I, Smets EMA, Han PKJ, de Haes H, Laarhoven H. How long do I have? Observational study on communication about life expectancy with advanced cancer patients. Patient Educ Couns. 2017;100(10):1820-7.
85. Heyland DK, Allan DE, Rocker G, Dodek P, Pichora D, Gafni A. Discussing prognosis with patients and their families near the end of life: impact on satisfaction with end-of-life care. Open Med. 2009;3(2):e101-10.
86. Higginson IJ, Koffman J, Hopkins P, Prentice W, Burman R, Leonard S, et al. Development and evaluation of the feasibility and effects on staff, patients, and families of a new tool, the Psychosocial Assessment and Communication Evaluation (PACE), to improve communication and palliative care in intensive care and during clinical uncertainty. BMC medicine. 2013;11:1-14.
87. Hjelmfors L, Stromberg A, Friedrichsen M, Martensson J, Jaarsma T. Communicating prognosis and end-of-life care to heart failure patients: a survey of heart failure nurses' perspectives. Eur J Cardiovasc Nurs. 2014;13(2):152-61.
88. Hjelmfors L, Strömberg A, Friedrichsen M, Mårtensson J, Jaarsma T. Communication About the Heart Failure Trajectory in Patients, Their Families and Health Care Professionals. Sweden: Linkopings Universitet (Sweden); 2018.
89. Hoff L, Hermeren G. Between uncertainty and certainty. The Journal of clinical ethics. 2011;22(2):139-50.
90. Hoff L, Hermeren G. Identifying challenges to communicating with patients about their imminent death. The Journal of clinical ethics. 2014;25(4):296-306.
91. Holloway RG, Gramling R, Kelly AG. Estimating and communicating prognosis in advanced neurologic disease. Neurology. 2013;80(8):764-72.
92. Hudson BF, Shulman C, Low J, Hewett N, Daley J, Davis S, et al. Challenges to discussing palliative care with people experiencing homelessness: a qualitative study. BMJ Open. 2017;7(11):e017502.
93. Hui D, Mo L, Paiva CE. The Importance of Prognostication: Impact of Prognostic Predictions, Disclosures, Awareness, and Acceptance on Patient Outcomes. Curr Treat Options Oncol. 2021;22(2):12.
94. Hui D, Paiva CE, Del Fabbro EG, Steer C, Naberhuis J, van de Wetering M, et al. Prognostication in advanced cancer: update and directions for future research. Support Care Cancer. 2019;27(6):1973-84.
95. Innes S, Payne S. Advanced cancer patients' prognostic information preferences: a review. Palliat Med. 2009;23(1):29-39.
96. International Nurses' End-of-Life Decision-Making in Intensive Care Research G, Gallagher A, Bousso RS, McCarthy J, Kohlen H, Andrews T, et al. Negotiated reorienting: a grounded theory of nurses' end-of-life decision-making in the intensive care unit. Int J Nurs Stud. 2015;52(4):794-803.
97. Isaac M, Curtis JR. How to respond to an ICU patient asking if she/he is going to die. Intensive Care Med. 2017;43(2):220-2.
98. Islam Z, Pollock K, Patterson A, Hanjari M, Wallace L, Mururajani I, et al. Thinking ahead about medical treatments in advanced illness: a qualitative study of barriers and enablers in end-of-life care planning with patients and families from ethnically diverse backgrounds. Health Soc Care Deliv Res. 2023;11(7):1-135.
99. Jackson VA, Emanuel L. Navigating and Communicating about Serious Illness and End of Life. N Engl J Med. 2024;390(1):63-9.

100. Johnson M, Tod AM, Brummell S, Collins K. Prognostic communication in cancer: A critical interpretive synthesis of the literature. Eur J Oncol Nurs. 2015;19(5):554-67.
101. Johnson RF, Jr., Gustin J. Acute lung injury and acute respiratory distress syndrome requiring tracheal intubation and mechanical ventilation in the intensive care unit: impact on managing uncertainty for patient-centered communication. Am J Hosp Palliat Care. 2013;30(6):569-75.
102. Jones MB. A case analysis of the legal, professional, and ethical responsibilities of registered nurses for disclosure of prognostic information to dying adults (philosophy). United States -- Alabama: The University of Alabama at Birmingham; 1983.
103. Kantabanlang Y, Lin CP, Nilmanat K, Guo P. Palliative care needs of patients with hematologic malignancies and family caregivers and challenges of palliative care provision in Asia: A review of evidence. Semin Hematol. 2023;60(4):220-5.
104. Kapo JM, Casarett D. Review: Prognosis in chronic diseases. Annals of Long-Term Care. 2006;14(2):18-23.
105. Kimbell B. Living, dying and caring in advanced liver disease: the challenge of uncertainty. Scotland: The University of Edinburgh (United Kingdom); 2014.
106. Kimbell B, Boyd K, Kendall M, Iredale J, Murray SA. Managing uncertainty in advanced liver disease: a qualitative, multiperspective, serial interview study. BMJ Open. 2015;5(11):e009241.
107. Ko E, Fuentes D, Singh-Carlson S, Nedjat-Haiem F. Challenges and facilitators of hospice decision-making: a retrospective review of family caregivers of home hospice patients in a rural US-Mexico border region-a qualitative study. BMJ Open. 2020;10(7):e035634.
108. Krawczyk M, Gallagher R. Communicating prognostic uncertainty in potential end-of-life contexts: experiences of family members. BMC Palliat Care. 2016;15:59.
109. Kupeli N, Sampson EL, Leavey G, Harrington J, Davis S, Candy B, et al. Context, mechanisms and outcomes in end-of-life care for people with advanced dementia: family carers perspective. BMC Palliat Care. 2019;18(1):87.
110. Langmuir T, Chu A, Sehabi G, Giguere L, Lamarche J, Boudjatat W, et al. A new landscape in illness uncertainty: A systematic review and thematic synthesis of the experience of uncertainty in patients with advanced cancer receiving immunotherapy or targeted therapy. Psychooncology. 2023;32(3):356-67.
111. LeBlanc TW, Temel JS, Helft PR. "How Much Time Do I Have?": Communicating Prognosis in the Era of Exceptional Responders. Am Soc Clin Oncol Educ Book. 2018;38:787-94.
112. LeClaire MM, Oakes JM, Weinert CR. Communication of prognostic information for critically ill patients. Chest. 2005;128(3):1728-35.
113. Lee CW, Vitous CA, Silveira MJ, Forman J, Dossett LA, Mody L, et al. Delays in Palliative Care Referral Among Surgical Patients: Perspectives of Surgical Residents Across the State of Michigan. J Pain Symptom Manage. 2019;57(6):1080-8 e1.
114. Low J, Davis S, Vickerstaff V, Greenslade L, Hopkins K, Langford A, et al. Advanced chronic liver disease in the last year of life: a mixed methods study to understand how care in a specialist liver unit could be improved. BMJ Open. 2017;7(8):e016887.
115. Maguire P, Faulkner A. Communicate with cancer patients: 2. Handling uncertainty, collusion, and denial. BMJ. 1988;297(6654):972-4.

116. Mandel EI, Bernacki RE, Block SD. Serious Illness Conversations in ESRD. Clin J Am Soc Nephrol. 2017;12(5):854-63.
117. Marie C. Difficult conversations with dying people and their families: Marie Curie; 2014.
118. Marie C. What’s stopping doctors having that conversation about death? Marie Curie. 2019.
119. Marie C. Tips for talking to your patients about a prognosis. Marie Curie. 2020.
120. Masterson MP, Applebaum AJ, Buda K, Reisch S, Rosenfeld B. Don't Shoot the Messenger: Experiences of Delivering Prognostic Information in the Context of Advanced Cancer. Am J Hosp Palliat Care. 2018;35(12):1526-31.
121. Maxwell CA, Mixon AS, Conner E, Phillippi JC. Receptivity of Hospitalized Older Adults and Family Caregivers to Prognostic Information about Aging, Injury, and Frailty: A Qualitative Study. Int J Nurs Stud. 2020;109:103602.
122. McClung JA. End-of-life care in the treatment of advanced heart failure in the elderly. Cardiol Rev. 2013;21(1):9-15.
123. McDonald A, Sherlock J. A long and winding road: Improving communication with patients in the NHS: Marie Curie; 2016.
124. McLennon SM, Uhrich M, Lasiter S, Chamness AR, Helft PR. Oncology nurses' narratives about ethical dilemmas and prognosis-related communication in advanced cancer patients. Cancer Nurs. 2013;36(2):114-21.
125. Medendorp NM, Stiggelbout AM, Aalfs CM, Han PKJ, Smets EMA, Hillen MA. A scoping review of practice recommendations for clinicians' communication of uncertainty. Health Expect. 2021;24(4):1025-43.
126. Michael N, Shah R. Communication about Advanced Progressive Disease , Prognosis, and Advance Care Plans. In: Kissane DWMW, William B, editors. Psycho-Oncology in Palliative and End of Life Care. New York: Oxford Academic; 2022. p. 1-C.P207.
127. Moglia C, Palumbo F, Botto R, Iazzolino B, Ticozzi N, Calvo A, et al. Prognostic communication in amyotrophic lateral sclerosis: findings from a Nationwide Italian survey. Neurol Sci. 2024;45(12):5787-94.
128. Molzahn AE, Sheilds L, Bruce A, Schick-Makaroff K, Antonio M, Clark AM. Life and priorities before death: A narrative inquiry of uncertainty and end of life in people with heart failure and their family members. Eur J Cardiovasc Nurs. 2020;19(7):629-37.
129. Momen NC, Barclay SI. Addressing 'the elephant on the table': barriers to end of life care conversations in heart failure - a literature review and narrative synthesis. Curr Opin Support Palliat Care. 2011;5(4):312-6.
130. Mone S, Kerr H. Prognostic awareness in advanced cancer: an integrative literature review. BMJ Support Palliat Care. 2021;11(1):53-8.
131. Mount BM. Psychological Impact of Urologic Cancer. Cancer. 1980;45 Suppl 7:1985-92.
132. Nadicksbernd J. Why Do Community Healthcare Professionals Find Some Conversations Difficult? England: University of London, Royal Holloway College (United Kingdom); 2023.
133. Nagelschmidt K, Leppin N, Seifart C, Rief W, von Blanckenburg P. Systematic mixed-method review of barriers to end-of-life communication in the family context. BMJ Support Palliat Care. 2021;11(3):253-63.

134. Narayan M, Jones J, Portalupi LB, McIlvennan CK, Matlock DD, Allen LA. Patient Perspectives on Communication of Individualized Survival Estimates in Heart Failure. J Card Fail. 2017;23(4):272-7.
135. Ngwenya N, Crang C, Farquhar M, Rintoul RC, Mahadeva R, Calvert LD, et al. Communicating uncertainty: contrasting the communication experiences of patients with advanced COPD and incurable lung cancer. Fam Pract. 2021;38(5):637-43.
136. Nice. Care of dying adults in the last days of life. NICE. 2015.
137. Nice. Chronic heart failure in adults: diagnosis and management. NICE. 2018.
138. Noble H, Brazil K, Burns A, Hallahan S, Normand C, Roderick P, et al. Clinician views of patient decisional conflict when deciding between dialysis and conservative management: Qualitative findings from the PAlliative Care in chronic Kidney diSease (PACKS) study. Palliat Med. 2017;31(10):921-31.
139. Olsson MM, Windsor C, Chambers S, Green TL. A Scoping Review of End-of-Life Communication in International Palliative Care Guidelines for Acute Care Settings. J Pain Symptom Manage. 2021;62(2):425-37 e2.
140. Owusuaa C, van Beelen I, van der Heide A, van der Rijt CCD. Physicians' views on the usefulness and feasibility of identifying and disclosing patients' last phase of life: a focus group study. BMJ Support Palliat Care. 2021;14(e2).
141. Pardue CM. Uncertainty in the context of end-of-life communication in heart failure. Dissertation Abstracts International: Section B: The Sciences and Engineering. 2021;82(8):No-Specified.
142. Parker S, Clayton J, Hancock K, Walder S, Butow P, Carrick S, et al. Communicating Prognosis and Issues Surrounding End of Life (EOL) in adults in the advanced stages of a life-limiting illness: A Systematic Review. 2006.
143. Parker SM, Clayton JM, Hancock K, Walder S, Butow PN, Carrick S, et al. A systematic review of prognostic/end-of-life communication with adults in the advanced stages of a life-limiting illness: patient/caregiver preferences for the content, style, and timing of information. J Pain Symptom Manage. 2007;34(1):81-93.
144. Parry R. Communication in Palliative Care and About End of Life: A State-of-the-Art Literature Review of Conversation-Analytic Research in Healthcare. Research on Language and Social Interaction. 2024;57(1):127-48.
145. Parvez S, Abdel-Kader K, Song MK, Unruh M. Conveying uncertainty in prognosis to patients with ESRD. Blood Purif. 2015;39(1-3):58-64.
146. Payne S, Burton C, Addington-Hall J, Jones A. End-of-life issues in acute stroke care: a qualitative study of the experiences and preferences of patients and families. Palliat Med. 2010;24(2):146-53.
147. Peoples HA, Boone B, Blumenthal-Barby JS, Bruce CR. How Clinician-Family Interactions Potentially Impact Clinicians' Conceptualization and Discussions Regarding Prognostic Uncertainties. J Palliat Care. 2020;35(1):29-33.
148. Perumalswami CR, Jagsi R, Goold SD. Predicting a "Lazarus Effect" in Patients With Advanced Cancer Near the End of Life: Prognostic Uncertainty, Oncologists' Emotions, and Ethical Questions. Am J Bioeth. 2019;19(12):57-60.
149. Pfeil TA, Laryionava K, Reiter-Theil S, Hiddemann W, Winkler EC. What keeps oncologists from addressing palliative care early on with incurable cancer patients? An active stance seems key. Oncologist. 2015;20(1):56-61.
150. Piovesan M, Orr P, Tevyaw S, Roussos E, Cherid C, Bouchard S. A Prospective Study with Patients and Families on the Usefulness of Accurate

Prognosis for Palliative Care Patients. J Soc Work End Life Palliat Care. 2024;20(2):133-46.
151. Pontin D, Jordan N. Issues in prognostication for hospital specialist palliative care doctors and nurses: a qualitative inquiry. Palliat Med. 2013;27(2):165-71.
152. Pooler C, Richman-Eisenstat J, Kalluri M. Early integrated palliative approach for idiopathic pulmonary fibrosis: A narrative study of bereaved caregivers' experiences. Palliat Med. 2018;32(9):1455-64.
153. Powers BA, Norton SA, Schmitt MH, Quill TE, Metzger M. Meaning and practice of palliative care for hospitalized older adults with life limiting illnesses. J Aging Res. 2011;2011:406164.
154. Pun J, Chow JCH, Fok L, Cheung KM. Role of patients' family members in end-of-life communication: an integrative review. BMJ Open. 2023;13(2):e067304.
155. Rafferty KA, Cramer EM, Priddis D. Managing end-of-life uncertainty: applying problematic integration theory to spousal communication about death and dying. American Journal of Hospice and Palliative Medicine®. 2016;33(1):69-76.
156. Reid C, Gibbins J, Bloor S, Burcombe M, McCoubrie R, Forbes K. Healthcare professionals' perspectives on delivering end-of-life care within acute hospital trusts: a qualitative study. BMJ Support Palliat Care. 2015;5(5):490-5.
157. Rhee JJ, Zwar NA, Kemp LA. Why are advance care planning decisions not implemented? Insights from interviews with Australian general practitioners. J Palliat Med. 2013;16(10):1197-204.
158. Richards N, Ingleton C, Gardiner C, Gott M. Awareness contexts revisited: indeterminacy in initiating discussions at the end-of-life. J Adv Nurs. 2013;69(12):2654-64.
159. Ridley S, Fisher M. Uncertainty in end-of-life care. Curr Opin Crit Care. 2013;19(6):642-7.
160. Risco JR, Kelly AG, Holloway RG. Prognostication in neurology. Handb Clin Neurol. 2022;190:175-93.
161. Robinson J, Pilbeam C, Goodwin H, Raphael D, Waterworth S, Gott M. The impact of uncertainty on bereaved family's experiences of care at the end of life: a thematic analysis of free text survey data. BMC Palliat Care. 2021;20(1):60.
162. Rowe JG, Potts M, McGhie R, Dinh A, Engel I, England K, et al. Palliative Care Practice During the COVID-19 Pandemic: A Descriptive Qualitative Study of Palliative Care Clinicians. J Pain Symptom Manage. 2021;62(6):1111-6.
163. Salins N, Dhyani VS, Mathew M, Prasad A, Rao AP, Damani A, et al. Assessing palliative care practices in intensive care units and interpreting them using the lens of appropriate care concepts. An umbrella review. Intensive Care Med. 2024;50(9):1438-58.
164. Sanders JJ, Blanch-Hartigan D, Ericson J, Tarbi E, Rizzo D, Gramling R, et al. Methodological innovations to strengthen evidence-based serious illness communication. Patient Educ Couns. 2023;114:107790.
165. Scholz B, Goncharov L, Emmerich N, Lu VN, Chapman M, Clark SJ, et al. Clinicians' accounts of communication with patients in end-of-life care contexts: A systematic review. Patient Educ Couns. 2020;103(10):1913-21.
166. Schuster RA, Hong SY, Arnold RM, White DB. Do physicians disclose uncertainty when discussing prognosis in grave critical illness? Narrat Inq Bioeth. 2012;2(2):125-35.
167. Schutz REC, Creutzfeldt CJ. Three big things in neuropalliative care: Communication, personhood and uncertainty. J Neurol Sci. 2018;385:232-3.

168. Sedhom R, Bates-Pappas GE, Feldman J, Elk R, Gupta A, Fisch MJ, et al. Tumor Is Not the Only Target: Ensuring Equitable Person-Centered Supportive Care in the Era of Precision Medicine. Am Soc Clin Oncol Educ Book. 2024;44(3):e434026.
169. Selman LE, Bristowe K, Higginson IJ, Murtagh FEM. The views and experiences of older people with conservatively managed renal failure: a qualitative study of communication, information and decision-making. BMC Nephrol. 2019;20(1):38.
170. Sikhosana N, Whitehead D, Moxham L, Karacsony S, Namasivayam P, Fernandez R. Voice of persons with a life-limiting illness in conversation with healthcare professionals: systematic review and meta-synthesis. BMJ Support Palliat Care. 2023.
171. Simpkin AL, Armstrong KA. Communicating Uncertainty: a Narrative Review and Framework for Future Research. J Gen Intern Med. 2019;34(11):2586-91.
172. Smith AK, Glare P. Ethical issues in prognosis and prognostication. The Oxford handbook of ethics at the end of life. 2014:170-89.
173. Stanek MS, Norton SA. Expressions of Uncertainty from Patients with Advanced Cancer when Communicating with the Palliative Care Team. United States -- New York: University of Rochester; 2020.
174. Stiel H, Nagarajan SV, Forster BC, Clayton JM. Palliative care physicians' perspectives on transferring patients to nursing homes and communication strategies to facilitate this transition: A qualitative study. Palliat Med. 2019;33(3):323-31.
175. Stocker R. Experiences and perceptions of the role of palliative and end of life care in heart failure: a modified grounded theory study. England: University of Durham (United Kingdom); 2014.
176. Stocker R, Close H, Hancock H, Hungin APS. Should heart failure be regarded as a terminal illness requiring palliative care? A study of heart failure patients', carers' and clinicians' understanding of heart failure prognosis and its management. BMJ Support Palliat Care. 2017;7(4):464-9.
177. Sullivan AM, Lakoma MD, Matsuyama RK, Rosenblatt L, Arnold RM, Block SD. Diagnosing and discussing imminent death in the hospital: a secondary analysis of physician interviews. J Palliat Med. 2007;10(4):882-93.
178. Szawarski P. Classic cases revisited: Oscar the cat and predicting death. J Intensive Care Soc. 2016;17(4):341-5.
179. Tarbi EC, Gramling R, Bradway C, Broden EG, Meghani SH. "I Had a Lot More Planned": The Existential Dimensions of Prognosis Communication with Adults with Advanced Cancer. J Palliat Med. 2021;24(10):1443-54.
180. Tavares N, Hunt KJ, Jarrett N, Wilkinson TM. The preferences of patients with chronic obstructive pulmonary disease are to discuss palliative care plans with familiar respiratory clinicians, but to delay conversations until their condition deteriorates: A study guided by interpretative phenomenological analysis. Palliat Med. 2020;34(10):1361-73.
181. Tavares NC. Palliative Care Communication in Copd : Patients' Preferences and Clinicians' Judgements. England: University of Southampton (United Kingdom); 2019.
182. Temel JS, Shaw AT, Greer JA. Challenge of Prognostic Uncertainty in the Modern Era of Cancer Therapeutics. J Clin Oncol. 2016;34(30):3605-8.

183. Tetteh DA, Akhther N. Openness and topic avoidance in interpersonal communication about ovarian cancer: An uncertainty management perspective. Qual Res Med Healthc. 2021;5(3):9376.
184. Tilburgs B, Vernooij-Dassen M, Koopmans R, van Gennip H, Engels Y, Perry M. Barriers and facilitators for GPs in dementia advance care planning: A systematic integrative review. PLoS One. 2018;13(6):e0198535.
185. Tolsa L, Jones L, Michel P, Borasio GD, Jox RJ, Rutz Voumard R. 'We Have Guidelines, but We Can Also Be Artists': Neurologists Discuss Prognostic Uncertainty, Cognitive Biases, and Scoring Tools. Brain Sci. 2022;12(11):1591.
186. Trankle SA, Shanmugam S, Lewis E, Nicholson M, Hillman K, Cardona M. Are We Making Progress on Communication with People Who Are Near the End of Life in the Australian Health System? A Thematic Analysis. Health Commun. 2020;35(2):158-67.
187. Tuck KK, Brod L, Nutt J, Fromme EK. Preferences of patients with Parkinson's disease for communication about advanced care planning. Am J Hosp Palliat Care. 2015;32(1):68-77.
188. Udo C, Lovgren M, Lundquist G, Axelsson B. Palliative care physicians' experiences of end-of-life communication: A focus group study. Eur J Cancer Care (Engl). 2018;27(1).
189. van der Velden NCA, Han PKJ, van Laarhoven HWM, de Vos F, Hendriks LEL, Burgers SA, et al. Prevalence and Predictors of Physician-Patient Discordance in Prognostic Perceptions in Advanced Cancer. Oncologist. 2023;28(8):e653-e68.
190. van der Velden NCA, Meijers MC, Han PKJ, van Laarhoven HWM, Smets EMA, Henselmans I. The Effect of Prognostic Communication on Patient Outcomes in Palliative Cancer Care: a Systematic Review. Curr Treat Options Oncol. 2020;21(5):40.
191. van der Velden NCA, Smets EMA, van Vliet LM, Brom L, van Laarhoven HWM, Henselmans I. Effects of Prognostic Communication Strategies on Prognostic Perceptions, Treatment Decisions and End-Of-Life Anticipation in Advanced Cancer: An Experimental Study among Analogue Patients. J Pain Symptom Manage. 2024;67(6):478-89 e13.
192. van der Velden NCA, Smets EMA, van Vliet LM, Brom L, van Laarhoven HWM, Henselmans I. Effects of prognostic communication strategies on emotions, coping, and appreciation of consultations: An experimental study in advanced cancer. Palliat Support Care. 2024:1-13.
193. van der Velden NCA, van der Kleij MBA, Lehmann V, Smets EMA, Stouthard JML, Henselmans I, et al. Communication about Prognosis during Patient-Initiated Second Opinion Consultations in Advanced Cancer Care: An Observational Qualitative Analysis. Int J Environ Res Public Health. 2021;18(11).
194. van Eenennaam RM, Kruithof WJ, van Es MA, Kruitwagen-van Reenen ET, Westeneng HJ, Visser-Meily JMA, et al. Discussing personalized prognosis in amyotrophic lateral sclerosis: development of a communication guide. BMC Neurol. 2020;20(1):446.
195. Vlckova K, Tuckova A, Polakova K, Loucka M. Factors associated with prognostic awareness in patients with cancer: A systematic review. Psychooncology. 2020;29(6):990-1003.
196. Walczak A, Butow PN, Bu S, Clayton JM. A systematic review of evidence for end-of-life communication interventions: Who do they target, how are they structured and do they work? Patient Educ Couns. 2016;99(1):3-16.

197. Walczak A, Butow PN, Davidson PM, Bellemore FA, Tattersall MH, Clayton JM, et al. Patient perspectives regarding communication about prognosis and end-of-life issues: how can it be optimised? Patient Educ Couns. 2013;90(3):307-14.
198. Waldrop DP, Meeker MA, Kerr C, Skretny J, Tangeman J, Milch R. The nature and timing of family-provider communication in late-stage cancer: a qualitative study of caregivers' experiences. J Pain Symptom Manage. 2012;43(2):182-94.
199. Wijsenbeek MS, Bonella F, Orsatti L, Russell AM, Valenzuela C, Wuyts WA, et al. Communicating with patients with idiopathic pulmonary fibrosis: can we do it better? ERJ Open Res. 2022;8(1):00422-2021.
200. Wittenberg SM, Cohen LM. Estimating prognosis in end-stage renal disease. Progress in Palliative Care. 2013;17(4):165-9.
201. Wittenberg-Lyles EM, Goldsmith J, Sanchez-Reilly S, Ragan SL. Communicating a terminal prognosis in a palliative care setting: deficiencies in current communication training protocols. Soc Sci Med. 2008;66(11):2356-65.
202. Wu Y, Zhang X. Examining Conversation Analysis in Palliative Care: A Systematic Review. Health Commun. 2024;39(13):3072-83.
203. Xie W, Butcher R. Artificial Intelligence Decision Support Tools for End-of-Life Care Planning Conversations: CADTH Horizon Scan. 2023.
204. Young J, Lyons A, Egan R, Dew K. Embodied decisions unfolding over time: a meta-ethnography systematic review of people with cancer's reasons for delaying or declining end-of-life care. BMC Palliat Care. 2024;23(1):45.
205. Ziegler DS. Never say never. J Clin Oncol. 2015;33(5):518-20.
