# Supplementary material for: Improving communication of prognostic uncertainty in advanced illness: a realist review
Source: BMJ Open. 2026 Jul 1;16(7):e115385. doi: 10.1136/bmjopen-2025-115385 (PMC13663710; doi:10.1136/bmjopen-2025-115385)
Supplement: Supplementary file 8 [file bmjopen-2025-115385.draft_revisions.pdf]

BMJ Open is committed to open peer review. As part of this commitment we make the peer review history of every article we publish publicly available.

When an article is published we post the peer reviewers' comments and the authors' responses online. We also post the versions of the paper that were used during peer review. These are the versions that the peer review comments apply to.

The versions of the paper that follow are the versions that were submitted during the peer review process. They are not the versions of record or the final published versions. They should not be cited or distributed as the published version of this manuscript.

BMJ Open is an open access journal and the full, final, typeset and author-corrected version of record of the manuscript is available on our site with no access controls, subscription charges or pay-per-view fees (http://bmjopen.bmj.com).

If you have any questions on BMJ Open's open peer review process please email info.bmjopen@bmj.com

BMJ Open

Improving Communication of Prognostic Uncertainty in Advanced Illness: A Realist Review

Journal:	*BMJ Open*
Manuscript ID	bmjopen-2025-115385
Article Type:	Original research
Date Submitted by the Author:	16-Dec-2025

Complete List of Authors:	Etkind, Simon; University of Cambridge, Department of Public Health and Primary Care; Cambridge University Hospitals NHS Foundation Trust<br>Weetman, Katharine; University of Birmingham College of Medical and Dental Sciences<br>Hyams, Katy; University of Cambridge, Department of Public Health and Primary Care<br>Acharya, Tanvi; University of Cambridge, Department of Public Health and Primary Care<br>Shokraneh, Farhad; University of Cambridge, Department of Public Health and Primary Care<br>Macartney, John; University of Warwick Medical School

Keywords:	INTERNAL MEDICINE, PALLIATIVE CARE, GERIATRIC MEDICINE, Prognosis, Systematic Review

SCHOLARONE™
MANUSCRIPTS

I, the Submitting Author has the right to grant and does grant on behalf of all authors of the Work (as defined in the below author licence), an exclusive licence and/or a non-exclusive licence for contributions from authors who are: i) UK Crown employees; ii) where BMJ has agreed a CC-BY licence shall apply, and/or iii) in accordance with the terms applicable for US Federal Government officers or employees acting as part of their official duties; on a worldwide, perpetual, irrevocable, royalty-free basis to BMJ Publishing Group Ltd ("BMJ") its licensees and where the relevant Journal is co-owned by BMJ to the co-owners of the Journal, to publish the Work in this journal and any other BMJ products and to exploit all rights, as set out in our licence.

The Submitting Author accepts and understands that any supply made under these terms is made by BMJ to the Submitting Author unless you are acting as an employee on behalf of your employer or a postgraduate student of an affiliated institution which is paying any applicable article publishing charge ("APC") for Open Access articles. Where the Submitting Author wishes to make the Work available on an Open Access basis (and intends to pay the relevant APC), the terms of reuse of such Open Access shall be governed by a Creative Commons licence – details of these licences and which Creative Commons licence will apply to this Work are set out in our licence referred to above.

Other than as permitted in any relevant BMJ Author's Self Archiving Policies, I confirm this Work has not been accepted for publication elsewhere, is not being considered for publication elsewhere and does not duplicate material already published. I confirm all authors consent to publication of this Work and authorise the granting of this licence.

For peer review only

Improving Communication of Prognostic Uncertainty in Advanced Illness: A Realist Review

Authors

Simon Etkind*, (0000-0003-2863-8893) Assistant Professor of Palliative Care. sde23@medschl.cam.ac.uk[1,2] East Forvie Building, Forvie Site, Robinson Way, Cambridge, CB20SR *corresponding author.

Katharine Weetman, (0000-0003-0377-3933) Assistant Professor in Clinical Communication. k.e.weetman@bham.ac.uk[3]

Katy Hyams, Academic Clinical Fellow. kh851@cam.ac.uk[1]

Tanvi Acharya, Academic Clinical Fellow. ta387@medschl.cam.ac.uk[1]

Farhad Shokraneh,(0000-0001-9687-856) Research Associate. farhad@systematicreview.info[1]

John I MacArtney, (0000-0002-0879-4277) Associate Professor. John.Macartney@warwick.ac.uk[4]

Affiliations:

1. Primary Care Unit, University of Cambridge
2. Cambridge University Hospitals NHS Foundation Trust
3. School of Medical Sciences, College of Medicine & Health, University of Birmingham, B15 2TT
4. Warwick Medical School, University of Warwick

For peer review only

Abstract

Introduction

Prognostic uncertainty is common in advanced illness, driven by multimorbidity, treatment complexities, and unpredictable disease trajectories. When inadequately acknowledged or discussed, it can lead to patient and family distress and inhibit decision making. There is limited understanding of how communication of prognostic uncertainty can best support patients, families, and healthcare professionals.

Aims

To build a programme theory of how, why, and for whom communication of prognostic uncertainty "works" (or not) in advanced illness and palliative care contexts.

Methods

We conducted a realist systematic review. We searched six databases and multiple grey literature sources and included papers examining communication of prognostic uncertainty in adults with advanced illness. We assessed rigour and relevance of included papers and coded them qualitatively to identify Context–Mechanism–Outcome Configurations (CMOCs) relevant to prognostic uncertainty communication. Supported by stakeholder consultation, we refined these into a programme theory.

Results

From 4634 search results we included 205 studies, with 46,579 participants across 11 countries. In our final programme theory we identified contexts of communication: Socio-cultural norms and values, patient and family preferences, communication models and tools, clinician uncertainty tolerance, training and experience. Mechanisms of communication were timing and internal processing of discussion; language and content; explicit vs implicit discussion. Relevant outcomes were emotions, hope, distress, decision making and care planning. Effective communication varied based on individual characteristics and preferences, but was often characterised by openness, empathy, and tailoring to cultural and personal needs.

Conclusion

This programme theory provides a new understanding of the contexts and mechanisms that support effective communication of prognostic uncertainty to reduce distress and aid decision making. Sociocultural factors are widely recognised as important, but have the most limited supporting evidence. The model presented here can support professionals in individualised and compassionate communication of prognostic uncertainty in the context of advanced illness. Using this evidence-informed approach may enhance trust, preparedness, and shared care planning.

For peer review only - http://bmjopen.bmj.com/site/about/guidelines.xhtml

Background:

People with advanced illnesses commonly experience uncertainty in physical, social, practical, temporal and existential domains[1-3]. Whilst some illness uncertainties can be resolved e.g. through diagnostic testing or information provision, many cannot[4-6]. How uncertainty is managed and communicated is therefore increasingly important for those with serious illness and those caring for them[7], and is a recognised research priority[8].

Prognostic uncertainty is a specific subset of illness uncertainty that is increasing due to population changes and increasing illness complexity[9,10]. It can affect healthcare delivery in multiple settings by inhibiting timely end of life discussions and identification of needs[11], and can be a barrier for accessing services and benefits (e.g. palliative care, special rules access to benefits) that are gated by prognosis[12,13]. Consequently, poor communication of prognostic uncertainty may worsen inequities in the context of rising palliative care need[14,15]. For this reason, the importance of communicating prognostic uncertainty is increasingly recognised[5,8,16,17]

Most patients prefer open discussions of prognostic uncertainty, but such communication is often avoided or implicit[18-20], which may partly be attributed to health professional intolerance of uncertainty, perceptions that expressing uncertainty is a sign of failure, or clinical inexperience[18,19]. If poorly communicated or unshared, prognostic uncertainty can be distressing for patients, carers, and health professionals alike, inhibiting decision making.[3]

Communication of prognosis itself is well researched[16,21]. However, there is no evidence synthesis to inform how prognostic *uncertainty* can be best communicated to improve patient outcomes. Many patient, health system, and wider factors are likely to affect optimal communication,[22,23] so an explanatory approach is needed to inform preferred approaches to prognostic uncertainty communication, whilst accounting for these contextual factors.

We aimed to identify, understand, and explain current practice of communicating prognostic uncertainty in advanced illness contexts, including how and why different outcomes are achieved through verbal and written communications between healthcare professionals, patients, and their relatives and/or carers.

For peer review only - http://bmjopen.bmj.com/site/about/guidelines.xhtml

Methods

Review question

How, why, and for whom does the communication of prognostic uncertainty "work" (or not) in serious illness contexts potentially relevant to palliative and end-of-life care?

Design

Stakeholder informed realist systematic review and synthesis, reported according to the RAMESES standards (appendix 1)[24]. We chose this theory-driven approach to generate explanations of how communicating prognostic uncertainty may be theorised to "work" (or not), and if so, under what contexts, for whom, why, and to what extent [25,26]. Realist methodology is particularly suited to investigate complex interventions and sociological issues, such as that under scrutiny here[10,25,27]. The review protocol is registered on Prospero (CRD42024563546)

Inclusion and exclusion criteria:

We included evidence relating to verbal or written communication of prognostic uncertainty in advanced and life-limiting illness contexts relevant to palliative care. We included communication between healthcare professionals AND patients and/or relatives and/or carers and/or other healthcare professionals, in any healthcare setting. We included all study designs, as long as they contained content that could be scrutinised against the initial programme theory and contained at least one statement regarding how or for whom communication of prognostic uncertainty works and why[25,26].

Exclusion criteria: Prognostic uncertainty for patients without advanced or life-limiting illness; evidence specifically related to patients <18 years old, communication of prognosis without specific consideration of uncertainty.

Initial programme theory

On 29th April 2024, we conducted scoping searches in PubMed and Google Scholar using a title search for terms prognosis, palliative, end-of-life, communication, and uncertainty to identify highly relevant papers.[28] These papers were reviewed by four authors (SE, KW, FS, JM) who independently developed ideas for an initial programme theory. The initial theory was developed through discussion of these ideas with patient and clinician expert contributors, and formed the starting point for our searches and analysis (appendix 2).

For peer review only - http://bmjopen.bmj.com/site/about/guidelines.xhtml

Searches

Following realist review guidelines,[28-30] we searched the following sources with no limitations to time, language, document type, or publication status on 18th August 2024[31]: Embase, MEDLINE, CINAHL, Cochrane Database of Systematic Reviews, Science Citation Index-Expanded via Web of Science, PsycINFO and the Social Science Citation Index. To identify grey literature, we searched the following websites and repositories: NHS Digital, NHS Improvement, Royal College of Physicians, General Medical Council, NICE Guidelines, Marie Curie, Google Scholar, ProQuest Dissertations & Theses Global, Trip Database and the Department of Health and Social Care.

The search strategy included terms relating to Prognosis; Uncertainty; and Palliative care/Advanced illness; search terminology was informed by papers from the scoping search (appendix 3).

Screening and data extraction.

A single reviewer screened search results for eligibility, with 10% second screening at title/abstract stage. We assessed inter-rater agreement on these 10% and considered a score of k>0.8 to reflect sufficient agreement. 30% of results were double screened at the full text stage. Disagreements were resolved by discussion within the research team and informed the approach to subsequent screening decisions.

We used a bespoke data extraction form to extract relevant information from each article, populated by one researcher, with 10% double data extraction (appendix 4).

To extract data relevant to "context, mechanism, outcome" configurations (CMOCs), we imported the full text of included articles into NVivo (version 14 with collaboration cloud, QSR international). TA, FS, KW, and SE coded included articles to identify text relevant to the programme theory. We coded generally for areas of ‘context’, ‘mechanism’ and ‘outcome, and specifically for areas such as “content of communication” that could contribute to CMOCs. The coding framework was regularly reviewed and updated by the research team during data extraction.

Synthesis

Based on the coding of "Contexts, mechanisms, and outcomes, we developed a series of CMOCs related to the review question. We generated thematic areas that CMOCs addressed through reflexive consideration of the coding of included articles. We then integrated the CMOCs and thematic areas into a programme theory through discussion within the research team, and with key stakeholders, including patients, carers, and clinicians.

For peer review only - http://bmjopen.bmj.com/site/about/guidelines.xhtml

Rigour and relevance assessments

We made a judgement of low, moderate or high rigour, based on an evaluation of the research methods in relation to the question being asked by each manuscript[32]. Papers collecting primary data were rated as 'high' rigour where methods were appropriate for the question, and there were no flaws identified that affected trustworthiness of the findings. If minor biases or issues were identified, we rated as moderate. If serious concerns limited interpretation, we rated as low rigour. Literature reviews were rated as low rigour if there was no description of a search strategy. Systematic reviews were rated as moderate or high rigour. Rigour was assessed by KH with 25% checked by SE

We also assessed relevance for all included papers[32]. Relevance was defined as the alignment of each paper to the review question and initial programme theory, and papers were rated as high, moderate, or low relevance based on the degree of focus on our research question

PPI

We consulted patients and clinicians throughout the review to ensure the review question and programme theory fit with lived experience. Stakeholders contributed to developing the initial and final programme theories, and refined the CMOC list.

For peer review only - http://bmjopen.bmj.com/site/about/guidelines.xhtml

Results

From 4634 search results we included 205 articles (Figure 1). (Cohens K 0.88 for agreement in double screened articles). Articles were mostly from the USA (71), UK (61), and continental Europe (27). Other countries were Canada (14), Australia (20), New Zealand (2), Ghana (1), Hong Kong (1), India (1), and China (1).

Figure 1: PRISMA Chart

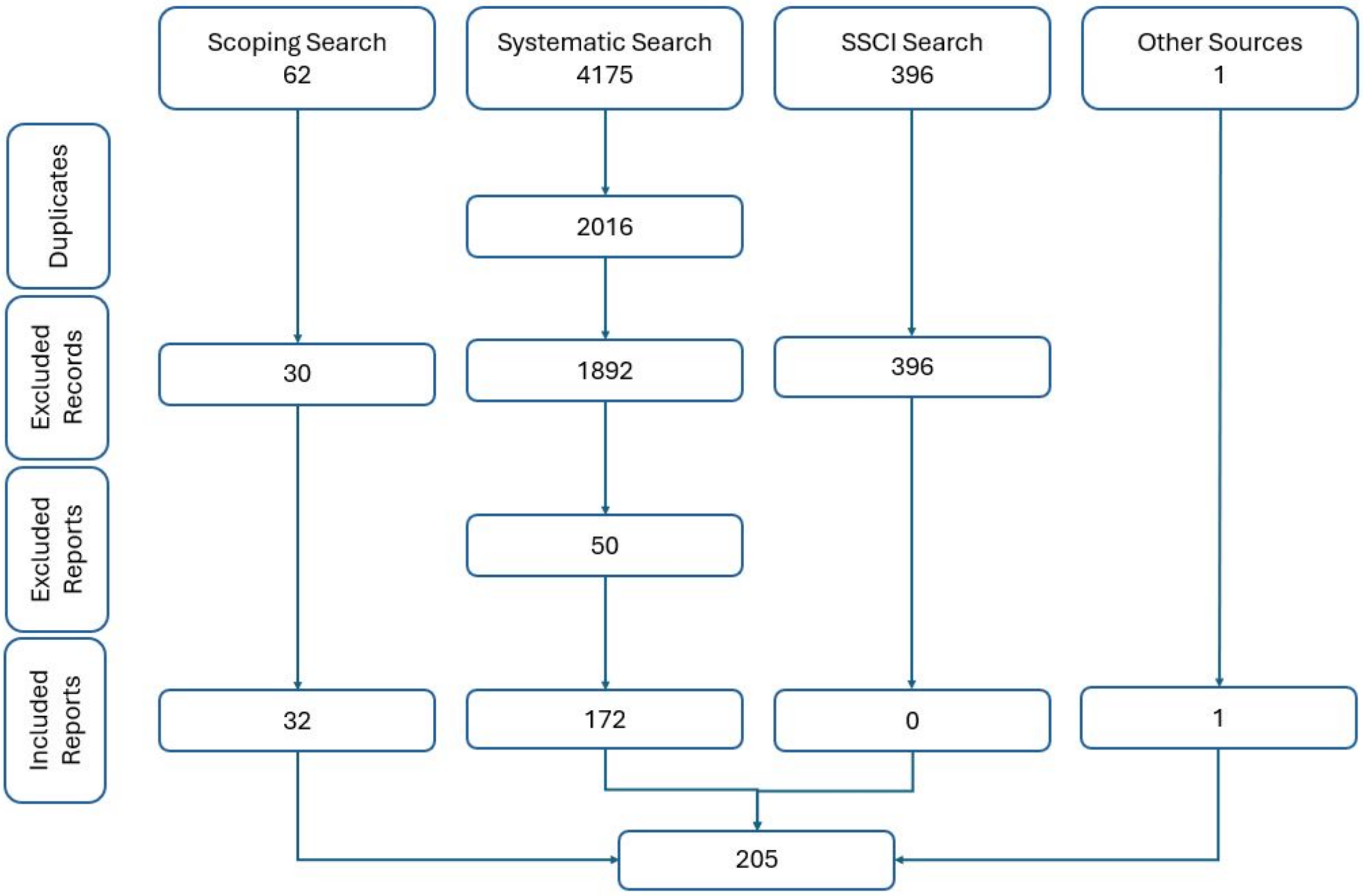

Most articles were qualitative analyses (79) or systematic/narrative literature reviews (71). We also included observational quantitative or mixed-methods studies, theses, policy documents, opinion or discussion articles, and book chapters. The 103 studies involving primary data collection had 46,579 unique participants, including 39,448 patients, 3,959 healthcare professionals, and 2003 carers. 82 articles covered all or mixed settings, with 75 in secondary care, 20 in primary or community care and 11 in hospice settings. 41 focused on HCPs only, 14 on patients, 15 on carers, and 102 on combinations of HCPs, patients and/or carers. The most common patient populations were cancer, heart failure, general palliative care and intensive care.

82 articles were of high rigour, 76 moderate, and 47 low. Most low rigour articles did not involve primary data collection. 63 articles were highly relevant to the programme theory, 91 moderately relevant, and 51 were of low relevance. (appendix 5)

For peer review only - http://bmjopen.bmj.com/site/about/guidelines.xhtml

Context–mechanism–outcome configurations

We identified 52 context-mechanism-outcome configurations [CMOC] within eight thematic areas.1. Patient and family preferences; 2. clinician training and experience; 3. prognostic communication models and interventions; 4. timing and initiation of prognostic dialogue; 5. content of communication; 6. explicit disclosure of uncertainty and open communication; 7. Tacit or avoided disclosure of information and uncertainty. 8. emotions, hope and distress**.** We elaborate on each of these below; examples of how CMOCs relate to the findings are given in the text. The full list of CMOCs is included in appendix 6.

1. *Patient and family preferences*

The inherent uncertainty in life-limiting illness cannot be eliminated[1,33,34] (e.g. difficulty of frailty prognostication[35], rare diseases[36], ICU contexts[37-39]) and patients and carers may expect a degree of prognostic uncertainty within life-limiting illness contexts[40]. Most included studies identified that patients usually want sensitive and honest communication by clinicians when prognosis is uncertain[41-47]; paired with empathy [CMOC9,11] [48-50] and a sense of optimism[17,46] or hopefulness[50-52] (but not false hope[20,38,53-55])[CMOC13]. In order for patients and families to plan ahead in physical, financial, social, emotional domains[36,39,56-58,59] [CMOC6] and make informed decisions at the end of their life[57,60] (e.g. regarding place of death), they need to be given the option of knowing prognostic information alongside realistic expectations and timeframes[61,62]. Unrealistic expectations can lead to overly aggressive treatment plans [CMOC35, 36, 41][51,63-66].

Internationally, although many people desire prognostic estimates and to be fully informed of prognostic uncertainty [CMOC5][17,20,51,56,59,63,65,67-78], information needs vary[36,49,57,69,71-73,79-83]; not all patients want to know [CMOC7][36,41,51,57,75,79,84-87]. In some situations. patients or families may be understood by healthcare professionals as being “in denial”[51,61,72,82], and if so, prognostic disclosure may be perceived to cause distress[52] or challenge a positive outlook[36,73,88,89]. Therefore, providing patients with informational choice and ascertaining individual preferences[7,43,64,67,79,90,91] is an inclusive approach, alongside consideration of sociocultural factors [CMOC1, 10][49,50,53,65,72,78,92,93]. Respecting cultural or social norms and religious or spiritual needs is important [CMOC47, 48][53,55,59,60,93,94] and may influence requests or preferences for prognostic information [CMOC46][50,51,53,60,93,95].

A few studies suggested some patient groups are less likely to want prognostic information such as older patients, those with lower education, higher anxiety, shorter life-expectancy, and non-Northern European cultures[50,72,83]. However, assumptions and stereotyping should be avoided[43,83], particularly as carer and clinician perceptions of patient preference may be inaccurate[50,76,91]. A problematic

For peer review only - http://bmjopen.bmj.com/site/about/guidelines.xhtml

situation can occur where patient and family/carer needs differ [CMOC39][56,76,80,91], for example where families do not want patients to know the prognosis[54,55,72,76,93,96,97] to protect them or sustain hope [CMOC10, 49, 50, 51][72,76,93,97]. Such requests for concealment may challenge norms of autonomy and lead to professionals feeling conflicted[54,96]. A few studies suggested that separate conversations with patients and families may be required [CMOC52][50,80], but erred towards respect for patient needs, consent, and confidentiality[50,72,95].

2. *Clinician training and experience*

A key factor affecting whether discussions of prognostic uncertainty occur, is the baseline clinician tolerance of uncertainty [CMOC 22, 26, 27, 29][1,7,52,66,98,99]. This was influenced by the nature of the relevant illness or condition and clinicians' experience[100-103]. Many studies concur that clinicians may feel underprepared or lack training in how to approach and navigate prognostic discussions[52,71,75,77,90,93,104-109]. As such, a lack of training, and low confidence communicating uncertainty, were key issues in preventing timely and effective prognostic discussions from taking place [CMOC18, 20, 25][41,47,64,86,110-114]. One of the specific training needs identified was skill in prognostication and addressing the tendency of clinicians to overestimate prognosis[54,60,80,108,115] , whether or not they acknowledge uncertainty. A further frequently cited training need was communication skills training[36,61,80,90,91,93,96,116,117]. Some studies report that clinicians with more experience may be more likely to initiate and engage in prognostic discussions[54,112,118,119], although evidence on this is conflicting[112]. Nonetheless, training is needed to enable optimal uncertainty communication practices[1,52].

3. *Prognostic communication models and interventions*

We identified many models to facilitate description of prognosis, and interventions to facilitate its communication.[1,16,40,52,65,70,75,91,97,113,120-125]. These approaches often covered framing of prognostic uncertainty e.g through categorical timeframe predictions[87,115], probabilities of risk[121], probabilistic estimates[40] or likelihoods of death within specified timeframes [87] e.g. 'likelihood a patient will die in the next week or the next month'[115], and individualised survival estimates[126]. Such tools may increase confidence in the degree of uncertainty surrounding a prognosis and so improve disclosure of prognostic information [CMOC17, 40][113], however they cannot eliminate uncertainty inherent to prognostic estimates[17,121]. Notably, prognostication and use and accuracy of tools may be affected by clinician experience[115,127], uncertainty tolerance [CMOC29][66], familiarity with the patient[113], and specialty[115]; clinicians commonly overestimate prognosis[49,115,128] so tools such as the "surprise" question [111] tend to show limited accuracy, although they can promote a holistic approach[111].

For peer review only - http://bmjopen.bmj.com/site/about/guidelines.xhtml

Communication models and interventions specifically addressing communication of uncertain prognosis included the best / worst case framework [CMOC6][94,129,130] , question prompt lists or sheets [43,87], Serious Illness Conversation Guide[65], Psychosocial Assessment and Communication Evaluation (PACE)[1,37], and checking understanding[83,120] and more specifically the 'ask tell ask' approach whereby information known is elicited followed by information provision and checking understanding[41,131]. This is pertinent as discrepancies between doctor's perception of patient's understanding and the patient's perception of understanding are commonly identified[43,50,76,87]. Gauging and clarifying a desire for information was also described as a "drip-feed" communication strategy[52]. Other communication strategies included combining verbal and written information[17,57] (e.g. numerical versus word-based estimates[55,132,133]), breaking bad news strategies such as SPIKES (Setting, Perception, Invitation, Knowledge, Empathy, and Strategy)[65,90,120], balancing hoping for the best against worry[34] or preparing for the worst[44,73,107,134]. Colour-coded pictographs[45] and graphics to represent risks were suggested as useful,[121] Though one study found that patients preferred words to diagrams[55].

4. *Timing and initiation of prognostic dialogue*

Timing of prognostic conversations was widely referenced[43,47,57,95,109,135-137], with some healthcare professionals delaying prognostic discussions until the patient was closer to the end of their life and uncertainty was reduced [CMOC26][36,60,63,77,86,107,122,138]. A patient appearing 'well' may increase clinician reluctance to initiate prognostic discussions[35,36,41]. A person's needs and preferences may change over time[49,50,57,112] so multiple opportunities for conversations and continuous communication is key[36,41,47,60,112,139,140]; the content and timing of the discussion may need negotiating[43,52] and the 'right time' [61] may vary between individuals[36,61,106,141]. The suggested time point for prognostic conversations was often early in the course of illness[51,52,74,77,105,112,116,127,139,142] i.e. at the time of or shortly after diagnosis[17,50,52,57,82,83] so that patients are well enough to be active in conversation[41]. Several studies cited patients, families, and specialist palliative clinicians, who felt that prognostic uncertainty was raised too late[20,69,86,126,143-146]; this missed opportunity can cause regret for caregivers, and may lead to unresolved bereavement narratives [CMOC7, 21][69,144].

Some studies noted ethical and legal principles affecting initiation of discussions. In addition to promotion of autonomy, truth-telling, and informed consent[18,43,84], it is a legal requirement in many countries that discussions about prognosis take place[43] and are documented in the dying person's care record[53,117,147]. However, despite requirements for disclosure and documentation, prognostic discussions do not necessarily occur in practice, and the effect of prognostic uncertainty on discussions in this context is not known[62,93,138 52,115]. There a further risk, or perception of risk, that appearing unsure, or providing an inaccurate prognosis could cause patient or

For peer review only - http://bmjopen.bmj.com/site/about/guidelines.xhtml

reputational harm[128,148]. The ethical duty of preventing harm can therefore increase reluctance to initiate prognostic uncertainty discussions[74,93].

Important considerations for initiating conversations about prognosis include establishing readiness[52,132,133,149,150] e.g. recognising cues[82], as well as current understanding [CMOC1][53,83,138,149,151,152] and preferences[69,91,107], consent to disclose[61], and asking how much they would like to know[46,53,132,133,153,154]. The physical setting can also be pertinent [CMOC45][83], it must be safe, confidential and have appropriate people present[52,53,59,72,83,155]. However, in practice such conversations can be either planned or spontaneous [156]. In person was usually deemed most appropriate[90] ,not soon after surgery[57] or by phone[96].

Prognostic uncertainty conversations should be ideally led by clinicians with a good relationship and knowledge of the patient [CMOC8][52,59,72,124,128,157]. Disclosure of prognostic uncertainty in this context, may strengthen trust [CMOC12][36,48,55,58,71,124]. In some families and cultures (e.g. Chinese[76]), it may be normalised or preferred that patients are informed of their prognosis via family members[59,93] and/or that the family decide what information is shared with the patient[72,76,93]. Nonetheless, many studies argued that healthcare professionals are ultimately responsible for raising prognostic uncertainty[43,63,71,82,86,127,140,158]. Professionals may, however, feel that another clinician or specialty may be better placed to lead the conversation[74,86,154,159]. Ambiguity as to whom should initiate a discussion can lead to hesitation and avoidance[62,73,77,107,108,112,116,135,144], especially in situations of poorly coordinated care. Healthcare professional attitudes and behaviours may vary by region or country[72,93] with some cultures deeming prognostic disclosure as prejudicial[93]. Other factors affecting timely conversations were: resource constraints such as limited time [CMOC43][60,93,104,111,113,160], particularly during appointments[44,77,82,86,93,110]; healthcare professional reluctance [CMOC44][43,62,68,138,161]; paternalistic medical culture[43]; fear of causing depression and distress[58,78,110] or patient harm[93,108] e.g. through taking away hope[75,78,104,113]; lack of private or suitable space for the discussion[114]; language barriers[54,59]; lack of healthcare communication skills and confidence in communicating uncertainty [CMOC18, 20][41,47,86,110-113]; lack of training[52,75,104,105]; fear of damaging the clinician-patient relationship[118,132,133], and notably uncertainty itself as a “barrier” [CMOC27][38,48,60,66,104,107,108,111,112,123,140,146,162,163].

The mechanism of healthcare professionals having difficulty in ‘managing their own emotions’ as a professional[135] as well as the impact of uncertainty on their professional identity and sense of competence and knowledge, could lead to the outcome of failing to raise prognostic uncertainty [100,124]. One study suggested that perception of time taken to explain prognostic uncertainty may be greater than the actual time required[104]. However, these discussions need to go ‘at the patient’s own pace’[41]. There is a balance in a healthcare professional raising the topic of prognosis and uncertainty whilst retaining honesty and fostering hope[86,150]; for this reason, some healthcare professionals waited to discuss prognosis until asked by the patient [CMOC4][36,41,47,73,82,86,93,111,143]. Discussions may also be indirectly initiated by

For peer review only - http://bmjopen.bmj.com/site/about/guidelines.xhtml

healthcare professionals and then 'made explicit by patients'[120] or vice versa[73,80,128]. On the contrary, some patients preferred, waited for, or expected clinicians to initiate[36,41,47,61,71,86,107,154], whilst other patients initiated and/or controlled such discussions[36,72,73,128,150]. Moreover, there is evidence of relatives or companions requesting prognostic information or 'time estimate requests' to initiate the dialogue with the healthcare professional [CMOC4][70,73,128,144,151]. There is a risk that if all parties wait for others to initiate, along with the other issues identified above, a conversation does not take place[41,105,117,119]. Open and timely negotiation of who is responsible for initiating prognostic conversations is required[119].

5. *Content of communication*

Tailoring the language used to the recipient and illness context with compassion are essential when discussing an uncertain prognosis[61,82]. Some patients may prefer sensitivity and others may prioritise directness[61]. Medical jargon without explanation, ambiguous language, and euphemisms should be avoided [CMOC32][20,50,83,117,149,151]; health professionals are directed to use specific terms such as 'death' and 'dying'[83,151], although language should be individualised, and in high context cultures such directness may be less appropriate[120].

These conversations also provide patients and their families opportunities to ask questions and talk about their fears[53]. There was a tendency for patients and families to prefer clinicians to be honest about their prognosis and the uncertainty of this[40-43,104,164]; they often value clear, timely and consistent prognostic information about what is likely to happen and the expected disease progression and life expectancy[59,91,106,112,128]. Although many patients may prefer explicit communication of statistics and survival likelihoods [17,71-73,84], others may prefer vagueness or ambiguity to minimise shock and distress, so an individualised or personalised approach is important[43,67,70,76].

Closing the conversation should include summarisation of the discussion alongside signposting to resources and support[165], including planning for the future[52,128] and symptom control[166], and any follow up[90]. Some clinicians and patients or families prefer to end the discussion on "certainty" or controllable topics such as pain management and reassurance about prioritisation of the patient's comfort [CMOC23][166]; this is sometimes referred to as emphasising the positive[71,80,131,166].

6. *Explicit disclosure of uncertainty and honest communication*

A patient's prognosis and individual disease progression is inherently uncertain[39,104,149,151,153] and therefore clinicians tolerating and normalising[144] this uncertainty is important for a patient- and family- centred approach[34,49] and improving palliative and end of life care [CMOC3][39,167]. Research

For peer review only - http://bmjopen.bmj.com/site/about/guidelines.xhtml

evidence[34,52,72,93,95,110,149,151,168] and guidance[79,164] acknowledges the potential benefits of open, sensitive, and honest communication regarding a patient's prognosis (e.g. patient and family satisfaction[20,49,55,70,75], enhanced wellbeing[58], increased trust[20,40,49,58] and understanding[65,112]), including explicitly disclosing the uncertainty in predicting their prognosis[169]. Honest and explicit communication is evidenced in a range of disease contexts such as heart failure[86,164], respiratory disease[34], advanced kidney disease [107], COPD [105], cancer [CMOC16][17,170], and stroke [CMOC19][33,171]. Many, but not all[55,169], patients and families appreciate clear, truthful, and compassionate communication of prognosis, including discussion of uncertainty[40,149,151,171-173] as this can be pivotal to inform decision making [CMOC2, 6, 14][43,55,70] in respect of a patient's preferences, values, and end of life wishes[33,52,53,60,103,108,140,163].

The uncertainty involved in prognostication, and risk of being wrong, may prompt health professional avoidance[61,123,173], but often patients do not expect health professionals to know exactly how long someone has left to live[61,174]. Therefore, language recommended for acknowledging uncertainty, whilst being honest and informative, is to consider a range of possible scenarios [CMOC6], or to use indicative timeframes e.g. days, weeks, months or years[52,61,80,128,149,151,166,175] rather than exact timeframes[55,59,93,113,115,153]. Honesty about uncertainty in prognostic information does not mean clinicians necessarily declaring they 'don't know'[149,151], which may be detrimental to patient trust/confidence [7,48]. Rather, healthcare professionals can highlight the irreducible nature of prognostic uncertainty alongside a clear and tailored explanation [CMOC37][166], whilst framing information with phrases such as 'I think'[99,149,151,176] and qualifiers/hedging 'could' 'probably'[120,151]. It is often helpful for patients and families to understand how and why prognosis is uncertain and why it may be estimated as 'months rather than years'[61,128], based on evidence such as explaining their signs, symptoms, and deterioration in an accessible manner[59,149,151,166] without overestimation[55,108,115]. For example, the phrase 'sick enough to die' is cited as an example of how to communicate death as a possible or probable outcome[20,79]. Such communication and strategies should allow a clinician to explicitly state that a prognosis is uncertain without undermining their experience, training or role in the relationship [CMOC3][39,151]. However, explicit disclosure of prognostic uncertainty in isolation is not enough[7,52], this must be coupled with skilled delivery[58], sensitivity[49,51,79,91,120], and appropriate content of information[34] in order not to trigger negative effects such as false hope or damaging the relationship[59,61]. Clinicians should try to emphasise the value of supportive care available and non-abandonment[52,73,80,177], if possible, alongside acknowledging prognostic uncertainty and addressing the patient's and families emotions[144], to make it known that such care and support can be continued [CMOC9, CMOC11][49,80].

7. *Tacit or avoided disclosure of information and uncertainty*

For peer review only - http://bmjopen.bmj.com/site/about/guidelines.xhtml

Clinicians may not know what to say[119,162] and may therefore avoid disclosing prognostic information to patients and carers [CMOC21][40,60,61,106,123,138,150,172,173]. Two of the main reasons cited in the literature for such avoidance were prognostic uncertainty itself [CMOC27, 28][107,111,112,119,159] and fear of taking away hope[1,60,75,78,104,113,150,169,172,178]. This healthcare professional avoidance or tacit communication may take several forms in practice including expressing uncertainty as certainty [CMOC22, 31, 38][113,124] (e.g. to influence decision making) [99,108], denial[95], indirect or vague terms[71,124,179], excessive optimism[71,80,108] or avoidance of giving bad news [CMOC33][41,77,96], providing a lack of information (both quantity and quality)[142] or lack of clarity [CMOC30] [72,142], focussing on now or treatments[38] rather than the future[41,72,77], delaying the disclosure[36,60,77,86,107,138], or complete avoidance of the conversation altogether [124,179]. Unless such avoidance was motivated by patients' and carers' preferences, it tended to trigger negative outcomes[93] for patients and/or families such as feeling ill prepared for the future [68,142]/not knowing what to expect[162], lack of advance care planning [CMOC34, 42][36,62], frustration[57,136,142] and anger[61], diminishing trust of clinicians[61,138], poorer satisfaction[50], and isolation[56,142]. Notably, clinician avoidance of talking about prognostic uncertainty may be driven by their own emotional discomfort, or fear of causing distress to patients[47,50,58,71,78,106-108,110,124,144,180].

8. *Emotions, hope and distress*

Hope was a central motivator affecting whether communication of prognostic uncertainty occurred. A common contributing factor to clinician reluctance to disclose prognosis and prognostic uncertainty is desire to preserve hope, and fear of causing harm, anxiety, upset, or distress[40,50,58,78,93,106,108,110,112,144]. Conversely, some evidence[62,82] suggests that avoiding discussion of uncertainty can lead to false hope [CMOC 38], and also that patients *without* clear prognostic information and awareness may be 'more likely to experience anxiety'[76] and prognostic outcomes are not necessarily distressing[78,116] or depressing[75,76,112]; however, there is mixed and contrasting evidence on these outcomes [60]. Furthermore, patients may be more harmed by the dishonesty of clinicians delaying, not asking or sharing their prognosis with them [CMOC24][50,62,75,95]; this can lead to mistrust in healthcare professionals[69] and depression as well as unresolved bereavement narratives[40,69,144]. Relatedly, another determinant of prognostic conversations is often clinician concern of 'a loss of hope',[41,59,66,75,78,90,104,113] which can lead to introduction of prognostic uncertainty to promote (false) hope [CMOC33][55]. However, so long as patients want to engage in prognostic discussions, there is limited evidence that such information decreases hope[70,112] and indeed some may view uncertainty itself as 'a source of hope'[40,48,98] which may reduce anxiety[44]; therefore, clinician's fears of taking away hope may often be unfounded [CMOC13][70,93,112,172]. Despite this, there exists a paradox of patients and carers wanting to be informed, all the while fearing the future[1] and the emotional impact of "knowing"[128]; this is complex for clinicians to navigate and

For peer review only - http://bmjopen.bmj.com/site/about/guidelines.xhtml

requires clarity, skill and compassion[69,75,98]. For example in Cagle et al. (2016)[59], one carer expressed the sentiment that, *"regardless of the potential to take away hope, she would want to know"* (p.7)[59].

For peer review only

For peer review only - http://bmjopen.bmj.com/site/about/guidelines.xhtml

Based on discussion of these themes with stakeholder and PPI contributors, we developed a final programme theory (**figure 2)**. A more detailed description of the theory is available as a supplementary file (appendix 7). References for all included articles are available in appendix 8.

Figure 2. Final programme theory of how communication of prognostic uncertainty 'works', for whom, and in what contexts

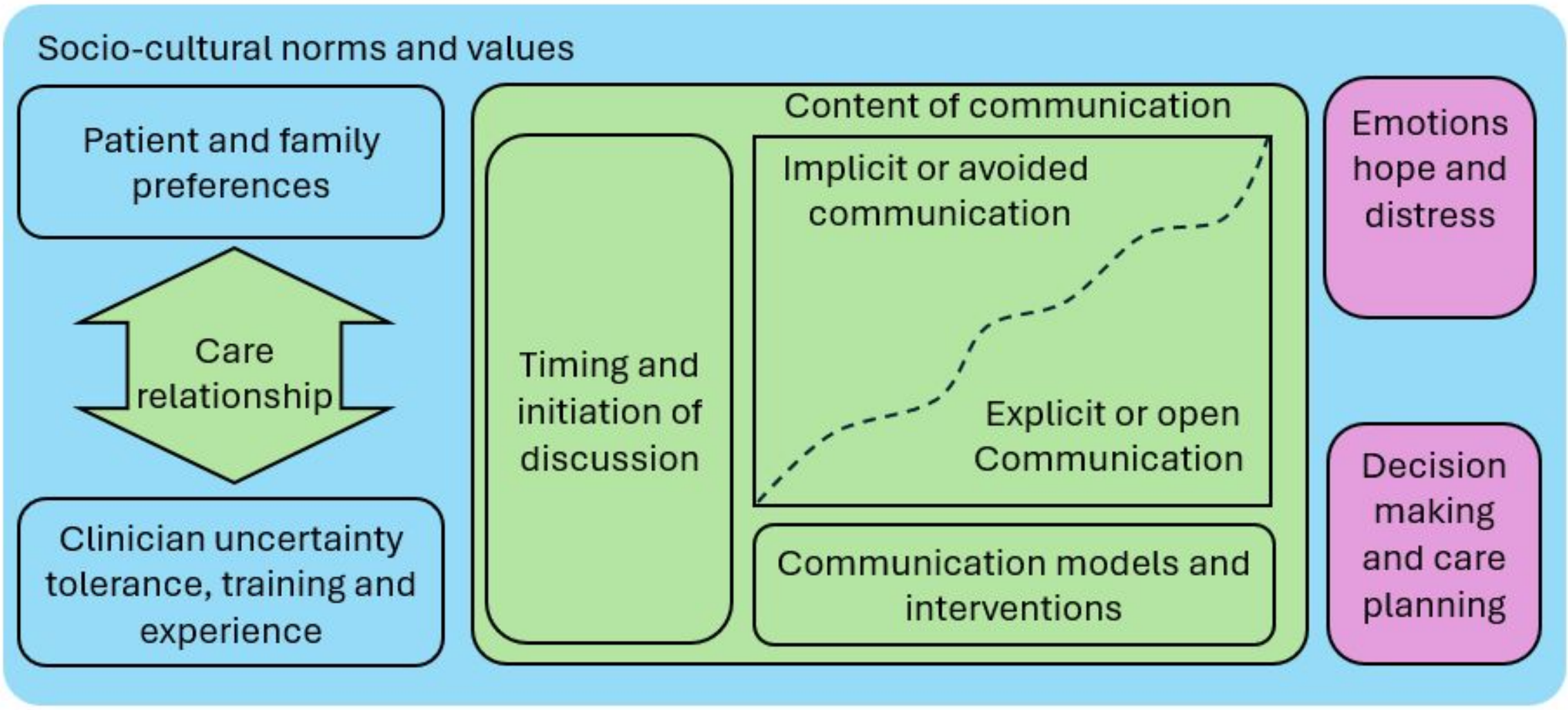

For peer review only - http://bmjopen.bmj.com/site/about/guidelines.xhtml

Discussion:

Statement of principle findings

The programme theory developed in this review characterises contexts and mechanisms that influence whether and how prognostic uncertainty is communicated between healthcare professionals, patients and carers, and the outcomes affected by such communication. We found that when communicating an uncertain prognosis, the degree of explicitness affects shared understanding and trust between clinicians and patients/carers, impacting experiences of distress, acceptance, and future care planning. Key contextual factors including sociocultural norms and values, variation in individual communication preferences, and clinician tolerance of uncertainty shape how communication occurs. Although widely recognised as important, there was limited evidence on how various sociocultural factors affect communication.

Strengths and limitations

We reviewed extensive evidence including academic papers, guidelines and theses. We were therefore able to consider a range of advanced illness contexts: whilst many papers focused on malignancy, a variety of other illness and care contexts were included. The interdisciplinary research team and regular stakeholder input increased trustworthiness of the findings.

The stakeholder-informed realist approach involved patient and clinician perspectives throughout, alongside in-depth evidence synthesis. Stakeholders particularly contributed to generation of the programme theory, and to refining the CMOCs. Communication, prognosis and uncertainty are complex and nuanced concepts; interrogating them through realist synthesis enabled us to go beyond description to develop an explanatory theory[25].

In line with the realist approach, we aimed to synthesise sufficient literature to develop a programme theory rather than finding all existing relevant literature. The review was therefore not exhaustive. However, we are confident that our searches identified adequate literature to inform a robust theory. Despite the breadth of our search, which was not limited by language, most included articles originated from the USA, Europe and Australia, so perspectives from elsewhere could be under-represented.

Comparison with existing literature:

This is, to our knowledge, the first realist synthesis of evidence regarding communication of prognostic uncertainty. There is however an overlapping body of evidence on communication of prognosis, which identifies prognostic uncertainty as a problem in communication and care delivery[93,181]. We extend the evidence base by interrogating

For peer review only - http://bmjopen.bmj.com/site/about/guidelines.xhtml

prognostic uncertainty itself and exploring underlying mechanisms affecting its communication.

Literature reviews on prognostic communication in cancer support these findings, including the need for knowledge and expertise about the patient, to individually tailor disclosure and establish individual, nuanced patient preferences[17]. The complex interplay between honesty, hope and uncertainty is known[17,72,182], and the theory presented here goes some way to explaining that relationship in the context of prognostic communication. Honesty and explicit yet sensitive disclosure of uncertainty can foster patient-clinician trust and, contrary to some clinicians' fears, do not universally destroy hope. Findings by Clayton et al [2008] that hope can be derived from many different sources in advanced illness contexts, indicate that accurate prognostic predictions are not patients' sole sources of hope[183]. Disclosure of prognostic uncertainty could therefore be reframed in a more nuanced way.

Sociocultural factors influence preferences and attitudes towards prognostic information[17,93,125]. Examples include cultural tendencies towards implicit disclosure and euphemism[184], (mis)perceptions that discussing death is (socially) a "taboo"[185], and paradigms of cultural collectivism[125]. Whilst sociocultural factors seem particularly important in shaping attitudes to communication of prognostic uncertainty, there is limited supporting empirical evidence, and assumptions or generalisations based upon perceived cultural background should be avoided.

Meaning & implications

This programme theory provides a novel understanding of the contexts and mechanisms that support effective communication of prognostic uncertainty in serious illness and palliative care, demonstrating the complexity and nuance behind such conversations.

The theory presented here can support professionals in individualised and compassionate communication of prognostic uncertainty to reduce distress, enhance trust, and enable shared care planning. Whilst prognostic discussions are sometimes avoided or delayed until uncertainty is reduced, earlier and/or more transparent communication could improve decision making and timely access to services. Healthcare professionals should engage with prognostic uncertainty and reflect upon their own experience, tolerance, training and confidence in communicating it, given the potential for such factors to influence the occurrence and helpfulness of prognostic conversations. A standardised approach is unhelpful; flexibility and reflexivity could help professionals to communicate with sensitivity and respect for the individual and their sociocultural background.

For peer review only

For peer review only - http://bmjopen.bmj.com/site/about/guidelines.xhtml

Unanswered questions and future research

Future research should focus on translation of these insights into clinician training and practice guidelines, exploring how this programme theory may inform the design and evaluation of approaches to care that integrate prognostic uncertainty, such as parallel planning for multiple possible health outcomes. We found little evidence regarding written communication of prognostic uncertainty and further research here is needed. To help clinicians develop individualised approaches to prognostic communication, future research should also explore how sociocultural factors manifest when communicating prognostic uncertainty in practice and how such factors could be sensitively approached.

For peer review only

For peer review only - http://bmjopen.bmj.com/site/about/guidelines.xhtml

List of Appendices

1. RAMESES checklist
2. initial programme theory
3. search strategy
4. extraction proforma
5. details of included articles
6. Context Mechanism Outcome Configuration table
7. detailed programme theory
8. supplementary references

Acknowledgements

We would like to thank the patient and public involvement contributors as well as clinical stakeholders who provided their expertise and guidance for this review work, and helped us refine the programme theory.

Transparency:

The lead author affirms that the manuscript is an honest, accurate, and transparent account of the study being reported; that no important aspects of the study have been omitted; and that any discrepancies from the study as registered on Prospero have been explained.

Funding/conflicts of interest

This project was funded by Marie Curie (grant number MCSGS-23-201/ G763113)

The University of Birmingham supported Dr Weetman's time for drafting the manuscript.

Dr MacArtney is supported by a Marie Curie Fellowship (2023-26) ref: MCRFS-18-101, and has received funding from Marie Curie, NIHR, Australian Research Council for other projects in the past 36 months

Dr Etkind has received funding from Addenbrookes Charitable Trust, Marie Curie, Alborada Trust for other projects in the past 36 months

All authors have completed the Unified Competing Interest form (available on request from the corresponding author) and declare: no support from any organisation for the submitted work other than that described above; no financial relationships with any organisations that might have an interest in the submitted work in the previous three years other than that described above; no other relationships or activities that could appear to have influenced the submitted work.

For peer review only - http://bmjopen.bmj.com/site/about/guidelines.xhtml

Contributorship

The corresponding author attests that all listed authors meet authorship criteria and that no others meeting the criteria have been omitted

- Project conceptualisation and securing funding: JM, KW, SE
- Design and methodology FS, JM, KW, SE
- Searching, screening and data extraction, FS, KH, KW, SE, TA
- Synthesis KH, KW, SE, TA
- Writing of initial draft FS, KH, KW, SE, TA
- Review and approval of final manuscript FS, JM, KH, KW, SE, TA
- Dr Etkind and Dr MacArtney are joint guarantors of this manuscript and its contents

For peer review only

For peer review only - http://bmjopen.bmj.com/site/about/guidelines.xhtml

References

1. Etkind SN, Koffman J. Approaches to managing uncertainty in people with life-limiting conditions: role of communication and palliative care. Postgrad Med J. 2016;92(1089):412-7.
2. Han PK, Klein WM, Arora NK. Varieties of Uncertainty in Health Care A Conceptual Taxonomy. Medical Decision Making. 2011;31(6):828-38.
3. Etkind SN, Li J, Louca J, Hopkins SA, Kuhn I, Spathis A, et al. Total Uncertainty: A systematic review and thematic synthesis of experiences of uncertainty in older people with advanced multimorbidity, their informal carers, and health professionals. . Age and Ageing 2022;51(8).
4. Etkind SN, Bristowe K, Bailey K, Selman LE, Murtagh FEM. How does uncertainty shape patient experience in advanced illness? A secondary analysis of qualitative data. Palliative Medicine. 2016;31(2):171-80.
5. Etkind SN, Koffman J. Approaches to managing uncertainty in people with life-limiting conditions: role of communication and palliative care. Postgraduate Medical Journal. 2016.
6. MacLeod RD. Certainty is an illusion: lessons for palliative care. Journal of the Royal Society of Medicine. 2024;117(5):161-4.
7. Medendorp NM, Stiggelbout AM, Aalfs CM, Han PKJ, Smets EMA, Hillen MA. A scoping review of practice recommendations for clinicians' communication of uncertainty. Health Expect. 2021;24(4):1025-43.
8. Etkind SN, Barclay S, Spathis A, Hopkins SA, Bowers B, Koffman J. Uncertainty in serious illness: A national interdisciplinary consensus exercise to identify clinical research priorities. PLoS ONE. 2024;19(2):e0289522.
9. Kingston A, Robinson L, Booth H, Knapp M, Jagger C, for the Mp. Projections of multi-morbidity in the older population in England to 2035: estimates from the Population Ageing and Care Simulation (PACSim) model. Age and Ageing. 2018;47(3):374-80.
10. Murray SA, Boyd K, Moine S, Kendall M, Macpherson S, Mitchell G, et al. Using illness trajectories to inform person centred, advance care planning. BMJ (Clinical research ed). 2024;384:e067896.
11. Leach I, Mayland C, Turner N, Mitchell S. Understanding patient views and experiences of the IDENTIfication of PALLiative care needs (IDENTI-PALL): a qualitative interview study. British Journal of General Practice. 2023.
12. Tobin J, Rogers A, Winterburn I, Tullie S, Kalyanasundaram A, Kuhn I, et al. Hospice care access inequalities: a systematic review and narrative synthesis. BMJ supportive & palliative care. 2022;12(2):142-51.
13. Gold Standards Famework Proactive Identification Guidance [Available from: https://www.goldstandardsframework.org.uk/wp-content/uploads/2025/07/Proactive-Identification-Guidance.pdf.
14. Etkind SN, Bone AE, Lovell N, Gomes B, Evans CE, Murtagh FE, et al. How many people will need palliative care in 2040? Past trends, future projections and implications for services. BMC Medicine. 2017(15):102.
15. Marmot M. Health equity in England: the Marmot review 10 years on. BMJ (Clinical research ed). 2020;368.
16. Hui D, Paiva CE, Del Fabbro EG, Steer C, Naberhuis J, van de Wetering M, et al. Prognostication in advanced cancer: update and directions for future research. Support Care Cancer. 2019;27(6):1973-84.

For peer review only - http://bmjopen.bmj.com/site/about/guidelines.xhtml

17. Johnson M, Tod AM, Brummell S, Collins K. Prognostic communication in cancer: A critical interpretive synthesis of the literature. Eur J Oncol Nurs. 2015;19(5):554-67.
18. Simpkin AL, Armstrong KA. Communicating Uncertainty: a Narrative Review and Framework for Future Research. Journal of general internal medicine. 2019;34(11):2586-91.
19. Cox CL, Miller BM, Kuhn I, Fritz Z. Diagnostic uncertainty in primary care: what is known about its communication, and what are the associated ethical issues? Family Practice. 2021;38(5):654-68.
20. Krawczyk M, Gallagher R. Communicating prognostic uncertainty in potential end-of-life contexts: experiences of family members. BMC palliative care. 2016;15:59.
21. Clayton JM, Hancock KM, Butow PN, Tattersall MH, Currow DC, Adler J, et al. Clinical practice guidelines for communicating prognosis and end-of-life issues with adults in the advanced stages of a life-limiting illness, and their caregivers. Med J Aust. 2007;186(S12):S77-s105.
22. Broeder P. Informed communication in high context and low context cultures. Journal of Education, Innovation and Communication. 2021;3(1):13-24.
23. Hallenbeck J. High context illness and dying in a low context medical world. American Journal of Hospice and Palliative Medicine®. 2006;23(2):113-8.
24. Wong G, Greenhalgh T, Westhorp G, Buckingham J, Pawson R. RAMESES publication standards: realist syntheses. BMC Medicine. 2013;11(1):21.
25. Pawson R, Greenhalgh T, Harvey G, Walshe K. Realist synthesis-an introduction. ESRC res methods program. 2004;2:55.
26. Pawson R, Greenhalgh T, Harvey G, Walshe K. Realist review - a new method of systematic review designed for complex policy interventions. Journal of Health Services Research & Policy. 2005;10(1_suppl):21-34.
27. Murray SA, Kendall M, Boyd K, Sheikh A. Illness trajectories and palliative care. BMJ: British Medical Journal. 2005;330(7498):1007.
28. Coleman S, Wright JM, Nixon J, Schoonhoven L, Twiddy M, Greenhalgh J. Searching for Programme theories for a realist evaluation: a case study comparing an academic database search and a simple Google search. BMC medical research methodology. 2020;20(1):217.
29. Booth A, Briscoe S, Wright JM. The “realist search”: a systematic scoping review of current practice and reporting. Research synthesis methods. 2020;11(1):14-35.
30. Booth A, Wright J, Briscoe S. Scoping and searching to support realist approaches. Doing realist research. 2018:147-66.
31. Aali G, Shokraneh F. No limitations to language, date, publication type, and publication status in search step of systematic reviews. Journal of Clinical Epidemiology. 2021;133.
32. Dada S, Dalkin S, Gilmore B, Hunter R, Mukumbang FC. Applying and reporting relevance, richness and rigour in realist evidence appraisals: Advancing key concepts in realist reviews. Research Synthesis Methods. 2023;14(3):504-14.
33. Connolly T, Coats H, DeSanto K, Jones J. The experience of uncertainty for patients, families and healthcare providers in post-stroke palliative and end-of-life care: a qualitative meta-synthesis. Age Ageing. 2021;50(2):534-45.
34. Johnson RF, Jr., Gustin J. Acute lung injury and acute respiratory distress syndrome requiring tracheal intubation and mechanical ventilation in the intensive

For peer review only

care unit: impact on managing uncertainty for patient-centered communication. The American journal of hospice & palliative care. 2013;30(6):569-75.
35. Combes S. Conversations on Living and Dying: Facilitating Advance Care Planning with Older People Living with Frailty. Developing and Refining an Intervention. England: University of London, King's College (United Kingdom); 2021.
36. Gough N, Ross JR, Riley J, Judson I, Koffman J. 'When something is this rare ... how do you know bad really is bad...?'-views on prognostic discussions from patients with advanced soft tissue sarcoma. BMJ Support Palliat Care. 2019;9(1):100-7.
37. Higginson IJ, Koffman J, Hopkins P, Prentice W, Burman R, Leonard S, et al. Development and evaluation of the feasibility and effects on staff, patients, and families of a new tool, the Psychosocial Assessment and Communication Evaluation (PACE), to improve communication and palliative care in intensive care and during clinical uncertainty. BMC medicine. 2013;11:1-14.
38. Gutierrez KM. Communication of prognostic information in an ICU at end of life: practices among and between nurses, physicians and family members: University of Minnesota; 2010.
39. Gutierrez KM. Prognostic categories and timing of negative prognostic communication from critical care physicians to family members at end-of-life in an intensive care unit. Nurs Inq. 2013;20(3):232-44.
40. Evans LR, Boyd EA, Malvar G, Apatira L, Luce JM, Lo B, et al. Surrogate decision-makers' perspectives on discussing prognosis in the face of uncertainty. Am J Respir Crit Care Med. 2009;179(1):48-53.
41. Barclay S, Momen N, Case-Upton S, Kuhn I, Smith E. End-of-life care conversations with heart failure patients: a systematic literature review and narrative synthesis. Br J Gen Pract. 2011;61(582):e49-62.
42. Carlini J, Bahudin D, Michaleff ZA, Plunkett E, She EN, Clark J, et al. Discordance and concordance on perception of quality care at end of life between older patients, caregivers and clinicians: a scoping review. Eur Geriatr Med. 2022;13(1):87-99.
43. Innes S, Payne S. Advanced cancer patients' prognostic information preferences: a review. Palliat Med. 2009;23(1):29-39.
44. Kimbell B, Boyd K, Kendall M, Iredale J, Murray SA. Managing uncertainty in advanced liver disease: a qualitative, multiperspective, serial interview study. BMJ Open. 2015;5(11):e009241.
45. Maxwell CA, Mixon AS, Conner E, Phillippi JC. Receptivity of Hospitalized Older Adults and Family Caregivers to Prognostic Information about Aging, Injury, and Frailty: A Qualitative Study. Int J Nurs Stud. 2020;109:103602.
46. Jackson VA, Emanuel L. Navigating and Communicating about Serious Illness and End of Life. The New England journal of medicine. 2024;390(1):63-9.
47. Momen NC, Barclay SI. Addressing 'the elephant on the table': barriers to end of life care conversations in heart failure - a literature review and narrative synthesis. Current opinion in supportive and palliative care. 2011;5(4):312-6.
48. Parvez S, Abdel-Kader K, Song MK, Unruh M. Conveying uncertainty in prognosis to patients with ESRD. Blood Purif. 2015;39(1-3):58-64.
49. Stanek MS, Norton SA. Expressions of Uncertainty from Patients with Advanced Cancer when Communicating with the Palliative Care Team. United States -- New York: University of Rochester; 2020.

For peer review only - http://bmjopen.bmj.com/site/about/guidelines.xhtml

50. Parker S, Clayton J, Hancock K, Walder S, Butow P, Carrick S, et al. Communicating Prognosis and Issues Surrounding End of Life (EOL) in adults in the advanced stages of a life-limiting illness: A Systematic Review. 2006.
51. Piovesan M, Orr P, Tevyaw S, Roussos E, Cherid C, Bouchard S. A Prospective Study with Patients and Families on the Usefulness of Accurate Prognosis for Palliative Care Patients. J Soc Work End Life Palliat Care. 2024;20(2):133-46.
52. Etkind SN, Fennessy R, Wang J, Simpson RM, O'Neill B, Barclay SIG, et al. Communicating prognostic uncertainties in advanced multimorbidity: a multimethod qualitative study to co-design practice recommendations. Eur Geriatr Med. 2025.
53. Nice. Care of dying adults in the last days of life. NICE. 2015.
54. McLennon SM, Uhrich M, Lasiter S, Chamness AR, Helft PR. Oncology nurses' narratives about ethical dilemmas and prognosis-related communication in advanced cancer patients. Cancer Nurs. 2013;36(2):114-21.
55. van Eenennaam RM, Kruithof WJ, van Es MA, Kruitwagen-van Reenen ET, Westeneng HJ, Visser-Meily JMA, et al. Discussing personalized prognosis in amyotrophic lateral sclerosis: development of a communication guide. BMC Neurol. 2020;20(1):446.
56. Fenton A, Fletcher KM, Kizildag D, Borstelmann NA, Kessler D, Cronin C, et al. Cancer Caregivers' Prognostic and End-of-Life Communication Needs and Experiences and their Impact. J Pain Symptom Manage. 2023;65(1):16-25.
57. Halkett GK, Lobb EA, Oldham L, Nowak AK. The information and support needs of patients diagnosed with High Grade Glioma. Patient Educ Couns. 2010;79(1):112-9.
58. Hui D, Mo L, Paiva CE. The Importance of Prognostication: Impact of Prognostic Predictions, Disclosures, Awareness, and Acceptance on Patient Outcomes. Curr Treat Options Oncol. 2021;22(2):12.
59. Cagle JG, McClymont KM, Thai JN, Smith AK. "If You Don't Know, All of a Sudden, They're Gone": Caregiver Perspectives About Prognostic Communication for Disabled Elderly Adults. J Am Geriatr Soc. 2016;64(6):1299-306.
60. Gray TF, Temel JS, El-Jawahri A. Illness and prognostic understanding in patients with hematologic malignancies. Blood Rev. 2021;45:100692.
61. Marie C. What's stopping doctors having that conversation about death? Marie Curie. 2019.
62. Fallowfield LJ, Jenkins VA, Beveridge HA. Truth may hurt but deceit hurts more: communication in palliative care. Palliat Med. 2002;16(4):297-303.
63. Heyland DK, Allan DE, Rocker G, Dodek P, Pichora D, Gafni A. Discussing prognosis with patients and their families near the end of life: impact on satisfaction with end-of-life care. Open Med. 2009;3(2):e101-10.
64. van der Velden NCA, Han PKJ, van Laarhoven HWM, de Vos F, Hendriks LEL, Burgers SA, et al. Prevalence and Predictors of Physician-Patient Discordance in Prognostic Perceptions in Advanced Cancer. Oncologist. 2023;28(8):e653-e68.
65. Chen JJ, Roldan CS, Nichipor AN, Balboni TA, Krishnan MS, Revette AC, et al. Prognostic Understanding and Goals of Palliative Radiotherapy: A Qualitative Study. J Pain Symptom Manage. 2022;64(6):567-76.
66. Habib AR, Chen R, Magnavita ES, Jaung T, Awad MM, Odejide O, et al. Prevalence and Tolerance of Prognostic Uncertainty Among Thoracic Oncologists. Oncologist. 2021;26(8):e1480-e2.

For peer review only - http://bmjopen.bmj.com/site/about/guidelines.xhtml

67. Harrison M, Gardiner C, Taylor B, Ejegi-Memeh S, Darlison L. Understanding the palliative care needs and experiences of people with mesothelioma and their family carers: An integrative systematic review. Palliat Med. 2021;35(6):1039-51.
68. Cormican O, Dowling M. Providing Care to People Living with a Chronic Hematological Malignancy: A Qualitative Evidence Synthesis of Informal Carers' Experiences. Semin Oncol Nurs. 2022;38(6):151338.
69. Diggle MFJ, Schutz S, Butcher D. Bereaved Caregivers' Experiences of End of Life Care For People With Advanced Heart Failure: A Narrative Synthesis. Omega (Westport). 2025;90(4):1581-608.
70. Dickerson LK, Lipson TA, Chauhan SSB, Allen GI, Young B, Park JO, et al. Evaluating surgeon communication of pancreatic cancer prognosis using the VitalTalk ADAPT framework. J Surg Oncol. 2024;130(3):476-84.
71. Geerse OP, Lamas DJ, Sanders JJ, Paladino J, Kavanagh J, Henrich NJ, et al. A Qualitative Study of Serious Illness Conversations in Patients with Advanced Cancer. Journal of palliative medicine. 2019;22(7):773-81.
72. Hagerty RG, Butow PN, Ellis PM, Dimitry S, Tattersall MH. Communicating prognosis in cancer care: a systematic review of the literature. Ann Oncol. 2005;16(7):1005-53.
73. Henselmans I, Smets EMA, Han PKJ, de Haes H, Laarhoven H. How long do I have? Observational study on communication about life expectancy with advanced cancer patients. Patient Educ Couns. 2017;100(10):1820-7.
74. Hoff L, Hermeren G. Between uncertainty and certainty. The Journal of clinical ethics. 2011;22(2):139-50.
75. Holloway RG, Gramling R, Kelly AG. Estimating and communicating prognosis in advanced neurologic disease. Neurology. 2013;80(8):764-72.
76. Chan WC. Being aware of the prognosis: how does it relate to palliative care patients' anxiety and communication difficulty with family members in the Hong Kong Chinese context? Journal of palliative medicine. 2011;14(9):997-1003.
77. Stocker R, Close H, Hancock H, Hungin APS. Should heart failure be regarded as a terminal illness requiring palliative care? A study of heart failure patients', carers' and clinicians' understanding of heart failure prognosis and its management. BMJ Support Palliat Care. 2017;7(4):464-9.
78. Disler R, Pascoe A, Luckett T, Donesky D, Irving L, Currow DC, et al. Barriers to Palliative Care Referral and Advance Care Planning (ACP) for Patients With COPD: A Cross-Sectional Survey of Palliative Care Nurses. The American journal of hospice & palliative care. 2022;39(2):169-77.
79. British Geriatrics Society. End of Life Care in Frailty: Identification and prognostication. CLINICAL GUIDELINES. 2020.
80. Chu C, Anderson R, White N, Stone P. Prognosticating for Adult Patients With Advanced Incurable Cancer: a Needed Oncologist Skill. Curr Treat Options Oncol. 2020;21(1):5.
81. Hjelmfors L, Strömberg A, Friedrichsen M, Mårtensson J, Jaarsma T. Communication About the Heart Failure Trajectory in Patients, Their Families and Health Care Professionals. Sweden: Linkopings Universitet (Sweden); 2018.
82. Kimbell B. Living, dying and caring in advanced liver disease: the challenge of uncertainty. Scotland: The University of Edinburgh (United Kingdom); 2014.
83. Parker SM, Clayton JM, Hancock K, Walder S, Butow PN, Carrick S, et al. A systematic review of prognostic/end-of-life communication with adults in the advanced stages of a life-limiting illness: patient/caregiver preferences for the

For peer review only - http://bmjopen.bmj.com/site/about/guidelines.xhtml

content, style, and timing of information. J Pain Symptom Manage. 2007;34(1):81-93.
84. Smith AK, Glare P. Ethical issues in prognosis and prognostication. The Oxford handbook of ethics at the end of life. 2014:170-89.
85. Marie C. Difficult conversations with dying people and their families: Marie Curie; 2014.
86. Stocker R. Experiences and perceptions of the role of palliative and end of life care in heart failure: a modified grounded theory study. England: University of Durham (United Kingdom); 2014.
87. Michael N, Shah R. Communication about Advanced Progressive Disease , Prognosis, and Advance Care Plans. In: Kissane DWMW, William B, editors. Psycho-Oncology in Palliative and End of Life Care. New York: Oxford Academic; 2022. p. 1-C.P207.
88. Nagelschmidt K, Leppin N, Seifart C, Rief W, von Blanckenburg P. Systematic mixed-method review of barriers to end-of-life communication in the family context. BMJ Support Palliat Care. 2021;11(3):253-63.
89. Chou WS, Hamel LM, Thai CL, Debono D, Chapman RA, Albrecht TL, et al. Discussing prognosis and treatment goals with patients with advanced cancer: A qualitative analysis of oncologists' language. Health Expect. 2017;20(5):1073-80.
90. Becker C, Beck K, Vincent A, Hunziker S. Communication challenges in end-of-life decisions. Swiss medical weekly. 2020;150:w20351.
91. Bristowe K, Carey I, Hopper A, Shouls S, Prentice W, Caulkin R, et al. Patient and carer experiences of clinical uncertainty and deterioration, in the face of limited reversibility: A comparative observational study of the AMBER care bundle. Palliat Med. 2015;29(9):797-807.
92. Ko E, Fuentes D, Singh-Carlson S, Nedjat-Haiem F. Challenges and facilitators of hospice decision-making: a retrospective review of family caregivers of home hospice patients in a rural US-Mexico border region-a qualitative study. BMJ Open. 2020;10(7):e035634.
93. Hancock K, Clayton JM, Parker SM, Wal der S, Butow PN, Carrick S, et al. Truth-telling in discussing prognosis in advanced life-limiting illnesses: a systematic review. Palliat Med. 2007;21(6):507-17.
94. Hendershot KA, Elias MN, Taylor BL, Wahlster S, Creutzfeldt CJ. An Update on Palliative Care in Neurocritical Care: Providing Goal-Concordant Care in the Face of Prognostic Uncertainty. Curr Treat Options Neurol. 2023;25(12):517-29.
95. Fisher M, Ridley S. Uncertainty in end-of-life care and shared decision making. Critical care and resuscitation : journal of the Australasian Academy of Critical Care Medicine. 2012;14(1):81-7.
96. Marie Curie. Difficult conversations with dying people and their families. 2014 March 2014.
97. Kantabanlang Y, Lin CP, Nilmanat K, Guo P. Palliative care needs of patients with hematologic malignancies and family caregivers and challenges of palliative care provision in Asia: A review of evidence. Semin Hematol. 2023;60(4):220-5.
98. Ridley S, Fisher M. Uncertainty in end-of-life care. Curr Opin Crit Care. 2013;19(6):642-7.
99. Schuster RA, Hong SY, Arnold RM, White DB. Do physicians disclose uncertainty when discussing prognosis in grave critical illness? Narrat Inq Bioeth. 2012;2(2):125-35.

For peer review only - http://bmjopen.bmj.com/site/about/guidelines.xhtml

100. Gofton TE, Chum M, Schulz V, Gofton BT, Sarpal A, Watling C. Challenges facing palliative neurology practice: A qualitative analysis. J Neurol Sci. 2018;385:225-31.
101. Broom A, Kirby E, Good P, Wootton J, Adams J. The troubles of telling: managing communication about the end of life. Qual Health Res. 2014;24(2):151-62.
102. Hudson BF, Shulman C, Low J, Hewett N, Daley J, Davis S, et al. Challenges to discussing palliative care with people experiencing homelessness: a qualitative study. BMJ Open. 2017;7(11):e017502.
103. Pooler C, Richman-Eisenstat J, Kalluri M. Early integrated palliative approach for idiopathic pulmonary fibrosis: A narrative study of bereaved caregivers' experiences. Palliat Med. 2018;32(9):1455-64.
104. Risco JR, Kelly AG, Holloway RG. Prognostication in neurology. Handb Clin Neurol. 2022;190:175-93.
105. Ngwenya N, Crang C, Farquhar M, Rintoul RC, Mahadeva R, Calvert LD, et al. Communicating uncertainty: contrasting the communication experiences of patients with advanced COPD and incurable lung cancer. Fam Pract. 2021;38(5):637-43.
106. Young J, Lyons A, Egan R, Dew K. Embodied decisions unfolding over time: a meta-ethnography systematic review of people with cancer's reasons for delaying or declining end-of-life care. BMC palliative care. 2024;23(1):45.
107. Mandel EI, Bernacki RE, Block SD. Serious Illness Conversations in ESRD. Clin J Am Soc Nephrol. 2017;12(5):854-63.
108. Dias LM, Barra W, Rego F. Advance Care Planning in Oncology: Challenges in Clinical Practice. Portugal: Universidade do Porto (Portugal); 2022.
109. Scholz B, Goncharov L, Emmerich N, Lu VN, Chapman M, Clark SJ, et al. Clinicians' accounts of communication with patients in end-of-life care contexts: A systematic review. Patient Educ Couns. 2020;103(10):1913-21.
110. Chen JJ, Gamble K, Graham-Wisener L, McGlade K, Doherty J, Donnelly P, et al. GP perceptions of the adequacy of community-based care for patients with advanced heart failure in a UK region (NI): a qualitative study. Open Heart. 2018;5(1):e000734.
111. Gerlach C, Goebel S, Weber S, Weber M, Sleeman KE. Space for intuition - the 'Surprise'-Question in haemato-oncology: Qualitative analysis of experiences and perceptions of haemato-oncologists. Palliat Med. 2019;33(5):531-40.
112. Habib AR, Cronin AM, Earle CC, Tulsky JA, Mack JW, Abel GA, et al. How Do Blood Cancer Doctors Discuss Prognosis? Findings from a National Survey of Hematologic Oncologists. Journal of palliative medicine. 2019;22(6):677-84.
113. Hallen SA, Hootsmans NA, Blaisdell L, Gutheil CM, Han PK. Physicians' perceptions of the value of prognostic models: the benefits and risks of prognostic confidence. Health Expect. 2015;18(6):2266-77.
114. Low J, Davis S, Vickerstaff V, Greenslade L, Hopkins K, Langford A, et al. Advanced chronic liver disease in the last year of life: a mixed methods study to understand how care in a specialist liver unit could be improved. BMJ Open. 2017;7(8):e016887.
115. Chu C, White N, Stone P. Prognostication in palliative care. Clin Med (Lond). 2019;19(4):306-10.
116. Pfeil TA, Laryionava K, Reiter-Theil S, Hiddemann W, Winkler EC. What keeps oncologists from addressing palliative care early on with incurable cancer patients? An active stance seems key. Oncologist. 2015;20(1):56-61.

For peer review only - http://bmjopen.bmj.com/site/about/guidelines.xhtml

117. Bloomer MJ, Botti M, Runacres F, Poon P, Barnfield J, Hutchinson AM. Communicating end-of-life care goals and decision-making among a multidisciplinary geriatric inpatient rehabilitation team: A qualitative descriptive study. Palliat Med. 2018;32(10):1615-23.
118. Masterson MP, Applebaum AJ, Buda K, Reisch S, Rosenfeld B. Don't Shoot the Messenger: Experiences of Delivering Prognostic Information in the Context of Advanced Cancer. The American journal of hospice & palliative care. 2018;35(12):1526-31.
119. Tavares NC. Palliative Care Communication in Copd : Patients' Preferences and Clinicians' Judgements. England: University of Southampton (United Kingdom); 2019.
120. Ekberg S, Parry R, Land V, Ekberg K, Pino M, Antaki C, et al. Communicating with patients and families about illness progression and end of life: a review of studies using direct observation of clinical practice. BMC palliative care. 2021;20(1):186.
121. Harrison M, Han PKJ, Rabin B, Bell M, Kay H, Spooner L, et al. Communicating uncertainty in cancer prognosis: a review of web-based prognostic tools. Patient education and counseling. 2019;102(5):842-9.
122. Reid C, Gibbins J, Bloor S, Burcombe M, McCoubrie R, Forbes K. Healthcare professionals' perspectives on delivering end-of-life care within acute hospital trusts: a qualitative study. BMJ Support Palliat Care. 2015;5(5):490-5.
123. Bailey SJ, Cogle K. Talking about dying: How to begin honest conversations about what lies ahead: Royal College of Physicians; 2018.
124. Epstein RM. Facing epistemic and complex uncertainty in serious illness: The role of mindfulness and shared mind. Patient Educ Couns. 2021;104(11):2635-42.
125. Pun J, Chow JCH, Fok L, Cheung KM. Role of patients' family members in end-of-life communication: an integrative review. BMJ Open. 2023;13(2):e067304.
126. Narayan M, Jones J, Portalupi LB, McIlvennan CK, Matlock DD, Allen LA. Patient Perspectives on Communication of Individualized Survival Estimates in Heart Failure. J Card Fail. 2017;23(4):272-7.
127. Owusuaa C, van Beelen I, van der Heide A, van der Rijt CCD. Physicians' views on the usefulness and feasibility of identifying and disclosing patients' last phase of life: a focus group study. BMJ Support Palliat Care. 2021;14(e2).
128. van der Velden NCA, van der Kleij MBA, Lehmann V, Smets EMA, Stouthard JML, Henselmans I, et al. Communication about Prognosis during Patient-Initiated Second Opinion Consultations in Advanced Cancer Care: An Observational Qualitative Analysis. Int J Environ Res Public Health. 2021;18(11).
129. Lee CW, Evans E, Vitous CA, Suwanabol PA. Living with uncertainty in surgery: integrating palliative care principles into conversations as a solution to patient and family, provider, and health system disquietude. Ann Palliat Med. 2023;12(1):16-20.
130. Goss AL, Voumard RR, Engelberg RA, Curtis JR, Creutzfeldt CJ. Do They Have a Choice? Surrogate Decision-Making After Severe Acute Brain Injury. Crit Care Med. 2023;51(7):924-35.
131. Goodlin SJ, Quill TE, Arnold RM. Communication and decision-making about prognosis in heart failure care. J Card Fail. 2008;14(2):106-13.
132. van der Velden NCA, Smets EMA, van Vliet LM, Brom L, van Laarhoven HWM, Henselmans I. Effects of prognostic communication strategies on emotions, coping, and appreciation of consultations: An experimental study in advanced cancer. Palliat Support Care. 2024:1-13.

For peer review only - http://bmjopen.bmj.com/site/about/guidelines.xhtml

133. van der Velden NCA, Smets EMA, van Vliet LM, Brom L, van Laarhoven HWM, Henselmans I. Effects of Prognostic Communication Strategies on Prognostic Perceptions, Treatment Decisions and End-Of-Life Anticipation in Advanced Cancer: An Experimental Study among Analogue Patients. J Pain Symptom Manage. 2024;67(6):478-89 e13.
134. Powers BA, Norton SA, Schmitt MH, Quill TE, Metzger M. Meaning and practice of palliative care for hospitalized older adults with life limiting illnesses. J Aging Res. 2011;2011:406164.
135. Cottrell L, Economos G, Evans C, Silber E, Burman R, Nicholas R, et al. A realist review of advance care planning for people with multiple sclerosis and their families. PLoS ONE. 2020;15(10):e0240815.
136. Crowther JL. Dementia: What Care Do Patients and Informal Carers Need in the Last Year of Life and Time Surrounding Death? An Exploratory Qualitative Study. England: The University of Liverpool (United Kingdom); 2011.
137. Mone S, Kerr H. Prognostic awareness in advanced cancer: an integrative literature review. BMJ Support Palliat Care. 2021;11(1):53-8.
138. Hoff L, Hermeren G. Identifying challenges to communicating with patients about their imminent death. The Journal of clinical ethics. 2014;25(4):296-306.
139. Olsson MM, Windsor C, Chambers S, Green TL. A Scoping Review of End-of-Life Communication in International Palliative Care Guidelines for Acute Care Settings. J Pain Symptom Manage. 2021;62(2):425-37 e2.
140. Udo C, Lovgren M, Lundquist G, Axelsson B. Palliative care physicians' experiences of end-of-life communication: A focus group study. Eur J Cancer Care (Engl). 2018;27(1).
141. Clarke G, Fistein E, Holland A, Tobin J, Barclay S, Barclay S. Planning for an uncertain future in progressive neurological disease: a qualitative study of patient and family decision-making with a focus on eating and drinking. BMC Neurol. 2018;18(1):115.
142. Fitzsimons D, Doherty LC, Murphy M, Dixon L, Donnelly P, McDonald K, et al. Inadequate Communication Exacerbates the Support Needs of Current and Bereaved Caregivers in Advanced Heart Failure and Impedes Shared Decision-making. J Cardiovasc Nurs. 2019;34(1):11-9.
143. Trankle SA, Shanmugam S, Lewis E, Nicholson M, Hillman K, Cardona M. Are We Making Progress on Communication with People Who Are Near the End of Life in the Australian Health System? A Thematic Analysis. Health Commun. 2020;35(2):158-67.
144. Robinson J, Pilbeam C, Goodwin H, Raphael D, Waterworth S, Gott M. The impact of uncertainty on bereaved family's experiences of care at the end of life: a thematic analysis of free text survey data. BMC palliative care. 2021;20(1):60.
145. Formagini T, Poague C, O'Neal A, Brooks JV. "When I Heard the Word Palliative": Obscuring and Clarifying Factors Affecting the Stigma Around Palliative Care Referral in Oncology. JCO Oncol Pract. 2022;18(1):e72-e9.
146. Green E, Gardiner C, Gott M, Ingleton C. Exploring the extent of communication surrounding transitions to palliative care in heart failure: the perspectives of health care professionals. Journal of palliative care. 2011;27(2):107-16.
147. Bloomer MJ, Botti M, Runacres F, Poon P, Barnfield J, Hutchinson AM. End-of-life care for older people in subacute care: A retrospective clinical audit. Collegian. 2019;26(1):22-7.

For peer review only - http://bmjopen.bmj.com/site/about/guidelines.xhtml

148. Bruun A, White N, Oostendorp L, Stone P, Bloch S. Time estimates in prognostic discussions: A conversation analytic study of hospice multidisciplinary team meetings. Palliat Med. 2024;38(5):593-601.
149. Anderson RJ. Communication with the Relatives and Friends of Imminently Dying Patients in a Hospice. England: University of London, University College London (United Kingdom); 2020.
150. Walczak A, Butow PN, Davidson PM, Bellemore FA, Tattersall MH, Clayton JM, et al. Patient perspectives regarding communication about prognosis and end-of-life issues: how can it be optimised? Patient Educ Couns. 2013;90(3):307-14.
151. Anderson RJ, Stone PC, Low JTS, Bloch S. Managing uncertainty and references to time in prognostic conversations with family members at the end of life: A conversation analytic study. Palliat Med. 2020;34(7):896-905.
152. Stiel H, Nagarajan SV, Forster BC, Clayton JM. Palliative care physicians' perspectives on transferring patients to nursing homes and communication strategies to facilitate this transition: A qualitative study. Palliat Med. 2019;33(3):323-31.
153. Moglia C, Palumbo F, Botto R, Iazzolino B, Ticozzi N, Calvo A, et al. Prognostic communication in amyotrophic lateral sclerosis: findings from a Nationwide Italian survey. Neurol Sci. 2024;45(12):5787-94.
154. Hjelmfors L, Stromberg A, Friedrichsen M, Martensson J, Jaarsma T. Communicating prognosis and end-of-life care to heart failure patients: a survey of heart failure nurses' perspectives. Eur J Cardiovasc Nurs. 2014;13(2):152-61.
155. Tuck KK, Brod L, Nutt J, Fromme EK. Preferences of patients with Parkinson's disease for communication about advanced care planning. The American journal of hospice & palliative care. 2015;32(1):68-77.
156. Gutierrez KM. Prognostic communication of critical care nurses and physicians at end of life. Dimens Crit Care Nurs. 2012;31(3):170-82.
157. Tavares N, Hunt KJ, Jarrett N, Wilkinson TM. The preferences of patients with chronic obstructive pulmonary disease are to discuss palliative care plans with familiar respiratory clinicians, but to delay conversations until their condition deteriorates: A study guided by interpretative phenomenological analysis. Palliat Med. 2020;34(10):1361-73.
158. Garland EL, Bruce A, Stajduhar K. Exposing barriers to end-of-life communication in heart failure: an integrative review. Canadian journal of cardiovascular nursing = Journal canadien en soins infirmiers cardio-vasculaires. 2013;23(1):12-8.
159. Chang A, Datta-Barua I, McLaughlin B, Daly B. A survey of prognosis discussions held by health-care providers who request palliative care consultation. Palliat Med. 2014;28(4):312-7.
160. Han PK, Dieckmann NF, Holt C, Gutheil C, Peters E. Factors Affecting Physicians' Intentions to Communicate Personalized Prognostic Information to Cancer Patients at the End of Life: An Experimental Vignette Study. Med Decis Making. 2016;36(6):703-13.
161. Chow J, Senderovich H. It's Time to Talk: Challenges in Providing Integrated Palliative Care in Advanced Congestive Heart Failure. A Narrative Review. Curr Cardiol Rev. 2018;14(2):128-37.
162. Nadicksbernd J. Why Do Community Healthcare Professionals Find Some Conversations Difficult? England: University of London, Royal Holloway College (United Kingdom); 2023.

For peer review only

163. Gonella S, Basso I, Dimonte V, Giulio PD. The role of end-of-life communication in contributing to palliative-oriented care at the end-of-life in nursing home. Int J Palliat Nurs. 2022;28(1):16-26.
164. Nice. Chronic heart failure in adults: diagnosis and management. NICE. 2018.
165. Marie C. Tips for talking to your patients about a prognosis. Marie Curie. 2020.
166. Anderson RJ, Stone PC, Low JTS, Bloch S. Transitioning out of prognostic talk in discussions with families of hospice patients at the end of life: A conversation analytic study. Patient Educ Couns. 2021;104(5):1075-85.
167. Szawarski P. Classic cases revisited: Oscar the cat and predicting death. J Intensive Care Soc. 2016;17(4):341-5.
168. Anstey S. Clinical thinking, knowledge and the referral practices of consultant physicians to specialist palliative care services: an interpretative phenomenological analysis. Wales: Cardiff University (United Kingdom); 2012.
169. Wijsenbeek MS, Bonella F, Orsatti L, Russell AM, Valenzuela C, Wuyts WA, et al. Communicating with patients with idiopathic pulmonary fibrosis: can we do it better? ERJ Open Res. 2022;8(1):00422-2021.
170. Temel JS, Shaw AT, Greer JA. Challenge of Prognostic Uncertainty in the Modern Era of Cancer Therapeutics. Journal of clinical oncology : official journal of the American Society of Clinical Oncology. 2016;34(30):3605-8.
171. Payne S, Burton C, Addington-Hall J, Jones A. End-of-life issues in acute stroke care: a qualitative study of the experiences and preferences of patients and families. Palliat Med. 2010;24(2):146-53.
172. Gadoud AC. A palliative care approach for people with advanced heart failure: recognition of need, transitions in care and impact on patients, family carers and clinicians. England: University of Hull (United Kingdom); 2013.
173. Tolsa L, Jones L, Michel P, Borasio GD, Jox RJ, Rutz Voumard R. 'We Have Guidelines, but We Can Also Be Artists': Neurologists Discuss Prognostic Uncertainty, Cognitive Biases, and Scoring Tools. Brain Sci. 2022;12(11):1591.
174. Richards N, Ingleton C, Gardiner C, Gott M. Awareness contexts revisited: indeterminacy in initiating discussions at the end-of-life. J Adv Nurs. 2013;69(12):2654-64.
175. Parry R. Communication in Palliative Care and About End of Life: A State-of-the-Art Literature Review of Conversation-Analytic Research in Healthcare. Research on Language and Social Interaction. 2024;57(1):127-48.
176. Wu Y, Zhang X. Examining Conversation Analysis in Palliative Care: A Systematic Review. Health Commun. 2024;39(13):3072-83.
177. van der Velden NCA, Meijers MC, Han PKJ, van Laarhoven HWM, Smets EMA, Henselmans I. The Effect of Prognostic Communication on Patient Outcomes in Palliative Cancer Care: a Systematic Review. Curr Treat Options Oncol. 2020;21(5):40.
178. Walczak A, Butow PN, Bu S, Clayton JM. A systematic review of evidence for end-of-life communication interventions: Who do they target, how are they structured and do they work? Patient Educ Couns. 2016;99(1):3-16.
179. Han PK. The Need for Uncertainty: A Case for Prognostic Silence. Perspect Biol Med. 2016;59(4):567-75.
180. Noble H, Brazil K, Burns A, Hallahan S, Normand C, Roderick P, et al. Clinician views of patient decisional conflict when deciding between dialysis and conservative management: Qualitative findings from the PAlliative Care in chronic Kidney diSease (PACKS) study. Palliat Med. 2017;31(10):921-31.

For peer review only - http://bmjopen.bmj.com/site/about/guidelines.xhtml

181. Tavares N, Jarrett N, Hunt K, Wilkinson T. Palliative and end-of-life care conversations in COPD: a systematic literature review. ERJ Open Res. 2017;3(2).
182. Anderson RJ, Bloch S, Armstrong M, Stone PC, Low JT. Communication between healthcare professionals and relatives of patients approaching the end-of-life: A systematic review of qualitative evidence. Palliat Med. 2019;33(8):926-41.
183. Clayton JM, Hancock K, Parker S, Butow PN, Walder S, Carrick S, et al. Sustaining hope when communicating with terminally ill patients and their families: a systematic review. Psychooncology. 2008;17(7):641-59.
184. Chung I, Khoo SY, Low LL. An Insight Into the Experiences of Malaysian Patients With Advanced Cancer and Their Preferences in End-of-Life Care: A Qualitative Study. The American journal of hospice & palliative care. 2025;42(1):5-13.
185. DeBoer RJ, Wabl CA, Mushi BP, Uwamahoro P, Athanas R, Ndoli DA, et al. A scoping review of clinical communication in cancer care in Africa. The Oncologist. 2025;30(4):oyaf039.

For peer review only

For peer review only - http://bmjopen.bmj.com/site/about/guidelines.xhtml

Appendix 1. Rameses reporting checklist

	Item	details	Reporting
TITLE			
1		In the title, identify the document as a realist synthesis or review	Title
ABSTRACT			
2		While acknowledging publication requirements and house style, abstracts should ideally contain brief details of: the study's background, review question or objectives; search strategy; methods of selection, appraisal, analysis and synthesis of sources; main results; and implications for practice.	Abstract
INTRODUCTION			
3	Rationale for review	Explain why the review is needed and what it is likely to contribute to existing understanding of the topic area.	Background P3
4	Objectives and focus of review	State the objective(s) of the review and/or the review question(s). Define and provide a rationale for the focus of the review.	P3
METHODS			

For peer review only - http://bmjopen.bmj.com/site/about/guidelines.xhtml

	Item	details	Reporting
5	Changes in the review process	Any changes made to the review process that was initially planned should be briefly described and justified.	n/a
6	Rationale for using realist synthesis	Explain why realist synthesis was considered the most appropriate method to use.	P4
7	Scoping the literature	Describe and justify the initial process of exploratory scoping of the literature.	P4-5
8	Searching processes	While considering specific requirements of the journal or other publication outlet, state and provide a rationale for how the iterative searching was done. Provide details on all the sources accessed for information in the review. Where searching in electronic databases has taken place, the details should include, for example, name of database, search terms, dates of coverage and date last searched. If individuals familiar with the relevant literature and/or topic area were contacted, indicate how they were identified and selected.	P5
9	Selection and appraisal of documents	Explain how judgements were made about including and excluding data from documents, and justify these.	P5

For peer review only - http://bmjopen.bmj.com/site/about/guidelines.xhtml

	Item	details	Reporting
10	Data extraction	Describe and explain which data or information were extracted from the included documents and justify this selection.	P5
11	Analysis and synthesis processes	Describe the analysis and synthesis processes in detail. This section should include information on the constructs analyzed and describe the analytic process.	P5-6
RESULTS			
12	Document flow diagram	Provide details on the number of documents assessed for eligibility and included in the review with reasons for exclusion at each stage as well as an indication of their source of origin (for example, from searching databases, reference lists and so on). You may consider using the example templates (which are likely to need modification to suit the data) that are provided.	Figure 1
13	Document characteristics	Provide information on the characteristics of the documents included in the review.	P7, supplementary information
14	Main findings	Present the key findings with a specific focus on theory building and testing.	P7-16
DISCUSSION			

For peer review only - http://bmjopen.bmj.com/site/about/guidelines.xhtml

	Item	details	Reporting
15	Summary of findings	Summarize the main findings, taking into account the review's objective(s), research question(s), focus and intended audience(s).	P17
16	Strengths, limitations and future research directions	Discuss both the strengths of the review and its limitations. These should include (but need not be restricted to) (a) consideration of all the steps in the review process and (b) comment on the overall strength of evidence supporting the explanatory insights which emerged.<br><br>The limitations identified may point to areas where further work is needed.	P17
17	Comparison with existing literature	Where applicable, compare and contrast the review's findings with the existing literature (for example, other reviews) on the same topic.	P17-18
18	Conclusion and recommendation s	List the main implications of the findings and place these in the context of other relevant literature. If appropriate, offer recommendations for policy and practice.	P18-19
19	Funding	Provide details of funding source (if any) for the review, the role played by the funder (if any) and any conflicts of interests of the reviewers.	P20

For peer review only - http://bmjopen.bmj.com/site/about/guidelines.xhtml

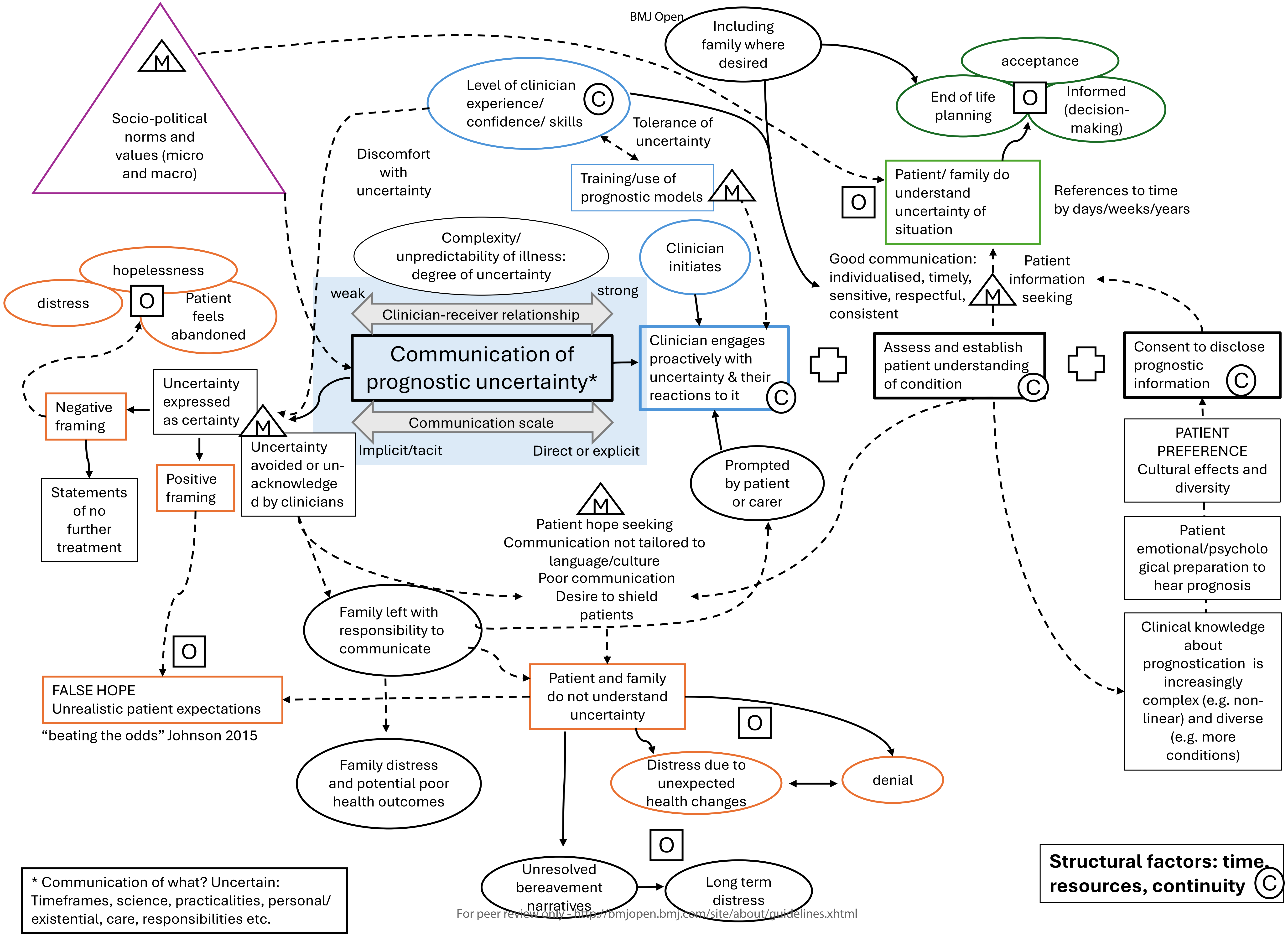
M
Socio-political norms and values (micro and macro)
Including family where desired
acceptance
End of life planning
O
Informed (decision-making)
Level of clinician experience/ confidence/ skills
C
Tolerance of uncertainty
Discomfort with uncertainty
Training/use of prognostic models
M
O
Patient/ family do understand uncertainty of situation
References to time by days/weeks/years
Complexity/ unpredictability of illness: degree of uncertainty
Clinician initiates
Good communication: individualised, timely, sensitive, respectful, consistent
M
Patient information seeking
hopelessness
distress
O
Patient feels abandoned
weak
strong
Clinician-receiver relationship
Communication of prognostic uncertainty*
Communication scale
Implicit/tacit
Direct or explicit
Clinician engages proactively with uncertainty & their reactions to it
C
Assess and establish patient understanding of condition
C
Consent to disclose prognostic information
C
Negative framing
Uncertainty expressed as certainty
M
Uncertainty avoided or un-acknowledge d by clinicians
Positive framing
Statements of no further treatment
M
Patient hope seeking
Communication not tailored to language/culture
Poor communication
Desire to shield patients
Prompted by patient or carer
PATIENT PREFERENCE Cultural effects and diversity
Patient emotional/psychological preparation to hear prognosis
Clinical knowledge about prognostication is increasingly complex (e.g. non-linear) and diverse (e.g. more conditions)
Family left with responsibility to communicate
O
FALSE HOPE Unrealistic patient expectations
"beating the odds" Johnson 2015
Patient and family do not understand uncertainty
O
Family distress and potential poor health outcomes
Distress due to unexpected health changes
denial
O
Unresolved bereavement narratives
Long term distress
Structural factors: time, resources, continuity
C
* Communication of what? Uncertain: Timeframes, science, practicalities, personal/ existential, care, responsibilities etc.

MEDLINE via Ovid SP

Database: Ovid MEDLINE(R) ALL <1946 to August 15, 2024>

1 Communication Barriers/ or Health Communication/ or Nonverbal Communication/ or Teach-Back Communication/ or Communication/ or Disclosure/ or Truth Disclosure/ or Interpersonal Relations/ or Professional-Patient Relations/ or Nurse-Patient Relations/ or Physician-Patient Relations/ or Patient Discharge Summaries/ or (Communicat* or Conversation* or Discussing or Discussed or Discuss or Discusses or Disclos* or Dialog* or Nurse-Patient Relation* or Patient-Nurse Relation* or Doctor-Patient Relation* or Physician-Patient Relation* or Patient-Doctor Relation* or Patient-Physician Relation* or Patient-Health Care Professional Relation* or Patient-Health Professional Relation* or Patient-Health Professional Relation* or Patient-Healthcare Professional Relation* or Patient-Professional Relation* or Professional-Patient Relation* or Interpersonal or Patient Discharge Summar* or Patient Discharge Letter*).ti,ab. (2281379)

2 Prognosis/ or Life Expectancy/ or Disease Progression/ or Clinical Deterioration/ or (Prognos* or Predict* or Forecast* or Life Expectanc* or Disease Progress* or Disease Exacerbat* or Deteriorat*).ti,ab. (3456789)

3 Uncertainty/ or (Uncertain* or Indetermin* or Ambigu* or Unpredictab* or Vague* or Doubt*).ti,ab. (395891)

4 Palliative Care/ or Palliative Medicine/ or Hospice Care/ or Hospices/ or "Hospice and Palliative Care Nursing"/ or Terminal Care/ or Death/ or Bereavement/ or (Palliat* or Hospice* or Bereav* or Terminal* or End-of-Life or Death* or Advanced Illness* or Advanced Disease* or Life-Limiting or Die or Dying or Bereav*).ti,ab. (1810528)

5 and/1-4 (920)

Embase via Ovid SP

Database: Embase <1974 to 2024 August 16>

1 Interpersonal Communication/ or Communication Skill/ or Communication Barrier/ or Nonverbal Communication/ or Verbal Communication/ or Conversation/ or Written Communication/ or Professional-Patient Relationship/ or Nurse Patient Relationship/ or Doctor Patient Relationship/ or Patient Centered Communication/ or (Communicat* or Conversation* or Discussing or Discussed or Discuss or Discusses or Disclos* or Dialog* or Nurse-Patient Relation* or Patient-Nurse Relation* or Doctor-Patient Relation* or Physician-Patient Relation* or Patient-Doctor Relation* or Patient-Physician Relation* or Patient-Health Care Professional Relation* or Patient-Health Professional Relation* or Patient-Health Professional Relation* or Patient-Healthcare Professional Relation* or Patient-Professional Relation* or Professional-Patient Relation* or Interpersonal or Patient Discharge Summar* or Patient Discharge Letter*).ti,ab. (2667971)

2 Prognosis/ or Life Expectancy/ or Disease Exacerbation/ or Deterioration/ or (Prognos* or Predict* or Forecast* or Life Expectanc* or Disease Progress* or Disease Exacerbat* or Deteriorat* or Disease Aggravat*).ti,ab. (4642834)

3 Uncertainty/ or Intolerance of Uncertainty Scale/ or Mishel Uncertainty in Illness Scale/ or Mishel Uncertainty in Illness Theory/ or (Uncertain* or Indetermin* or Ambigu* or Unpredictab* or Vague* or Doubt* or Mishel*).ti,ab. (519031)

For peer review only

4 exp Palliative Therapy/ or Palliative Nursing/ or Hospice Care/ or Hospice/ or Hospice Patient/ or Hospice Nursing/ or Terminal Care/ or Terminally Ill Patient/ or exp Terminal Disease/ or Death/ or Dying/ or Bereavement/ or (Palliat* or Hospice* or Bereav* or Terminal* or End-of-Life or Death* or Advanced Illness* or Advanced Disease* or Life-Limiting or Die or Dying or Bereav*).ti,ab. (2504665)

5 and/1-4 (1753)

6 limit 5 to embase (764)

PsycINFO via Ovid SP

Database: APA PsycInfo <1806 to August 2024 Week 3>

1 Communication/ or Communication Barriers/ or Nonverbal Communication/ or Verbal Communication/ or Conversation/ or Written Communication/ or Communication Skills/ or Communication Skills Training/ or Interpersonal Interaction/ or Interpersonal Communication/ or (Communicat* or Conversation* or Discussing or Discussed or Discuss or Discusses or Disclos* or Dialog* or Nurse-Patient Relation* or Patient-Nurse Relation* or Doctor-Patient Relation* or Physician-Patient Relation* or Patient-Doctor Relation* or Patient-Physician Relation* or Patient-Health Care Professional Relation* or Patient-Health Professional Relation* or Patient-Health Professional Relation* or Patient-Healthcare Professional Relation* or Patient-Professional Relation* or Professional-Patient Relation* or Interpersonal or Patient Discharge Summar* or Patient Discharge Letter*).ti,ab. (1205127)

2 Prognosis/ or Life Expectancy/ or Disease Progression/ or (Prognos* or Predict* or Forecast* or Life Expectanc* or Disease Progress* or Disease Exacerbat* or Deteriorat*).ti,ab. (619978)

3 exp Uncertainty/ or "Ambiguity (Non-Stimulus)"/ or Doubt/ or (Uncertain* or Indetermin* or Ambigu* or Unpredictab* or Vague* or Doubt*).ti,ab. (130464)

4 Palliative Care/ or Hospice/ or Terminally Ill Patients/ or Terminal Cancer/ or "Death and Dying"/ or Bereavement/ or (Palliat* or Hospice* or Bereav* or Terminal* or End-of-Life or Death* or Advanced Illness* or Advanced Disease* or Life-Limiting or Die or Dying or Bereav*).ti,ab. (186761)

5 and/1-4 (261)

CINAHL via EBSCOhost

Limiters - Exclude MEDLINE records

(((MH "Communication") OR (MH "Communication Barriers") OR (MH "Communication Skills") OR (MH "Communication Skills Training") OR (MH "Conversation") OR (MH "Nonverbal Communication") OR (MH "Truth Disclosure") OR (MH "Professional-Patient Relations") OR (MH "Physician-Patient Relations") OR (MH "Nurse-Patient Relations") OR (MH "Interpersonal Relations") OR (MH "Patient Discharge Summaries")) OR TI (Communicat* OR Conversation* OR Discussing OR Discussed OR Discuss OR Discusses OR Disclos* OR Dialog* OR Nurse-Patient Relation* OR Patient-Nurse Relation* OR Doctor-Patient Relation* OR Physician-Patient Relation* OR Patient-Doctor Relation* OR Patient-Physician Relation* OR Patient-Health Care Professional Relation* OR Patient-Health Professional Relation* OR Patient-Health Professional Relation* OR Patient-Healthcare Professional Relation* OR Patient-Professional Relation* OR Professional-Patient Relation* OR Interpersonal OR Patient Discharge Summar* OR Patient Discharge Letter*) OR AB (

For peer review only

Communicat* OR Conversation* OR Discussing OR Discussed OR Discuss OR Discusses OR Disclos* OR Dialog* OR Nurse-Patient Relation* OR Patient-Nurse Relation* OR Doctor-Patient Relation* OR Physician-Patient Relation* OR Patient-Doctor Relation* OR Patient-Physician Relation* OR Patient-Health Care Professional Relation* OR Patient-Health Professional Relation* OR Patient-Health Professional Relation* OR Patient-Healthcare Professional Relation* OR Patient-Professional Relation* OR Professional-Patient Relation* OR Interpersonal OR Patient Discharge Summar* OR Patient Discharge Letter*)) AND (((MH "Prognosis") OR (MH "Life Expectancy") OR (MH "Disease Progression") OR (MH "Clinical Deterioration")) OR TI (Prognos* OR Predict* OR Forecast* OR Life Expectanc* OR Disease Progress* OR Disease Exacerbat* OR Deteriorat*) OR AB (Prognos* OR Predict* OR Forecast* OR Life Expectanc* OR Disease Progress* OR Disease Exacerbat* OR Deteriorat*)) AND (((MH "Uncertainty") OR (MH "Mishel Uncertainty in Illness Theory") OR (MH "Mishel Uncertainty in Illness Scale")) OR TI (Uncertain* OR Indetermin* OR Ambigu* OR Unpredictab* OR Vague* OR Doubt*) OR AB (Uncertain* OR Indetermin* OR Ambigu* OR Unpredictab* OR Vague* OR Doubt*)) AND (((MH "Palliative Care") OR (MH "Palliative Care Nurses") OR (MH "Palliative Care Nursing") OR (MH "Palliative Medicine") OR (MH "Hospice Nurses") OR (MH "Hospice Patients") OR (MH "Hospice Nursing") OR (MH "Hospice Care") OR (MH "Hospices") OR (MH "National Association for Home Care & Hospice") OR (MH "Hospice and Palliative Nurses Association") OR (MH "Hospice (Saba CCC)") OR (MH "Terminal Care") OR (MH "Terminal Care (Saba CCC)") OR (MH "Terminally Ill Patients") OR (MH "Death") OR (MH "Bereavement")) OR TI (Palliat* OR Hospice* OR Bereav* OR Terminal* OR End-of-Life OR Death* OR Advanced Illness* OR Advanced Disease* OR Life-Limiting OR Die OR Dying OR Bereav*) OR AB (Palliat* OR Hospice* OR Bereav* OR Terminal* OR End-of-Life OR Death* OR Advanced Illness* OR Advanced Disease* OR Life-Limiting OR Die OR Dying OR Bereav*))

214

Cochrane Database of Systematic Reviews

Issue 8 of 12, August 2024

Date Run: 18/08/2024 22:03:17

([mh "Communication Barriers"] or [mh "Health Communication"] or [mh "Nonverbal Communication"] or [mh "Teach-Back Communication"] or [mh Communication] or [mh Disclosure] or [mh "Truth Disclosure"] or [mh "Interpersonal Relations"] or [mh "Professional-Patient Relations"] or [mh "Nurse-Patient Relations"] or [mh "Physician-Patient Relations"] or [mh "Patient Discharge Summaries"] or (Communicat* or Conversation* or Discussing or Discussed or Discuss or Discusses or Disclos* or Dialog* or Nurse-Patient Relation* or Patient-Nurse Relation* or Doctor-Patient Relation* or Physician-Patient Relation* or Patient-Doctor Relation* or Patient-Physician Relation* or Patient-Health Care Professional Relation* or Patient-Health Professional Relation* or Patient-Health Professional Relation* or Patient-Healthcare Professional Relation* or Patient-Professional Relation* or Professional-Patient Relation* or Interpersonal or Patient Discharge Summar* or Patient Discharge Letter*):ti,ab) AND ([mh Prognosis] or [mh "Life Expectancy"] or [mh "Disease Progression"] or [mh "Clinical Deterioration"] or (Prognos* or Predict* or Forecast* or Life Expectanc* or Disease Progress* or Disease Exacerbat* or Deteriorat*):ti,ab) AND ([mh Uncertainty] or (Uncertain* or Indetermin* or Ambigu* or Unpredictab* or Vague* or Doubt*):ti,ab) AND ([mh "Palliative Care"] or [mh "Palliative Medicine"] or [mh "Hospice Care"] or [mh Hospices] or [mh "Hospice and Palliative Care Nursing"] or [mh "Terminal Care"] or [mh Death] or [mh Bereavement] or (Palliat* or Hospice* or Bereav* or Terminal* or End-of-Life or Death* or Advanced Illness* or Advanced Disease* or Life-Limiting or Die or Dying or Bereav*):ti,ab)

25

ProQuest Dissertations & Theses Global

18 August 2024 15:09

S1 title(Communicat* or Conversation* or Discussing or Discussed or Discuss or Discusses or Disclos* or Dialog* or Nurse-Patient Relation* or Patient-Nurse Relation* or Doctor-Patient Relation* or Physician-Patient Relation* or Patient-Doctor Relation* or Patient-Physician Relation* or Patient-Health Care Professional Relation* or Patient-Health Professional Relation* or Patient-Health Professional Relation* or Patient-Healthcare Professional Relation* or Patient-Professional Relation* or Professional-Patient Relation* or Interpersonal or Patient Discharge Summar* or Patient Discharge Letter*) OR abstract(Communicat* or Conversation* or Discussing or Discussed or Discuss or Discusses or Disclos* or Dialog* or Nurse-Patient Relation* or Patient-Nurse Relation* or Doctor-Patient Relation* or Physician-Patient Relation* or Patient-Doctor Relation* or Patient-Physician Relation* or Patient-Health Care Professional Relation* or Patient-Health Professional Relation* or Patient-Health Professional Relation* or Patient-Healthcare Professional Relation* or Patient-Professional Relation* or Professional-Patient Relation* or Interpersonal or Patient Discharge Summar* or Patient Discharge Letter*) 11884219

S2 title(Prognos* or Predict* or Forecast* or Life Expectanc* or Disease Progress* or Disease Exacerbat* or Deteriorat*) OR abstract(Prognos* or Predict* or Forecast* or Life Expectanc* or Disease Progress* or Disease Exacerbat* or Deteriorat*) 7103093

S3 title(Uncertain* or Indetermin* or Ambigu* or Unpredictab* or Vague* or Doubt*) OR abstract(Uncertain* or Indetermin* or Ambigu* or Unpredictab* or Vague* or Doubt*) 2471013

S4 title(Palliat* or Hospice* or Bereav* or Terminal* or End-of-Life or Death* or Advanced Illness* or Advanced Disease* or Life-Limiting or Die or Dying or Bereav*) OR abstract(Palliat* or Hospice* or Bereav* or Terminal* or End-of-Life or Death* or Advanced Illness* or Advanced Disease* or Life-Limiting or Die or Dying or Bereav*) 14366671

S5 [S1] AND [S2] AND [S3] AND [S4] 319

Science Citation Index-Expanded via Web of Science

Web of Science Search Strategy (v0.1)

1: Communicat* or Conversation* or Discussing or Discussed or Discuss or Discusses or Disclos* or Dialog* or Nurse-Patient Relation* or Patient-Nurse Relation* or Doctor-Patient Relation* or Physician-Patient Relation* or Patient-Doctor Relation* or Patient-Physician Relation* or Patient-Health Care Professional Relation* or Patient-Health Professional Relation* or Patient-Health Professional Relation* or Patient-Healthcare Professional Relation* or Patient-Professional Relation* or Professional-Patient Relation* or Interpersonal or Patient Discharge Summar* or Patient Discharge Letter* (Title) OR Communicat* or Conversation* or Discussing or Discussed or Discuss or Discusses or Disclos* or Dialog* or Nurse-Patient Relation* or Patient-Nurse Relation* or Doctor-Patient Relation* or Physician-Patient Relation* or Patient-Doctor Relation* or Patient-Physician Relation* or Patient-Health Care Professional Relation* or Patient-Health Professional Relation* or Patient-Health Professional Relation* or Patient-Healthcare Professional Relation* or Patient-Professional Relation* or Professional-Patient Relation* or Interpersonal or Patient Discharge Summar* or Patient Discharge Letter* (Abstract) Editions: WOS.SCI Date Run: Sun Aug 18 2024 16:16:01 GMT+0100 (British Summer Time) Results: 3442984

2: Prognos* or Predict* or Forecast* or Life Expectanc* or Disease Progress* or Disease Exacerbat* or Deteriorat* (Title) OR Prognos* or Predict* or Forecast* or Life Expectanc* or Disease Progress* or Disease Exacerbat* or Deteriorat* (Abstract) Editions: WOS.SCI Date Run: Sun Aug 18 2024 16:16:28 GMT+0100 (British Summer Time) Results: 4892944

3: Uncertain* or Indetermin* or Ambigu* or Unpredictab* or Vague* or Doubt* (Title) OR Uncertain* or Indetermin* or Ambigu* or Unpredictab* or Vague* or Doubt* (Abstract) Editions: WOS.SCI Date Run: Sun Aug 18 2024 16:17:05 GMT+0100 (British Summer Time) Results: 792328

4: Palliat* or Hospice* or Bereav* or Terminal* or End-of-Life or Death* or Advanced Illness* or Advanced Disease* or Life-Limiting or Die or Dying or Bereav* (Title) OR Palliat* or Hospice* or Bereav* or Terminal* or End-of-Life or Death* or Advanced Illness* or Advanced Disease* or Life-Limiting or Die or Dying or Bereav* (Abstract) Editions: WOS.SCI Date Run: Sun Aug 18 2024 16:17:26 GMT+0100 (British Summer Time) Results: 2533135

5: #4 AND #3 AND #2 AND #1 Editions: WOS.SCI Date Run: Sun Aug 18 2024 16:17:46 GMT+0100 (British Summer Time) Results: 1077

Google Scholar

#1 allintitle:Communicating|Communication Prognosis|Prognostic Uncertainty 7

#2 allintitle:Communicating|Communication Uncertainty Palliative|Hospice|"End of Life"|"Life Limiting" 6

7 + 6 = 12 (+ 1 duplicate)

Trip Database

PICO Search

Population: palliative OR "end of life"

Intervention: communicating OR communication

Outcome: (prognosis OR prognostic) AND uncertainty

8 results

NICE Guidelines

(Communicating OR Communication) AND (Prognosis OR Prognostic) Uncertainty AND (Palliative OR Hospice OR "End of Life" OR "Life Limiting")

20

Marie Curie via Google

(Prognosis OR Prognostic) Uncertainty site:mariecurie.org.uk

110

NHS Digital via Google

(Prognosis OR Prognostic) Uncertainty site:digital.nhs.uk

For peer review only

6

NHS Improvement via Google

(Prognosis OR Prognostic) Uncertainty site:improvement.nhs.uk

0

Royal College of Physicians via Google

(Prognosis OR Prognostic) Uncertainty site:rcp.ac.uk

19

General Medical Council via Google

(Communicating OR Communication) (Prognosis OR Prognostic) Uncertainty (Palliative OR "End of Life") site:gmc-uk.org

146

Department of Health via Google

intitle:(Communicating OR Communication) (Prognosis OR Prognostic) Uncertainty (Palliative OR "End of Life") site:gov.uk

273

For peer review only

List of fields for data extraction:

1st Author

Year

Country

Title

Aim

Study design (primary data collection (qualitative, quantitative, mixed), literature review (systematic or narrative), policy document, guideline, thesis, book chapter other)

Health professionals, Patients, Carers? (describe if combination)

Patient population & diagnoses (details of study populatino)

Setting (inpatient, outpatient, community, primary care, details of specialty if given)

type of communication described(verbal, written, patient/carer-professional, interprofessional, mixed or multiple types)

communication intervention (describe if relevant)

number of participants (primary data collection only)

Rigour assessment score (high, moderate, low)

Relevance score (high, moderate, low)

Additional details (free text)

For peer review only

Appendix 5. Details of included articles.

1st Author	Year	Country	Aim	Study design	HCP, Patients or Carers?	Patient population & diagnoses	Setting	Rigour	Relevance
						e.g. stroke, multimorbidity, frailty,			
Marie Curie	2014	UK	gain a deeper insight into the needs of terminally ill people and their non-professional carers, and understand experiences across the journey from diagnosis to death	Primary data collection - qualitative	Patients and carers	General Palliative care	Community	Moderate	Moderate
Marie Curie (Anderson)	2020	UK	to find the communication techniques that worked best at building awareness, helping decision making and closing the conversation.	Other	HCP + Patients + Carers	General medicine	All or mixed settings	Low	High
NICE	2015	UK	to improve end of life care for people in their last days of life by communicating respectfully and involving them, and the people important to them, in decisions and by maintaining their comfort and dignity	Policy document/guidance	N/A	last days of life	All or mixed settings	Moderate	Moderate
NICE	2018	UK	to improve diagnosis and treatment to increase the length and quality of life for people with heart failure	Policy document/guidance	N/A	heart failure	All or mixed settings	Moderate	Low
Alwidyan	2023	UK	to investigate healthcare professionals' barriers to and enablers of deprescribing in older hospice patients at the end of life and prioritise relevant theoretical domains for behaviour change to be incorporated into future interventions to facilitate deprescribing	Primary data collection - qualitative	HCP only	General palliative care - older patients	Hospice	High	Low
Anderson	2020	UK	To understand the practices used to accomplish prognostication and other relevant activities in conversations/communication between HCPs and relatives/friends of patients who are imminently dying	Thesis	HCP + Carers	General palliative care	Hospice	High	Moderate
Anderson	2020	UK	To understand how clinicians and the relatives/friends of patients at the very end of life manage uncertainty and reference time in prognostic conversations	Primary data collection - qualitative	HCP + Carers	General palliative care	Hospice	High	High
Anderson	2021	UK	To examine transitions out of prognostic talk in interactions between clinicians and the relatives and friends of imminently dying hospice patients.	Primary data collection - qualitative	Other	General palliative care	Hospice	High	Low

Appendix 5. Details of included articles.

1st Author	Year	Country	Aim	Study design	HCP, Patients or Carers?	Patient population & diagnoses	Setting	Rigour	Relevance
Anstey	2012	UK	To explore the personal and professional experience of consultant cardiologists, oncologists and old age psychiatrists and the consequent impact on referral practices to specialist palliative care services	Primary data collection - qualitative	HCP only	Cardiology, Oncology, Old-Age Psychiatry	Secondary care	Moderate	Low
Bailey	2018	UK	To offer advice and support to doctors to develop and improve in confidence when discussing their patients' preferences and values as they approach end of life	Policy document/guidance	HCP + Patients	Mixed - general medicine and palliative care	All or mixed settings	Moderate	Moderate
Barclay	2011	UK	To review the literature concerning conversations about EOLC between patients with heart failure and healthcare professionals, with respect to the prevalence of conversations; patients' and practitioners' preferences for their timing and content; and the facilitators and blockers to conversations.	Literature review	HCP + Patients + Carers	Heart failure	All or mixed settings	High	High
Baum	2020	USA	To provide commentary on a study (Seow et al) regarding potential utility of a patient-oriented prognostic tool	Literature review	N/A	Cancer	All or mixed settings	Low	Low
Bayuo	2021	Ghana	To explore the end of life care experiences of burn care staff and ascertain how their experiences can facilitate the development of clinical guidelines.	Primary data collection - qualitative	HCP only	Severe burns	Secondary care	High	Low
Becker	2020	Switzerland	To discuss communication challenges in EOL decision making and outline strategies from an area of growing interest and research	Literature review	Other	General palliative care	Other	Low	High
BGS	2020	UK	to support clinicians and others to consider the needs of frail older people as they move towards the end of their lives and help them to provide high quality care	Policy document/guidance	N/A	Frailty	Other	Low	Moderate
Bloomer	2018	Australia	to explore how end-of-life care goals and decision-making are communicated in a geriatric inpatient rehabilitation setting.	Primary data collection - qualitative	HCP only	Geriatrics, frailty	Community	Moderate	Moderate
Bloomer	2019	Australia	To investigate end-of-life care provision for older people in subacute care.	Primary data collection - quantitative observational	HCP + Patients + Carers	Geriatrics	Other	Moderate	Low

For peer review only

Appendix 5. Details of included articles.<br>1st Author	Year	Country	Aim	Study design	HCP, Patients or Carers?	Patient population & diagnoses	Setting	Rigour	Relevance
Borbasi	2021	Australia	To describe the experience and perspectives of residential aged care managers on providing end of life care for residents living with dementia.	Primary data collection - qualitative	HCP only	Dementia	Care Home	High	Moderate
Bristowe	2015	UK	To examine the experience of care supported by the AMBER care bundle compared to standard care in the context of clinical uncertainty, deterioration and limited reversibility.	Primary data collection - qualitative	Patients and carers	General medicine	Secondary care	Moderate	High
Broom	2014	Australia	To explore a range of medical specialists' experiences of referral to palliative care.	Primary data collection - qualitative	HCP only	General Palliative care	Community	Moderate	Low
Bruun	2024 a	UK	To explore how hospice multidisciplinary team (MDT) members oriented to the delicacy of prognostication during their meetings.	Primary data collection - qualitative	HCP only	General palliative care	Hospice	High	Moderate
Bruun	2024 b	UK	To explore how temporality is conveyed in relation to patients' prognoses during hospice multidisciplinary team meetings.	Primary data collection - qualitative	HCP only	General palliative care	Hospice	High	High
Burgers	2022	Netherlands	To understand the challenges in daily clinical practice experienced by HCPs from different disciplines who provide palliative as well as general care to AYAs (adolescents and young adults) with a UPCP (uncertain and/or poor cancer prognosis).	Primary data collection - qualitative	HCP only	Young adults with advanced cancer	All or mixed settings	High	Moderate
Cagle	2016	USA	To respond to the need for a greater understanding of caregiver preferences and attitudes toward being included in discussions about life expectancy	Primary data collection - qualitative	Carers only	Adults with disability age 65+	Community	High	High
Carlini	2022	Australia	To synthesise and critically analyse the points of discordance and concordance between consumers and clinicians about quality end-of-life care.	Literature review	HCP + Patients + Carers	Older patients aged >60	All or mixed settings	Moderate	Low
Cauley	2016	USA	To describe how surgeons approach treatment decisions to avoid nonbeneficial surgery, or engage in preoperative conversations about end-of-life (EOL) care, and to identify modifiable factors to reduce nonbeneficial surgery near the EOL.	Primary data collection - qualitative	HCP only	Older patients with surgical emergencies	Secondary care	Moderate	Low

For peer review only

Appendix 5. Details of included articles.

1st Author	Year	Country	Aim	Study design	HCP, Patients or Carers?	Patient population & diagnoses	Setting	Rigour	Relevance
Cavanagh	2020	USA	We review the literature concerning patient, caregiver, and clinician perspectives on prognosis communication in heart failure	Literature review	HCP + Patients + Carers	Heart failure	Other	Moderate	Low
Chan	2011	Hong Kong	To explore the relationships among the patient's awareness of the prognosis, the family's awareness of the prognosis, the patient's anxiety, and difficulty in communicating with family members.	Primary data collection - quantitative observational	Patients and carers	General palliative care	Secondary care	Low	Moderate
Chang	2014	USA	To examine prognosis discussions held with hospitalized patients for whom palliative care consultations were requested, and if prognosis discussions did not occur, to explore why not.	Primary data collection - qualitative + quantitative	HCP only	General palliative care	Secondary care	Moderate	Moderate
Chen	2018	UK	To assess the adequacy of community-based services available in Northern Ireland (NI) and to meet the multidimensional needs of patients living with heart failure (HF), as experienced and perceived by general practitioners (GP).	Primary data collection - qualitative	HCP only	Advanced heart failure	Primary care	Moderate	Low
Chen	2022	USA	To explore patients' perceptions of and preferences for communication surrounding goals of palliative RT and cancer prognosis.	Primary data collection - qualitative	Patients only	Oncology - bone or lung metastases	Secondary care	High	Low
Chou	2017	USA	Describes oncologists' language during discussions of prognosis and treatment goals in clinical interactions with African American patients diagnosed with cancer.	Primary data collection - qualitative	HCP + Patients	African Americans with stage III or IV cancer	Secondary care	High	High
Chow	2018	Canada	To identify the main challenges to the access and delivery of palliative care in patients with advanced congestive heart failure, and to summarize recommendations for clinical practice based on the available literature.	Literature review	HCP + Patients + Carers	Advanced heart failure	N/A	Moderate	Moderate
Christakis	1999	USA	To understand how and why physicians do and do not prognosticate	Literature review	HCP + Patients	Mixed	N/A	Moderate	Moderate

For peer review only

Appendix 5. Details of included articles. 1st Author	Year	Country	Aim	Study design	HCP, Patients or Carers?	Patient population & diagnoses	Setting	Rigour	Relevance
Christakis	1995	USA	To address the questions: what is the role of prognosis in medical thought and practice? Why is prognosis not as explicit a part of medicine as diagnosis and therapy?	Thesis	HCP + Patients + Carers	Mixed	Other	High	High
Chu	2019	UK	Narrative review discussing prognostication in Palliative Care	Literature review	HCP + Patients + Carers	General palliative care	All or mixed settings	Low	High
Chu	2020	UK	To evaluate the recent literature on prognostication for patients with advanced cancer and to suggest ways in which physicians can improve their own clinical practice in formulating and communicating prognostic estimates.	Literature review	HCP + Patients + Carers	Advanced cancer	All or mixed settings	Low	High
Clarke	2018	UK	To investigate how patients and their family members make decisions about their future care as their condition deteriorates, with a particular focus on mealtimes, eating and drinking	Primary data collection - qualitative	HCP + Patients + Carers	Progressive neurological disease	Community	High	Moderate
Cohen	2016	USA	To assess the effectiveness of an educational quality improvement intervention designed to enhance fellows' communication with patients who have advanced kidney disease.	Primary data collection - qualitative + quantitative	HCP only	Advanced kidney disease	Secondary care	Moderate	Moderate
Combes	2021	UK	To develop and refine an intervention to increase advance care planning engagement with older people living with frailty.	Thesis	HCP + Patients	Older people with frailty	Community	High	Low
Connolly	2021	USA	to conduct a qualitative metasynthesis to answer the following research question:What are the post-stroke PC/EOL experiences from the perspectives of patients, their families and healthcare professionals (HCPs)	Literature review	HCP + Patients + Carers	Stroke	All or mixed settings	High	Moderate
Cormican	2022	Ireland	To synthesize qualitative research evidence on the experiences of informal carers caring for a patient with a chronic haematological malignancy	Literature review	Carers only	Chronic haematological malignancy	All or mixed settings	High	Low
Cottrell	2020	UK	To develop and refine an initial theory on engagement in ACP for people with MS and to identify ways to improve its uptake for those who desire it.	Literature review	HCP + Patients + Carers	Multiple Sclerosis	All or mixed settings	High	Low

For peer review only

Appendix 5. Details of included articles.

1st Author	Year	Country	Aim	Study design	HCP, Patients or Carers?	Patient population & diagnoses	Setting	Rigour	Relevance
Creutzfeldt	2016	USA	To present current knowledge and recommendations regarding communication tasks and practice approaches for neurologists as they practice primary palliative care, including discussing serious news, managing symptoms, aligning treatment with patient preferences, introducing hospice/terminal care, and using the multiprofessional approach.	Literature review	HCP + Patients + Carers	Neurology	N/A	Low	Moderate
Crowther	2011	UK	To explore needs of people with dementia and their informal carers in the last year of life and surrounding death	Thesis	HCP + Patients + Carers	Dementia	Community	High	Moderate
Dias	2022	Portugal	To explore physicians' barriers in performing advanced care planning in patients with cancer; to propose a guide for discussion of advanced care planning for Brazilian physicians	Thesis	HCP + Patients	Oncology	Secondary care	High	Low
Dickerson	2024	USA	To characterize surgeon communication of pancreatic cancer prognosis using VitalTalk's ADAPT framework	Primary data collection - qualitative	HCP + Patients	Oncology - pancreatic cancer	Secondary care	Moderate	Moderate
Diggle	2022	UK	To present and synthesise the current evidence around bereaved caregivers' experiences of end of life care for people with heart failure	Literature review	Carers only	Heart failure	All or mixed settings	High	Low
Disler	2022	Australia, New Zealand	To explore attitudes and perceived barriers to ACP in self-identified palliative care nurses engaged and interested in care for patients with COPD.	Primary data collection - qualitative + quantitative	HCP only	COPD	All or mixed settings	Moderate	Low
Downer	2022	USA	Narrative review discussing communication about time-limited trials in the intensive care unit	Literature review	N/A	Intensive Care	Secondary care	Low	Moderate
Ekberg	2021	UK	Informs evidence-based communication about illness progression and end of life through a rapid review of studies that directly observe how experienced clinicians manage such discussions.	Literature review	HCP + Patients + Carers	General palliative care	All or mixed settings	Moderate	High

For peer review only

Appendix 5. Details of included articles.<br>1st Author	Year	Country	Aim	Study design	HCP, Patients or Carers?	Patient population & diagnoses	Setting	Rigour	Relevance
Epstein	2021	USA	Explore typical reactions to epistemic and complex uncertainty and offer preliminary proposals for how mindfulness and shared mind might help clinicians and patients navigate more effectively towards clear, health-promoting and values-concordant responses.	Literature review	HCP + Patients + Carers	Serious illness	All or mixed settings	Low	High
Etkind	2016	UK	To examine in detail the concept of uncertainty in illness and its impact on patients and professionals; to appraise current practice and evidence in addressing uncertainty in life-limiting illness	Literature review	HCP + Patients + Carers	Life-limiting illness	All or mixed settings	Moderate	High
Etkind	2025	UK	To co-design clinical practice recommendations for communication of prognostic uncertainty in older people with advanced multimorbidity, based on the lived experience and expertise of patients, carers, and healthcare professionals.	Primary data collection - qualitative	HCP + Patients + Carers	Advanced multimorbidity	Other	High	High
Evans	2009	USA	To understand surrogate decision makers' views regarding whether physicians should discuss prognosis in the face of uncertainty.	Primary data collection - qualitative	Carers only	Intensive Care	Secondary care	High	High
Fallowfield	2002	UK	Narrative review discussing communication of truth vs deceit in palliative care	Literature review	HCP + Patients + Carers	General palliative care	All or mixed settings	Low	Moderate
Fenton	2023	USA	To investigate cancer caregivers' communication experiences and potential impact on patient and caregiver outcomes.	Primary data collection - qualitative	Carers only	Advanced cancer	Secondary care	High	Moderate
Fisher	2012	Australia	Narrative review discussing uncertainty in end-of-life care and shared decision making	Literature review	HCP + Patients + Carers	Intensive Care	Secondary care	Low	High
Fitzsimons	2019	UK and Ireland	To explore caregivers" experience when caring for a loved one with advanced heart failure at the end of life and to identify any unmet psychosocial needs.	Primary data collection - qualitative	Carers only	Heart failure	Secondary care	High	Low
Formagini	2022	USA	To explore key factors that affect the strength of the stigma around palliative care referrals and its association with end of life in cancer care.	Primary data collection - qualitative	HCP + Patients + Carers	Oncology	Secondary care	Moderate	Low

Appendix 5. Details of included articles.

1st Author	Year	Country	Aim	Study design	HCP, Patients or Carers?	Patient population & diagnoses	Setting	Rigour	Relevance
Gadoud	2013	UK	To explore aspects of a palliative care approach for people with advanced HF: recognition of need, transitions in care and impact on patients, family carers and clinicians	Thesis	HCP + Patients + Carers	Heart failure	All or mixed settings	High	Moderate
Gallagher	2015	Brazil, England, Germany, Ireland, Palestine	To understand nurses' EoL decision-making practices in ICUs in different cultural contexts	Primary data collection - qualitative	HCP only	Intensive Care	Secondary care	High	Low
Garland	2013	Canada	To review the existing literature to identify barriers that inhibit end-of-life communication in the heart failure population.	Literature review	HCP + Patients + Carers	Heart failure	All or mixed settings	High	Moderate
Geerse	2019	USA	To characterize the content of serious illness conversations and identify opportunities for improvement	Primary data collection - qualitative	HCP + Patients	Oncology	Secondary care	High	Moderate
Gerlach	2019	Germany	To explore experiences, views and perceptions of haemato-oncologists on the use of the 'Surprise'-Question in the haemato-oncology outpatients clinics of a university hospital in Germany.	Primary data collection - qualitative	HCP only	Haematological malignancy	Secondary care	Moderate	Moderate
Gofton	2018	Canada	To develop a conceptual understanding of the specific characteristics of palliative care in neurology and the challenges of providing palliative care in the setting of neurological illness.	Primary data collection - qualitative	HCP + Patients + Carers	Neurological disease	Secondary care	Moderate	Low
Gonella	2022	Italy	To investigate how end-of-life communication may contribute to palliative-oriented care at the end-of-life in nursing homes according to both families' and nurses' perspective. Secondly, to identify the contextual factors internal to the nursing home that may influence the timing and quality of communication. Thirdly, to confirm the foundations for a first theory of end-of-life communication	Primary data collection - qualitative	HCP + Carers	Palliative care in care homes	Care Home	Moderate	Low
Goodlin	2008	USA	Narrative review applying the current knowledge about discussing prognosis and participatory decision-making to heart failure care.	Literature review	HCP + Patients + Carers	Heart failure	All or mixed settings	Low	High

For peer review only

Appendix 5. Details of included articles.

1st Author	Year	Country	Aim	Study design	HCP, Patients or Carers?	Patient population & diagnoses	Setting	Rigour	Relevance
Goss	2023	USA	To better understand surrogate experiences and needs during the period of active decision-making in SABI (severe acute brain injury), to inform interventions to support SABI patients and families and improve clinician-surrogate communication.	Primary data collection - qualitative	Carers only	Severe acute brain injury	Secondary care	High	High
Gough	2019	UK	To explore patient-centred accounts of the value and timing of prognostic discussions in advanced soft tissue sarcoma	Primary data collection - qualitative	Patients only	Advanced soft tissue sarcoma	Secondary care	Moderate	Moderate
Gramling	2013	USA	To identify and describe major types of prognosis conversations that occur in the natural setting of palliative care consultation.	Primary data collection - qualitative	HCP + Patients + Carers	General palliative care	Secondary care	High	Low
Gramling	2013 a	USA	To describe prognosis communication during PC consultation with seriously ill hospitalized patients.	Primary data collection - qualitative + quantitative	HCP + Patients + Carers	seriously hospitalised patients	Secondary care	Moderate	Moderate
Gray	2021	USA	To describe the current literature on illness and prognostic understanding in patients with haematologic malignancies, to examine the insights gained from literature about illness and prognostic understanding in patients with solid tumors, and to discuss future directions to inform our approach to studying illness and prognostic understanding in haematologic malignancies.	Literature review	HCP + Patients + Carers	Haematological malignancy	All or mixed settings	Moderate	Moderate
Green	2011	UK	To explore the perspectives of health care professionals on the transition of heart failure patients to a palliative care approach.	Primary data collection - qualitative	HCP only	Heart failure	All or mixed settings	Moderate	Moderate
Gutierrez	2010	USA	To explore the communication practices of prognostic information at end of life between and among critical care nurses, physicians, and family members in ICU throughout patients' trajectories, and the social, institutional, professional, and political influences which shape these practices	Thesis	HCP + Patients + Carers	Intensive Care	Secondary care	High	High

For peer review only

Appendix 5. Details of included articles.<br>1st Author	Year	Country	Aim	Study design	HCP, Patients or Carers?	Patient population & diagnoses	Setting	Rigour	Relevance
Gutierrez	2012	USA	Explore prognostic communication in ICU settings	Primary data collection - qualitative	HCP + Carers	Intensive Care	Secondary care	High	Moderate
Gutierrez	2013	USA	To identify the timing of and influences on critical care physician communication of negative prognoses to family members in an ICU.	Primary data collection - qualitative	HCP + Carers	Intensive Care	Secondary care	High	Moderate
Habib	2019	USA	To characterise reported practices and predictors of prognostic discussions for a cohort of hematologic oncologists.	Primary data collection - quantitative observational	HCP only	Haematological malignancy	Secondary care	High	Moderate
Habib	2021	USA	To characterize whether prognostic uncertainty has increased and if tolerance of uncertainty is associated with prognostic discussion practices in thoracic oncology.	Primary data collection - quantitative observational	HCP only	Lung cancer	Secondary care	Moderate	High
Hagerty	2005	Australia	To clarify the current available knowledge of patient preferences, clinician views and current practice regarding the communication of prognosis.	Literature review	HCP + Patients	Cancer	All or mixed settings	Moderate	High
Halkett	2010	Australia	To understand patients' experiences of High Grade Glioma and identify and describe information and support needs along the disease trajectory.	Primary data collection - qualitative	Patients and carers	High grade glioma	Secondary care	High	Moderate
Hallen	2015	USA	To explore physicians' perceptions of the value of Clinician Prediction Models in EOL care.	Primary data collection - qualitative	HCP only	General medicine	Secondary care	Moderate	High
Han	2016 a	USA	Reflective piece discussing prognostic disclosure and uncertainty	Other	N/A	General palliative care	N/A	Low	Moderate
Han	2016 b	USA	To explore the effects of personalized numeric prognostic information on physicians' intentions to communicate prognosis to cancer patients at the end of life, and to identify factors that moderate these effects.	Primary data collection - quantitative observational	HCP only	End-stage cancer	Primary care	Moderate	Moderate

For peer review only

Appendix 5. Details of included articles. 1st Author	Year	Country	Aim	Study design	HCP, Patients or Carers?	Patient population & diagnoses	Setting	Rigour	Relevance
Hancock	2007	Australia	To conduct a systematic review of truth-telling in discussing prognosis with patients with progressive, advanced life-limiting illnesses and their caregivers.	Literature review	HCP + Patients + Carers	Advanced life-limiting illness	All or mixed settings	High	Moderate
Harrison	2019	Canada	To review how web-based prognosis tools for cancer patients and clinicians describe aleatory (risk estimates) and epistemic (imprecision in risk estimates) uncertainties.	Primary data collection - quantitative observational	N/A	Cancer	N/A	Moderate	Moderate
Harrison	2021	UK	To describe the palliative care needs and experiences of people with mesothelioma and their family carers.	Literature review	HCP + Patients + Carers	Mesothelioma	All or mixed settings	High	Low
Hendershot	2023	USA	To investigate the complexities and interplay between the concepts of prognostic uncertainty and patient preferences as they relate to the delivery of goal-concordant care to patients with severe acute brain injuries (SABI) in the (Neuro-ICU).Neurological Intensive Care Unit	Literature review	HCP + Patients + Carers	Severe acute brain injury	Secondary care	Low	High
Henselmans	2017	Netherlands	To examine how communication about life expectancy is initiated in consultations about palliative chemotherapy, and what prognostic information is presented.	Primary data collection - qualitative	HCP + Patients	Advanced cancer	Secondary care	High	High
Heyland	2009	Canada	To enrich our understanding of the relation between satisfaction with endoflife (EOL) care and discussions regarding the patient's prognosis.	Primary data collection - quantitative observational	Patients and carers	Advanced illness	Secondary care	Moderate	Low
Higginson	2013	UK	To develop and evaluate an interventional tool to improve communication and palliative care, using the ICU as an example of where this is difficult.	Other	HCP + Carers	Intensive Care Medicine	Primary care	High	Moderate
Hjelmfors	2014	Sweden	To describe HF nurses' perspectives on, and daily practice regarding, discussing prognosis and end-of-life care with HF patients in outpatient care. It further aims to explore barriers, facilitators and related factors for discussing these issues.	Primary data collection - quantitative observational	HCP only	Heart failure	All or mixed settings	Moderate	Moderate

Appendix 5. Details of included articles.<br>1st Author	Year	Country	Aim	Study design	HCP, Patients or Carers?	Patient population & diagnoses	Setting	Rigour	Relevance
Hjelmfors	2018	Sweden	To improve communication about the heart failure trajectory in patients, their families, and health care professionals	Thesis	HCP + Patients + Carers	Heart failure	All or mixed settings	High	Low
Hoff	2011	Sweden	To obtain a deeper understanding of how physicians are dealing with decision making and providing information to patients in situations where pertinent scientific knowledge is lacking or uncertain	Primary data collection - qualitative	HCP only	Oncology (Haematological and Lung)	Secondary care	Moderate	Moderate
Hoff	2014	Sweden	To identify inherent challenges in communicating information about imminent death.	Primary data collection - qualitative	HCP + Patients	Oncology (Haematological and Lung)	Secondary care	Moderate	High
Holloway	2013	USA	Narrative review discussing estimating and communicating prognosis in advanced neurologic disease	Literature review	HCP + Patients + Carers	Neurology	All or mixed settings	Low	High
Hudson	2017	UK	To explore the views and experiences of people who are homeless and those supporting them regarding conversations and approaches to palliative care.	Primary data collection - qualitative	HCP + Patients	General palliative care in patients experiencing homelessness	Community	High	Moderate
Hui	2019	USA	To provide an update on prognostication in patients with advanced cancer, and to discuss future directions for research in this field.	Literature review	HCP + Patients + Carers	Advanced cancer	All or mixed settings	Low	Moderate
Hui	2021	USA	Narrative review discussing the impact of prognostication on patient outcomes.	Literature review	HCP + Patients + Carers	Advanced cancer	All or mixed settings	Low	Moderate
Innes	2009	UK	To examine the research literature, to identify preferences for prognostic awareness in advanced cancer patients, to assess how much and what type of prognostic information cancer patients want in advanced disease and to identify any benefits that patients attribute to prognostic awareness.	Literature review	HCP + Patients + Carers	Advanced cancer	All or mixed settings	High	Moderate
Isaac	2017	USA	Editorial on discussing prognosis in the intensive care unit	Other	N/A	Intensive Care Medicine	Secondary care	Low	Moderate

For peer review only

Appendix 5. Details of included articles. 1st Author	Year	Country	Aim	Study design	HCP, Patients or Carers?	Patient population & diagnoses	Setting	Rigour	Relevance
Islam	2023	UK	To address the question: what are the barriers to and enablers of ethnically diverse patients, family caregivers and health-care professionals engaging in end-of-life care planning?	Primary data collection - qualitative	HCP + Patients + Carers	General palliative care	All or mixed settings	High	High
Jackson	2024	USA	Evidence supporting various communication strategies is presented, followed by a review of formal guidelines, when they exist	Literature review	N/A	Serious illness	All or mixed settings	Low	Moderate
Johnson	2012	USA	A case presentation of acute lung injury (ALI) progressing to acute respiratory distress syndrome (ARDS) requiring tracheal intubation and mechanical ventilation (ETMV) and narrative review discussing uncertainty in critical illness.	Literature review	HCP + Patients + Carers	Intensive Care Medicine	Secondary care	Low	High
Johnson	2015	UK	To explore the process of disclosure, patient experience and preferences for information regarding prognosis or risk of recurrence	Literature review	Patients only	Cancer	All or mixed settings	High	High
Jones	1983	USA	To determine the legal, professional, and ethical responsibilities of registered nurses as they make decisions about disclosure of prognostic information to dying adults	Thesis	HCP + Patients	Dying adults	All or mixed settings	Low	Low
Kantabanlan g	2023	UK	To provide an overview of current evidence on the experiences and palliative care needs of patients with hematologic malignancies and their families and the barriers and challenges of integrating palliative care into hematology care in Asia.	Literature review	HCP + Patients + Carers	Haematological malignancy	All or mixed settings	Low	Low
Kapo	2006	USA	Narrative review discussing prognosis in chronic diseases	Literature review	HCP + Patients + Carers	Chronic life-limiting illness	All or mixed settings	Low	Moderate
Kimbell	2014	UK	To broaden our understanding of the experience of living and dying with advanced liver disease, and to examine the utility of a qualitative longitudinal, multi-perspective methodology in end-of-life research.	Thesis	HCP + Patients + Carers	Advanced liver disease	All or mixed settings	High	High
Kimbell	2015	UK	To understand the experiences and support needs of people with advanced liver disease and those of their lay and professional carers to	Primary data collection - qualitative	HCP + Patients + Carers	Advanced liver disease	All or mixed settings	High	Moderate

For peer review only

Appendix 5. Details of included articles.

1st Author	Year	Country	Aim	Study design	HCP, Patients or Carers?	Patient population & diagnoses	Setting	Rigour	Relevance
			inform improvements in the supportive and palliative care of this rapidly growing but currently neglected patient group.						
Ko	2020	USA	To explore decision-making experiences, related to utilisation of hospice care programmes from a retrospective perspective, with family caregivers (FCGs) in a rural US–Mexico border region.	Primary data collection - qualitative	Carers only	General palliative care	Community	Moderate	Low
Krawczyk	2016	Canada	To report on the concept of "communicating prognostic uncertainty" which emerged from a mixed methods survey asking family members to rank their satisfaction in seven domains of hospital end-of-life care.	Primary data collection - qualitative	Carers only	General palliative care	All or mixed settings	Moderate	High
Kupeli	2019	UK	To understand the similarities and differences between healthcare professionals' and family carers' perspectives on optimal end-of-life care, gain insights into how the interdependent roles of family carers and HCPs can be optimised, and make recommendations for policy and practice.	Primary data collection - qualitative	Carers only	Advanced dementia	Care Home	Moderate	Moderate
Langmuir	2023	Canada	To review the literature on the experiences of uncertainty for individuals with advanced or metastatic cancer patients who are receiving immunotherapy or TT.	Literature review	Patients only	Advanced cancer	Secondary care	High	Moderate
LeBlanc	2018	USA	Narrative review discussing communicating prognosis in oncology	Literature review	N/A	Advanced cancer	N/A	Low	High
LeClaire	2005	USA	To determine whether the timing of prognostic information delivery by physicians is associated with caregiver satisfaction with communication or decision making in the ICU.	Primary data collection - quantitative observational	Carers only	Intensive Care	Secondary care	Moderate	Low
Lee	2022	Canada	Narrative review discussing uncertainty in surgical palliative care	Literature review	Other	Surgery	All or mixed settings	Low	Moderate
Low	2017	UK	To identify the limitations in palliative care provision in the last year of life for people with liver cirrhosis and potential barriers to and enablers of palliative care.	Primary data collection - qualitative	HCP + Patients	Advanced chronic liver disease	Secondary care	Moderate	Low
Maguire	1988	UK	Opinion article discussing handling uncertainty, collusion and denial when communicating with cancer patients	Other	N/A	Oncology	N/A	Low	Moderate

Appendix 5. Details of included articles.

1st Author	Year	Country	Aim	Study design	HCP, Patients or Carers?	Patient population & diagnoses	Setting	Rigour	Relevance
Mandel	2017	USA	To identify the barriers to serious illness conversations in the dialysis population, review best practices in and specific approaches to conducting serious illness conversations, and offer solutions to overcome barriers as well as practical advice, including specific language and tools, to implement serious illness conversations in the dialysis population.	Literature review	N/A	End-stage renal disease	All or mixed settings	Low	High
Masterson	2018	USA	To draw on the experiences of clinicians to clarify the concept of prognostic understanding and examine the mechanisms that contribute to patient understanding and uptake of prognostic information.	Primary data collection - qualitative	HCP only	Advanced cancer	Secondary care	Moderate	High
Maxwell	2020	USA	To examine hospitalized older adults' and family caregivers' receptivity to general (vs. individualized) prognostic information about frailty, injury, and one-year outcomes; and to determine information needs based on prognostic information.	Primary data collection - qualitative	Patients and carers	Frailty/older adults	Secondary care	Moderate	Low
McClung	2013	USA	To discuss specific clinical recommendations and an analysis of some of the ethical issues involved in the provision of care to elderly patients in the terminal stages of heart failure.	Literature review	HCP + Patients + Carers	Advanced heart failure	All or mixed settings	Moderate	Low
McDonald	2016	UK	Policy document on improving communication with patients in the National Health Service	Policy document/guidance	N/A	General palliative care	N/A	Moderate	Low
McLennon	2013	USA	To describe the frequency and types of ethical dilemmas experienced by oncology nurses caring for advanced cancer patients and to summarise their written comments about prognosis-related communications.	Primary data collection - qualitative	HCP only	Oncology	Secondary care	Moderate	Moderate
Medendorp	2021	Netherlands	To generate an overview of available recommendations on how to communicate uncertainty with patients during clinical encounters.	Literature review	N/A	General medicine	All or mixed settings	Moderate	High
Michael	2022	UK	This chapter covers the importance of cancer communication and strategies to aid practitioners in discussing recurrence, progression, prognostication, and transition to palliative care.	Book chapter	N/A	Advanced progressive disease	All or mixed settings	Low	Moderate

For peer review only

Appendix 5. Details of included articles.

1st Author	Year	Country	Aim	Study design	HCP, Patients or Carers?	Patient population & diagnoses	Setting	Rigour	Relevance
Moglia	2024	Italy	To investigate neurologists' attitudes toward prognostic communication and their knowledge and utilization of the ENCALS survival model in clinical practice.	Primary data collection - quantitative observational	HCP only	Amyotrophic Lateral Sclerosis	Secondary care	Moderate	High
Molzahn	2020	Canada	To describe how people diagnosed with heart failure and their family members describe uncertainty related to impending death.	Primary data collection - qualitative	Patients and carers	Advanced heart failure	Secondary care	High	High
Momen	2011	UK	To reviews the extent to which there is evidence that conversations with clinicians about end-of-life care occur for heart failure patients in practice.	Literature review	HCP + Patients	Heart failure	All or mixed settings	High	Moderate
Mone	2021	UK	To identify if there is concordance between actual prognosis and accurate prognostic awareness in individuals with advanced cancer, and to identify the rationale for any discordance between the two.	Literature review	N/A	Advanced cancer	N/A	High	Moderate
Mount	1980	Canada	Editorial discussing the psychological impact of urological cancer	Other	N/A	Oncology and general palliative care	Secondary care	Low	Moderate
Nadicksbernd	2023	UK	To explore professionals' perspectives of difficult conversations about life-limiting, deteriorating health and end-of-life.	Thesis	HCP only	General palliative care	Community	High	Moderate
Nagelschmidt	2020	Germany	To identify barriers that hinder or influence the discussion of end- of- life issues in the family context.	Literature review	Patients and carers	terminally ill adults	All or mixed settings	Moderate	Low
Narayan	2017	USA	To characterise heart failure patient preferences for, reactions to, and application of individualized SHFM prognostic information along with individualized SHFM (Seattle Heart Failure Model) prognostic information along with their understanding of uncertainty in population-based estimates.	Primary data collection - qualitative	Patients only	Heart failure	Secondary care	High	High
Ngwenya	2021	UK	To identify factors that impact communication and support and recommend ways to improve patients' understanding of living with life-threatening illness.	Primary data collection - qualitative	HCP + Patients + Carers	Lung cancer and COPD	Primary care	Moderate	High

For peer review only

1st Author	Year	Country	Aim	Study design	HCP, Patients or Carers?	Patient population & diagnoses	Setting	Rigour	Relevance
Noble	2017	UK	To explore clinician views on decisional conflict in patients with end-stage kidney disease.	Primary data collection - qualitative	HCP only	End-stage renal disease	Secondary care	Moderate	Moderate
Olsson	2021	Australia	To explore existing guidelines around palliative care to increase current understanding of end-of-life communication processes applicable to the acute care setting.	Literature review	Other	end of life	Secondary care	Moderate	Low
Owusuaa	2021	Netherla nds	To explore physicians' views on identifying and disclosing the last phase of life of patients with different illness trajectories.	Primary data collection - qualitative	HCP only	Cancer, COPD	All or mixed settings	Moderate	Moderate
Pardue	2020	USA	To apply Mishel's (1990) model of uncertainty in chronic illness to heart failure patients as a framework for clarifying the role uncertainty plays in end-of-life communication; to determine whether time perspectives play a role in heart failure patients' engagement in end-of-life communication; to elucidate the complex relationships between time perspective and uncertainty in illness and their association with end-of-life communication.	Primary data collection - quantitative observational	Patients only	Heart failure	All or mixed settings	Moderate	Moderate
Parker	2006	Australia	To conduct a systematic review regarding communication of prognosis and EoL issues with adults, and or their caregivers, with an advanced progressive life limiting illness.	Literature review	HCP + Patients + Carers	Advanced illness	All or mixed settings	High	High
Parker	2007	Australia	to review studies regarding prognostic/endof-life communication with adult patients in the advanced stages of a life-limiting illness and their caregivers	Literature review	HCP + Patients + Carers	Advanced illness	All or mixed settings	High	Moderate
Parry	2024	UK	To systematically collate, examine, and summarize what knowledge and insights have been established by Conversation Analysis research in palliative care and in other healthcare settings where there are interactions about the end of the patient's life	Literature review	N/A	General palliative care	N/A	Low	High
Parvez	2015	USA	To consider the concept of 'uncertainty' in clinical practice for end stage renal disease from physician and patient perspectives.	Literature review	HCP + Patients	End stage renal disease	All or mixed settings	Low	High

Table title (in header): **Appendix 5. Details of included articles.**

For peer review only

Appendix 5. Details of included articles.

1st Author	Year	Country	Aim	Study design	HCP, Patients or Carers?	Patient population & diagnoses	Setting	Rigour	Relevance
Payne	2010	UK	To identify patients' and family members' experiences of acute stroke and their preferences for end-of-life care.	Primary data collection - qualitative	Patients and carers	Acute stroke	Secondary care	Moderate	High
Peoples	2020	USA	To identify factors that influence how prognostic uncertainty is viewed by physicians, as it relates to their communications with families.	Primary data collection - qualitative	HCP only	General medicine/surgery	Secondary care	High	High
Perumalswami	2019	USA	To present an overview of the role of prognostic uncertainty in oncologists' emotions, why the landscape of prognostication for cancer is changing, and how certain medical advances that have the potential to induce a "Lazarus effect" for patients near death present daily ethical challenges for oncologists	Literature review	N/A	Oncology	Secondary care	Low	Moderate
Pfeil	2015	Germany	To explore the factors that hinder communication about the terminal nature of advance cancer, and to reconstruct how physicians and nurses in oncology perceive their roles in preparing patients for end-of-life (EOL) decisions.	Primary data collection - qualitative	HCP only	Advanced cancer	Secondary care	Moderate	Moderate
Piovesan	2024	Canada	To evaluate the usefulness, for patients and families, of knowing the prognosis during the last days of life and to identify its utility in planning the activities that are important to them during their remaining days.	Primary data collection - quantitative observational	Patients and carers	Hospice inpatients	Hospice	Moderate	Moderate
Pontin	2011	UK	To explore hospital specialist palliative care professionals' experience of prognostication.	Primary data collection - qualitative	HCP only	General palliative care	Secondary care	Moderate	High
Pooler	2018	Canada	To explore bereaved caregivers' experiences and perceptions of an early integrated palliative approach implemented at a Multidisciplinary Interstitial Lung Disease Clinic.	Primary data collection - qualitative	Carers only	Idiopathic Pulmonary Fibrosis	Secondary care	Moderate	Low
Powers	2011	USA	To illustrate distinctions and intersections of palliative care and end-of-life services through examples from case-centered data of older adults cared for during a four-year ethnographic study of an acute care hospital palliative care consultation service.	Primary data collection - qualitative	HCP + Patients + Carers	General palliative care	Secondary care	Low	Moderate

Appendix 5. Details of included articles.

1st Author	Year	Country	Aim	Study design	HCP, Patients or Carers?	Patient population & diagnoses	Setting	Rigour	Relevance
Pun	2023	Hong Kong	To synthesise empirical findings on the role of family in end- of- life (EOL) communication and to identify the communicative practices that are essential for EOL decision- making in family- oriented cultures.	Literature review	HCP + Patients + Carers	end of life care	All or mixed settings	Moderate	Moderate
Rafferty	2016	USA	To explore spouses' reasons for seeking and avoiding conversations regarding their partners' EOL care preferences, and examine whether certain demographic factors (eg, income, gender, age) more likely contributed to the initiation of EOL conversations.	Primary data collection - qualitative + quantitative	Carers only	End of life care	All or mixed settings	Moderate	Low
Reid	2015	UK	To explore healthcare professionals' views on delivering end-of-life care within an acute hospital trust	Primary data collection - qualitative	HCP only	General palliative care	Secondary care	Moderate	Moderate
Rhee	2013	Australia	To explore the perspectives of general practitioners (GPs) on factors influencing the implementation of ACPs.	Primary data collection - qualitative	HCP only	General practice	Primary care	High	Low
Richards	2013	UK	To explore if and how information about a transition to a palliative care approach was communicated to patients recently discharged from hospital and who fulfilled standardized criteria for palliative care need.	Primary data collection - qualitative	Patients and carers	General palliative care	Secondary care	Moderate	Moderate
Ridley	2013	Australia	Narrative review discussing uncertainty in end-of-life decision making	Literature review	N/A	Intensive care medicine	Secondary care	Low	High
Risco	2022	USA	To discuss the importance of prognostication and developing a prognostic mindset, as well as providing a framework for formulating prognosis and communicating "what to expect."	Literature review	N/A	Neurology	All or mixed settings	Low	High
Robinson	2021	New Zealand	To explore the impact of uncertainty on how death is remembered by bereaved family members of people with heart disease.	Primary data collection - qualitative	Carers only	Heart disease	All or mixed settings	Moderate	High
Rowe	2021	USA	To qualitatively explore the challenges presented by the COVID-19 pandemic from the perspective of palliative care clinicians.	Primary data collection - qualitative	HCP only	General palliative care	All or mixed settings	High	Low

For peer review only

Appendix 5. Details of included articles.<br>1st Author	Year	Country	Aim	Study design	HCP, Patients or Carers?	Patient population & diagnoses	Setting	Rigour	Relevance
Salins	2024	India	To address palliative care needs in the ICU by synthesising palliative care practices provided at end-of-life to critically ill patients and their families before, during, and after ICU admission.	Literature review	HCP + Patients + Carers	Intensive care	Secondary care	High	Moderate
Sanders	2023	Canada	To propose three methodological innovations to advance the basic science of serious illness communication.	Literature review	N/A	Serious illness	All or mixed settings	Low	Moderate
Scholz	2020	Australia	To synthesise clinicians' understandings of communication in end-of-life care.	Literature review	HCP only	Intensive care	All or mixed settings	Moderate	Low
Schuster	2012	USA	To evaluate whether and how physicians discuss prognostic uncertainty with surrogate decision makers of patients who are highly likely, but not certain, to die.	Primary data collection - qualitative + quantitative	HCP + Carers	Intensive care	Secondary care	Moderate	High
Schutz	2018	USA	Editorial discussing communication, personhood and uncertainty in neuropalliative care	Literature review	HCP + Carers	Neuropalliative care	N/A	Low	Low
Sedhom	2024	USA	Narrative review discussing person-centred supportive care in precision oncology	Literature review	HCP + Patients + Carers	Oncology	All or mixed settings	Low	High
Selman	2019	UK	To explore views and experiences of communication, information provision and treatment decision-making among older patients receiving conservative care.	Primary data collection - qualitative	Patients only	Older people with end-stage renal disease	Secondary care	High	Moderate
Sikhosana	2023	Australia	To synthesise the best available evidence on the experience of persons living with a life-limiting illness about expressing their voice during communication with healthcare professionals.	Literature review	Patients only	Life-limiting illness	All or mixed settings	High	Moderate
Simpkin	2019	USA	To provide a narrative review of studies relating to the communication of uncertainty, highlighting current strategies together with challenges and barriers, and outline a framework for future research.	Literature review	HCP + Patients + Carers	General	All or mixed settings	Low	High

For peer review only

Appendix 5. Details of included articles.

1st Author	Year	Country	Aim	Study design	HCP, Patients or Carers?	Patient population & diagnoses	Setting	Rigour	Relevance
Smith	2014		To examine ethical issues in disease prognosis and prognostication in palliative care from a clinical perspective.	Book chapter	N/A	General palliative care	All or mixed settings	Moderate	High
Stanek	2020	USA	(a) provide an analysis and clarification of the concept of goals of care, (b) identify the frequency, distribution, and domains of uncertainty expressed by patients with advanced cancer in their communication with the palliative care team using Han's taxonomy of uncertainty, and (c) describe the magnitude and precision of the association between types of expressed patient uncertainty and patients' self-rated prognosis.	Thesis	Patients only	Advanced cancer	Secondary care	High	High
Stiel	2019	Australia	To explore the experiences of and communication techniques used by palliative care physicians as they consider and discuss nursing home placements for their patients.	Primary data collection - qualitative	HCP only	General palliative care	All or mixed settings	High	Low
Stocker	2014	UK	To explore experiences of giving or receiving a prognosis and managing the transition point from diagnosis to palliative and end of life care for those with heart failure.	Thesis	HCP + Patients + Carers	Heart failure	All or mixed settings	High	High
Stocker	2017	UK	To explore experiences of giving or receiving a prognosis and advanced palliative care planning for those with heart failure.	Primary data collection - qualitative	HCP + Patients + Carers	Heart failure	Secondary care	High	Moderate
Sullivan	2007	USA	To describe whether and when physicians report recognizing and communicating the imminence of death and identify potential barriers and facilitators to recognition and communication about dying in the hospital.	Primary data collection - qualitative	HCP only	General/terminal illness	Secondary care	Moderate	Moderate
Szawarski	2016	UK	Editorial discussing uncertainty in critical illness	Literature review	N/A	Critical illness	N/A	Low	High
Tarbi	2021	USA	To describe the existential dimensions of prognosis communication in naturally-occurring palliative care conversations.	Primary data collection - qualitative + quantitative	HCP + Patients + Carers	Advanced cancer	Secondary care	High	Moderate

For peer review only

Appendix 5. Details of included articles.<br>1st Author	Year	Country	Aim	Study design	HCP, Patients or Carers?	Patient population & diagnoses	Setting	Rigour	Relevance
Tavares	2019	UK	To understand COPD patients' preferences for palliative care discussions; and explore clinicians' opinions and experiences when holding these conversations.	Thesis	HCP + Patients	COPD	All or mixed settings	High	Moderate
Tavares	2020	UK	To understand the preferences of patients with chronic obstructive pulmonary disease for discussions about palliative and advance care planning with clinicians.	Primary data collection - qualitative	Patients only	COPD	All or mixed settings	High	Low
Temel	2016	USA	Narrative review discussing prognostic uncertainty in the modern era of cancer therapeutics	Literature review	N/A	Oncology	All or mixed settings	Low	High
Tetteh	2021	USA	To examine openness and topic avoidance in interpersonal communication about ovarian cancer.	Primary data collection - qualitative	Patients only	Ovarian cancer	All or mixed settings	High	Moderate
Tilburgs	2018	Netherlands	To determine the barriers and facilitators faced by GPs related to ACP with people with dementia.	Literature review	HCP + Patients + Carers	Dementia	Primary care	High	Low
Tolsa	2022	Switzerland	To explore (1) the perspectives and experiences of clinicians regarding the use of predictive scores for LST decision making in severe acute stroke, and (2) clinicians' awareness of their own cognitive biases in this context.	Primary data collection - qualitative	HCP only	Severe acute stroke	Secondary care	High	Moderate
Tosh	2018	UK	Reflective article discussing conversations about death	Other	N/A	General/end-of-life	N/A	Low	Moderate
Trankle	2020	Australia	To complement our quantitative survey data by providing a greater understanding of the experiences and views of clinicians when engaging in EoL discussions.	Primary data collection - qualitative + quantitative	HCP only	General palliative care	All or mixed settings	High	Moderate
Tuck	2015	USA	To determine preferences of patients with Parkinson's Disease for timing and initiation of discussions regarding treatment, prognosis, advanced care planning, and end-of-life care options such as hospice.	Primary data collection - qualitative + quantitative	Patients only	Parkinson's Disease	Secondary care	High	Low

For peer review only

Appendix 5. Details of included articles. 1st Author	Year	Country	Aim	Study design	HCP, Patients or Carers?	Patient population & diagnoses	Setting	Rigour	Relevance
Udo	2018	Sweden	To explore palliative home care physicians' experiences regarding end-of-life breakpoint communication	Primary data collection - qualitative	HCP only	General palliative care	Community	High	Moderate
van der Velden	2020	Netherlands	To offer up-to-date, evidence-based guidance on prognostic communication in palliative oncology.	Literature review	Patients only	Oncology	All or mixed settings	Moderate	Moderate
van der Velden	2021	Netherlands	To explore (1) how advanced cancer patients phrase their questions and ideas about prognosis; (2) how consulting oncologists discuss prognosis with advanced cancer patients; and (3) how advanced cancer patients and consulting oncologists discuss prognostic information previously provided by the referring oncologist.	Primary data collection - qualitative	HCP + Patients	Advanced cancer	Secondary care	High	High
van der Velden	2023	Netherlands	(1) describe the extent and direction of prognostic discordance, patients' prognostic information preferences in cases of prognostic discordance, and physicians' awareness of prognostic discordance; and (2) examine which patient, physician, and caregiver factors predict prognostic discordance.	Primary data collection - quantitative observational	HCP + Patients + Carers	Advanced cancer	Secondary care	High	Moderate
van der Velden	2024	Netherlands	To investigate the effects of prognostic communication strategies (prognostic disclosure vs. communication of unpredictability vs. non-disclosure; standard vs.standard and best-case vs.standard, best- and worst-case survival scenarios; numerical vs.word-based estimates) on prognostic perceptions, treatment decision-making and end-of-life anticipation in advanced cancer.	Primary data collection - quantitative observational	Other	Advanced cancer	All or mixed settings	Moderate	Moderate
van der Velden	2024 b	Netherlands	To investigate effects of prognostic communication strategies on emotions, coping, and appreciation of consultations in advanced cancer.	Primary data collection - quantitative observational	Other	Advanced cancer	All or mixed settings	Moderate	Moderate
van Eenennaam	2020	Netherlands	To develop a communication guide to support neurologists and rehabilitation physicians in discussing personalized prognosis, tailored to the individual needs and preferences of people with ALS and their families.	Policy document/guidance	HCP + Patients + Carers	Amyotrophic Lateral Sclerosis	All or mixed settings	Moderate	High

Appendix 5. Details of included articles.<br>1st Author	Year	Country	Aim	Study design	HCP, Patients or Carers?	Patient population & diagnoses	Setting	Rigour	Relevance
Vlckova	2020	Czech Republic	To synthesise the available literature on factors associated with accurate prognostic awareness in patients with cancer.	Literature review	HCP + Patients	Oncology	All or mixed settings	High	Moderate
Walczak	2013	Australia, USA	To explore patients' perspectives across two cultures (Australia and USA) regarding communication about prognosis and end-of-life care issues and to consider the ways in which these discussions can be optimised.	Primary data collection - qualitative	Patients only	Oncology	Secondary care	High	High
Walczak	2016	Australia	To identify and synthesise evidence for interventions targeting end-of-life communication.	Literature review	HCP + Patients + Carers	End-of-life care	All or mixed settings	High	Low
Waldrop	2012	USA	Guided by concepts from the Sense of Coherence Theory, which illuminates factors that contribute to adaptation in stressful situations, the objective of this study was to explore and describe family caregivers' accounts of the nature and timing of communication they had with a loved one's health care provider(s) during the advanced stages of cancer and before hospice enrollment.	Primary data collection - qualitative	Carers only	Advanced cancer	Hospice	High	Moderate
Wijsenbeek	2022	Netherlands	Consider the difficulties inherent in discussions with patients with IPF and their loved ones, and how clinicians might communicate with patients more effectively, from breaking the news about the diagnosis to providing support throughout the course of the disease.	Literature review	HCP + Patients + Carers	Idiopathic Pulmonary Fibrosis	All or mixed settings	Low	High
Wittenberg	2009	USA	Narrative review discussing estimating prognosis in end-stage renal disease	Literature review	HCP + Patients + Carers	End-stage renal disease	Secondary care	Low	Moderate
Wittenberg-Lyles	2008	USA	To understand the use and effectiveness of current communication protocols in terminal prognosis disclosures.	Primary data collection - qualitative	HCP + Patients + Carers	General palliative care	Secondary care	Low	Low
Wu	2024	China	To synthesise conversation analytic studies examining communication between healthcare professionals (HCPs) and patients/companions in the setting of palliative care.	Literature review	HCP + Patients + Carers	General palliative care	All or mixed settings	Moderate	High

Appendix 5. Details of included articles.<br>1st Author	Year	Country	Aim	Study design	HCP, Patients or Carers?	Patient population & diagnoses	Setting	Rigour	Relevance
Xie	2023	Canada	Narrative review discussing Artificial Intelligence decision support tools for end-of-life care planning conversations	Literature review	HCP + Patients + Carers	End-of-life care	All or mixed settings	Moderate	Low
Young	2024	New Zealand	To synthesise literature on patients in the last months of life due to a cancer diagnosis who have declined a referral to end-of-life care.	Literature review	Patients and carers	Cancer	All or mixed settings	High	High
Ziegler	2015	Australia	Reflective article discussing risk and prognosis	Other	N/A		N/A	Low	Moderate

For peer review only

Appendix 6. Table of CMO constructs

	Context	Mechanism	Outcome
1	well trained, experienced clinician	Assess and establish patient understanding of condition	Good communication: individualised, timely, **sensitive**, respectful, consistent
2	well trained, experienced clinician	explicit/open communication of prognostic uncertainty	shared understanding of situation, sense of meaning, acceptance, facilitates care planning
3	clinician tolerates uncertainty	explicit/open communication of prognostic uncertainty	shared understanding of situation, sense of meaning, acceptance, facilitates care planning
4	patient/family/carer prompt prognostic discussion	Clinician engages proactively with uncertainty & their reactions to it	explicit/open communication of prognostic uncertainty
5	patient too unwell to communicate	honest/open communication of prognostic uncertainty with family	family satisfaction with end of life care
6	uncertain future illness trajectory	communication of a range of possible scenarios/repeated discussions	patient/family can prepare
7	patient or family unwilling to discuss uncertainty, prefer not to know	conversation not initiated	lack of understanding may lead to denial, distress, and even unresolved bereavement narratives for family, but can also enable patient to have hope and 'live in the moment'
8	strong care relationship, e.g. from relational continuity	collaboration/ working together to enable explicit/open/confident communication of prognostic uncertainty	shared understanding of situation, sense of meaning, acceptance, facilitates care planning
9	empathic care relationship	being available and appearing caring	patient and family feel supported despite uncertainty
10	differences in patient vs relative communication needs	degree to which communication is based on assessment of individual needs/preferences	differing communication needs are met
11	empathic care relationship	collaborative approach to understanding prognostic uncertainty	shared understanding of situation, acceptance, facilitates care planning

For peer review only

	Context	Mechanism	Outcome
12	poor previous communication	Acknowledgement of previous error, open acknowledgement of prognostic uncertainty	builds trust, avoids further conflict
13	complex illness situations	case conference with family	helps to explore and manage uncertainty, leaves room for hope, increases trust, supports decision making
14	treatment decision making in context of prognostic uncertainty	using the concept of 'practical certainty' i.e. 'we are as sure as we can be' during communication with patient and/or family	supports decision making whilst acknowledging inherent uncertainty
15	perceived expertise/authority of clinician delivering prognostic information	value that patient/family place on prognostic information	degree to which uncertainty is accepted/integrated
16	Uncertain prognosis in cancer care	having prognostic discussion with treating oncologist with whom patient has a prior relationship	greater prognostic awareness, hearing prognostic uncertainty from a senior clinician is perceived as more acceptable
17	baseline level of clinician expertise	clinician training/use of prognostic models	Clinician engages proactively with uncertainty & their reactions to it
18	knowledge of best practice but absence of training/experience/ strategies	clinicians anxious about holding conversations and avoid them	implicit/tacit/no communication of prognostic uncertainty
19	Acute illness with uncertain recovery, e.g. severe stroke	use prognostic tools as an aid + clinicians to recognise own biases regarding communicating prognostic uncertainty	improves decision making + clinician-patient/family relationship (especially where families prefer explicit communication of uncertainties)
20	inexperienced, poorly trained clinician	conversation not initiated with patient/family	family/informal caregivers may feel pressure to hold or communicate uncertainty themselves, leading to distress, disempowerment and regret
21	weak care relationship	implicit/tacit/no communication of prognostic uncertainty	lack of understanding, loss of trust, unresolved bereavement narratives

For peer review only

	Context	Mechanism	Outcome
22	patient or relative asks about prognosis	uncertainty may or may not be mentioned depending on clinician tolerance/experience/judgement	Variation in degree of understanding of prognostic uncertainty
23	emotional distress from uncertainty	moving from discussion of uncertainty to end on something where there is certainty	orients towards action and certainty, supports planning
24	Desire to shield patients	vague communication; Uncertainty avoided or un-acknowledged by clinicians	Patient and family do not understand prognostic uncertainty, may lead to greater distress
25	clinicians not confident of prognosis	do not engage in discussions about it	patients unaware what to expect, could cause anxiety or distress
26	Timing of discussion - delayed until near death	Clinician delays discussion until near to patient death to increase the certainty of prognostic information and avoid uncertainty	Family and patient informed late of prognosis/minimal time to process and plan due to clinician low tolerance of uncertainty
27	Prognostic uncertainty as barrier to discussion, often because of poor tolerance by clinician	Discussion avoided by healthcare professionals	Barrier to timely and appropriate ACP engagement
28	Uncertainty avoided or un-acknowledged by clinicians	Family left with responsibility to communicate	Family distress and potential poor health outcomes
29	clinician does not tolerate uncertainty	clinician delays conversation until prognostic uncertainty resolved	late communication of prognosis or prognostic uncertainty, low patient understanding, delayed decisions, however may be appropriate if information may be available soon
30	deteriorating patient	use of proxy constructs e.g. offering unscheduled hospital appointment to communicate short prognosis	lack of clarity can lead to misunderstandings, but may also foster hope
31	uncertainty presented as certainty by clinicians- positive framing	expected outcome does not occur	clinician feels distressed

For peer review only

	Context	Mechanism	Outcome
32	use of jargon/poor communication training etc	lack of clarity/prognostic uncertainty not communicated in a way that patients/families can understand	patient and family do not understand prognostic uncertainty
33	death is expected/in terminal phase	clinician introduces optimistic uncertainty to avoid giving bad news	false hope
34	advance care planning (ACP) discussion	Clinician concern that prognostic uncertainty means decision making is inappropriate	uncertainty is a barrier to ACP if using the 'decision making' model, but not if outlining future possibilities.
35	particularly unpredictable illness situation	challenging for clinicians to manage expectations in a changing situation	higher risk that patient and family do not understand uncertainty
36	finely balanced decision re aggressiveness of future care (e.g. in heart failure)	tacit/implicit/no communication of prognostic uncertainty	lack of understanding leading to aggressive care not in line with preferences.
37	different illnesses have different levels of uncertainty and reactions to it	tailor prognostic communication to nature of illness & trajectory	better prognostic awareness in illnesses that have more unpredictable trajectories
38	clinician does not tolerate uncertainty	uncertainty presented as certainty – positive or negative framing	false hope feelings of distress, hopelessness, abandonment
39	differences in patient vs relative communication needs	standardised communication	needs not met
40	no recognition of prognostic uncertainty	Communication frameworks and tools including AI nudge re potentially poor prognosis	increased likelihood of communication of prognosis when these tools are used
41	Unclear prognostic information and disclosure of uncertainty without explaining future possibilities	Patient perceives positive prognosis	Patient pursues unnecessarily aggressive treatments
42	ACP discussions	managing prognostic uncertainty by incorporating discussion of 'typical' illness trajectories	supports care planning in context of prognostic uncertainty
43	lack of time/resources, lack of access to clinicians	weak care relationship	implicit/tacit/no communication of prognostic uncertainty

For peer review only

	Context	Mechanism	Outcome
44	poor continuity of care	conflicting information from different providers	uncertainty (and associated distress) magnified, further discussions may be avoided for fear of introducing more conflicting information
45	variation in communication practices dependent on care environment/ culture (e.g. hospice vs hospital etc)	ability to make time for these discussions	explicit/open communication of prognostic uncertainty
46	socio-ethico-political norms and values	ability/willingness of patient/family to engage with prognostic information	level of understanding & ability to plan based on prognostic knowledge
47	patient/family sociocultural background	Communication not tailored to language/cultural preferences	patient and family do not understand prognostic uncertainty
48	cultural barriers to discussing uncertainty e.g. misconceptions that palliative care and dying are synonymous	clinicians less likely to raise uncertainty. focus on disease management rather than wider concerns	lack of communication/discussions delayed until uncertainty resolved
49	culture of avoiding explicit discussion of prognosis or uncertainty (high context communication)	family believe informing patient may harm their wellbeing	a) patient may have poor understanding of prognostic information, potentially causing anxiety and further uncertainty of situation b) cultural preferences are met
50	Patient/family spiritual/sociocultural background and views	Patient blocks or avoids discussion of prognosis and uncertainty "keeping it positive"	Discussion is delayed until patient is nearer end of life/ prognosis is more certain and short, resulting in more limited scope for care planning
51	Patient/family spiritual/sociocultural background and views	Family requests that patient is not given prognostic information within certain cultural contexts	Patient has no understanding or knowledge of prognosis - family may have been told separately
52	patient who lacks capacity to contribute to discussions e.g. advanced dementia	Degree of family awareness of patients wishes	Family awareness of patient wishes affects how easy it is to discuss prognosis or end of life care, which may affect understanding of uncertain illness trajectory

For peer review only

Appendix: detailed programme theory

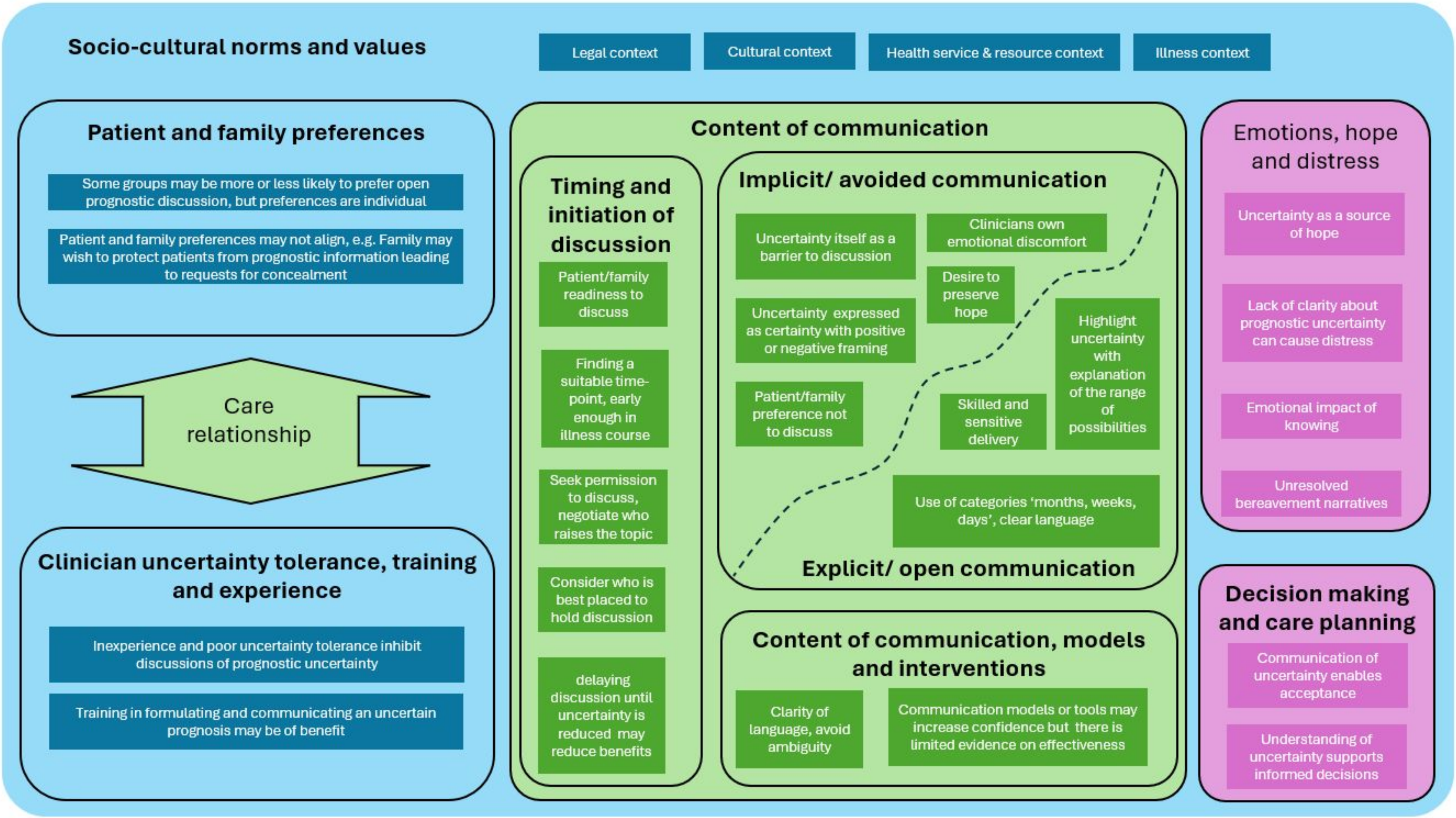

References for all included articles

1. Alwidyan T, McCorry NK, Parsons C. Healthcare professionals' perspectives of deprescribing in older patients at the end of life in hospice care: a qualitative study using the Theoretical Domains Framework. Int J Pharm Pract. 2023;31(3):305-13.
2. Anderson RJ. Communication with the Relatives and Friends of Imminently Dying Patients in a Hospice. England: University of London, University College London (United Kingdom); 2020.
3. Anderson RJ, Stone PC, Low JTS, Bloch S. Managing uncertainty and references to time in prognostic conversations with family members at the end of life: A conversation analytic study. Palliat Med. 2020;34(7):896-905.
4. Anderson RJ, Stone PC, Low JTS, Bloch S. Transitioning out of prognostic talk in discussions with families of hospice patients at the end of life: A conversation analytic study. Patient Educ Couns. 2021;104(5):1075-85.
5. Anstey S. Clinical thinking, knowledge and the referral practices of consultant physicians to specialist palliative care services: an interpretative phenomenological analysis. Wales: Cardiff University (United Kingdom); 2012.
6. Bailey SJ, Cogle K. Talking about dying: How to begin honest conversations about what lies ahead: Royal College of Physicians; 2018.
7. Barclay S, Momen N, Case-Upton S, Kuhn I, Smith E. End-of-life care conversations with heart failure patients: a systematic literature review and narrative synthesis. Br J Gen Pract. 2011;61(582):e49-62.
8. Baum LVM, Friedman D. The Uncertain Science of Predicting Death. JAMA Netw Open. 2020;3(4):e201736.
9. Bayuo J, Bristowe K, Harding R, Agbeko AE, Wong FKY, Agyei FB, et al. "Hanging in a balance": A qualitative study exploring clinicians' experiences of providing care at the end of life in the burn unit. Palliat Med. 2021;35(2):417-25.
10. Becker C, Beck K, Vincent A, Hunziker S. Communication challenges in end-of-life decisions. Swiss Med Wkly. 2020;150:w20351.
11. Bgs. End of Life Care in Frailty: Identification and prognostication. CLINICAL GUIDELINES. 2020.
12. Bloomer MJ, Botti M, Runacres F, Poon P, Barnfield J, Hutchinson AM. Communicating end-of-life care goals and decision-making among a multidisciplinary geriatric inpatient rehabilitation team: A qualitative descriptive study. Palliat Med. 2018;32(10):1615-23.
13. Bloomer MJ, Botti M, Runacres F, Poon P, Barnfield J, Hutchinson AM. End-of-life care for older people in subacute care: A retrospective clinical audit. Collegian. 2019;26(1):22-7.
14. Borbasi JAL, Tong A, Ritchie A, Poulos CJ, Clayton JM. "A good death but there was all this tension around"- perspectives of residential managers on the experience of delivering end of life care for people living with dementia. BMC Geriatr. 2021;21(1):306.
15. Bristowe K, Carey I, Hopper A, Shouls S, Prentice W, Caulkin R, et al. Patient and carer experiences of clinical uncertainty and deterioration, in the face of limited reversibility: A comparative observational study of the AMBER care bundle. Palliat Med. 2015;29(9):797-807.
16. Broom A, Kirby E, Good P, Wootton J, Adams J. The troubles of telling: managing communication about the end of life. Qual Health Res. 2014;24(2):151-62.

For peer review only

17. Bruun A, White N, Oostendorp L, Stone P, Bloch S. Time estimates in prognostic discussions: A conversation analytic study of hospice multidisciplinary team meetings. Palliat Med. 2024;38(5):593-601.
18. Bruun A, White N, Oostendorp L, Stone P, Bloch S. Prognostication As an Interactionally Delicate Matter: A Conversation Analytic Study of Hospice Multidisciplinary Team Meetings. Health Commun. 2025;40(6):956-64.
19. Burgers VWG, van den Bent MJ, Darlington AE, Gualtherie van Weezel AE, Compter A, Tromp JM, et al. A qualitative study on the challenges health care professionals face when caring for adolescents and young adults with an uncertain and/or poor cancer prognosis. ESMO Open. 2022;7(3):100476.
20. Cagle JG, McClymont KM, Thai JN, Smith AK. "If You Don't Know, All of a Sudden, They're Gone": Caregiver Perspectives About Prognostic Communication for Disabled Elderly Adults. J Am Geriatr Soc. 2016;64(6):1299-306.
21. Carlini J, Bahudin D, Michaleff ZA, Plunkett E, She EN, Clark J, et al. Discordance and concordance on perception of quality care at end of life between older patients, caregivers and clinicians: a scoping review. Eur Geriatr Med. 2022;13(1):87-99.
22. Cauley CE, Block SD, Koritsanszky LA, Gass JD, Frydman JL, Nurudeen SM, et al. Surgeons' Perspectives on Avoiding Nonbeneficial Treatments in Seriously Ill Older Patients with Surgical Emergencies: A Qualitative Study. J Palliat Med. 2016;19(5):529-37.
23. Cavanagh CE, Rosman L, Spatz ES, Fried T, Gandhi PU, Soucier RJ, et al. Dying to know: prognosis communication in heart failure. ESC Heart Fail. 2020;7(6):3452-63.
24. Chan WC. Being aware of the prognosis: how does it relate to palliative care patients' anxiety and communication difficulty with family members in the Hong Kong Chinese context? J Palliat Med. 2011;14(9):997-1003.
25. Chang A, Datta-Barua I, McLaughlin B, Daly B. A survey of prognosis discussions held by health-care providers who request palliative care consultation. Palliat Med. 2014;28(4):312-7.
26. Chen JJ, Gamble K, Graham-Wisener L, McGlade K, Doherty J, Donnelly P, et al. GP perceptions of the adequacy of community-based care for patients with advanced heart failure in a UK region (NI): a qualitative study. Open Heart. 2018;5(1):e000734.
27. Chen JJ, Roldan CS, Nichipor AN, Balboni TA, Krishnan MS, Revette AC, et al. Prognostic Understanding and Goals of Palliative Radiotherapy: A Qualitative Study. J Pain Symptom Manage. 2022;64(6):567-76.
28. Chou WS, Hamel LM, Thai CL, Debono D, Chapman RA, Albrecht TL, et al. Discussing prognosis and treatment goals with patients with advanced cancer: A qualitative analysis of oncologists' language. Health Expect. 2017;20(5):1073-80.
29. Chow J, Senderovich H. It's Time to Talk: Challenges in Providing Integrated Palliative Care in Advanced Congestive Heart Failure. A Narrative Review. Curr Cardiol Rev. 2018;14(2):128-37.
30. Christakis NA. Prognostication and bioethics. Daedalus. 1999;128(4):197-214.
31. Christakis NA, Fox RC. Prognostication and death in medical thought and practice. United States -- Pennsylvania: University of Pennsylvania; 1995.
32. Chu C, Anderson R, White N, Stone P. Prognosticating for Adult Patients With Advanced Incurable Cancer: a Needed Oncologist Skill. Curr Treat Options Oncol. 2020;21(1):5.

33. Chu C, White N, Stone P. Prognostication in palliative care. Clin Med (Lond). 2019;19(4):306-10.
34. Clarke G, Fistein E, Holland A, Tobin J, Barclay S, Barclay S. Planning for an uncertain future in progressive neurological disease: a qualitative study of patient and family decision-making with a focus on eating and drinking. BMC Neurol. 2018;18(1):115.
35. Cohen RA, Jackson VA, Norwich D, Schell JO, Schaefer K, Ship AN, et al. A Nephrology Fellows' Communication Skills Course: An Educational Quality Improvement Report. Am J Kidney Dis. 2016;68(2):203-11.
36. Combes S. Conversations on Living and Dying: Facilitating Advance Care Planning with Older People Living with Frailty. Developing and Refining an Intervention. England: University of London, King's College (United Kingdom); 2021.
37. Connolly T, Coats H, DeSanto K, Jones J. The experience of uncertainty for patients, families and healthcare providers in post-stroke palliative and end-of-life care: a qualitative meta-synthesis. Age Ageing. 2021;50(2):534-45.
38. Cormican O, Dowling M. Providing Care to People Living with a Chronic Hematological Malignancy: A Qualitative Evidence Synthesis of Informal Carers' Experiences. Semin Oncol Nurs. 2022;38(6):151338.
39. Cottrell L, Economos G, Evans C, Silber E, Burman R, Nicholas R, et al. A realist review of advance care planning for people with multiple sclerosis and their families. PLoS One. 2020;15(10):e0240815.
40. Creutzfeldt CJ, Robinson MT, Holloway RG. Neurologists as primary palliative care providers: Communication and practice approaches. Neurol Clin Pract. 2016;6(1):40-8.
41. Crowther JL. Dementia: What Care Do Patients and Informal Carers Need in the Last Year of Life and Time Surrounding Death? An Exploratory Qualitative Study. England: The University of Liverpool (United Kingdom); 2011.
42. Dias LM, Barra W, Rego F. Advance Care Planning in Oncology: Challenges in Clinical Practice. Portugal: Universidade do Porto (Portugal); 2022.
43. Dickerson LK, Lipson TA, Chauhan SSB, Allen GI, Young B, Park JO, et al. Evaluating surgeon communication of pancreatic cancer prognosis using the VitalTalk ADAPT framework. J Surg Oncol. 2024;130(3):476-84.
44. Diggle MFJ, Schutz S, Butcher D. Bereaved Caregivers' Experiences of End of Life Care For People With Advanced Heart Failure: A Narrative Synthesis. Omega (Westport). 2025;90(4):1581-608.
45. Disler R, Pascoe A, Luckett T, Donesky D, Irving L, Currow DC, et al. Barriers to Palliative Care Referral and Advance Care Planning (ACP) for Patients With COPD: A Cross-Sectional Survey of Palliative Care Nurses. Am J Hosp Palliat Care. 2022;39(2):169-77.
46. Downer K, Gustin J, Lincoln T, Goodman L, Barnett MD. Communicating About Time-Limited Trials. Chest. 2022;161(1):202-7.
47. Ekberg S, Parry R, Land V, Ekberg K, Pino M, Antaki C, et al. Communicating with patients and families about illness progression and end of life: a review of studies using direct observation of clinical practice. BMC Palliat Care. 2021;20(1):186.
48. Epstein RM. Facing epistemic and complex uncertainty in serious illness: The role of mindfulness and shared mind. Patient Educ Couns. 2021;104(11):2635-42.
49. Etkind SN, Fennessy R, Wang J, Simpson RM, O'Neill B, Barclay SIG, et al. Communicating prognostic uncertainties in advanced multimorbidity: a multimethod qualitative study to co-design practice recommendations. Eur Geriatr Med. 2025.

For peer review only

50. Etkind SN, Koffman J. Approaches to managing uncertainty in people with life-limiting conditions: role of communication and palliative care. Postgrad Med J. 2016;92(1089):412-7.
51. Evans LR, Boyd EA, Malvar G, Apatira L, Luce JM, Lo B, et al. Surrogate decision-makers' perspectives on discussing prognosis in the face of uncertainty. Am J Respir Crit Care Med. 2009;179(1):48-53.
52. Fallowfield LJ, Jenkins VA, Beveridge HA. Truth may hurt but deceit hurts more: communication in palliative care. Palliat Med. 2002;16(4):297-303.
53. Fenton A, Fletcher KM, Kizildag D, Borstelmann NA, Kessler D, Cronin C, et al. Cancer Caregivers' Prognostic and End-of-Life Communication Needs and Experiences and their Impact. J Pain Symptom Manage. 2023;65(1):16-25.
54. Fisher M, Ridley S. Uncertainty in end-of-life care and shared decision making. Critical care and resuscitation : journal of the Australasian Academy of Critical Care Medicine. 2012;14(1):81-7.
55. Fitzsimons D, Doherty LC, Murphy M, Dixon L, Donnelly P, McDonald K, et al. Inadequate Communication Exacerbates the Support Needs of Current and Bereaved Caregivers in Advanced Heart Failure and Impedes Shared Decision-making. J Cardiovasc Nurs. 2019;34(1):11-9.
56. Formagini T, Poague C, O'Neal A, Brooks JV. "When I Heard the Word Palliative": Obscuring and Clarifying Factors Affecting the Stigma Around Palliative Care Referral in Oncology. JCO Oncol Pract. 2022;18(1):e72-e9.
57. Gadoud AC. A palliative care approach for people with advanced heart failure: recognition of need, transitions in care and impact on patients, family carers and clinicians. England: University of Hull (United Kingdom); 2013.
58. Garland EL, Bruce A, Stajduhar K. Exposing barriers to end-of-life communication in heart failure: an integrative review. Canadian journal of cardiovascular nursing = Journal canadien en soins infirmiers cardio-vasculaires. 2013;23(1):12-8.
59. Geerse OP, Lamas DJ, Sanders JJ, Paladino J, Kavanagh J, Henrich NJ, et al. A Qualitative Study of Serious Illness Conversations in Patients with Advanced Cancer. J Palliat Med. 2019;22(7):773-81.
60. Gerlach C, Goebel S, Weber S, Weber M, Sleeman KE. Space for intuition - the 'Surprise'-Question in haemato-oncology: Qualitative analysis of experiences and perceptions of haemato-oncologists. Palliat Med. 2019;33(5):531-40.
61. Gofton TE, Chum M, Schulz V, Gofton BT, Sarpal A, Watling C. Challenges facing palliative neurology practice: A qualitative analysis. J Neurol Sci. 2018;385:225-31.
62. Gonella S, Basso I, Dimonte V, Giulio PD. The role of end-of-life communication in contributing to palliative-oriented care at the end-of-life in nursing home. Int J Palliat Nurs. 2022;28(1):16-26.
63. Goodlin SJ, Quill TE, Arnold RM. Communication and decision-making about prognosis in heart failure care. J Card Fail. 2008;14(2):106-13.
64. Goss AL, Voumard RR, Engelberg RA, Curtis JR, Creutzfeldt CJ. Do They Have a Choice? Surrogate Decision-Making After Severe Acute Brain Injury. Crit Care Med. 2023;51(7):924-35.
65. Gough N, Ross JR, Riley J, Judson I, Koffman J. 'When something is this rare ... how do you know bad really is bad...?'-views on prognostic discussions from patients with advanced soft tissue sarcoma. BMJ Support Palliat Care. 2019;9(1):100-7.

For peer review only

66. Gramling R, Norton S, Ladwig S, Winters P, Metzger M, Quill T, et al. Latent classes of prognosis conversations in palliative care: a mixed-methods study. J Palliat Med. 2013;16(6):653-60.
67. Gramling R, Norton SA, Ladwig S, Metzger M, DeLuca J, Gramling D, et al. Direct observation of prognosis communication in palliative care: a descriptive study. J Pain Symptom Manage. 2013;45(2):202-12.
68. Gray TF, Temel JS, El-Jawahri A. Illness and prognostic understanding in patients with hematologic malignancies. Blood Rev. 2021;45:100692.
69. Green E, Gardiner C, Gott M, Ingleton C. Exploring the extent of communication surrounding transitions to palliative care in heart failure: the perspectives of health care professionals. Journal of palliative care. 2011;27(2):107-16.
70. Gutierrez KM. Communication of prognostic information in an ICU at end of life: practices among and between nurses, physicians and family members: University of Minnesota; 2010.
71. Gutierrez KM. Prognostic communication of critical care nurses and physicians at end of life. Dimens Crit Care Nurs. 2012;31(3):170-82.
72. Gutierrez KM. Prognostic categories and timing of negative prognostic communication from critical care physicians to family members at end-of-life in an intensive care unit. Nurs Inq. 2013;20(3):232-44.
73. Habib AR, Chen R, Magnavita ES, Jaung T, Awad MM, Odejide O, et al. Prevalence and Tolerance of Prognostic Uncertainty Among Thoracic Oncologists. Oncologist. 2021;26(8):e1480-e2.
74. Habib AR, Cronin AM, Earle CC, Tulsky JA, Mack JW, Abel GA, et al. How Do Blood Cancer Doctors Discuss Prognosis? Findings from a National Survey of Hematologic Oncologists. J Palliat Med. 2019;22(6):677-84.
75. Hagerty RG, Butow PN, Ellis PM, Dimitry S, Tattersall MH. Communicating prognosis in cancer care: a systematic review of the literature. Ann Oncol. 2005;16(7):1005-53.
76. Halkett GK, Lobb EA, Oldham L, Nowak AK. The information and support needs of patients diagnosed with High Grade Glioma. Patient Educ Couns. 2010;79(1):112-9.
77. Hallen SA, Hootsmans NA, Blaisdell L, Gutheil CM, Han PK. Physicians' perceptions of the value of prognostic models: the benefits and risks of prognostic confidence. Health Expect. 2015;18(6):2266-77.
78. Han PK. The Need for Uncertainty: A Case for Prognostic Silence. Perspect Biol Med. 2016;59(4):567-75.
79. Han PK, Dieckmann NF, Holt C, Gutheil C, Peters E. Factors Affecting Physicians' Intentions to Communicate Personalized Prognostic Information to Cancer Patients at the End of Life: An Experimental Vignette Study. Med Decis Making. 2016;36(6):703-13.
80. Hancock K, Clayton JM, Parker SM, Wal der S, Butow PN, Carrick S, et al. Truth-telling in discussing prognosis in advanced life-limiting illnesses: a systematic review. Palliat Med. 2007;21(6):507-17.
81. Harrison M, Gardiner C, Taylor B, Ejegi-Memeh S, Darlison L. Understanding the palliative care needs and experiences of people with mesothelioma and their family carers: An integrative systematic review. Palliat Med. 2021;35(6):1039-51.
82. Harrison M, Han PKJ, Rabin B, Bell M, Kay H, Spooner L, et al. Communicating uncertainty in cancer prognosis: a review of web-based prognostic tools. Patient education and counseling. 2019;102(5):842-9.

For peer review only

83. Hendershot KA, Elias MN, Taylor BL, Wahlster S, Creutzfeldt CJ. An Update on Palliative Care in Neurocritical Care: Providing Goal-Concordant Care in the Face of Prognostic Uncertainty. Curr Treat Options Neurol. 2023;25(12):517-29.
84. Henselmans I, Smets EMA, Han PKJ, de Haes H, Laarhoven H. How long do I have? Observational study on communication about life expectancy with advanced cancer patients. Patient Educ Couns. 2017;100(10):1820-7.
85. Heyland DK, Allan DE, Rocker G, Dodek P, Pichora D, Gafni A. Discussing prognosis with patients and their families near the end of life: impact on satisfaction with end-of-life care. Open Med. 2009;3(2):e101-10.
86. Higginson IJ, Koffman J, Hopkins P, Prentice W, Burman R, Leonard S, et al. Development and evaluation of the feasibility and effects on staff, patients, and families of a new tool, the Psychosocial Assessment and Communication Evaluation (PACE), to improve communication and palliative care in intensive care and during clinical uncertainty. BMC medicine. 2013;11:1-14.
87. Hjelmfors L, Stromberg A, Friedrichsen M, Martensson J, Jaarsma T. Communicating prognosis and end-of-life care to heart failure patients: a survey of heart failure nurses' perspectives. Eur J Cardiovasc Nurs. 2014;13(2):152-61.
88. Hjelmfors L, Strömberg A, Friedrichsen M, Mårtensson J, Jaarsma T. Communication About the Heart Failure Trajectory in Patients, Their Families and Health Care Professionals. Sweden: Linkopings Universitet (Sweden); 2018.
89. Hoff L, Hermeren G. Between uncertainty and certainty. The Journal of clinical ethics. 2011;22(2):139-50.
90. Hoff L, Hermeren G. Identifying challenges to communicating with patients about their imminent death. The Journal of clinical ethics. 2014;25(4):296-306.
91. Holloway RG, Gramling R, Kelly AG. Estimating and communicating prognosis in advanced neurologic disease. Neurology. 2013;80(8):764-72.
92. Hudson BF, Shulman C, Low J, Hewett N, Daley J, Davis S, et al. Challenges to discussing palliative care with people experiencing homelessness: a qualitative study. BMJ Open. 2017;7(11):e017502.
93. Hui D, Mo L, Paiva CE. The Importance of Prognostication: Impact of Prognostic Predictions, Disclosures, Awareness, and Acceptance on Patient Outcomes. Curr Treat Options Oncol. 2021;22(2):12.
94. Hui D, Paiva CE, Del Fabbro EG, Steer C, Naberhuis J, van de Wetering M, et al. Prognostication in advanced cancer: update and directions for future research. Support Care Cancer. 2019;27(6):1973-84.
95. Innes S, Payne S. Advanced cancer patients' prognostic information preferences: a review. Palliat Med. 2009;23(1):29-39.
96. International Nurses' End-of-Life Decision-Making in Intensive Care Research G, Gallagher A, Bousso RS, McCarthy J, Kohlen H, Andrews T, et al. Negotiated reorienting: a grounded theory of nurses' end-of-life decision-making in the intensive care unit. Int J Nurs Stud. 2015;52(4):794-803.
97. Isaac M, Curtis JR. How to respond to an ICU patient asking if she/he is going to die. Intensive Care Med. 2017;43(2):220-2.
98. Islam Z, Pollock K, Patterson A, Hanjari M, Wallace L, Mururajani I, et al. Thinking ahead about medical treatments in advanced illness: a qualitative study of barriers and enablers in end-of-life care planning with patients and families from ethnically diverse backgrounds. Health Soc Care Deliv Res. 2023;11(7):1-135.
99. Jackson VA, Emanuel L. Navigating and Communicating about Serious Illness and End of Life. N Engl J Med. 2024;390(1):63-9.

For peer review only

100. Johnson M, Tod AM, Brummell S, Collins K. Prognostic communication in cancer: A critical interpretive synthesis of the literature. Eur J Oncol Nurs. 2015;19(5):554-67.
101. Johnson RF, Jr., Gustin J. Acute lung injury and acute respiratory distress syndrome requiring tracheal intubation and mechanical ventilation in the intensive care unit: impact on managing uncertainty for patient-centered communication. Am J Hosp Palliat Care. 2013;30(6):569-75.
102. Jones MB. A case analysis of the legal, professional, and ethical responsibilities of registered nurses for disclosure of prognostic information to dying adults (philosophy). United States -- Alabama: The University of Alabama at Birmingham; 1983.
103. Kantabanlang Y, Lin CP, Nilmanat K, Guo P. Palliative care needs of patients with hematologic malignancies and family caregivers and challenges of palliative care provision in Asia: A review of evidence. Semin Hematol. 2023;60(4):220-5.
104. Kapo JM, Casarett D. Review: Prognosis in chronic diseases. Annals of Long-Term Care. 2006;14(2):18-23.
105. Kimbell B. Living, dying and caring in advanced liver disease: the challenge of uncertainty. Scotland: The University of Edinburgh (United Kingdom); 2014.
106. Kimbell B, Boyd K, Kendall M, Iredale J, Murray SA. Managing uncertainty in advanced liver disease: a qualitative, multiperspective, serial interview study. BMJ Open. 2015;5(11):e009241.
107. Ko E, Fuentes D, Singh-Carlson S, Nedjat-Haiem F. Challenges and facilitators of hospice decision-making: a retrospective review of family caregivers of home hospice patients in a rural US-Mexico border region-a qualitative study. BMJ Open. 2020;10(7):e035634.
108. Krawczyk M, Gallagher R. Communicating prognostic uncertainty in potential end-of-life contexts: experiences of family members. BMC Palliat Care. 2016;15:59.
109. Kupeli N, Sampson EL, Leavey G, Harrington J, Davis S, Candy B, et al. Context, mechanisms and outcomes in end-of-life care for people with advanced dementia: family carers perspective. BMC Palliat Care. 2019;18(1):87.
110. Langmuir T, Chu A, Sehabi G, Giguere L, Lamarche J, Boudjatat W, et al. A new landscape in illness uncertainty: A systematic review and thematic synthesis of the experience of uncertainty in patients with advanced cancer receiving immunotherapy or targeted therapy. Psychooncology. 2023;32(3):356-67.
111. LeBlanc TW, Temel JS, Helft PR. "How Much Time Do I Have?": Communicating Prognosis in the Era of Exceptional Responders. Am Soc Clin Oncol Educ Book. 2018;38:787-94.
112. LeClaire MM, Oakes JM, Weinert CR. Communication of prognostic information for critically ill patients. Chest. 2005;128(3):1728-35.
113. Lee CW, Vitous CA, Silveira MJ, Forman J, Dossett LA, Mody L, et al. Delays in Palliative Care Referral Among Surgical Patients: Perspectives of Surgical Residents Across the State of Michigan. J Pain Symptom Manage. 2019;57(6):1080-8 e1.
114. Low J, Davis S, Vickerstaff V, Greenslade L, Hopkins K, Langford A, et al. Advanced chronic liver disease in the last year of life: a mixed methods study to understand how care in a specialist liver unit could be improved. BMJ Open. 2017;7(8):e016887.
115. Maguire P, Faulkner A. Communicate with cancer patients: 2. Handling uncertainty, collusion, and denial. BMJ. 1988;297(6654):972-4.

For peer review only

116. Mandel EI, Bernacki RE, Block SD. Serious Illness Conversations in ESRD. Clin J Am Soc Nephrol. 2017;12(5):854-63.
117. Marie C. Difficult conversations with dying people and their families: Marie Curie; 2014.
118. Marie C. What's stopping doctors having that conversation about death? Marie Curie. 2019.
119. Marie C. Tips for talking to your patients about a prognosis. Marie Curie. 2020.
120. Masterson MP, Applebaum AJ, Buda K, Reisch S, Rosenfeld B. Don't Shoot the Messenger: Experiences of Delivering Prognostic Information in the Context of Advanced Cancer. Am J Hosp Palliat Care. 2018;35(12):1526-31.
121. Maxwell CA, Mixon AS, Conner E, Phillippi JC. Receptivity of Hospitalized Older Adults and Family Caregivers to Prognostic Information about Aging, Injury, and Frailty: A Qualitative Study. Int J Nurs Stud. 2020;109:103602.
122. McClung JA. End-of-life care in the treatment of advanced heart failure in the elderly. Cardiol Rev. 2013;21(1):9-15.
123. McDonald A, Sherlock J. A long and winding road: Improving communication with patients in the NHS: Marie Curie; 2016.
124. McLennon SM, Uhrich M, Lasiter S, Chamness AR, Helft PR. Oncology nurses' narratives about ethical dilemmas and prognosis-related communication in advanced cancer patients. Cancer Nurs. 2013;36(2):114-21.
125. Medendorp NM, Stiggelbout AM, Aalfs CM, Han PKJ, Smets EMA, Hillen MA. A scoping review of practice recommendations for clinicians' communication of uncertainty. Health Expect. 2021;24(4):1025-43.
126. Michael N, Shah R. Communication about Advanced Progressive Disease , Prognosis, and Advance Care Plans. In: Kissane DWMW, William B, editors. Psycho-Oncology in Palliative and End of Life Care. New York: Oxford Academic; 2022. p. 1-C.P207.
127. Moglia C, Palumbo F, Botto R, Iazzolino B, Ticozzi N, Calvo A, et al. Prognostic communication in amyotrophic lateral sclerosis: findings from a Nationwide Italian survey. Neurol Sci. 2024;45(12):5787-94.
128. Molzahn AE, Sheilds L, Bruce A, Schick-Makaroff K, Antonio M, Clark AM. Life and priorities before death: A narrative inquiry of uncertainty and end of life in people with heart failure and their family members. Eur J Cardiovasc Nurs. 2020;19(7):629-37.
129. Momen NC, Barclay SI. Addressing 'the elephant on the table': barriers to end of life care conversations in heart failure - a literature review and narrative synthesis. Curr Opin Support Palliat Care. 2011;5(4):312-6.
130. Mone S, Kerr H. Prognostic awareness in advanced cancer: an integrative literature review. BMJ Support Palliat Care. 2021;11(1):53-8.
131. Mount BM. Psychological Impact of Urologic Cancer. Cancer. 1980;45 Suppl 7:1985-92.
132. Nadicksbernd J. Why Do Community Healthcare Professionals Find Some Conversations Difficult? England: University of London, Royal Holloway College (United Kingdom); 2023.
133. Nagelschmidt K, Leppin N, Seifart C, Rief W, von Blanckenburg P. Systematic mixed-method review of barriers to end-of-life communication in the family context. BMJ Support Palliat Care. 2021;11(3):253-63.

For peer review only - http://bmjopen.bmj.com/site/about/guidelines.xhtml

134. Narayan M, Jones J, Portalupi LB, McIlvennan CK, Matlock DD, Allen LA. Patient Perspectives on Communication of Individualized Survival Estimates in Heart Failure. J Card Fail. 2017;23(4):272-7.
135. Ngwenya N, Crang C, Farquhar M, Rintoul RC, Mahadeva R, Calvert LD, et al. Communicating uncertainty: contrasting the communication experiences of patients with advanced COPD and incurable lung cancer. Fam Pract. 2021;38(5):637-43.
136. Nice. Care of dying adults in the last days of life. NICE. 2015.
137. Nice. Chronic heart failure in adults: diagnosis and management. NICE. 2018.
138. Noble H, Brazil K, Burns A, Hallahan S, Normand C, Roderick P, et al. Clinician views of patient decisional conflict when deciding between dialysis and conservative management: Qualitative findings from the PAlliative Care in chronic Kidney diSease (PACKS) study. Palliat Med. 2017;31(10):921-31.
139. Olsson MM, Windsor C, Chambers S, Green TL. A Scoping Review of End-of-Life Communication in International Palliative Care Guidelines for Acute Care Settings. J Pain Symptom Manage. 2021;62(2):425-37 e2.
140. Owusuaa C, van Beelen I, van der Heide A, van der Rijt CCD. Physicians' views on the usefulness and feasibility of identifying and disclosing patients' last phase of life: a focus group study. BMJ Support Palliat Care. 2021;14(e2).
141. Pardue CM. Uncertainty in the context of end-of-life communication in heart failure. Dissertation Abstracts International: Section B: The Sciences and Engineering. 2021;82(8):No-Specified.
142. Parker S, Clayton J, Hancock K, Walder S, Butow P, Carrick S, et al. Communicating Prognosis and Issues Surrounding End of Life (EOL) in adults in the advanced stages of a life-limiting illness: A Systematic Review. 2006.
143. Parker SM, Clayton JM, Hancock K, Walder S, Butow PN, Carrick S, et al. A systematic review of prognostic/end-of-life communication with adults in the advanced stages of a life-limiting illness: patient/caregiver preferences for the content, style, and timing of information. J Pain Symptom Manage. 2007;34(1):81-93.
144. Parry R. Communication in Palliative Care and About End of Life: A State-of-the-Art Literature Review of Conversation-Analytic Research in Healthcare. Research on Language and Social Interaction. 2024;57(1):127-48.
145. Parvez S, Abdel-Kader K, Song MK, Unruh M. Conveying uncertainty in prognosis to patients with ESRD. Blood Purif. 2015;39(1-3):58-64.
146. Payne S, Burton C, Addington-Hall J, Jones A. End-of-life issues in acute stroke care: a qualitative study of the experiences and preferences of patients and families. Palliat Med. 2010;24(2):146-53.
147. Peoples HA, Boone B, Blumenthal-Barby JS, Bruce CR. How Clinician-Family Interactions Potentially Impact Clinicians' Conceptualization and Discussions Regarding Prognostic Uncertainties. J Palliat Care. 2020;35(1):29-33.
148. Perumalswami CR, Jagsi R, Goold SD. Predicting a "Lazarus Effect" in Patients With Advanced Cancer Near the End of Life: Prognostic Uncertainty, Oncologists' Emotions, and Ethical Questions. Am J Bioeth. 2019;19(12):57-60.
149. Pfeil TA, Laryionava K, Reiter-Theil S, Hiddemann W, Winkler EC. What keeps oncologists from addressing palliative care early on with incurable cancer patients? An active stance seems key. Oncologist. 2015;20(1):56-61.
150. Piovesan M, Orr P, Tevyaw S, Roussos E, Cherid C, Bouchard S. A Prospective Study with Patients and Families on the Usefulness of Accurate

For peer review only

Prognosis for Palliative Care Patients. J Soc Work End Life Palliat Care. 2024;20(2):133-46.
151. Pontin D, Jordan N. Issues in prognostication for hospital specialist palliative care doctors and nurses: a qualitative inquiry. Palliat Med. 2013;27(2):165-71.
152. Pooler C, Richman-Eisenstat J, Kalluri M. Early integrated palliative approach for idiopathic pulmonary fibrosis: A narrative study of bereaved caregivers' experiences. Palliat Med. 2018;32(9):1455-64.
153. Powers BA, Norton SA, Schmitt MH, Quill TE, Metzger M. Meaning and practice of palliative care for hospitalized older adults with life limiting illnesses. J Aging Res. 2011;2011:406164.
154. Pun J, Chow JCH, Fok L, Cheung KM. Role of patients' family members in end-of-life communication: an integrative review. BMJ Open. 2023;13(2):e067304.
155. Rafferty KA, Cramer EM, Priddis D. Managing end-of-life uncertainty: applying problematic integration theory to spousal communication about death and dying. American Journal of Hospice and Palliative Medicine®. 2016;33(1):69-76.
156. Reid C, Gibbins J, Bloor S, Burcombe M, McCoubrie R, Forbes K. Healthcare professionals' perspectives on delivering end-of-life care within acute hospital trusts: a qualitative study. BMJ Support Palliat Care. 2015;5(5):490-5.
157. Rhee JJ, Zwar NA, Kemp LA. Why are advance care planning decisions not implemented? Insights from interviews with Australian general practitioners. J Palliat Med. 2013;16(10):1197-204.
158. Richards N, Ingleton C, Gardiner C, Gott M. Awareness contexts revisited: indeterminacy in initiating discussions at the end-of-life. J Adv Nurs. 2013;69(12):2654-64.
159. Ridley S, Fisher M. Uncertainty in end-of-life care. Curr Opin Crit Care. 2013;19(6):642-7.
160. Risco JR, Kelly AG, Holloway RG. Prognostication in neurology. Handb Clin Neurol. 2022;190:175-93.
161. Robinson J, Pilbeam C, Goodwin H, Raphael D, Waterworth S, Gott M. The impact of uncertainty on bereaved family's experiences of care at the end of life: a thematic analysis of free text survey data. BMC Palliat Care. 2021;20(1):60.
162. Rowe JG, Potts M, McGhie R, Dinh A, Engel I, England K, et al. Palliative Care Practice During the COVID-19 Pandemic: A Descriptive Qualitative Study of Palliative Care Clinicians. J Pain Symptom Manage. 2021;62(6):1111-6.
163. Salins N, Dhyani VS, Mathew M, Prasad A, Rao AP, Damani A, et al. Assessing palliative care practices in intensive care units and interpreting them using the lens of appropriate care concepts. An umbrella review. Intensive Care Med. 2024;50(9):1438-58.
164. Sanders JJ, Blanch-Hartigan D, Ericson J, Tarbi E, Rizzo D, Gramling R, et al. Methodological innovations to strengthen evidence-based serious illness communication. Patient Educ Couns. 2023;114:107790.
165. Scholz B, Goncharov L, Emmerich N, Lu VN, Chapman M, Clark SJ, et al. Clinicians' accounts of communication with patients in end-of-life care contexts: A systematic review. Patient Educ Couns. 2020;103(10):1913-21.
166. Schuster RA, Hong SY, Arnold RM, White DB. Do physicians disclose uncertainty when discussing prognosis in grave critical illness? Narrat Inq Bioeth. 2012;2(2):125-35.
167. Schutz REC, Creutzfeldt CJ. Three big things in neuropalliative care: Communication, personhood and uncertainty. J Neurol Sci. 2018;385:232-3.

For peer review only

168. Sedhom R, Bates-Pappas GE, Feldman J, Elk R, Gupta A, Fisch MJ, et al. Tumor Is Not the Only Target: Ensuring Equitable Person-Centered Supportive Care in the Era of Precision Medicine. Am Soc Clin Oncol Educ Book. 2024;44(3):e434026.
169. Selman LE, Bristowe K, Higginson IJ, Murtagh FEM. The views and experiences of older people with conservatively managed renal failure: a qualitative study of communication, information and decision-making. BMC Nephrol. 2019;20(1):38.
170. Sikhosana N, Whitehead D, Moxham L, Karacsony S, Namasivayam P, Fernandez R. Voice of persons with a life-limiting illness in conversation with healthcare professionals: systematic review and meta-synthesis. BMJ Support Palliat Care. 2023.
171. Simpkin AL, Armstrong KA. Communicating Uncertainty: a Narrative Review and Framework for Future Research. J Gen Intern Med. 2019;34(11):2586-91.
172. Smith AK, Glare P. Ethical issues in prognosis and prognostication. The Oxford handbook of ethics at the end of life. 2014:170-89.
173. Stanek MS, Norton SA. Expressions of Uncertainty from Patients with Advanced Cancer when Communicating with the Palliative Care Team. United States -- New York: University of Rochester; 2020.
174. Stiel H, Nagarajan SV, Forster BC, Clayton JM. Palliative care physicians' perspectives on transferring patients to nursing homes and communication strategies to facilitate this transition: A qualitative study. Palliat Med. 2019;33(3):323-31.
175. Stocker R. Experiences and perceptions of the role of palliative and end of life care in heart failure: a modified grounded theory study. England: University of Durham (United Kingdom); 2014.
176. Stocker R, Close H, Hancock H, Hungin APS. Should heart failure be regarded as a terminal illness requiring palliative care? A study of heart failure patients', carers' and clinicians' understanding of heart failure prognosis and its management. BMJ Support Palliat Care. 2017;7(4):464-9.
177. Sullivan AM, Lakoma MD, Matsuyama RK, Rosenblatt L, Arnold RM, Block SD. Diagnosing and discussing imminent death in the hospital: a secondary analysis of physician interviews. J Palliat Med. 2007;10(4):882-93.
178. Szawarski P. Classic cases revisited: Oscar the cat and predicting death. J Intensive Care Soc. 2016;17(4):341-5.
179. Tarbi EC, Gramling R, Bradway C, Broden EG, Meghani SH. "I Had a Lot More Planned": The Existential Dimensions of Prognosis Communication with Adults with Advanced Cancer. J Palliat Med. 2021;24(10):1443-54.
180. Tavares N, Hunt KJ, Jarrett N, Wilkinson TM. The preferences of patients with chronic obstructive pulmonary disease are to discuss palliative care plans with familiar respiratory clinicians, but to delay conversations until their condition deteriorates: A study guided by interpretative phenomenological analysis. Palliat Med. 2020;34(10):1361-73.
181. Tavares NC. Palliative Care Communication in Copd : Patients' Preferences and Clinicians' Judgements. England: University of Southampton (United Kingdom); 2019.
182. Temel JS, Shaw AT, Greer JA. Challenge of Prognostic Uncertainty in the Modern Era of Cancer Therapeutics. J Clin Oncol. 2016;34(30):3605-8.

For peer review only

183. Tetteh DA, Akhther N. Openness and topic avoidance in interpersonal communication about ovarian cancer: An uncertainty management perspective. Qual Res Med Healthc. 2021;5(3):9376.
184. Tilburgs B, Vernooij-Dassen M, Koopmans R, van Gennip H, Engels Y, Perry M. Barriers and facilitators for GPs in dementia advance care planning: A systematic integrative review. PLoS One. 2018;13(6):e0198535.
185. Tolsa L, Jones L, Michel P, Borasio GD, Jox RJ, Rutz Voumard R. 'We Have Guidelines, but We Can Also Be Artists': Neurologists Discuss Prognostic Uncertainty, Cognitive Biases, and Scoring Tools. Brain Sci. 2022;12(11):1591.
186. Trankle SA, Shanmugam S, Lewis E, Nicholson M, Hillman K, Cardona M. Are We Making Progress on Communication with People Who Are Near the End of Life in the Australian Health System? A Thematic Analysis. Health Commun. 2020;35(2):158-67.
187. Tuck KK, Brod L, Nutt J, Fromme EK. Preferences of patients with Parkinson's disease for communication about advanced care planning. Am J Hosp Palliat Care. 2015;32(1):68-77.
188. Udo C, Lovgren M, Lundquist G, Axelsson B. Palliative care physicians' experiences of end-of-life communication: A focus group study. Eur J Cancer Care (Engl). 2018;27(1).
189. van der Velden NCA, Han PKJ, van Laarhoven HWM, de Vos F, Hendriks LEL, Burgers SA, et al. Prevalence and Predictors of Physician-Patient Discordance in Prognostic Perceptions in Advanced Cancer. Oncologist. 2023;28(8):e653-e68.
190. van der Velden NCA, Meijers MC, Han PKJ, van Laarhoven HWM, Smets EMA, Henselmans I. The Effect of Prognostic Communication on Patient Outcomes in Palliative Cancer Care: a Systematic Review. Curr Treat Options Oncol. 2020;21(5):40.
191. van der Velden NCA, Smets EMA, van Vliet LM, Brom L, van Laarhoven HWM, Henselmans I. Effects of Prognostic Communication Strategies on Prognostic Perceptions, Treatment Decisions and End-Of-Life Anticipation in Advanced Cancer: An Experimental Study among Analogue Patients. J Pain Symptom Manage. 2024;67(6):478-89 e13.
192. van der Velden NCA, Smets EMA, van Vliet LM, Brom L, van Laarhoven HWM, Henselmans I. Effects of prognostic communication strategies on emotions, coping, and appreciation of consultations: An experimental study in advanced cancer. Palliat Support Care. 2024:1-13.
193. van der Velden NCA, van der Kleij MBA, Lehmann V, Smets EMA, Stouthard JML, Henselmans I, et al. Communication about Prognosis during Patient-Initiated Second Opinion Consultations in Advanced Cancer Care: An Observational Qualitative Analysis. Int J Environ Res Public Health. 2021;18(11).
194. van Eenennaam RM, Kruithof WJ, van Es MA, Kruitwagen-van Reenen ET, Westeneng HJ, Visser-Meily JMA, et al. Discussing personalized prognosis in amyotrophic lateral sclerosis: development of a communication guide. BMC Neurol. 2020;20(1):446.
195. Vlckova K, Tuckova A, Polakova K, Loucka M. Factors associated with prognostic awareness in patients with cancer: A systematic review. Psychooncology. 2020;29(6):990-1003.
196. Walczak A, Butow PN, Bu S, Clayton JM. A systematic review of evidence for end-of-life communication interventions: Who do they target, how are they structured and do they work? Patient Educ Couns. 2016;99(1):3-16.

For peer review only

197. Walczak A, Butow PN, Davidson PM, Bellemore FA, Tattersall MH, Clayton JM, et al. Patient perspectives regarding communication about prognosis and end-of-life issues: how can it be optimised? Patient Educ Couns. 2013;90(3):307-14.
198. Waldrop DP, Meeker MA, Kerr C, Skretny J, Tangeman J, Milch R. The nature and timing of family-provider communication in late-stage cancer: a qualitative study of caregivers' experiences. J Pain Symptom Manage. 2012;43(2):182-94.
199. Wijsenbeek MS, Bonella F, Orsatti L, Russell AM, Valenzuela C, Wuyts WA, et al. Communicating with patients with idiopathic pulmonary fibrosis: can we do it better? ERJ Open Res. 2022;8(1):00422-2021.
200. Wittenberg SM, Cohen LM. Estimating prognosis in end-stage renal disease. Progress in Palliative Care. 2013;17(4):165-9.
201. Wittenberg-Lyles EM, Goldsmith J, Sanchez-Reilly S, Ragan SL. Communicating a terminal prognosis in a palliative care setting: deficiencies in current communication training protocols. Soc Sci Med. 2008;66(11):2356-65.
202. Wu Y, Zhang X. Examining Conversation Analysis in Palliative Care: A Systematic Review. Health Commun. 2024;39(13):3072-83.
203. Xie W, Butcher R. Artificial Intelligence Decision Support Tools for End-of-Life Care Planning Conversations: CADTH Horizon Scan. 2023.
204. Young J, Lyons A, Egan R, Dew K. Embodied decisions unfolding over time: a meta-ethnography systematic review of people with cancer's reasons for delaying or declining end-of-life care. BMC Palliat Care. 2024;23(1):45.
205. Ziegler DS. Never say never. J Clin Oncol. 2015;33(5):518-20.

For peer review only

BMJ Open

Improving Communication of Prognostic Uncertainty in Advanced Illness: A Realist Review

Journal:	*BMJ Open*
Manuscript ID	bmjopen-2025-115385.R1
Article Type:	Original research
Date Submitted by the Author:	11-May-2026
Complete List of Authors:	Etkind, Simon; University of Cambridge, Department of Public Health and Primary Care; Cambridge University Hospitals NHS Foundation Trust<br>Weetman, Katharine; University of Birmingham College of Medical and Dental Sciences<br>Hyams, Katy; University of Cambridge, Department of Public Health and Primary Care<br>Acharya, Tanvi; University of Cambridge, Department of Public Health and Primary Care<br>Shokraneh, Farhad; University of Cambridge, Department of Public Health and Primary Care<br>Macartney, John; University of Warwick Medical School
**Primary Subject Heading**:	Communication
Secondary Subject Heading:	General practice / Family practice, Geriatric medicine, Palliative care, Medical education and training
Keywords:	INTERNAL MEDICINE, PALLIATIVE CARE, GERIATRIC MEDICINE, Prognosis, Systematic Review

SCHOLARONE™ MANUSCRIPTS

I, the Submitting Author has the right to grant and does grant on behalf of all authors of the Work (as defined in the below author licence), an exclusive licence and/or a non-exclusive licence for contributions from authors who are: i) UK Crown employees; ii) where BMJ has agreed a CC-BY licence shall apply, and/or iii) in accordance with the terms applicable for US Federal Government officers or employees acting as part of their official duties; on a worldwide, perpetual, irrevocable, royalty-free basis to BMJ Publishing Group Ltd ("BMJ") its licensees and where the relevant Journal is co-owned by BMJ to the co-owners of the Journal, to publish the Work in this journal and any other BMJ products and to exploit all rights, as set out in our licence.

The Submitting Author accepts and understands that any supply made under these terms is made by BMJ to the Submitting Author unless you are acting as an employee on behalf of your employer or a postgraduate student of an affiliated institution which is paying any applicable article publishing charge ("APC") for Open Access articles. Where the Submitting Author wishes to make the Work available on an Open Access basis (and intends to pay the relevant APC), the terms of reuse of such Open Access shall be governed by a Creative Commons licence – details of these licences and which Creative Commons licence will apply to this Work are set out in our licence referred to above.

Other than as permitted in any relevant BMJ Author's Self Archiving Policies, I confirm this Work has not been accepted for publication elsewhere, is not being considered for publication elsewhere and does not duplicate material already published. I confirm all authors consent to publication of this Work and authorise the granting of this licence.

For peer review only

Improving Communication of Prognostic Uncertainty in Advanced Illness: A Realist Review

Authors

Simon Etkind*, (0000-0003-2863-8893) Assistant Professor of Palliative Care. sde23@medschl.cam.ac.uk[1,2] East Forvie Building, Forvie Site, Robinson Way, Cambridge, CB20SR *corresponding author.

Katharine Weetman, (0000-0003-0377-3933) Assistant Professor in Clinical Communication. k.e.weetman@bham.ac.uk[3]

Katy Hyams, Academic Clinical Fellow. kh851@cam.ac.uk[1]

Tanvi Acharya, Academic Clinical Fellow. ta387@medschl.cam.ac.uk[1]

Farhad Shokraneh, (0000-0001-9687-856) Research Associate. farhad@systematicreview.info[1]

John I MacArtney, (0000-0002-0879-4277) Associate Professor. John.Macartney@warwick.ac.uk[4]

Affiliations:

1. Primary Care Unit, University of Cambridge
2. Cambridge University Hospitals NHS Foundation Trust
3. School of Medical Sciences, College of Medicine & Health, University of Birmingham, B15 2TT
4. Warwick Medical School, University of Warwick

For peer review only

Abstract

Objectives

Prognostic uncertainty is common in advanced illness, driven by multimorbidity, treatment complexities, and unpredictable disease trajectories. When inadequately acknowledged or discussed, it can lead to patient and family distress and inhibit decision making.

We aimed to build a programme theory of how, why, and for whom communication of prognostic uncertainty “works” (or not) in advanced illness and palliative care contexts.

Design

Realist systematic review, reported according to RAMESES criteria

Data Sources

Embase, MEDLINE, CINAHL, Cochrane Database of Systematic Reviews, SCI via Web of Science, PsycINFO, SSCI and multiple grey literature sources

Eligibility Criteria evidence examining communication of prognostic uncertainty in adults with advanced illnesses.

Data Extraction and synthesis

We coded papers qualitatively to identify Context–Mechanism–Outcome Configurations (CMOCs) relevant to prognostic uncertainty communication. Supported by clinical and lay stakeholder consultation, we refined these into a programme theory.

Results

From 4634 search results we included 205 studies, with 46,579 participants across 11 countries. The final programme theory identified contexts of communication: Socio-cultural norms and values, patient and family preferences, communication models and interventions, clinician uncertainty tolerance, training and experience. Mechanisms of communication were mediated by the care relationship; timing and initiation of discussion; content of communication; explicit vs implicit communication. Relevant outcomes were emotions, hope, distress, decision making and care planning. There was no single model of effective communication as this varied based on individual characteristics and preferences, but openness was usually viewed positively.

Conclusion

This programme theory provides a new understanding of the contexts and mechanisms, such as explicitness of discussion, that support effective communication of prognostic uncertainty across advanced illness contexts to reduce distress and aid decision making. Sociocultural factors are important, but have the most limited supporting evidence, especially in low- and middle-income countries.

For peer review only - http://bmjopen.bmj.com/site/about/guidelines.xhtml

The model presented here may support professionals in individualised and compassionate communication of prognostic uncertainty

Strengths and Limitations of the study:

- This review used an extensive search designed by an information specialist which enabled synthesis of a large and diverse literature across multiple illness contexts
- The realist design enabled theory development to explain how communication of prognostic uncertainty works, for whom and in what contexts
- Stakeholder involvement ensured relevance to the population of interest
- Whilst there were no language limitations in our search, we searched primarily English language databases which may have limited our ability to access literature in other languages
- In line with the realist approach, we aimed to synthesise sufficient literature to develop a programme theory rather than finding all existing relevant literature

For peer review only

Background:

People with advanced illnesses commonly experience uncertainty in physical, social, practical, temporal and existential domains[1-3]. Whilst some illness uncertainties can be resolved e.g. through diagnostic testing or information provision, many cannot[4,5]. How uncertainty is managed and communicated is therefore increasingly important for those with serious illness and those caring for them[6], and is a recognised research priority[7].

Prognostic uncertainty is a specific subset of illness uncertainty that is increasing due to population changes and increasing illness complexity[8,9]. It can affect healthcare delivery in multiple settings by inhibiting timely end of life discussions and identification of needs[10], and can be a barrier for accessing services and benefits (e.g. palliative care, special rules access to benefits) that are gated by prognosis[11,12]. Consequently, poor communication of prognostic uncertainty may worsen inequities in the context of rising palliative care need[13,14]. For this reason, the importance of communicating prognostic uncertainty is increasingly recognised[7,15,16]

Most patients prefer open discussions of prognostic uncertainty, but such communication is often avoided or implicit[17-19], which may partly be attributed to health professional intolerance of uncertainty, perceptions that expressing uncertainty is a sign of failure, or clinical inexperience[17,18]. If poorly communicated or unshared, prognostic uncertainty can be distressing for patients, carers, and health professionals alike, inhibiting decision making.[3]

Communication of prognosis itself is well researched[15,20]. However, there is no evidence synthesis to inform how prognostic *uncertainty* can be best communicated to improve patient outcomes, and existing research has not explicitly focused on the mechanisms of communication. Many patient, health system, and wider factors are likely to affect optimal communication,[21,22] so an explanatory approach is needed to inform preferred approaches to prognostic uncertainty communication, whilst accounting for these contextual factors.

We aimed to identify, understand, and explain current practice of communicating prognostic uncertainty in advanced illness contexts, including how and why different outcomes are achieved through verbal and written communications between healthcare professionals, patients, and their relatives and/or carers.

For peer review only - http://bmjopen.bmj.com/site/about/guidelines.xhtml

Methods

Review question

How, why, and for whom does the communication of prognostic uncertainty "work" (or not) in serious illness contexts potentially relevant to palliative and end-of-life care?

Design

Stakeholder informed realist systematic review and synthesis, reported according to the RAMESES standards (appendix 1)[23]. We chose this theory-driven approach to generate explanations of how communicating prognostic uncertainty may be theorised to "work" (or not), and if so, under what contexts, for whom, why, and to what extent [24,25]. Realist methodology is particularly suited to investigate complex interventions and sociological issues, such as that under scrutiny here[9,24,26]. The review protocol is registered on Prospero (CRD42024563546). This was a literature review, therefore ethical approval was not required

Patient and public involvement

The review was supported by an advisory group of two clinicians (one GP, one palliative care consultant), and two lay advisors with relevant lived experience. The group comprised advisors from across the UK and were from a range of cultural backgrounds.

We consulted the advisory group throughout the review to ensure the question and programme theory fit with lived experience. Stakeholders contributed to developing the initial and final programme theories, and refined the CMOC list.

Inclusion and exclusion criteria

We included evidence relating to verbal or written communication of prognostic uncertainty in advanced and life-limiting illness contexts relevant to palliative care. We included communication between healthcare professionals AND patients and/or relatives and/or carers and/or other healthcare professionals, in any healthcare setting. We included all study designs, as long as they contained content that could be scrutinised against the initial programme theory and contained at least one statement regarding how or for whom communication of prognostic uncertainty works and why[24,25]. We included published peer reviewed papers, and grey literature such as reports, theses and guidelines.[25,27]

For peer review only - http://bmjopen.bmj.com/site/about/guidelines.xhtml

Exclusion criteria: Prognostic uncertainty for patients without advanced or life-limiting illness; evidence specifically related to patients <18 years old, communication of prognosis without specific consideration of uncertainty.

Initial programme theory

On 29th April 2024, we conducted scoping searches in PubMed and Google Scholar using a title search for terms prognosis, palliative, end-of-life, communication, and uncertainty and identified 54 highly relevant papers.[28,29] These papers were reviewed by four authors (SE, KW, FS, JM) who each produced summary points and independently developed ideas for an initial programme theory. The initial theory was then refined through repeated discussion of these ideas within the research team and patient and clinician advisory group.[24,25] We produced a visual 'map' of the initial theory (appendix 2) in collaboration with the advisory group. At each review of the theory we monitored its iterative development, and considered it sufficiently developed when no major conceptual changes were identified. The initial theory formed the starting point for our searches and analysis (appendix 2).

Searches

Following realist review guidelines,[28,30,31] we searched the following sources with no limitations to time, language, document type, or publication status on 18th August 2024[32]: Embase, MEDLINE, CINAHL, Cochrane Database of Systematic Reviews, Science Citation Index-Expanded via Web of Science, PsycINFO and the Social Science Citation Index. To identify grey literature, we searched the following websites and repositories: NHS Digital, NHS Improvement, Royal College of Physicians, General Medical Council, NICE Guidelines, Marie Curie, Google Scholar, ProQuest Dissertations & Theses Global, Trip Database and the Department of Health and Social Care.

The search strategy was designed with an information specialist (FS) and included terms relating to Prognosis; Uncertainty; and Palliative care/Advanced illness; search terminology was informed by papers from the scoping search (appendix 3).

Screening and data extraction.

A single reviewer screened search results for eligibility, with 10% second screening at title/abstract stage, as per previous realist reviews.[27,33] We assessed inter-rater agreement on these 10% and considered a score of k>0.8 to reflect sufficient agreement. 30% of results were double screened at the full text stage. Disagreements were resolved by discussion within the research team (SE, TA, KW &

For peer review only - http://bmjopen.bmj.com/site/about/guidelines.xhtml

FS) with reference to the inclusion/exclusion criteria. These discussions enabled a consistent interpretation of the inclusion criteria in subsequent screening decisions.

We used a bespoke data extraction form to extract relevant information from each article, populated by one researcher, with 10% double data extraction (appendix 4).

To extract data relevant to "context, mechanism, outcome" configurations (CMOCs), we imported the full text of included articles into NVivo (version 14 with collaboration cloud, QSR international). TA, FS, KW, and SE coded included articles to identify text relevant to the programme theory. We coded generally for areas of ‘context’, ‘mechanism’ and ‘outcome, and specifically for areas such as “content of communication” that could contribute to CMOCs. The coding framework was regularly reviewed and updated by the research team during data extraction.

Synthesis

Based on the coding of "contexts, mechanisms, and outcomes”, we developed a series of CMOCs related to the review question, i.e. statements relating to communicating prognostic uncertainty where a context could be linked to a mechanism and outcome. These were tabulated during coding and each new CMOC was checked against the existing list to avoid repetition. The full list was subsequently checked after analysis and 4 overlapping CMOCs were combined

The research team reviewed the CMOC list in relation to the domains of the initial programme theory, and generated thematic areas which CMOCs related to. Additional domains which extended the initial programme theory were also identified during coding through reflexive consideration of the CMOCs coded..

The final programme theory was developed by considering the relationships of the thematic areas (and the CMOCs within them) to each other. We did not attempt to categorise thematic areas themselves as “contexts”, “mechanisms” or “outcomes” because most themes bridged across these categories, but we did consider interactions between thematic areas in the context of prognostic uncertainty communication to build a visual model of the programme theory.

The research team then considered whether and how each CMOC fit within the theory; Where needed, we adjusted the configuration of the theory to ensure all CMOCs ‘fit’ We also conducted one round of written feedback and one meeting with the advisory group to support theory development

Rigour and relevance assessments

We made a judgement of low, moderate or high rigour, based on an evaluation of the research methods in relation to the question being asked by each manuscript[34].

For peer review only - http://bmjopen.bmj.com/site/about/guidelines.xhtml

Papers collecting primary data were rated as 'high' rigour where methods were appropriate for the question, and there were no flaws identified that affected trustworthiness of the findings. If minor biases or issues were identified, we rated as moderate. If serious concerns limited interpretation, we rated as low rigour. Literature reviews were rated as low rigour if there was no description of a search strategy. Systematic reviews were rated as moderate or high rigour. Rigour was assessed by KH with 25% checked by SE, Relevance was assessed by KH with 50% checked by SE.

We also assessed relevance for all included papers[34]. Relevance was defined as the alignment of each paper to the review question and initial programme theory, and papers were rated as high, moderate, or low relevance based on the degree of focus on our research question

Rigour and relevance scored were available to the research team at the point of theory development, which enabled us to adjust the weight given to CMOCs depending on the evidence supporting them, however we were mindful that in some cases, useful concepts can be identified in 'bad' research.[35]

For peer review only

Results

From 4634 search results we included 205 articles (Figure 1). (Cohens K 0.88 for agreement in double screened articles). Articles were mostly from the USA (71), UK (61), and continental Europe (27). Other countries were Canada (14), Australia (20), New Zealand (2), Ghana (1), Hong Kong (1), India (1), and China (1).

[INSERT FIGURE 1 HERE]

Most articles were qualitative analyses (79) or systematic/narrative literature reviews (71). We also included observational quantitative or mixed-methods studies, theses, policy documents, opinion or discussion articles, and book chapters. The 103 studies involving primary data collection had 46,579 unique participants, including 39,448 patients, 3,959 healthcare professionals, and 2003 carers. 82 articles covered all or mixed settings, with 75 in secondary care, 20 in primary or community care and 11 in hospice settings. 41 focused on HCPs only, 14 on patients, 15 on carers, and 102 on combinations of HCPs, patients and/or carers. The most common patient populations were cancer, heart failure, general palliative care and intensive care.

82 articles were of high rigour, 76 moderate, and 47 low. Most low rigour articles did not involve primary data collection. 63 articles were highly relevant to the programme theory, 91 moderately relevant, and 51 were of low relevance. (appendix 5)

Context–mechanism–outcome configurations

We identified 52 context-mechanism-outcome configurations [CMOC] within eight thematic areas.1. Patient and family preferences; 2. clinician training and experience; 3. prognostic communication models and interventions; 4. timing and initiation of prognostic dialogue; 5. content of communication; 6. explicit disclosure of uncertainty and open communication; 7. Implicit or avoided disclosure of information and uncertainty. 8. emotions, hope and distress**.** We elaborate on each of these below; examples of how CMOCs relate to the findings are given in the text. The full list of CMOCs is included in appendix 6.

Based on discussion of these areas with stakeholder and PPI contributors, we developed a final programme theory (**figure 2)**. References for all included articles are available in appendix 7.

[INSERT FIGURE 2 HERE]

1. *Patient and family preferences*

For peer review only - http://bmjopen.bmj.com/site/about/guidelines.xhtml

The inherent uncertainty in life-limiting illness cannot be eliminated[1,36,37] (e.g. difficulty of frailty prognostication[38], rare diseases[39], ICU contexts[40-42]) and patients and carers may expect a degree of prognostic uncertainty within life-limiting illness contexts[43]. Most included studies identified that patients usually want sensitive and honest communication by clinicians when prognosis is uncertain[44-50]; paired with empathy [CMOC9,11] [51-53] and a sense of optimism[16,49] or hopefulness[53-55] (but not false hope[19,41,56-58])[CMOC13]. In order for patients and families to plan ahead in physical, financial, social and emotional domains[39,42,59-61,62] [CMOC6] and make informed decisions at the end of their life[60,63] (e.g. regarding place of death), they need to be given the option of knowing prognostic information alongside realistic expectations and timeframes[64,65]. Unrealistic expectations can lead to overly aggressive treatment plans [CMOC35, 36, 41][54,66-69].

Internationally, although many people desire prognostic estimates and to be fully informed of prognostic uncertainty [CMOC5][16,19,54,59,62,66,68,70-81], information needs vary[39,52,60,72,74-76,82-86]; not all patients want to know [CMOC7][39,44,54,60,78,82,87-90]. In some situations. patients or families may be understood by healthcare professionals as being "in denial"[54,64,75,85], and if so, prognostic disclosure may be perceived to cause distress[55] or challenge a positive outlook[39,76,91,92]. Therefore, providing patients with informational choice and ascertaining individual preferences[6,46,67,70,82,93,94] is an inclusive approach, alongside consideration of sociocultural factors [CMOC1, 10][52,53,56,68,75,81,95,96]. Respecting cultural or social norms and religious or spiritual needs is important [CMOC47, 48][56,58,62,63,96,97] and may influence requests or preferences for prognostic information [CMOC46][53,54,56,63,96,98].

A few studies suggested some patient groups are less likely to want prognostic information such as older patients, those with lower education, higher anxiety, shorter life-expectancy, and non-Northern European cultures[53,75,86]. However, assumptions and stereotyping should be avoided[46,86], particularly as carer and clinician perceptions of patient preference may be inaccurate[53,79,94]. A problematic situation can occur where patient and family/carer needs differ [CMOC39][59,79,83,94], for example where families do not want patients to know the prognosis[57,58,75,79,88,96,99] to protect them or sustain hope [CMOC10, 49, 50, 51][75,79,96,99]. Such requests for concealment may challenge norms of autonomy and lead to professionals feeling conflicted[57,88]. A few studies suggested that separate conversations with patients and families may be required [CMOC52][53,83], but erred towards respect for patient needs, consent, and confidentiality[53,75,98].

2. *Clinician training and experience*

A key factor affecting whether discussions of prognostic uncertainty occur, is the baseline clinician tolerance of uncertainty [CMOC 22, 26, 27, 29][1,6,55,69,100,101]. This was influenced by the nature of the relevant illness or condition and clinicians'

For peer review only - http://bmjopen.bmj.com/site/about/guidelines.xhtml

experience[102-105]. Many studies concur that clinicians may feel underprepared or lack training in how to approach and navigate prognostic discussions[55,74,78,80,93,96,106-111]. As such, a lack of training, and low confidence in communicating uncertainty, were key issues in preventing timely and effective prognostic discussions from taking place [CMOC18, 20, 25][44,50,67,89,112-116]. One of the specific training needs identified was skill in prognostication and addressing the tendency of clinicians to overestimate prognosis[57,63,83,110,117] , whether or not they acknowledge uncertainty. A further frequently cited training need was communication skills training[39,64,83,88,93,94,96,118,119]. Some studies report that clinicians with more experience may be more likely to initiate and engage in prognostic discussions[57,114,120,121], although evidence on this is conflicting[114]. Nonetheless, training is needed to enable optimal uncertainty communication practices[1,55].

3. *Prognostic communication models and interventions*

We identified many models to facilitate description of prognosis, and interventions to facilitate its communication.[1,15,43,55,68,73,78,94,99,115,122-127]. These approaches often covered framing of prognostic uncertainty e.g through categorical timeframe predictions[90,117], probabilities of risk[123], probabilistic estimates[43] or likelihoods of death within specified timeframes [90] e.g. 'likelihood a patient will die in the next week or the next month'[117], and individualised survival estimates[128]. Such tools may increase confidence in the degree of uncertainty surrounding a prognosis and so improve disclosure of prognostic information [CMOC17, 40][115], however they cannot eliminate uncertainty inherent to prognostic estimates[16,123]. Notably, prognostication and use and accuracy of tools may be affected by clinician experience[117,129], uncertainty tolerance [CMOC29][69], familiarity with the patient[115], and speciality[117]; clinicians commonly overestimate prognosis[52,117,130] so tools such as the "surprise" question [113] tend to show limited accuracy, although they can promote a holistic approach[113].

Communication models and interventions specifically addressing communication of uncertain prognosis included the best / worst case framework [CMOC6][97,131,132] , question prompt lists or sheets [46,90], Serious Illness Conversation Guide[68], Psychosocial Assessment and Communication Evaluation (PACE)[1,40], and checking understanding[86,122] and more specifically the 'ask tell ask' approach whereby information known is elicited followed by information provision and checking understanding[44,133]. This is pertinent as discrepancies between doctor's perception of patient's understanding and the patient's perception of understanding are commonly identified[46,53,79,90]. Gauging and clarifying a desire for information was also described as a "drip-feed" communication strategy[55]. Other communication strategies included combining verbal and written information[16,60] (e.g. numerical versus word-based estimates[58,134,135]), breaking bad news strategies such as SPIKES (Setting, Perception, Invitation, Knowledge, Empathy, and Strategy)[68,93,122],

For peer review only

balancing hoping for the best against worry[37] or preparing for the worst[47,76,109,136]. Colour-coded pictographs[48] and graphics to represent risks were suggested as useful,[123] Though one study found that patients preferred words to diagrams[58].

4. *Timing and initiation of prognostic dialogue*

Timing of prognostic conversations was widely referenced[46,50,60,98,111,137-139], with some healthcare professionals delaying prognostic discussions until the patient was closer to the end of their life and uncertainty was reduced [CMOC26][39,63,66,80,89,109,124,140]. A patient appearing 'well' may increase clinician reluctance to initiate prognostic discussions[38,39,44]. A person's needs and preferences may change over time[52,53,60,114] so multiple opportunities for conversations and continuous communication is key[39,44,50,63,114,141,142]; the content and timing of the discussion may need negotiating[46,55] and the 'right time' [64] may vary between individuals[39,64,108,143]. The suggested time point for prognostic conversations was often early in the course of illness[54,55,77,80,107,114,118,129,141,144] i.e. at the time of or shortly after diagnosis[16,53,55,60,85,86] so that patients are well enough to be active in conversation[44]. Several studies cited patients, families, and specialist palliative clinicians, who felt that prognostic uncertainty was raised too late[19,72,89,128,145-148]; this missed opportunity can cause regret for caregivers, and may lead to unresolved bereavement narratives [CMOC7, 21][72,146].

Some studies noted ethical and legal principles affecting initiation of discussions. In addition to promotion of autonomy, truth-telling, and informed consent[17,46,87], it is a legal requirement in many countries that discussions about prognosis take place[46] and are documented in the dying person's care record[56,119,149]. However, despite requirements for disclosure and documentation, prognostic discussions do not necessarily occur in practice, and the effect of prognostic uncertainty on discussions in this context is not known[65,96,140 55,117]. There is a further risk, or perception of risk, that appearing unsure, or providing an inaccurate prognosis could cause patient or reputational harm[130,150]. The ethical duty of preventing harm can therefore increase reluctance to initiate prognostic uncertainty discussions[77,96].

Important considerations for initiating conversations about prognosis include establishing readiness[55,134,135,151,152] e.g. recognising cues[85], as well as current understanding [CMOC1][56,86,140,151,153,154] and preferences[72,94,109], consent to disclose[64], and asking how much they would like to know[49,56,134,135,155,156]. The physical setting can also be pertinent [CMOC45][86], it must be safe, confidential and have appropriate people present[55,56,62,75,86,157]. However, in practice such conversations can be either planned or spontaneous [158]. In person was usually deemed most appropriate[93] ,not soon after surgery[60] or by phone[88].

Prognostic uncertainty conversations should ideally be led by clinicians with a good relationship and knowledge of the patient [CMOC8][55,62,75,126,130,159]. Disclosure of

For peer review only - http://bmjopen.bmj.com/site/about/guidelines.xhtml

prognostic uncertainty in this context, may strengthen trust [CMOC12][39,51,58,61,74,126]. In some families and cultures (e.g. Chinese[79]), it may be normalised or preferred that patients are informed of their prognosis via family members[62,96] and/or that the family decide what information is shared with the patient[75,79,96]. Nonetheless, many studies argued that healthcare professionals are ultimately responsible for raising prognostic uncertainty[46,66,74,85,89,129,142,160]. Professionals may, however, feel that another clinician or speciality may be better placed to lead the conversation[77,89,156,161]. Ambiguity as to who should initiate a discussion can lead to hesitation and avoidance[65,76,80,109,110,114,118,137,146], especially in situations of poorly coordinated care. Healthcare professional attitudes and behaviours may vary by region or country[75,96] with some cultures deeming prognostic disclosure as prejudicial[96]. Other factors affecting timely conversations were: resource constraints such as limited time [CMOC43][63,96,106,113,115,162], particularly during appointments[47,80,85,89,96,112]; healthcare professional reluctance [CMOC44][46,65,71,140,163]; paternalistic medical culture[46]; fear of causing depression and distress[61,81,112] or patient harm[96,110] e.g. through taking away hope[78,81,106,115]; lack of private or suitable space for the discussion[116]; language barriers[57,62]; lack of healthcare communication skills and confidence in communicating uncertainty [CMOC18, 20][44,50,89,112-115]; lack of training[55,78,106,107]; fear of damaging the clinician-patient relationship[120,134,135], and notably uncertainty itself as a "barrier" [CMOC27][41,51,63,69,106,109,110,113,114,125,142,148,164,165].

The mechanism of healthcare professionals having difficulty in 'managing their own emotions' as a professional[137] as well as the impact of uncertainty on their professional identity and sense of competence and knowledge, could lead to the outcome of failing to raise prognostic uncertainty [102,126]. One study suggested that perception of time taken to explain prognostic uncertainty may be greater than the actual time required[106]. However, these discussions need to go 'at the patient's own pace'[44]. There is a balance in a healthcare professional raising the topic of prognosis and uncertainty whilst retaining honesty and fostering hope[89,152]; for this reason, some healthcare professionals waited to discuss prognosis until asked by the patient [CMOC4][39,44,50,76,85,89,96,113,145]. Discussions may also be indirectly initiated by healthcare professionals and then 'made explicit by patients'[122] or vice versa[76,83,130]. On the contrary, some patients preferred, waited for, or expected clinicians to initiate[39,44,50,64,74,89,109,156], whilst other patients initiated and/or controlled such discussions[39,75,76,130,152]. Moreover, there is evidence of relatives or companions requesting prognostic information or 'time estimate requests' to initiate the dialogue with the healthcare professional [CMOC4][73,76,130,146,153]. There is a risk that if all parties wait for others to initiate, along with the other issues identified above, a conversation does not take place[44,107,119,121]. Open and timely negotiation of who is responsible for initiating prognostic conversations is required[121].

5. *Content of communication*

For peer review only - http://bmjopen.bmj.com/site/about/guidelines.xhtml

Tailoring the language used to the recipient and illness context with compassion is essential when discussing an uncertain prognosis[64,85]. Some patients may prefer sensitivity and others may prioritise directness[64]. Medical jargon without explanation, ambiguous language, and euphemisms should be avoided [CMOC32][19,53,86,119,151,153]; health professionals are directed to use specific terms such as 'death' and 'dying'[86,153], although language should be individualised, and in high context cultures such directness may be less appropriate[122].

These conversations also provide patients and their families opportunities to ask questions and talk about their fears[56]. There was a tendency for patients and families to prefer clinicians to be honest about their prognosis and the uncertainty of this[43-46,106,166]; they often value clear, timely and consistent prognostic information about what is likely to happen and the expected disease progression and life expectancy[62,94,108,114,130]. Although many patients may prefer explicit communication of statistics and survival likelihoods [16,74-76,87], others may prefer vagueness or ambiguity to minimise shock and distress, so an individualised or personalised approach is important[46,70,73,79].

Closing the conversation should include summarisation of the discussion alongside signposting to resources and support[167], including planning for the future[55,130] and symptom control[168], and any follow up[93]. Some clinicians and patients or families prefer to end the discussion on "certainty" or controllable topics such as pain management and reassurance about prioritisation of the patient's comfort [CMOC23][168]; this is sometimes referred to as emphasising the positive[74,83,133,168].

6. *Explicit disclosure of uncertainty and honest communication*

A patient's prognosis and individual disease progression is inherently uncertain[42,106,151,153,155] and therefore clinicians tolerating and normalising[146] this uncertainty is important for a patient- and family- centred approach[37,52] and improving palliative and end of life care [CMOC3][42,169]. Research evidence[37,55,75,96,98,112,151,153,170] and guidance[82,166] acknowledges the potential benefits of open, sensitive, and honest communication regarding a patient's prognosis (e.g. patient and family satisfaction[19,52,58,73,78], enhanced wellbeing[61], increased trust[19,43,52,61] and understanding[68,114]), including explicitly disclosing the uncertainty in predicting their prognosis[171]. Honest and explicit communication is evidenced in a range of disease contexts such as heart failure[89,166], respiratory disease[37], advanced kidney disease [109], COPD [107], cancer [CMOC16][16,172], and stroke [CMOC19][36,173]. Many, but not all[58,171], patients and families appreciate clear, truthful, and compassionate communication of prognosis, including discussion of uncertainty[43,151,153,173-175] as this can be pivotal to inform decision making [CMOC2, 6, 14][46,58,73] in respect of a patient's preferences, values, and end of life wishes[36,55,56,63,105,110,142,165].

For peer review only - http://bmjopen.bmj.com/site/about/guidelines.xhtml

The uncertainty involved in prognostication, and risk of being wrong, may prompt health professional avoidance[64,125,175], but often patients do not expect health professionals to know exactly how long someone has left to live[64,176]. Therefore, language recommended for acknowledging uncertainty, whilst being honest and informative, is to consider a range of possible scenarios [CMOC6], or to use indicative timeframes e.g. days, weeks, months or years[55,64,83,130,151,153,168,177] rather than exact timeframes[58,62,96,115,117,155]. Honesty about uncertainty in prognostic information does not mean clinicians necessarily declaring they 'don't know'[151,153], which may be detrimental to patient trust/confidence [6,51]. Rather, healthcare professionals can highlight the irreducible nature of prognostic uncertainty alongside a clear and tailored explanation [CMOC37][168], whilst framing information with phrases such as 'I think'[101,151,153,178] and qualifiers/hedging 'could' 'probably'[122,153]. It is often helpful for patients and families to understand how and why prognosis is uncertain and why it may be estimated as 'months rather than years'[64,130], based on evidence such as explaining their signs, symptoms, and deterioration in an accessible manner[62,151,153,168] without overestimation[58,110,117]. For example, the phrase 'sick enough to die' is cited as an example of how to communicate death as a possible or probable outcome[19,82]. Such communication and strategies should allow a clinician to explicitly state that a prognosis is uncertain without undermining their experience, training or role in the relationship [CMOC3][42,153]. However, explicit disclosure of prognostic uncertainty in isolation is not enough[6,55], this must be coupled with skilled delivery[61], sensitivity[52,54,82,94,122], and appropriate content of information[37] in order not to trigger negative effects such as false hope or damaging the relationship[62,64]. Clinicians should try to emphasise the value of supportive care available and non-abandonment[55,76,83,179], if possible, alongside acknowledging prognostic uncertainty and addressing the patient's and families emotions[146], to make it known that such care and support can be continued [CMOC9, CMOC11][52,83].

7. *Implicit or avoided disclosure of information and uncertainty*

Clinicians may not know what to say[121,164] and may therefore avoid disclosing prognostic information to patients and carers [CMOC21][43,63,64,108,125,140,152,174,175]. Two of the main reasons cited in the literature for such avoidance were prognostic uncertainty itself [CMOC27, 28][109,113,114,121,161] and fear of taking away hope[1,63,78,81,106,115,152,171,174,180]. This healthcare professional avoidance or tacit communication may take several forms in practice including expressing uncertainty as certainty [CMOC22, 31, 38][115,126] (e.g. to influence decision making) [101,110], denial[98], indirect or vague terms[74,126,181], excessive optimism[74,83,110] or avoidance of giving bad news [CMOC33][44,80,88], providing a lack of information (both quantity and quality)[144] or lack of clarity [CMOC30] [75,144], focussing on now or treatments[41] rather than the future[44,75,80], delaying the disclosure[39,63,80,89,109,140], or complete avoidance of the conversation altogether [126,181]. Unless such avoidance was motivated by patients' and carers' preferences, it tended to trigger negative outcomes[96] for

For peer review only - http://bmjopen.bmj.com/site/about/guidelines.xhtml

patients and/or families such as feeling ill prepared for the future [71,144]/not knowing what to expect[164], lack of advance care planning [CMOC34, 42][39,65], frustration[60,138,144] and anger[64], diminishing trust of clinicians[64,140], poorer satisfaction[53], and isolation[59,144]. Notably, clinician avoidance of talking about prognostic uncertainty may be driven by their own emotional discomfort, or fear of causing distress to patients[50,53,61,74,81,108-110,112,126,146,182].

8. *Emotions, hope and distress*

Hope was a central motivator affecting whether communication of prognostic uncertainty occurred and could have multiple and variable effects. A common contributing factor to clinician reluctance to disclose prognosis and prognostic uncertainty is desire to preserve hope, and fear of causing harm, anxiety, upset, or distress[43,53,61,81,96,108,110,112,114,146]. Conversely, some evidence[65,85] suggests that avoiding discussion of uncertainty can lead to false hope [CMOC 38], and also that patients *without* clear prognostic information and awareness may be 'more likely to experience anxiety'[79] and prognostic outcomes are not necessarily distressing[81,118] or depressing[78,79,114]; however, there is mixed and contrasting evidence on these outcomes, particularly around distress [63]. Furthermore, patients may be more harmed by the dishonesty of clinicians delaying, not asking or sharing their prognosis with them [CMOC24][53,65,78,98]; this can lead to mistrust in healthcare professionals[72] and depression as well as unresolved bereavement narratives[43,72,146]. Relatedly, another determinant of prognostic conversations is often clinician's concern of 'a loss of hope',[44,62,69,78,81,93,106,115] which can lead to introduction of prognostic uncertainty to promote (false) hope [CMOC33][58]. So long as patients want to engage in prognostic discussions, there is limited evidence that communicating uncertainty decreases hope[73,114] and indeed some may view uncertainty itself as 'a source of hope'[43,51,100] which may reduce anxiety[47]; therefore, clinician's fears of taking away hope may often be unfounded [CMOC13][73,96,114,174]. Despite this, there exists a paradox of patients and carers wanting to be informed, all the while fearing the future[1] and the emotional impact of "knowing"[130]; this is complex for clinicians to navigate and requires clarity, skill and compassion[72,78,100]. For example in Cagle et al. (2016)[62], one carer expressed the sentiment that, *"regardless of the potential to take away hope, she would want to know"* (p.7)[62].

Figure 3 presents a more detailed description of the theory , illustrating the links between individual CMOCs and thematic areas.

[INSERT FIGURE 3 HERE]

For peer review only - http://bmjopen.bmj.com/site/about/guidelines.xhtml

Discussion:

Statement of principle findings

The programme theory developed in this review characterises contexts and mechanisms that influence whether and how prognostic uncertainty is communicated between healthcare professionals, patients and carers, and the outcomes affected by such communication. We found that when communicating an uncertain prognosis, the degree of explicitness appears to influence shared understanding and trust between clinicians and patients/carers, impacting experiences of distress, acceptance, and future care planning. Key contextual factors including sociocultural norms and values, variation in individual communication preferences, and clinician tolerance of uncertainty shape how communication occurs. Although widely recognised as important, there was limited evidence on how various sociocultural factors affect communication.

Strengths and limitations

We reviewed extensive evidence including academic papers, guidelines and theses. We were therefore able to consider a range of advanced illness contexts: whilst many papers focused on malignancy, a variety of other illness and care contexts were included. The interdisciplinary research team and regular stakeholder input increased trustworthiness of the findings.

The stakeholder-informed realist approach involved patient and clinician perspectives throughout, alongside in-depth evidence synthesis. Stakeholders particularly contributed to generation of the programme theory, and to refining the CMOCs. Communication, prognosis and uncertainty are complex and nuanced concepts; interrogating them through realist synthesis enabled us to go beyond description to develop an explanatory theory[24].

In line with the realist approach, we aimed to synthesise sufficient literature to develop a programme theory rather than finding all existing relevant literature. The review was therefore not exhaustive. However, we are confident that our searches identified adequate literature to inform a robust theory. Despite the breadth of our search, which was not limited by language, most included articles originated from the USA, Europe and Australia, so perspectives from elsewhere, particularly from lower- and middle-income countries, could be under-represented. We were not able to search repositories of literature in other languages. We were not able to conduct full double screening of search results, but there was good agreement on the proportion that were double screened. It should also be noted that the included literature was in part of moderate or low quality, which limits the conclusions that can be drawn, and further empirical work to validate this theory may help to identify the most useful approaches to communication in specific contexts

For peer review only - http://bmjopen.bmj.com/site/about/guidelines.xhtml

Comparison with existing literature:

This is, to our knowledge, the first realist synthesis of evidence regarding communication of prognostic uncertainty. There is however an overlapping body of evidence on communication of prognosis, which identifies prognostic uncertainty as a problem in communication and care delivery[96,183]. We extend the evidence base by interrogating prognostic uncertainty itself and unveiling the underlying mechanisms affecting its communication.

Literature reviews on prognostic communication in cancer support our findings, including the need for knowledge and expertise about the patient, to individually tailor disclosure and establish individual, nuanced patient preferences[16]. Whilst the complex interplay between honesty, hope and uncertainty is known[16,75,184]; the theory presented here adds to existing evidence by explaining that relationship in the context of prognostic communication. Honesty and explicit yet sensitive disclosure of prognostic uncertainty may foster patient-clinician trust and, contrary to some clinicians' fears, leaves space for hope. Findings by Clayton et al [2008] that hope can be derived from many different sources in advanced illness contexts, indicate that accurate prognostic predictions are not patients' sole sources of hope[185]. Disclosure of prognostic uncertainty could therefore be reframed in a more nuanced way.

Work on prognostic disclosure indicates that sociocultural factors, such as cultural tendencies towards implicit disclosure and euphemism[186], (mis)perceptions that discussing death is (socially) a "taboo"[187], and paradigms of cultural collectivism[127] influence preferences and attitudes towards prognostic information[16,96,127].. The finding presented here add to knowledge by highlighting the particular importance of sociocultural factors in shaping attitudes to communication of prognostic *uncertainty*, albeit with limited supporting empirical evidence. The implication is that assumptions or generalisations based upon perceived cultural background should be avoided.

Meaning & implications

This programme theory provides a novel understanding of the contexts and mechanisms that support effective communication of prognostic uncertainty in serious illness and palliative care, demonstrating the complexity and nuance behind such conversations.

The theory presented here can support professionals in individualised and compassionate communication of prognostic uncertainty to reduce distress, enhance trust, and enable shared care planning. Whilst prognostic discussions are sometimes avoided or delayed until uncertainty is reduced, earlier and/or more transparent communication could improve decision making and timely access to services. Confidence can be a key limiting factor, so approaches that support healthcare professionals to develop confidence to engage with

For peer review only - http://bmjopen.bmj.com/site/about/guidelines.xhtml

prognostic uncertainty by reflecting on their own experience with and tolerance of uncertainty, will likely be of benefit. Supporting clinicians to understand and develop their tolerance of uncertainty may influence the occurrence and helpfulness of prognostic conversations. A standardised approach is unhelpful; flexibility and reflexivity could help professionals to communicate with sensitivity and respect for the individual and their sociocultural background.

Unanswered questions and future research

Future research should focus on translation of these insights into clinician training and practice guidelines, exploring how this programme theory may inform the design and evaluation of approaches to care that integrate prognostic uncertainty, such as parallel planning for multiple possible health outcomes. We found little evidence regarding written communication of prognostic uncertainty and further research here is needed.

To help clinicians develop individualised, and culturally appropriate approaches to prognostic communication, future implementation-focused empirical work should also explore how this theory applies in practice. In particular we should explore how sociocultural factors affect communication of prognostic uncertainty in different geographical, cultural, and health system contexts and how communication can be sensitively approached.

For peer review only

For peer review only - http://bmjopen.bmj.com/site/about/guidelines.xhtml

List of Appendices

1. RAMESES checklist
2. initial programme theory
3. search strategy
4. extraction proforma
5. details of included articles
6. Context Mechanism Outcome Configuration table

7. supplementary references

Acknowledgements

We would like to thank the patient and public involvement contributors as well as clinical stakeholders who provided their expertise and guidance for this review work, and helped us refine the programme theory.

Transparency:

The lead author affirms that the manuscript is an honest, accurate, and transparent account of the study being reported; that no important aspects of the study have been omitted; and that any discrepancies from the study as registered on Prospero have been explained.

Funding/conflicts of interest

This project was funded by Marie Curie (grant number MCSGS-23-201/ G763113)

The University of Birmingham supported Dr Weetman's time for drafting the manuscript.

Dr MacArtney is supported by a Marie Curie Fellowship (2023-26) ref: MCRFS-18-101, and has received funding from Marie Curie, NIHR, Australian Research Council for other projects in the past 36 months

Dr Etkind has received funding from Addenbrookes Charitable Trust, Marie Curie, Alborada Trust for other projects in the past 36 months

All authors have completed the Unified Competing Interest form (available on request from the corresponding author) and declare: no support from any organisation for the submitted work other than that described above; no financial relationships with any organisations that might have an interest in the submitted work in the previous three years other than that described above; no other relationships or activities that could appear to have influenced the submitted work.

For peer review only

Contributorship

The corresponding author attests that all listed authors meet authorship criteria and that no others meeting the criteria have been omitted

- Project conceptualisation and securing funding: JM, KW, SE
- Design and methodology FS, JM, KW, SE
- Searching, screening and data extraction, FS, KH, KW, SE, TA
- Synthesis KH, KW, SE, TA
- Writing of initial draft FS, KH, KW, SE, TA
- Review and approval of final manuscript FS, JM, KH, KW, SE, TA
- Dr Etkind is guarantor of this manuscript and its contents

For peer review only

For peer review only - http://bmjopen.bmj.com/site/about/guidelines.xhtml

References

1. Etkind SN, Koffman J. Approaches to managing uncertainty in people with life-limiting conditions: role of communication and palliative care. Postgrad Med J. 2016;92(1089):412-7.
2. Han PK, Klein WM, Arora NK. Varieties of Uncertainty in Health Care A Conceptual Taxonomy. Medical Decision Making. 2011;31(6):828-38.
3. Etkind SN, Li J, Louca J, Hopkins SA, Kuhn I, Spathis A, et al. Total Uncertainty: A systematic review and thematic synthesis of experiences of uncertainty in older people with advanced multimorbidity, their informal carers, and health professionals. . Age and Ageing 2022;51(8).
4. MacLeod RD. Certainty is an illusion: lessons for palliative care. Journal of the Royal Society of Medicine. 2024;117(5):161-4.
5. Etkind SN, Bristowe K, Bailey K, Selman LE, Murtagh FEM. How does uncertainty shape patient experience in advanced illness? A secondary analysis of qualitative data. Palliative Medicine. 2016;31(2):171-80.
6. Medendorp NM, Stiggelbout AM, Aalfs CM, Han PKJ, Smets EMA, Hillen MA. A scoping review of practice recommendations for clinicians' communication of uncertainty. Health Expect. 2021;24(4):1025-43.
7. Etkind SN, Barclay S, Spathis A, Hopkins SA, Bowers B, Koffman J. Uncertainty in serious illness: A national interdisciplinary consensus exercise to identify clinical research priorities. PLoS ONE. 2024;19(2):e0289522.
8. Kingston A, Robinson L, Booth H, Knapp M, Jagger C, for the Mp. Projections of multi-morbidity in the older population in England to 2035: estimates from the Population Ageing and Care Simulation (PACSim) model. Age and Ageing. 2018;47(3):374-80.
9. Murray SA, Boyd K, Moine S, Kendall M, Macpherson S, Mitchell G, et al. Using illness trajectories to inform person centred, advance care planning. BMJ (Clinical research ed). 2024;384:e067896.
10. Leach I, Mayland C, Turner N, Mitchell S. Understanding patient views and experiences of the IDENTIfication of PALLiative care needs (IDENTI-PALL): a qualitative interview study. British Journal of General Practice. 2023.
11. Tobin J, Rogers A, Winterburn I, Tullie S, Kalyanasundaram A, Kuhn I, et al. Hospice care access inequalities: a systematic review and narrative synthesis. BMJ supportive & palliative care. 2022;12(2):142-51.
12. Gold Standards Famework Proactive Identification Guidance [Available from: https://www.goldstandardsframework.org.uk/wp-content/uploads/2025/07/Proactive-Identification-Guidance.pdf.
13. Etkind SN, Bone AE, Lovell N, Gomes B, Evans CE, Murtagh FE, et al. How many people will need palliative care in 2040? Past trends, future projections and implications for services. BMC Medicine. 2017(15):102.
14. Marmot M. Health equity in England: the Marmot review 10 years on. BMJ (Clinical research ed). 2020;368.
15. Hui D, Paiva CE, Del Fabbro EG, Steer C, Naberhuis J, van de Wetering M, et al. Prognostication in advanced cancer: update and directions for future research. Support Care Cancer. 2019;27(6):1973-84.
16. Johnson M, Tod AM, Brummell S, Collins K. Prognostic communication in cancer: A critical interpretive synthesis of the literature. Eur J Oncol Nurs. 2015;19(5):554-67.

For peer review only - http://bmjopen.bmj.com/site/about/guidelines.xhtml

17. Simpkin AL, Armstrong KA. Communicating Uncertainty: a Narrative Review and Framework for Future Research. Journal of general internal medicine. 2019;34(11):2586-91.
18. Cox CL, Miller BM, Kuhn I, Fritz Z. Diagnostic uncertainty in primary care: what is known about its communication, and what are the associated ethical issues? Family Practice. 2021;38(5):654-68.
19. Krawczyk M, Gallagher R. Communicating prognostic uncertainty in potential end-of-life contexts: experiences of family members. BMC palliative care. 2016;15:59.
20. Clayton JM, Hancock KM, Butow PN, Tattersall MH, Currow DC, Adler J, et al. Clinical practice guidelines for communicating prognosis and end-of-life issues with adults in the advanced stages of a life-limiting illness, and their caregivers. Med J Aust. 2007;186(S12):S77-s105.
21. Broeder P. Informed communication in high context and low context cultures. Journal of Education, Innovation and Communication. 2021;3(1):13-24.
22. Hallenbeck J. High context illness and dying in a low context medical world. American Journal of Hospice and Palliative Medicine®. 2006;23(2):113-8.
23. Wong G, Greenhalgh T, Westhorp G, Buckingham J, Pawson R. RAMESES publication standards: realist syntheses. BMC Medicine. 2013;11(1):21.
24. Pawson R, Greenhalgh T, Harvey G, Walshe K. Realist synthesis-an introduction. ESRC res methods program. 2004;2:55.
25. Pawson R, Greenhalgh T, Harvey G, Walshe K. Realist review - a new method of systematic review designed for complex policy interventions. Journal of Health Services Research & Policy. 2005;10(1_suppl):21-34.
26. Murray SA, Kendall M, Boyd K, Sheikh A. Illness trajectories and palliative care. BMJ: British Medical Journal. 2005;330(7498):1007.
27. Weetman K, Wong G, Scott E, MacKenzie E, Schnurr S, Dale J. Improving best practice for patients receiving hospital discharge letters: a realist review. BMJ Open. 2019;9(6):e027588.
28. Coleman S, Wright JM, Nixon J, Schoonhoven L, Twiddy M, Greenhalgh J. Searching for Programme theories for a realist evaluation: a case study comparing an academic database search and a simple Google search. BMC medical research methodology. 2020;20(1):217.
29. Weetman K, Wong G, Scott E, Schnurr S, Dale J. Improving best practice for patients receiving hospital discharge letters: a realist review protocol. BMJ Open. 2017;7(11):e018353.
30. Booth A, Briscoe S, Wright JM. The "realist search": a systematic scoping review of current practice and reporting. Research synthesis methods. 2020;11(1):14-35.
31. Booth A, Wright J, Briscoe S. Scoping and searching to support realist approaches. Doing realist research. 2018:147-66.
32. Aali G, Shokraneh F. No limitations to language, date, publication type, and publication status in search step of systematic reviews. Journal of Clinical Epidemiology. 2021;133.
33. Lawson L, Cooper M, Tolley C, Hand A, Nazar H. Do hospital-to-home transitions work for older adults with multiple long-term conditions including dementia? A realist review. BMC Geriatrics. 2025;25(1):511.
34. Dada S, Dalkin S, Gilmore B, Hunter R, Mukumbang FC. Applying and reporting relevance, richness and rigour in realist evidence appraisals: Advancing key concepts in realist reviews. Research Synthesis Methods. 2023;14(3):504-14.

For peer review only - http://bmjopen.bmj.com/site/about/guidelines.xhtml

35. Pawson R. Digging for Nuggets: How ‘Bad’ Research Can Yield ‘Good’ Evidence. International Journal of Social Research Methodology. 2006;9(2):127-42.
36. Connolly T, Coats H, DeSanto K, Jones J. The experience of uncertainty for patients, families and healthcare providers in post-stroke palliative and end-of-life care: a qualitative meta-synthesis. Age Ageing. 2021;50(2):534-45.
37. Johnson RF, Jr., Gustin J. Acute lung injury and acute respiratory distress syndrome requiring tracheal intubation and mechanical ventilation in the intensive care unit: impact on managing uncertainty for patient-centered communication. The American journal of hospice & palliative care. 2013;30(6):569-75.
38. Combes S. Conversations on Living and Dying: Facilitating Advance Care Planning with Older People Living with Frailty. Developing and Refining an Intervention. England: University of London, King's College (United Kingdom); 2021.
39. Gough N, Ross JR, Riley J, Judson I, Koffman J. 'When something is this rare ... how do you know bad really is bad...?'-views on prognostic discussions from patients with advanced soft tissue sarcoma. BMJ Support Palliat Care. 2019;9(1):100-7.
40. Higginson IJ, Koffman J, Hopkins P, Prentice W, Burman R, Leonard S, et al. Development and evaluation of the feasibility and effects on staff, patients, and families of a new tool, the Psychosocial Assessment and Communication Evaluation (PACE), to improve communication and palliative care in intensive care and during clinical uncertainty. BMC medicine. 2013;11:1-14.
41. Gutierrez KM. Communication of prognostic information in an ICU at end of life: practices among and between nurses, physicians and family members: University of Minnesota; 2010.
42. Gutierrez KM. Prognostic categories and timing of negative prognostic communication from critical care physicians to family members at end-of-life in an intensive care unit. Nurs Inq. 2013;20(3):232-44.
43. Evans LR, Boyd EA, Malvar G, Apatira L, Luce JM, Lo B, et al. Surrogate decision-makers' perspectives on discussing prognosis in the face of uncertainty. Am J Respir Crit Care Med. 2009;179(1):48-53.
44. Barclay S, Momen N, Case-Upton S, Kuhn I, Smith E. End-of-life care conversations with heart failure patients: a systematic literature review and narrative synthesis. Br J Gen Pract. 2011;61(582):e49-62.
45. Carlini J, Bahudin D, Michaleff ZA, Plunkett E, She EN, Clark J, et al. Discordance and concordance on perception of quality care at end of life between older patients, caregivers and clinicians: a scoping review. Eur Geriatr Med. 2022;13(1):87-99.
46. Innes S, Payne S. Advanced cancer patients' prognostic information preferences: a review. Palliat Med. 2009;23(1):29-39.
47. Kimbell B, Boyd K, Kendall M, Iredale J, Murray SA. Managing uncertainty in advanced liver disease: a qualitative, multiperspective, serial interview study. BMJ Open. 2015;5(11):e009241.
48. Maxwell CA, Mixon AS, Conner E, Phillippi JC. Receptivity of Hospitalized Older Adults and Family Caregivers to Prognostic Information about Aging, Injury, and Frailty: A Qualitative Study. Int J Nurs Stud. 2020;109:103602.
49. Jackson VA, Emanuel L. Navigating and Communicating about Serious Illness and End of Life. The New England journal of medicine. 2024;390(1):63-9.
50. Momen NC, Barclay SI. Addressing 'the elephant on the table': barriers to end of life care conversations in heart failure - a literature review and narrative synthesis. Current opinion in supportive and palliative care. 2011;5(4):312-6.

For peer review only - http://bmjopen.bmj.com/site/about/guidelines.xhtml

51. Parvez S, Abdel-Kader K, Song MK, Unruh M. Conveying uncertainty in prognosis to patients with ESRD. Blood Purif. 2015;39(1-3):58-64.
52. Stanek MS, Norton SA. Expressions of Uncertainty from Patients with Advanced Cancer when Communicating with the Palliative Care Team. United States -- New York: University of Rochester; 2020.
53. Parker S, Clayton J, Hancock K, Walder S, Butow P, Carrick S, et al. Communicating Prognosis and Issues Surrounding End of Life (EOL) in adults in the advanced stages of a life-limiting illness: A Systematic Review. 2006.
54. Piovesan M, Orr P, Tevyaw S, Roussos E, Cherid C, Bouchard S. A Prospective Study with Patients and Families on the Usefulness of Accurate Prognosis for Palliative Care Patients. J Soc Work End Life Palliat Care. 2024;20(2):133-46.
55. Etkind SN, Fennessy R, Wang J, Simpson RM, O'Neill B, Barclay SIG, et al. Communicating prognostic uncertainties in advanced multimorbidity: a multimethod qualitative study to co-design practice recommendations. Eur Geriatr Med. 2025.
56. Nice. Care of dying adults in the last days of life. NICE. 2015.
57. McLennon SM, Uhrich M, Lasiter S, Chamness AR, Helft PR. Oncology nurses' narratives about ethical dilemmas and prognosis-related communication in advanced cancer patients. Cancer Nurs. 2013;36(2):114-21.
58. van Eenennaam RM, Kruithof WJ, van Es MA, Kruitwagen-van Reenen ET, Westeneng HJ, Visser-Meily JMA, et al. Discussing personalized prognosis in amyotrophic lateral sclerosis: development of a communication guide. BMC Neurol. 2020;20(1):446.
59. Fenton A, Fletcher KM, Kizildag D, Borstelmann NA, Kessler D, Cronin C, et al. Cancer Caregivers' Prognostic and End-of-Life Communication Needs and Experiences and their Impact. J Pain Symptom Manage. 2023;65(1):16-25.
60. Halkett GK, Lobb EA, Oldham L, Nowak AK. The information and support needs of patients diagnosed with High Grade Glioma. Patient Educ Couns. 2010;79(1):112-9.
61. Hui D, Mo L, Paiva CE. The Importance of Prognostication: Impact of Prognostic Predictions, Disclosures, Awareness, and Acceptance on Patient Outcomes. Curr Treat Options Oncol. 2021;22(2):12.
62. Cagle JG, McClymont KM, Thai JN, Smith AK. "If You Don't Know, All of a Sudden, They're Gone": Caregiver Perspectives About Prognostic Communication for Disabled Elderly Adults. J Am Geriatr Soc. 2016;64(6):1299-306.
63. Gray TF, Temel JS, El-Jawahri A. Illness and prognostic understanding in patients with hematologic malignancies. Blood Rev. 2021;45:100692.
64. Marie C. What’s stopping doctors having that conversation about death? Marie Curie. 2019.
65. Fallowfield LJ, Jenkins VA, Beveridge HA. Truth may hurt but deceit hurts more: communication in palliative care. Palliat Med. 2002;16(4):297-303.
66. Heyland DK, Allan DE, Rocker G, Dodek P, Pichora D, Gafni A. Discussing prognosis with patients and their families near the end of life: impact on satisfaction with end-of-life care. Open Med. 2009;3(2):e101-10.
67. van der Velden NCA, Han PKJ, van Laarhoven HWM, de Vos F, Hendriks LEL, Burgers SA, et al. Prevalence and Predictors of Physician-Patient Discordance in Prognostic Perceptions in Advanced Cancer. Oncologist. 2023;28(8):e653-e68.
68. Chen JJ, Roldan CS, Nichipor AN, Balboni TA, Krishnan MS, Revette AC, et al. Prognostic Understanding and Goals of Palliative Radiotherapy: A Qualitative Study. J Pain Symptom Manage. 2022;64(6):567-76.

For peer review only

For peer review only - http://bmjopen.bmj.com/site/about/guidelines.xhtml

69. Habib AR, Chen R, Magnavita ES, Jaung T, Awad MM, Odejide O, et al. Prevalence and Tolerance of Prognostic Uncertainty Among Thoracic Oncologists. Oncologist. 2021;26(8):e1480-e2.
70. Harrison M, Gardiner C, Taylor B, Ejegi-Memeh S, Darlison L. Understanding the palliative care needs and experiences of people with mesothelioma and their family carers: An integrative systematic review. Palliat Med. 2021;35(6):1039-51.
71. Cormican O, Dowling M. Providing Care to People Living with a Chronic Hematological Malignancy: A Qualitative Evidence Synthesis of Informal Carers' Experiences. Semin Oncol Nurs. 2022;38(6):151338.
72. Diggle MFJ, Schutz S, Butcher D. Bereaved Caregivers' Experiences of End of Life Care For People With Advanced Heart Failure: A Narrative Synthesis. Omega (Westport). 2025;90(4):1581-608.
73. Dickerson LK, Lipson TA, Chauhan SSB, Allen GI, Young B, Park JO, et al. Evaluating surgeon communication of pancreatic cancer prognosis using the VitalTalk ADAPT framework. J Surg Oncol. 2024;130(3):476-84.
74. Geerse OP, Lamas DJ, Sanders JJ, Paladino J, Kavanagh J, Henrich NJ, et al. A Qualitative Study of Serious Illness Conversations in Patients with Advanced Cancer. Journal of palliative medicine. 2019;22(7):773-81.
75. Hagerty RG, Butow PN, Ellis PM, Dimitry S, Tattersall MH. Communicating prognosis in cancer care: a systematic review of the literature. Ann Oncol. 2005;16(7):1005-53.
76. Henselmans I, Smets EMA, Han PKJ, de Haes H, Laarhoven H. How long do I have? Observational study on communication about life expectancy with advanced cancer patients. Patient Educ Couns. 2017;100(10):1820-7.
77. Hoff L, Hermeren G. Between uncertainty and certainty. The Journal of clinical ethics. 2011;22(2):139-50.
78. Holloway RG, Gramling R, Kelly AG. Estimating and communicating prognosis in advanced neurologic disease. Neurology. 2013;80(8):764-72.
79. Chan WC. Being aware of the prognosis: how does it relate to palliative care patients' anxiety and communication difficulty with family members in the Hong Kong Chinese context? Journal of palliative medicine. 2011;14(9):997-1003.
80. Stocker R, Close H, Hancock H, Hungin APS. Should heart failure be regarded as a terminal illness requiring palliative care? A study of heart failure patients', carers' and clinicians' understanding of heart failure prognosis and its management. BMJ Support Palliat Care. 2017;7(4):464-9.
81. Disler R, Pascoe A, Luckett T, Donesky D, Irving L, Currow DC, et al. Barriers to Palliative Care Referral and Advance Care Planning (ACP) for Patients With COPD: A Cross-Sectional Survey of Palliative Care Nurses. The American journal of hospice & palliative care. 2022;39(2):169-77.
82. British Geriatrics Society. End of Life Care in Frailty: Identification and prognostication. CLINICAL GUIDELINES. 2020.
83. Chu C, Anderson R, White N, Stone P. Prognosticating for Adult Patients With Advanced Incurable Cancer: a Needed Oncologist Skill. Curr Treat Options Oncol. 2020;21(1):5.
84. Hjelmfors L, Strömberg A, Friedrichsen M, Mårtensson J, Jaarsma T. Communication About the Heart Failure Trajectory in Patients, Their Families and Health Care Professionals. Sweden: Linkopings Universitet (Sweden); 2018.
85. Kimbell B. Living, dying and caring in advanced liver disease: the challenge of uncertainty. Scotland: The University of Edinburgh (United Kingdom); 2014.

For peer review only - http://bmjopen.bmj.com/site/about/guidelines.xhtml

86. Parker SM, Clayton JM, Hancock K, Walder S, Butow PN, Carrick S, et al. A systematic review of prognostic/end-of-life communication with adults in the advanced stages of a life-limiting illness: patient/caregiver preferences for the content, style, and timing of information. J Pain Symptom Manage. 2007;34(1):81-93.
87. Smith AK, Glare P. Ethical issues in prognosis and prognostication. The Oxford handbook of ethics at the end of life. 2014:170-89.
88. Marie Curie. Difficult conversations with dying people and their families. 2014 March 2014.
89. Stocker R. Experiences and perceptions of the role of palliative and end of life care in heart failure: a modified grounded theory study. England: University of Durham (United Kingdom); 2014.
90. Michael N, Shah R. Communication about Advanced Progressive Disease , Prognosis, and Advance Care Plans. In: Kissane DWMW, William B, editors. Psycho-Oncology in Palliative and End of Life Care. New York: Oxford Academic; 2022. p. 1-C.P207.
91. Nagelschmidt K, Leppin N, Seifart C, Rief W, von Blanckenburg P. Systematic mixed-method review of barriers to end-of-life communication in the family context. BMJ Support Palliat Care. 2021;11(3):253-63.
92. Chou WS, Hamel LM, Thai CL, Debono D, Chapman RA, Albrecht TL, et al. Discussing prognosis and treatment goals with patients with advanced cancer: A qualitative analysis of oncologists' language. Health Expect. 2017;20(5):1073-80.
93. Becker C, Beck K, Vincent A, Hunziker S. Communication challenges in end-of-life decisions. Swiss medical weekly. 2020;150:w20351.
94. Bristowe K, Carey I, Hopper A, Shouls S, Prentice W, Caulkin R, et al. Patient and carer experiences of clinical uncertainty and deterioration, in the face of limited reversibility: A comparative observational study of the AMBER care bundle. Palliat Med. 2015;29(9):797-807.
95. Ko E, Fuentes D, Singh-Carlson S, Nedjat-Haiem F. Challenges and facilitators of hospice decision-making: a retrospective review of family caregivers of home hospice patients in a rural US-Mexico border region-a qualitative study. BMJ Open. 2020;10(7):e035634.
96. Hancock K, Clayton JM, Parker SM, Wal der S, Butow PN, Carrick S, et al. Truth-telling in discussing prognosis in advanced life-limiting illnesses: a systematic review. Palliat Med. 2007;21(6):507-17.
97. Hendershot KA, Elias MN, Taylor BL, Wahlster S, Creutzfeldt CJ. An Update on Palliative Care in Neurocritical Care: Providing Goal-Concordant Care in the Face of Prognostic Uncertainty. Curr Treat Options Neurol. 2023;25(12):517-29.
98. Fisher M, Ridley S. Uncertainty in end-of-life care and shared decision making. Critical care and resuscitation : journal of the Australasian Academy of Critical Care Medicine. 2012;14(1):81-7.
99. Kantabanlang Y, Lin CP, Nilmanat K, Guo P. Palliative care needs of patients with hematologic malignancies and family caregivers and challenges of palliative care provision in Asia: A review of evidence. Semin Hematol. 2023;60(4):220-5.
100. Ridley S, Fisher M. Uncertainty in end-of-life care. Curr Opin Crit Care. 2013;19(6):642-7.
101. Schuster RA, Hong SY, Arnold RM, White DB. Do physicians disclose uncertainty when discussing prognosis in grave critical illness? Narrat Inq Bioeth. 2012;2(2):125-35.

For peer review only - http://bmjopen.bmj.com/site/about/guidelines.xhtml

102. Gofton TE, Chum M, Schulz V, Gofton BT, Sarpal A, Watling C. Challenges facing palliative neurology practice: A qualitative analysis. J Neurol Sci. 2018;385:225-31.
103. Broom A, Kirby E, Good P, Wootton J, Adams J. The troubles of telling: managing communication about the end of life. Qual Health Res. 2014;24(2):151-62.
104. Hudson BF, Shulman C, Low J, Hewett N, Daley J, Davis S, et al. Challenges to discussing palliative care with people experiencing homelessness: a qualitative study. BMJ Open. 2017;7(11):e017502.
105. Pooler C, Richman-Eisenstat J, Kalluri M. Early integrated palliative approach for idiopathic pulmonary fibrosis: A narrative study of bereaved caregivers' experiences. Palliat Med. 2018;32(9):1455-64.
106. Risco JR, Kelly AG, Holloway RG. Prognostication in neurology. Handb Clin Neurol. 2022;190:175-93.
107. Ngwenya N, Crang C, Farquhar M, Rintoul RC, Mahadeva R, Calvert LD, et al. Communicating uncertainty: contrasting the communication experiences of patients with advanced COPD and incurable lung cancer. Fam Pract. 2021;38(5):637-43.
108. Young J, Lyons A, Egan R, Dew K. Embodied decisions unfolding over time: a meta-ethnography systematic review of people with cancer's reasons for delaying or declining end-of-life care. BMC palliative care. 2024;23(1):45.
109. Mandel EI, Bernacki RE, Block SD. Serious Illness Conversations in ESRD. Clin J Am Soc Nephrol. 2017;12(5):854-63.
110. Dias LM, Barra W, Rego F. Advance Care Planning in Oncology: Challenges in Clinical Practice. Portugal: Universidade do Porto (Portugal); 2022.
111. Scholz B, Goncharov L, Emmerich N, Lu VN, Chapman M, Clark SJ, et al. Clinicians' accounts of communication with patients in end-of-life care contexts: A systematic review. Patient Educ Couns. 2020;103(10):1913-21.
112. Chen JJ, Gamble K, Graham-Wisener L, McGlade K, Doherty J, Donnelly P, et al. GP perceptions of the adequacy of community-based care for patients with advanced heart failure in a UK region (NI): a qualitative study. Open Heart. 2018;5(1):e000734.
113. Gerlach C, Goebel S, Weber S, Weber M, Sleeman KE. Space for intuition - the 'Surprise'-Question in haemato-oncology: Qualitative analysis of experiences and perceptions of haemato-oncologists. Palliat Med. 2019;33(5):531-40.
114. Habib AR, Cronin AM, Earle CC, Tulsky JA, Mack JW, Abel GA, et al. How Do Blood Cancer Doctors Discuss Prognosis? Findings from a National Survey of Hematologic Oncologists. Journal of palliative medicine. 2019;22(6):677-84.
115. Hallen SA, Hootsmans NA, Blaisdell L, Gutheil CM, Han PK. Physicians' perceptions of the value of prognostic models: the benefits and risks of prognostic confidence. Health Expect. 2015;18(6):2266-77.
116. Low J, Davis S, Vickerstaff V, Greenslade L, Hopkins K, Langford A, et al. Advanced chronic liver disease in the last year of life: a mixed methods study to understand how care in a specialist liver unit could be improved. BMJ Open. 2017;7(8):e016887.
117. Chu C, White N, Stone P. Prognostication in palliative care. Clin Med (Lond). 2019;19(4):306-10.
118. Pfeil TA, Laryionava K, Reiter-Theil S, Hiddemann W, Winkler EC. What keeps oncologists from addressing palliative care early on with incurable cancer patients? An active stance seems key. Oncologist. 2015;20(1):56-61.

For peer review only - http://bmjopen.bmj.com/site/about/guidelines.xhtml

119. Bloomer MJ, Botti M, Runacres F, Poon P, Barnfield J, Hutchinson AM. Communicating end-of-life care goals and decision-making among a multidisciplinary geriatric inpatient rehabilitation team: A qualitative descriptive study. Palliat Med. 2018;32(10):1615-23.
120. Masterson MP, Applebaum AJ, Buda K, Reisch S, Rosenfeld B. Don't Shoot the Messenger: Experiences of Delivering Prognostic Information in the Context of Advanced Cancer. The American journal of hospice & palliative care. 2018;35(12):1526-31.
121. Tavares NC. Palliative Care Communication in Copd : Patients' Preferences and Clinicians' Judgements. England: University of Southampton (United Kingdom); 2019.
122. Ekberg S, Parry R, Land V, Ekberg K, Pino M, Antaki C, et al. Communicating with patients and families about illness progression and end of life: a review of studies using direct observation of clinical practice. BMC palliative care. 2021;20(1):186.
123. Harrison M, Han PKJ, Rabin B, Bell M, Kay H, Spooner L, et al. Communicating uncertainty in cancer prognosis: a review of web-based prognostic tools. Patient education and counseling. 2019;102(5):842-9.
124. Reid C, Gibbins J, Bloor S, Burcombe M, McCoubrie R, Forbes K. Healthcare professionals' perspectives on delivering end-of-life care within acute hospital trusts: a qualitative study. BMJ Support Palliat Care. 2015;5(5):490-5.
125. Bailey SJ, Cogle K. Talking about dying: How to begin honest conversations about what lies ahead: Royal College of Physicians; 2018.
126. Epstein RM. Facing epistemic and complex uncertainty in serious illness: The role of mindfulness and shared mind. Patient Educ Couns. 2021;104(11):2635-42.
127. Pun J, Chow JCH, Fok L, Cheung KM. Role of patients' family members in end-of-life communication: an integrative review. BMJ Open. 2023;13(2):e067304.
128. Narayan M, Jones J, Portalupi LB, McIlvennan CK, Matlock DD, Allen LA. Patient Perspectives on Communication of Individualized Survival Estimates in Heart Failure. J Card Fail. 2017;23(4):272-7.
129. Owusuaa C, van Beelen I, van der Heide A, van der Rijt CCD. Physicians' views on the usefulness and feasibility of identifying and disclosing patients' last phase of life: a focus group study. BMJ Support Palliat Care. 2021;14(e2).
130. van der Velden NCA, van der Kleij MBA, Lehmann V, Smets EMA, Stouthard JML, Henselmans I, et al. Communication about Prognosis during Patient-Initiated Second Opinion Consultations in Advanced Cancer Care: An Observational Qualitative Analysis. Int J Environ Res Public Health. 2021;18(11).
131. Lee CW, Evans E, Vitous CA, Suwanabol PA. Living with uncertainty in surgery: integrating palliative care principles into conversations as a solution to patient and family, provider, and health system disquietude. Ann Palliat Med. 2023;12(1):16-20.
132. Goss AL, Voumard RR, Engelberg RA, Curtis JR, Creutzfeldt CJ. Do They Have a Choice? Surrogate Decision-Making After Severe Acute Brain Injury. Crit Care Med. 2023;51(7):924-35.
133. Goodlin SJ, Quill TE, Arnold RM. Communication and decision-making about prognosis in heart failure care. J Card Fail. 2008;14(2):106-13.
134. van der Velden NCA, Smets EMA, van Vliet LM, Brom L, van Laarhoven HWM, Henselmans I. Effects of prognostic communication strategies on emotions, coping, and appreciation of consultations: An experimental study in advanced cancer. Palliat Support Care. 2024:1-13.

For peer review only - http://bmjopen.bmj.com/site/about/guidelines.xhtml

135. van der Velden NCA, Smets EMA, van Vliet LM, Brom L, van Laarhoven HWM, Henselmans I. Effects of Prognostic Communication Strategies on Prognostic Perceptions, Treatment Decisions and End-Of-Life Anticipation in Advanced Cancer: An Experimental Study among Analogue Patients. J Pain Symptom Manage. 2024;67(6):478-89 e13.
136. Powers BA, Norton SA, Schmitt MH, Quill TE, Metzger M. Meaning and practice of palliative care for hospitalized older adults with life limiting illnesses. J Aging Res. 2011;2011:406164.
137. Cottrell L, Economos G, Evans C, Silber E, Burman R, Nicholas R, et al. A realist review of advance care planning for people with multiple sclerosis and their families. PLoS ONE. 2020;15(10):e0240815.
138. Crowther JL. Dementia: What Care Do Patients and Informal Carers Need in the Last Year of Life and Time Surrounding Death? An Exploratory Qualitative Study. England: The University of Liverpool (United Kingdom); 2011.
139. Mone S, Kerr H. Prognostic awareness in advanced cancer: an integrative literature review. BMJ Support Palliat Care. 2021;11(1):53-8.
140. Hoff L, Hermeren G. Identifying challenges to communicating with patients about their imminent death. The Journal of clinical ethics. 2014;25(4):296-306.
141. Olsson MM, Windsor C, Chambers S, Green TL. A Scoping Review of End-of-Life Communication in International Palliative Care Guidelines for Acute Care Settings. J Pain Symptom Manage. 2021;62(2):425-37 e2.
142. Udo C, Lovgren M, Lundquist G, Axelsson B. Palliative care physicians' experiences of end-of-life communication: A focus group study. Eur J Cancer Care (Engl). 2018;27(1).
143. Clarke G, Fistein E, Holland A, Tobin J, Barclay S, Barclay S. Planning for an uncertain future in progressive neurological disease: a qualitative study of patient and family decision-making with a focus on eating and drinking. BMC Neurol. 2018;18(1):115.
144. Fitzsimons D, Doherty LC, Murphy M, Dixon L, Donnelly P, McDonald K, et al. Inadequate Communication Exacerbates the Support Needs of Current and Bereaved Caregivers in Advanced Heart Failure and Impedes Shared Decision-making. J Cardiovasc Nurs. 2019;34(1):11-9.
145. Trankle SA, Shanmugam S, Lewis E, Nicholson M, Hillman K, Cardona M. Are We Making Progress on Communication with People Who Are Near the End of Life in the Australian Health System? A Thematic Analysis. Health Commun. 2020;35(2):158-67.
146. Robinson J, Pilbeam C, Goodwin H, Raphael D, Waterworth S, Gott M. The impact of uncertainty on bereaved family's experiences of care at the end of life: a thematic analysis of free text survey data. BMC palliative care. 2021;20(1):60.
147. Formagini T, Poague C, O'Neal A, Brooks JV. "When I Heard the Word Palliative": Obscuring and Clarifying Factors Affecting the Stigma Around Palliative Care Referral in Oncology. JCO Oncol Pract. 2022;18(1):e72-e9.
148. Green E, Gardiner C, Gott M, Ingleton C. Exploring the extent of communication surrounding transitions to palliative care in heart failure: the perspectives of health care professionals. Journal of palliative care. 2011;27(2):107-16.
149. Bloomer MJ, Botti M, Runacres F, Poon P, Barnfield J, Hutchinson AM. End-of-life care for older people in subacute care: A retrospective clinical audit. Collegian. 2019;26(1):22-7.

For peer review only - http://bmjopen.bmj.com/site/about/guidelines.xhtml

150. Bruun A, White N, Oostendorp L, Stone P, Bloch S. Time estimates in prognostic discussions: A conversation analytic study of hospice multidisciplinary team meetings. Palliat Med. 2024;38(5):593-601.
151. Anderson RJ. Communication with the Relatives and Friends of Imminently Dying Patients in a Hospice. England: University of London, University College London (United Kingdom); 2020.
152. Walczak A, Butow PN, Davidson PM, Bellemore FA, Tattersall MH, Clayton JM, et al. Patient perspectives regarding communication about prognosis and end-of-life issues: how can it be optimised? Patient Educ Couns. 2013;90(3):307-14.
153. Anderson RJ, Stone PC, Low JTS, Bloch S. Managing uncertainty and references to time in prognostic conversations with family members at the end of life: A conversation analytic study. Palliat Med. 2020;34(7):896-905.
154. Stiel H, Nagarajan SV, Forster BC, Clayton JM. Palliative care physicians' perspectives on transferring patients to nursing homes and communication strategies to facilitate this transition: A qualitative study. Palliat Med. 2019;33(3):323-31.
155. Moglia C, Palumbo F, Botto R, Iazzolino B, Ticozzi N, Calvo A, et al. Prognostic communication in amyotrophic lateral sclerosis: findings from a Nationwide Italian survey. Neurol Sci. 2024;45(12):5787-94.
156. Hjelmfors L, Stromberg A, Friedrichsen M, Martensson J, Jaarsma T. Communicating prognosis and end-of-life care to heart failure patients: a survey of heart failure nurses' perspectives. Eur J Cardiovasc Nurs. 2014;13(2):152-61.
157. Tuck KK, Brod L, Nutt J, Fromme EK. Preferences of patients with Parkinson's disease for communication about advanced care planning. The American journal of hospice & palliative care. 2015;32(1):68-77.
158. Gutierrez KM. Prognostic communication of critical care nurses and physicians at end of life. Dimens Crit Care Nurs. 2012;31(3):170-82.
159. Tavares N, Hunt KJ, Jarrett N, Wilkinson TM. The preferences of patients with chronic obstructive pulmonary disease are to discuss palliative care plans with familiar respiratory clinicians, but to delay conversations until their condition deteriorates: A study guided by interpretative phenomenological analysis. Palliat Med. 2020;34(10):1361-73.
160. Garland EL, Bruce A, Stajduhar K. Exposing barriers to end-of-life communication in heart failure: an integrative review. Canadian journal of cardiovascular nursing = Journal canadien en soins infirmiers cardio-vasculaires. 2013;23(1):12-8.
161. Chang A, Datta-Barua I, McLaughlin B, Daly B. A survey of prognosis discussions held by health-care providers who request palliative care consultation. Palliat Med. 2014;28(4):312-7.
162. Han PK, Dieckmann NF, Holt C, Gutheil C, Peters E. Factors Affecting Physicians' Intentions to Communicate Personalized Prognostic Information to Cancer Patients at the End of Life: An Experimental Vignette Study. Med Decis Making. 2016;36(6):703-13.
163. Chow J, Senderovich H. It's Time to Talk: Challenges in Providing Integrated Palliative Care in Advanced Congestive Heart Failure. A Narrative Review. Curr Cardiol Rev. 2018;14(2):128-37.
164. Nadicksbernd J. Why Do Community Healthcare Professionals Find Some Conversations Difficult? England: University of London, Royal Holloway College (United Kingdom); 2023.

For peer review only - http://bmjopen.bmj.com/site/about/guidelines.xhtml

165. Gonella S, Basso I, Dimonte V, Giulio PD. The role of end-of-life communication in contributing to palliative-oriented care at the end-of-life in nursing home. Int J Palliat Nurs. 2022;28(1):16-26.
166. Nice. Chronic heart failure in adults: diagnosis and management. NICE. 2018.
167. Marie C. Tips for talking to your patients about a prognosis. Marie Curie. 2020.
168. Anderson RJ, Stone PC, Low JTS, Bloch S. Transitioning out of prognostic talk in discussions with families of hospice patients at the end of life: A conversation analytic study. Patient Educ Couns. 2021;104(5):1075-85.
169. Szawarski P. Classic cases revisited: Oscar the cat and predicting death. J Intensive Care Soc. 2016;17(4):341-5.
170. Anstey S. Clinical thinking, knowledge and the referral practices of consultant physicians to specialist palliative care services: an interpretative phenomenological analysis. Wales: Cardiff University (United Kingdom); 2012.
171. Wijsenbeek MS, Bonella F, Orsatti L, Russell AM, Valenzuela C, Wuyts WA, et al. Communicating with patients with idiopathic pulmonary fibrosis: can we do it better? ERJ Open Res. 2022;8(1):00422-2021.
172. Temel JS, Shaw AT, Greer JA. Challenge of Prognostic Uncertainty in the Modern Era of Cancer Therapeutics. Journal of clinical oncology : official journal of the American Society of Clinical Oncology. 2016;34(30):3605-8.
173. Payne S, Burton C, Addington-Hall J, Jones A. End-of-life issues in acute stroke care: a qualitative study of the experiences and preferences of patients and families. Palliat Med. 2010;24(2):146-53.
174. Gadoud AC. A palliative care approach for people with advanced heart failure: recognition of need, transitions in care and impact on patients, family carers and clinicians. England: University of Hull (United Kingdom); 2013.
175. Tolsa L, Jones L, Michel P, Borasio GD, Jox RJ, Rutz Voumard R. 'We Have Guidelines, but We Can Also Be Artists': Neurologists Discuss Prognostic Uncertainty, Cognitive Biases, and Scoring Tools. Brain Sci. 2022;12(11):1591.
176. Richards N, Ingleton C, Gardiner C, Gott M. Awareness contexts revisited: indeterminacy in initiating discussions at the end-of-life. J Adv Nurs. 2013;69(12):2654-64.
177. Parry R. Communication in Palliative Care and About End of Life: A State-of-the-Art Literature Review of Conversation-Analytic Research in Healthcare. Research on Language and Social Interaction. 2024;57(1):127-48.
178. Wu Y, Zhang X. Examining Conversation Analysis in Palliative Care: A Systematic Review. Health Commun. 2024;39(13):3072-83.
179. van der Velden NCA, Meijers MC, Han PKJ, van Laarhoven HWM, Smets EMA, Henselmans I. The Effect of Prognostic Communication on Patient Outcomes in Palliative Cancer Care: a Systematic Review. Curr Treat Options Oncol. 2020;21(5):40.
180. Walczak A, Butow PN, Bu S, Clayton JM. A systematic review of evidence for end-of-life communication interventions: Who do they target, how are they structured and do they work? Patient Educ Couns. 2016;99(1):3-16.
181. Han PK. The Need for Uncertainty: A Case for Prognostic Silence. Perspect Biol Med. 2016;59(4):567-75.
182. Noble H, Brazil K, Burns A, Hallahan S, Normand C, Roderick P, et al. Clinician views of patient decisional conflict when deciding between dialysis and conservative management: Qualitative findings from the PAlliative Care in chronic Kidney diSease (PACKS) study. Palliat Med. 2017;31(10):921-31.

For peer review only - http://bmjopen.bmj.com/site/about/guidelines.xhtml

183. Tavares N, Jarrett N, Hunt K, Wilkinson T. Palliative and end-of-life care conversations in COPD: a systematic literature review. ERJ Open Res. 2017;3(2).
184. Anderson RJ, Bloch S, Armstrong M, Stone PC, Low JT. Communication between healthcare professionals and relatives of patients approaching the end-of-life: A systematic review of qualitative evidence. Palliat Med. 2019;33(8):926-41.
185. Clayton JM, Hancock K, Parker S, Butow PN, Walder S, Carrick S, et al. Sustaining hope when communicating with terminally ill patients and their families: a systematic review. Psychooncology. 2008;17(7):641-59.
186. Chung I, Khoo SY, Low LL. An Insight Into the Experiences of Malaysian Patients With Advanced Cancer and Their Preferences in End-of-Life Care: A Qualitative Study. The American journal of hospice & palliative care. 2025;42(1):5-13.
187. DeBoer RJ, Wabl CA, Mushi BP, Uwamahoro P, Athanas R, Ndoli DA, et al. A scoping review of clinical communication in cancer care in Africa. The Oncologist. 2025;30(4):oyaf039.

For peer review only

Figure Legends

Figure 1: PRISMA Chart

Figure 2. Final programme theory of how communication of prognostic uncertainty ‘works’, for whom, and in what contexts

Figure 3. Detailed programme theory

For peer review only - http://bmjopen.bmj.com/site/about/guidelines.xhtml

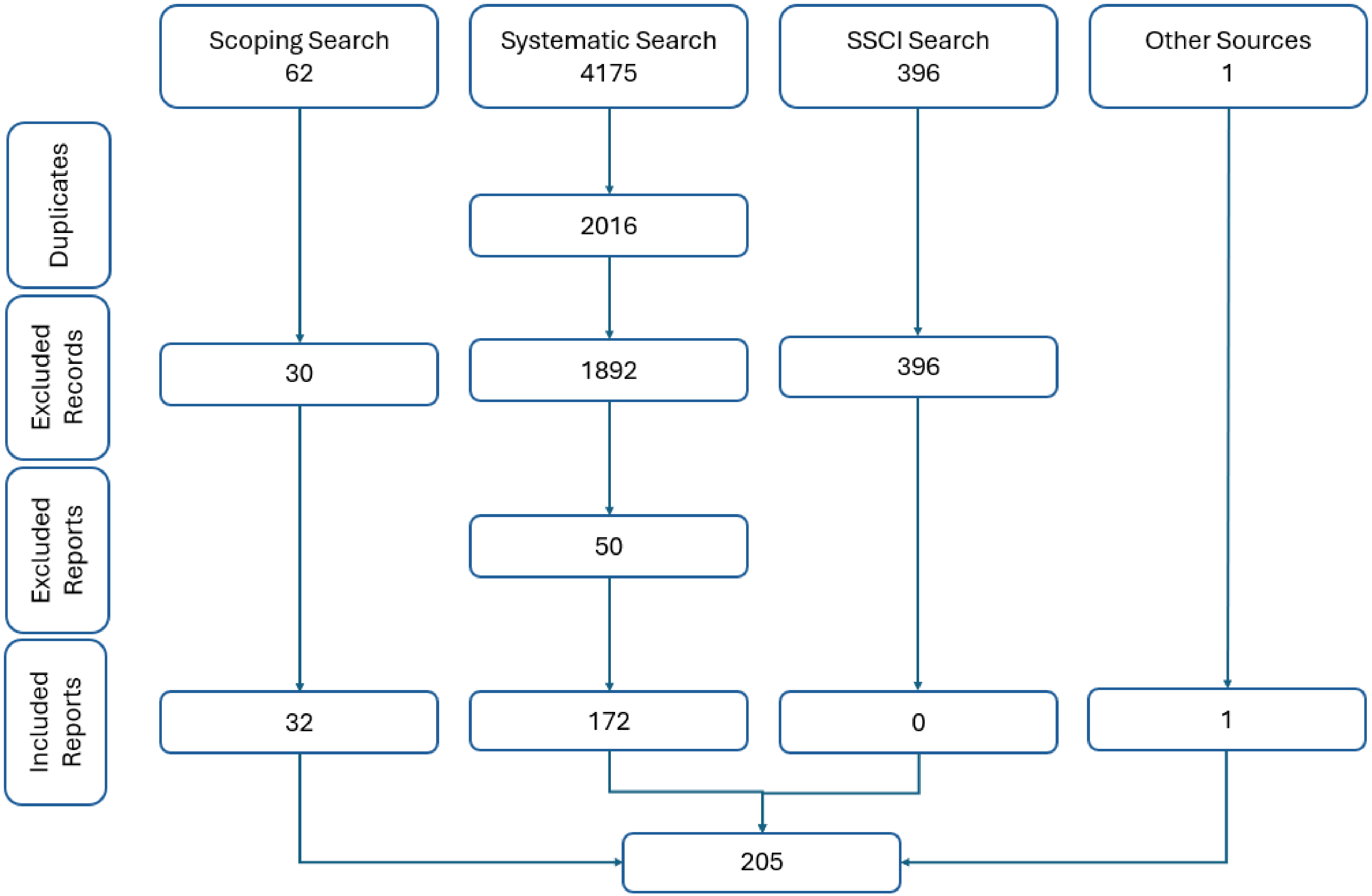
Scoping Search
62
Systematic Search
4175
SSCI Search
396
Other Sources
1
Duplicates
2016
Excluded Records
30
1892
396
Excluded Reports
50
Included Reports
32
172
0
1
205

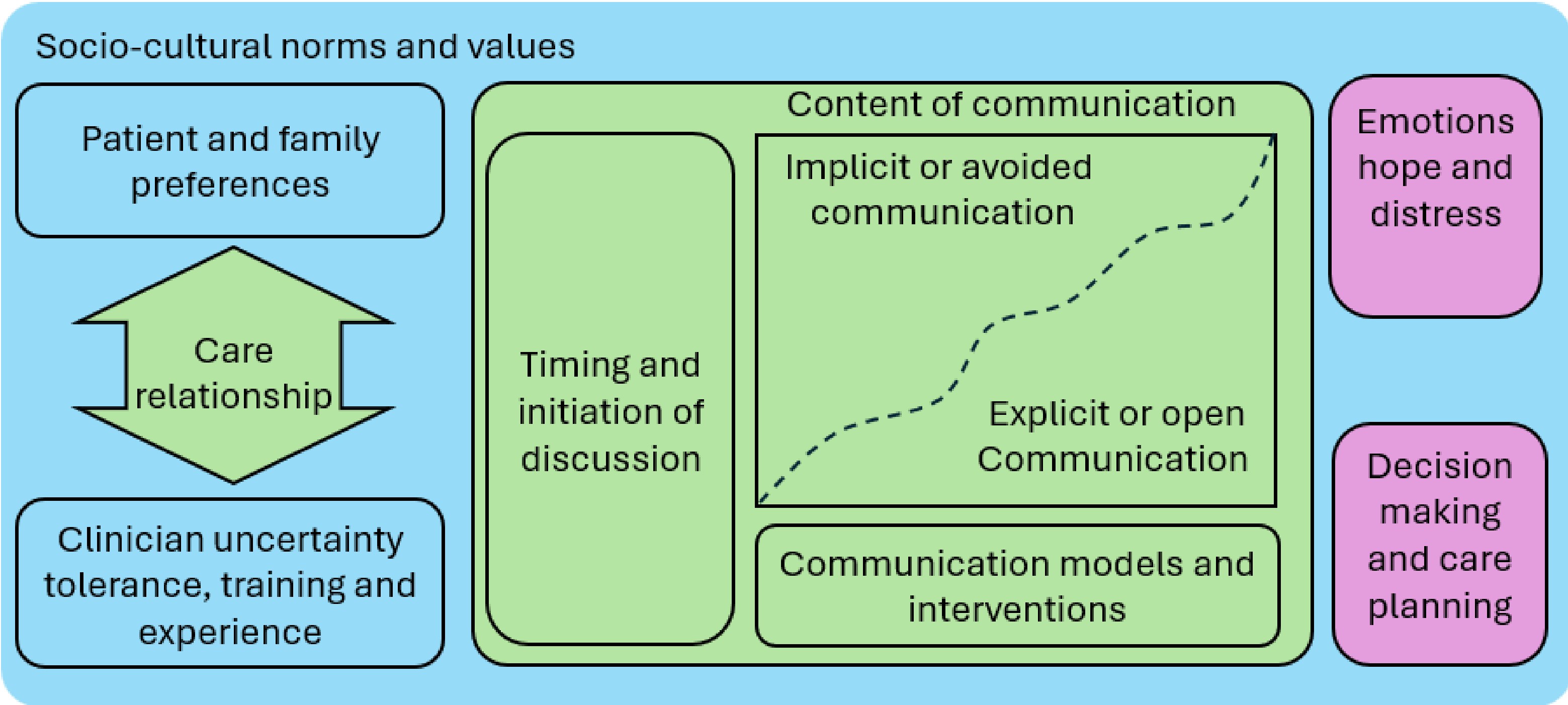
Socio-cultural norms and values
Patient and family preferences
Care relationship
Clinician uncertainty tolerance, training and experience
Content of communication
Timing and initiation of discussion
Implicit or avoided communication
Explicit or open Communication
Communication models and interventions
Emotions hope and distress
Decision making and care planning

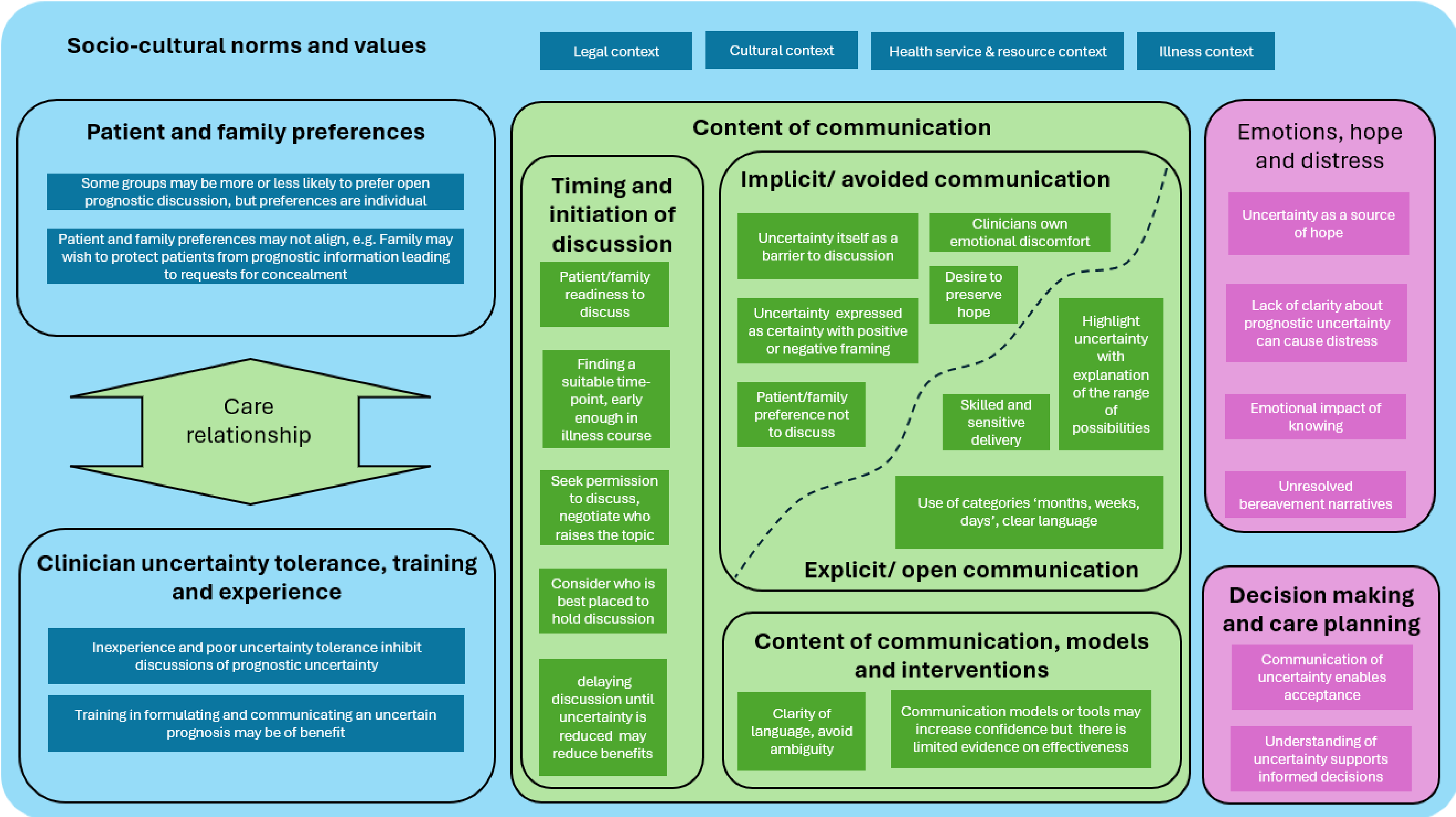
Socio-cultural norms and values
Legal context
Cultural context
Health service & resource context
Illness context
Patient and family preferences
Some groups may be more or less likely to prefer open prognostic discussion, but preferences are individual
Patient and family preferences may not align, e.g. Family may wish to protect patients from prognostic information leading to requests for concealment
Care relationship
Clinician uncertainty tolerance, training and experience
Inexperience and poor uncertainty tolerance inhibit discussions of prognostic uncertainty
Training in formulating and communicating an uncertain prognosis may be of benefit
Content of communication
Timing and initiation of discussion
Patient/family readiness to discuss
Finding a suitable time-point, early enough in illness course
Seek permission to discuss, negotiate who raises the topic
Consider who is best placed to hold discussion
delaying discussion until uncertainty is reduced may reduce benefits
Implicit/ avoided communication
Uncertainty itself as a barrier to discussion
Clinicians own emotional discomfort
Desire to preserve hope
Uncertainty expressed as certainty with positive or negative framing
Highlight uncertainty with explanation of the range of possibilities
Patient/family preference not to discuss
Skilled and sensitive delivery
Use of categories 'months, weeks, days', clear language
Explicit/ open communication
Content of communication, models and interventions
Clarity of language, avoid ambiguity
Communication models or tools may increase confidence but there is limited evidence on effectiveness
Emotions, hope and distress
Uncertainty as a source of hope
Lack of clarity about prognostic uncertainty can cause distress
Emotional impact of knowing
Unresolved bereavement narratives
Decision making and care planning
Communication of uncertainty enables acceptance
Understanding of uncertainty supports informed decisions

Appendix 1. Rameses reporting checklist

	Item	details	Reporting
TITLE			
1		In the title, identify the document as a realist synthesis or review	Title
ABSTRACT			
2		While acknowledging publication requirements and house style, abstracts should ideally contain brief details of: the study's background, review question or objectives; search strategy; methods of selection, appraisal, analysis and synthesis of sources; main results; and implications for practice.	Abstract
INTRODUCTION			
3	Rationale for review	Explain why the review is needed and what it is likely to contribute to existing understanding of the topic area.	Background P3
4	Objectives and focus of review	State the objective(s) of the review and/or the review question(s). Define and provide a rationale for the focus of the review.	P3
METHODS			

For peer review only - http://bmjopen.bmj.com/site/about/guidelines.xhtml

	Item	details	Reporting
5	Changes in the review process	Any changes made to the review process that was initially planned should be briefly described and justified.	n/a
6	Rationale for using realist synthesis	Explain why realist synthesis was considered the most appropriate method to use.	P4
7	Scoping the literature	Describe and justify the initial process of exploratory scoping of the literature.	P4-5
8	Searching processes	While considering specific requirements of the journal or other publication outlet, state and provide a rationale for how the iterative searching was done. Provide details on all the sources accessed for information in the review. Where searching in electronic databases has taken place, the details should include, for example, name of database, search terms, dates of coverage and date last searched. If individuals familiar with the relevant literature and/or topic area were contacted, indicate how they were identified and selected.	P5
9	Selection and appraisal of documents	Explain how judgements were made about including and excluding data from documents, and justify these.	P5

For peer review only - http://bmjopen.bmj.com/site/about/guidelines.xhtml

	Item	details	Reporting
10	Data extraction	Describe and explain which data or information were extracted from the included documents and justify this selection.	P5
11	Analysis and synthesis processes	Describe the analysis and synthesis processes in detail. This section should include information on the constructs analyzed and describe the analytic process.	P5-6
RESULTS			
12	Document flow diagram	Provide details on the number of documents assessed for eligibility and included in the review with reasons for exclusion at each stage as well as an indication of their source of origin (for example, from searching databases, reference lists and so on). You may consider using the example templates (which are likely to need modification to suit the data) that are provided.	Figure 1
13	Document characteristics	Provide information on the characteristics of the documents included in the review.	P7, supplementary information
14	Main findings	Present the key findings with a specific focus on theory building and testing.	P7-16
DISCUSSION			

For peer review only - http://bmjopen.bmj.com/site/about/guidelines.xhtml

	Item	details	Reporting
15	Summary of findings	Summarize the main findings, taking into account the review's objective(s), research question(s), focus and intended audience(s).	P17
16	Strengths, limitations and future research directions	Discuss both the strengths of the review and its limitations. These should include (but need not be restricted to) (a) consideration of all the steps in the review process and (b) comment on the overall strength of evidence supporting the explanatory insights which emerged.<br>The limitations identified may point to areas where further work is needed.	P17
17	Comparison with existing literature	Where applicable, compare and contrast the review's findings with the existing literature (for example, other reviews) on the same topic.	P17-18
18	Conclusion and recommendations	List the main implications of the findings and place these in the context of other relevant literature. If appropriate, offer recommendations for policy and practice.	P18-19
19	Funding	Provide details of funding source (if any) for the review, the role played by the funder (if any) and any conflicts of interests of the reviewers.	P20

For peer review only - http://bmjopen.bmj.com/site/about/guidelines.xhtml

Appendix 2. Initial programme theory

- Initial iterations of the draft theory (figure a) focused mainly on the clinical interaction itself and its outcomes.
- Discussions within the team and with the advisory group led to a broader lens, with addition of more contextual factors, particularly around family experiences, clinician factors, and the wider social context (figure b)

Figure a) early draft of initial theory

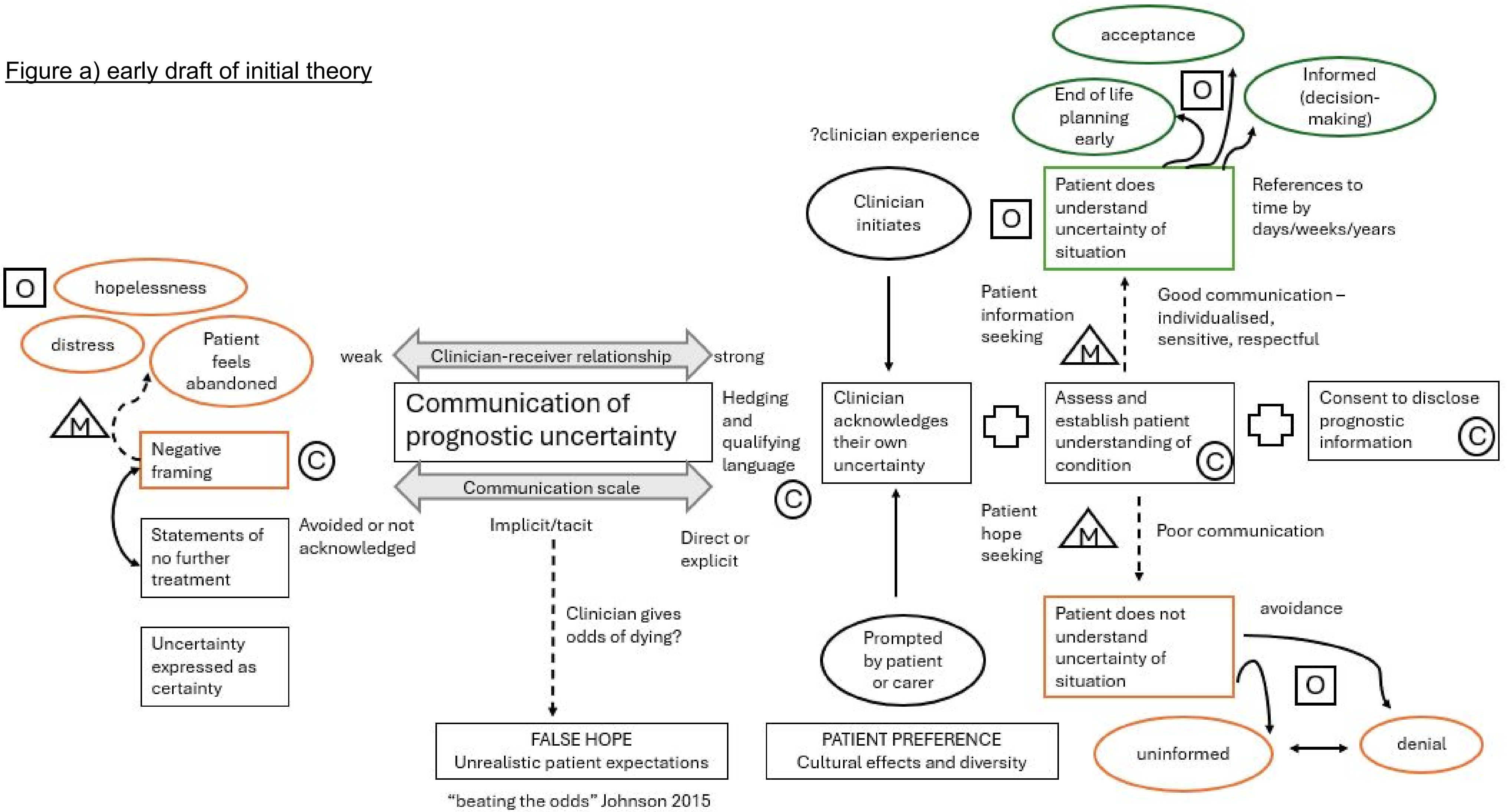

Figure b) agreed initial programme theory

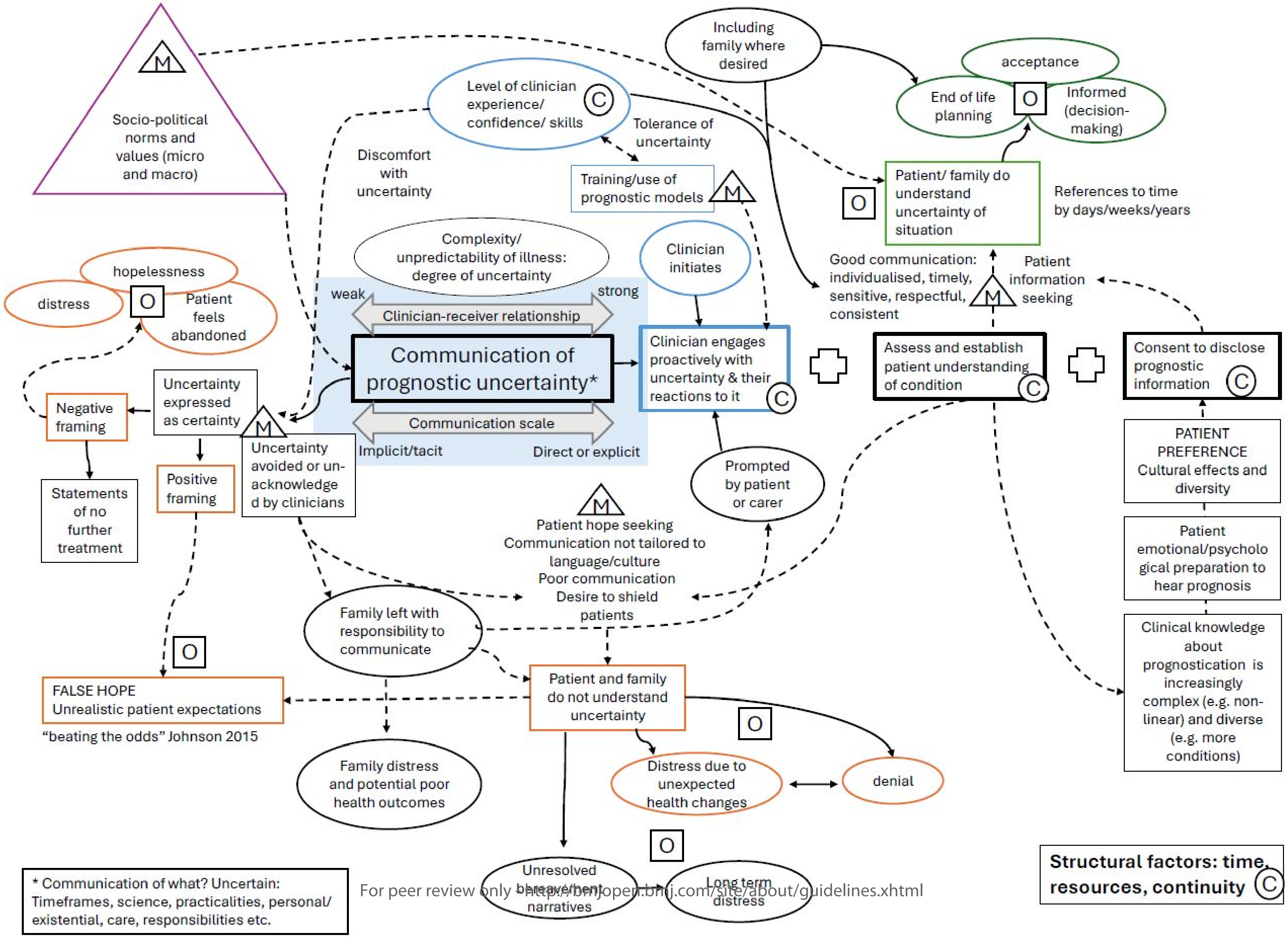

For peer review only - http://bmjopen.bmj.com/site/about/guidelines.xhtml

MEDLINE via Ovid SP

Database: Ovid MEDLINE(R) ALL <1946 to August 15, 2024>

1 Communication Barriers/ or Health Communication/ or Nonverbal Communication/ or Teach-Back Communication/ or Communication/ or Disclosure/ or Truth Disclosure/ or Interpersonal Relations/ or Professional-Patient Relations/ or Nurse-Patient Relations/ or Physician-Patient Relations/ or Patient Discharge Summaries/ or (Communicat* or Conversation* or Discussing or Discussed or Discuss or Discusses or Disclos* or Dialog* or Nurse-Patient Relation* or Patient-Nurse Relation* or Doctor-Patient Relation* or Physician-Patient Relation* or Patient-Doctor Relation* or Patient-Physician Relation* or Patient-Health Care Professional Relation* or Patient-Health Professional Relation* or Patient-Health Professional Relation* or Patient-Healthcare Professional Relation* or Patient-Professional Relation* or Professional-Patient Relation* or Interpersonal or Patient Discharge Summar* or Patient Discharge Letter*).ti,ab. (2281379)

2 Prognosis/ or Life Expectancy/ or Disease Progression/ or Clinical Deterioration/ or (Prognos* or Predict* or Forecast* or Life Expectanc* or Disease Progress* or Disease Exacerbat* or Deteriorat*).ti,ab. (3456789)

3 Uncertainty/ or (Uncertain* or Indetermin* or Ambigu* or Unpredictab* or Vague* or Doubt*).ti,ab. (395891)

4 Palliative Care/ or Palliative Medicine/ or Hospice Care/ or Hospices/ or "Hospice and Palliative Care Nursing"/ or Terminal Care/ or Death/ or Bereavement/ or (Palliat* or Hospice* or Bereav* or Terminal* or End-of-Life or Death* or Advanced Illness* or Advanced Disease* or Life-Limiting or Die or Dying or Bereav*).ti,ab. (1810528)

5 and/1-4 (920)

Embase via Ovid SP

Database: Embase <1974 to 2024 August 16>

1 Interpersonal Communication/ or Communication Skill/ or Communication Barrier/ or Nonverbal Communication/ or Verbal Communication/ or Conversation/ or Written Communication/ or Professional-Patient Relationship/ or Nurse Patient Relationship/ or Doctor Patient Relationship/ or Patient Centered Communication/ or (Communicat* or Conversation* or Discussing or Discussed or Discuss or Discusses or Disclos* or Dialog* or Nurse-Patient Relation* or Patient-Nurse Relation* or Doctor-Patient Relation* or Physician-Patient Relation* or Patient-Doctor Relation* or Patient-Physician Relation* or Patient-Health Care Professional Relation* or Patient-Health Professional Relation* or Patient-Health Professional Relation* or Patient-Healthcare Professional Relation* or Patient-Professional Relation* or Professional-Patient Relation* or Interpersonal or Patient Discharge Summar* or Patient Discharge Letter*).ti,ab. (2667971)

2 Prognosis/ or Life Expectancy/ or Disease Exacerbation/ or Deterioration/ or (Prognos* or Predict* or Forecast* or Life Expectanc* or Disease Progress* or Disease Exacerbat* or Deteriorat* or Disease Aggravat*).ti,ab. (4642834)

3 Uncertainty/ or Intolerance of Uncertainty Scale/ or Mishel Uncertainty in Illness Scale/ or Mishel Uncertainty in Illness Theory/ or (Uncertain* or Indetermin* or Ambigu* or Unpredictab* or Vague* or Doubt* or Mishel*).ti,ab. (519031)

For peer review only

4 exp Palliative Therapy/ or Palliative Nursing/ or Hospice Care/ or Hospice/ or Hospice Patient/ or Hospice Nursing/ or Terminal Care/ or Terminally Ill Patient/ or exp Terminal Disease/ or Death/ or Dying/ or Bereavement/ or (Palliat* or Hospice* or Bereav* or Terminal* or End-of-Life or Death* or Advanced Illness* or Advanced Disease* or Life-Limiting or Die or Dying or Bereav*).ti,ab. (2504665)

5 and/1-4 (1753)

6 limit 5 to embase (764)

PsycINFO via Ovid SP

Database: APA PsycInfo <1806 to August 2024 Week 3>

1 Communication/ or Communication Barriers/ or Nonverbal Communication/ or Verbal Communication/ or Conversation/ or Written Communication/ or Communication Skills/ or Communication Skills Training/ or Interpersonal Interaction/ or Interpersonal Communication/ or (Communicat* or Conversation* or Discussing or Discussed or Discuss or Discusses or Disclos* or Dialog* or Nurse-Patient Relation* or Patient-Nurse Relation* or Doctor-Patient Relation* or Physician-Patient Relation* or Patient-Doctor Relation* or Patient-Physician Relation* or Patient-Health Care Professional Relation* or Patient-Health Professional Relation* or Patient-Health Professional Relation* or Patient-Healthcare Professional Relation* or Patient-Professional Relation* or Professional-Patient Relation* or Interpersonal or Patient Discharge Summar* or Patient Discharge Letter*).ti,ab. (1205127)

2 Prognosis/ or Life Expectancy/ or Disease Progression/ or (Prognos* or Predict* or Forecast* or Life Expectanc* or Disease Progress* or Disease Exacerbat* or Deteriorat*).ti,ab. (619978)

3 exp Uncertainty/ or "Ambiguity (Non-Stimulus)"/ or Doubt/ or (Uncertain* or Indetermin* or Ambigu* or Unpredictab* or Vague* or Doubt*).ti,ab. (130464)

4 Palliative Care/ or Hospice/ or Terminally Ill Patients/ or Terminal Cancer/ or "Death and Dying"/ or Bereavement/ or (Palliat* or Hospice* or Bereav* or Terminal* or End-of-Life or Death* or Advanced Illness* or Advanced Disease* or Life-Limiting or Die or Dying or Bereav*).ti,ab. (186761)

5 and/1-4 (261)

CINAHL via EBSCOhost

Limiters - Exclude MEDLINE records

(((MH "Communication") OR (MH "Communication Barriers") OR (MH "Communication Skills") OR (MH "Communication Skills Training") OR (MH "Conversation") OR (MH "Nonverbal Communication") OR (MH "Truth Disclosure") OR (MH "Professional-Patient Relations") OR (MH "Physician-Patient Relations") OR (MH "Nurse-Patient Relations") OR (MH "Interpersonal Relations") OR (MH "Patient Discharge Summaries")) OR TI (Communicat* OR Conversation* OR Discussing OR Discussed OR Discuss OR Discusses OR Disclos* OR Dialog* OR Nurse-Patient Relation* OR Patient-Nurse Relation* OR Doctor-Patient Relation* OR Physician-Patient Relation* OR Patient-Doctor Relation* OR Patient-Physician Relation* OR Patient-Health Care Professional Relation* OR Patient-Health Professional Relation* OR Patient-Health Professional Relation* OR Patient-Healthcare Professional Relation* OR Patient-Professional Relation* OR Professional-Patient Relation* OR Interpersonal OR Patient Discharge Summar* OR Patient Discharge Letter*) OR AB (

Communicat* OR Conversation* OR Discussing OR Discussed OR Discuss OR Discusses OR Disclos* OR Dialog* OR Nurse-Patient Relation* OR Patient-Nurse Relation* OR Doctor-Patient Relation* OR Physician-Patient Relation* OR Patient-Doctor Relation* OR Patient-Physician Relation* OR Patient-Health Care Professional Relation* OR Patient-Health Professional Relation* OR Patient-Health Professional Relation* OR Patient-Healthcare Professional Relation* OR Patient-Professional Relation* OR Professional-Patient Relation* OR Interpersonal OR Patient Discharge Summar* OR Patient Discharge Letter*)) AND (((MH "Prognosis") OR (MH "Life Expectancy") OR (MH "Disease Progression") OR (MH "Clinical Deterioration")) OR TI (Prognos* OR Predict* OR Forecast* OR Life Expectanc* OR Disease Progress* OR Disease Exacerbat* OR Deteriorat*) OR AB (Prognos* OR Predict* OR Forecast* OR Life Expectanc* OR Disease Progress* OR Disease Exacerbat* OR Deteriorat*)) AND (((MH "Uncertainty") OR (MH "Mishel Uncertainty in Illness Theory") OR (MH "Mishel Uncertainty in Illness Scale")) OR TI (Uncertain* OR Indetermin* OR Ambigu* OR Unpredictab* OR Vague* OR Doubt*) OR AB (Uncertain* OR Indetermin* OR Ambigu* OR Unpredictab* OR Vague* OR Doubt*)) AND (((MH "Palliative Care") OR (MH "Palliative Care Nurses") OR (MH "Palliative Care Nursing") OR (MH "Palliative Medicine") OR (MH "Hospice Nurses") OR (MH "Hospice Patients") OR (MH "Hospice Nursing") OR (MH "Hospice Care") OR (MH "Hospices") OR (MH "National Association for Home Care & Hospice") OR (MH "Hospice and Palliative Nurses Association") OR (MH "Hospice (Saba CCC)") OR (MH "Terminal Care") OR (MH "Terminal Care (Saba CCC)") OR (MH "Terminally Ill Patients") OR (MH "Death") OR (MH "Bereavement")) OR TI (Palliat* OR Hospice* OR Bereav* OR Terminal* OR End-of-Life OR Death* OR Advanced Illness* OR Advanced Disease* OR Life-Limiting OR Die OR Dying OR Bereav*) OR AB (Palliat* OR Hospice* OR Bereav* OR Terminal* OR End-of-Life OR Death* OR Advanced Illness* OR Advanced Disease* OR Life-Limiting OR Die OR Dying OR Bereav*))

214

Cochrane Database of Systematic Reviews

Issue 8 of 12, August 2024

Date Run: 18/08/2024 22:03:17

([mh "Communication Barriers"] or [mh "Health Communication"] or [mh "Nonverbal Communication"] or [mh "Teach-Back Communication"] or [mh Communication] or [mh Disclosure] or [mh "Truth Disclosure"] or [mh "Interpersonal Relations"] or [mh "Professional-Patient Relations"] or [mh "Nurse-Patient Relations"] or [mh "Physician-Patient Relations"] or [mh "Patient Discharge Summaries"] or (Communicat* or Conversation* or Discussing or Discussed or Discuss or Discusses or Disclos* or Dialog* or Nurse-Patient Relation* or Patient-Nurse Relation* or Doctor-Patient Relation* or Physician-Patient Relation* or Patient-Doctor Relation* or Patient-Physician Relation* or Patient-Health Care Professional Relation* or Patient-Health Professional Relation* or Patient-Health Professional Relation* or Patient-Healthcare Professional Relation* or Patient-Professional Relation* or Professional-Patient Relation* or Interpersonal or Patient Discharge Summar* or Patient Discharge Letter*):ti,ab) AND ([mh Prognosis] or [mh "Life Expectancy"] or [mh "Disease Progression"] or [mh "Clinical Deterioration"] or (Prognos* or Predict* or Forecast* or Life Expectanc* or Disease Progress* or Disease Exacerbat* or Deteriorat*):ti,ab) AND ([mh Uncertainty] or (Uncertain* or Indetermin* or Ambigu* or Unpredictab* or Vague* or Doubt*):ti,ab) AND ([mh "Palliative Care"] or [mh "Palliative Medicine"] or [mh "Hospice Care"] or [mh Hospices] or [mh "Hospice and Palliative Care Nursing"] or [mh "Terminal Care"] or [mh Death] or [mh Bereavement] or (Palliat* or Hospice* or Bereav* or Terminal* or End-of-Life or Death* or Advanced Illness* or Advanced Disease* or Life-Limiting or Die or Dying or Bereav*):ti,ab)

For peer review only - http://bmjopen.bmj.com/site/about/guidelines.xhtml

25

ProQuest Dissertations & Theses Global

18 August 2024 15:09

S1 title(Communicat* or Conversation* or Discussing or Discussed or Discuss or Discusses or Disclos* or Dialog* or Nurse-Patient Relation* or Patient-Nurse Relation* or Doctor-Patient Relation* or Physician-Patient Relation* or Patient-Doctor Relation* or Patient-Physician Relation* or Patient-Health Care Professional Relation* or Patient-Health Professional Relation* or Patient-Health Professional Relation* or Patient-Healthcare Professional Relation* or Patient-Professional Relation* or Professional-Patient Relation* or Interpersonal or Patient Discharge Summar* or Patient Discharge Letter*) OR abstract(Communicat* or Conversation* or Discussing or Discussed or Discuss or Discusses or Disclos* or Dialog* or Nurse-Patient Relation* or Patient-Nurse Relation* or Doctor-Patient Relation* or Physician-Patient Relation* or Patient-Doctor Relation* or Patient-Physician Relation* or Patient-Health Care Professional Relation* or Patient-Health Professional Relation* or Patient-Health Professional Relation* or Patient-Healthcare Professional Relation* or Patient-Professional Relation* or Professional-Patient Relation* or Interpersonal or Patient Discharge Summar* or Patient Discharge Letter*) 11884219

S2 title(Prognos* or Predict* or Forecast* or Life Expectanc* or Disease Progress* or Disease Exacerbat* or Deteriorat*) OR abstract(Prognos* or Predict* or Forecast* or Life Expectanc* or Disease Progress* or Disease Exacerbat* or Deteriorat*) 7103093

S3 title(Uncertain* or Indetermin* or Ambigu* or Unpredictab* or Vague* or Doubt*) OR abstract(Uncertain* or Indetermin* or Ambigu* or Unpredictab* or Vague* or Doubt*) 2471013

S4 title(Palliat* or Hospice* or Bereav* or Terminal* or End-of-Life or Death* or Advanced Illness* or Advanced Disease* or Life-Limiting or Die or Dying or Bereav*) OR abstract(Palliat* or Hospice* or Bereav* or Terminal* or End-of-Life or Death* or Advanced Illness* or Advanced Disease* or Life-Limiting or Die or Dying or Bereav*) 14366671

S5 [S1] AND [S2] AND [S3] AND [S4] 319

Science Citation Index-Expanded via Web of Science

Web of Science Search Strategy (v0.1)

1: Communicat* or Conversation* or Discussing or Discussed or Discuss or Discusses or Disclos* or Dialog* or Nurse-Patient Relation* or Patient-Nurse Relation* or Doctor-Patient Relation* or Physician-Patient Relation* or Patient-Doctor Relation* or Patient-Physician Relation* or Patient-Health Care Professional Relation* or Patient-Health Professional Relation* or Patient-Health Professional Relation* or Patient-Healthcare Professional Relation* or Patient-Professional Relation* or Professional-Patient Relation* or Interpersonal or Patient Discharge Summar* or Patient Discharge Letter* (Title) OR Communicat* or Conversation* or Discussing or Discussed or Discuss or Discusses or Disclos* or Dialog* or Nurse-Patient Relation* or Patient-Nurse Relation* or Doctor-Patient Relation* or Physician-Patient Relation* or Patient-Doctor Relation* or Patient-Physician Relation* or Patient-Health Care Professional Relation* or Patient-Health Professional Relation* or Patient-Health Professional Relation* or Patient-Healthcare Professional Relation* or Patient-Professional Relation* or Professional-Patient Relation* or Interpersonal or Patient Discharge Summar* or Patient Discharge Letter* (Abstract) Editions: WOS.SCI Date Run: Sun Aug 18 2024 16:16:01 GMT+0100 (British Summer Time) Results: 3442984

For peer review only

2: Prognos* or Predict* or Forecast* or Life Expectanc* or Disease Progress* or Disease Exacerbat* or Deteriorat* (Title) OR Prognos* or Predict* or Forecast* or Life Expectanc* or Disease Progress* or Disease Exacerbat* or Deteriorat* (Abstract) Editions: WOS.SCI Date Run: Sun Aug 18 2024 16:16:28 GMT+0100 (British Summer Time) Results: 4892944

3: Uncertain* or Indetermin* or Ambigu* or Unpredictab* or Vague* or Doubt* (Title) OR Uncertain* or Indetermin* or Ambigu* or Unpredictab* or Vague* or Doubt* (Abstract) Editions: WOS.SCI Date Run: Sun Aug 18 2024 16:17:05 GMT+0100 (British Summer Time) Results: 792328

4: Palliat* or Hospice* or Bereav* or Terminal* or End-of-Life or Death* or Advanced Illness* or Advanced Disease* or Life-Limiting or Die or Dying or Bereav* (Title) OR Palliat* or Hospice* or Bereav* or Terminal* or End-of-Life or Death* or Advanced Illness* or Advanced Disease* or Life-Limiting or Die or Dying or Bereav* (Abstract) Editions: WOS.SCI Date Run: Sun Aug 18 2024 16:17:26 GMT+0100 (British Summer Time) Results: 2533135

5: #4 AND #3 AND #2 AND #1 Editions: WOS.SCI Date Run: Sun Aug 18 2024 16:17:46 GMT+0100 (British Summer Time) Results: 1077

Google Scholar

#1 allintitle:Communicating|Communication Prognosis|Prognostic Uncertainty 7

#2 allintitle:Communicating|Communication Uncertainty Palliative|Hospice|"End of Life"|"Life Limiting" 6

7 + 6 = 12 (+ 1 duplicate)

Trip Database

PICO Search

Population: palliative OR "end of life"

Intervention: communicating OR communication

Outcome: (prognosis OR prognostic) AND uncertainty

8 results

NICE Guidelines

(Communicating OR Communication) AND (Prognosis OR Prognostic) Uncertainty AND (Palliative OR Hospice OR "End of Life" OR "Life Limiting")

20

Marie Curie via Google

(Prognosis OR Prognostic) Uncertainty site:mariecurie.org.uk

110

NHS Digital via Google

(Prognosis OR Prognostic) Uncertainty site:digital.nhs.uk

For peer review only

6

NHS Improvement via Google

(Prognosis OR Prognostic) Uncertainty site:improvement.nhs.uk

0

Royal College of Physicians via Google

(Prognosis OR Prognostic) Uncertainty site:rcp.ac.uk

19

General Medical Council via Google

(Communicating OR Communication) (Prognosis OR Prognostic) Uncertainty (Palliative OR "End of Life") site:gmc-uk.org

146

Department of Health via Google

intitle:(Communicating OR Communication) (Prognosis OR Prognostic) Uncertainty (Palliative OR "End of Life") site:gov.uk

273

For peer review only

List of fields for data extraction:

1st Author

Year

Country

Title

Aim

Study design (primary data collection (qualitative, quantitative, mixed), literature review (systematic or narrative), policy document, guideline, thesis, book chapter other)

Health professionals, Patients, Carers? (describe if combination)

Patient population & diagnoses (details of study populatino)

Setting (inpatient, outpatient, community, primary care, details of specialty if given)

type of communication described(verbal, written, patient/carer-professional, interprofessional, mixed or multiple types)

communication intervention (describe if relevant)

number of participants (primary data collection only)

Rigour assessment score (high, moderate, low)

Relevance score (high, moderate, low)

Additional details (free text)

For peer review only

Appendix 5. Details of included articles. 1st Author	Year	Country	Aim	Study design	HCP, Patients or Carers?	Patient population & diagnoses	Setting	Rigour	Relevance
						e.g. stroke, multimorbidity, frailty,			
Marie Curie	2014	UK	gain a deeper insight into the needs of terminally ill people and their non-professional carers, and understand experiences across the journey from diagnosis to death	Primary data collection - qualitative	Patients and carers	General Palliative care	Community	Moderate	Moderate
Marie Curie (Anderson)	2020	UK	to find the communication techniques that worked best at building awareness, helping decision making and closing the conversation.	Other	HCP + Patients + Carers	General medicine	All or mixed settings	Low	High
NICE	2015	UK	to improve end of life care for people in their last days of life by communicating respectfully and involving them, and the people important to them, in decisions and by maintaining their comfort and dignity	Policy document/guidance	N/A	last days of life	All or mixed settings	Moderate	Moderate
NICE	2018	UK	to improve diagnosis and treatment to increase the length and quality of life for people with heart failure	Policy document/guidance	N/A	heart failure	All or mixed settings	Moderate	Low
Alwidyan	2023	UK	to investigate healthcare professionals' barriers to and enablers of deprescribing in older hospice patients at the end of life and prioritise relevant theoretical domains for behaviour change to be incorporated into future interventions to facilitate deprescribing	Primary data collection - qualitative	HCP only	General palliative care - older patients	Hospice	High	Low
Anderson	2020	UK	To understand the practices used to accomplish prognostication and other relevant activities in conversations/communication between HCPs and relatives/friends of patients who are imminently dying	Thesis	HCP + Carers	General palliative care	Hospice	High	Moderate
Anderson	2020	UK	To understand how clinicians and the relatives/friends of patients at the very end of life manage uncertainty and reference time in prognostic conversations	Primary data collection - qualitative	HCP + Carers	General palliative care	Hospice	High	High
Anderson	2021	UK	To examine transitions out of prognostic talk in interactions between clinicians and the relatives and friends of imminently dying hospice patients.	Primary data collection - qualitative	Other	General palliative care	Hospice	High	Low

For peer review only

Appendix 5. Details of included articles.<br>1st Author	Year	Country	Aim	Study design	HCP, Patients or Carers?	Patient population & diagnoses	Setting	Rigour	Relevance
Anstey	2012	UK	To explore the personal and professional experience of consultant cardiologists, oncologists and old age psychiatrists and the consequent impact on referral practices to specialist palliative care services	Primary data collection - qualitative	HCP only	Cardiology, Oncology, Old-Age Psychiatry	Secondary care	Moderate	Low
Bailey	2018	UK	To offer advice and support to doctors to develop and improve in confidence when discussing their patients' preferences and values as they approach end of life	Policy document/guidance	HCP + Patients	Mixed - general medicine and palliative care	All or mixed settings	Moderate	Moderate
Barclay	2011	UK	To review the literature concerning conversations about EOLC between patients with heart failure and healthcare professionals, with respect to the prevalence of conversations; patients' and practitioners' preferences for their timing and content; and the facilitators and blockers to conversations.	Literature review	HCP + Patients + Carers	Heart failure	All or mixed settings	High	High
Baum	2020	USA	To provide commentary on a study (Seow et al) regarding potential utility of a patient-oriented prognostic tool	Literature review	N/A	Cancer	All or mixed settings	Low	Low
Bayuo	2021	Ghana	To explore the end of life care experiences of burn care staff and ascertain how their experiences can facilitate the development of clinical guidelines.	Primary data collection - qualitative	HCP only	Severe burns	Secondary care	High	Low
Becker	2020	Switzerland	To discuss communication challenges in EOL decision making and outline strategies from an area of growing interest and research	Literature review	Other	General palliative care	Other	Low	High
BGS	2020	UK	to support clinicians and others to consider the needs of frail older people as they move towards the end of their lives and help them to provide high quality care	Policy document/guidance	N/A	Frailty	Other	Low	Moderate
Bloomer	2018	Australia	to explore how end-of-life care goals and decision-making are communicated in a geriatric inpatient rehabilitation setting.	Primary data collection - qualitative	HCP only	Geriatrics, frailty	Community	Moderate	Moderate
Bloomer	2019	Australia	To investigate end-of-life care provision for older people in subacute care.	Primary data collection - quantitative observational	HCP + Patients + Carers	Geriatrics	Other	Moderate	Low

For peer review only

Appendix 5. Details of included articles. 1st Author	Year	Country	Aim	Study design	HCP, Patients or Carers?	Patient population & diagnoses	Setting	Rigour	Relevance
Borbasi	2021	Australia	To describe the experience and perspectives of residential aged care managers on providing end of life care for residents living with dementia.	Primary data collection - qualitative	HCP only	Dementia	Care Home	High	Moderate
Bristowe	2015	UK	To examine the experience of care supported by the AMBER care bundle compared to standard care in the context of clinical uncertainty, deterioration and limited reversibility.	Primary data collection - qualitative	Patients and carers	General medicine	Secondary care	Moderate	High
Broom	2014	Australia	To explore a range of medical specialists' experiences of referral to palliative care.	Primary data collection - qualitative	HCP only	General Palliative care	Community	Moderate	Low
Bruun	2024 a	UK	To explore how hospice multidisciplinary team (MDT) members oriented to the delicacy of prognostication during their meetings.	Primary data collection - qualitative	HCP only	General palliative care	Hospice	High	Moderate
Bruun	2024 b	UK	To explore how temporality is conveyed in relation to patients' prognoses during hospice multidisciplinary team meetings.	Primary data collection - qualitative	HCP only	General palliative care	Hospice	High	High
Burgers	2022	Netherlands	To understand the challenges in daily clinical practice experienced by HCPs from different disciplines who provide palliative as well as general care to AYAs (adolescents and young adults) with a UPCP (uncertain and/or poor cancer prognosis).	Primary data collection - qualitative	HCP only	Young adults with advanced cancer	All or mixed settings	High	Moderate
Cagle	2016	USA	To respond to the need for a greater understanding of caregiver preferences and attitudes toward being included in discussions about life expectancy	Primary data collection - qualitative	Carers only	Adults with disability age 65+	Community	High	High
Carlini	2022	Australia	To synthesise and critically analyse the points of discordance and concordance between consumers and clinicians about quality end-of-life care.	Literature review	HCP + Patients + Carers	Older patients aged >60	All or mixed settings	Moderate	Low
Cauley	2016	USA	To describe how surgeons approach treatment decisions to avoid nonbeneficial surgery, or engage in preoperative conversations about end-of-life (EOL) care, and to identify modifiable factors to reduce nonbeneficial surgery near the EOL.	Primary data collection - qualitative	HCP only	Older patients with surgical emergencies	Secondary care	Moderate	Low

For peer review only - http://bmjopen.bmj.com/site/about/guidelines.xhtml

Appendix 5. Details of included articles.

1st Author	Year	Country	Aim	Study design	HCP, Patients or Carers?	Patient population & diagnoses	Setting	Rigour	Relevance
Cavanagh	2020	USA	We review the literature concerning patient, caregiver, and clinician perspectives on prognosis communication in heart failure	Literature review	HCP + Patients + Carers	Heart failure	Other	Moderate	Low
Chan	2011	Hong Kong	To explore the relationships among the patient's awareness of the prognosis, the family's awareness of the prognosis, the patient's anxiety, and difficulty in communicating with family members.	Primary data collection - quantitative observational	Patients and carers	General palliative care	Secondary care	Low	Moderate
Chang	2014	USA	To examine prognosis discussions held with hospitalized patients for whom palliative care consultations were requested, and if prognosis discussions did not occur, to explore why not.	Primary data collection - qualitative + quantitative	HCP only	General palliative care	Secondary care	Moderate	Moderate
Chen	2018	UK	To assess the adequacy of community-based services available in Northern Ireland (NI) and to meet the multidimensional needs of patients living with heart failure (HF), as experienced and perceived by general practitioners (GP).	Primary data collection - qualitative	HCP only	Advanced heart failure	Primary care	Moderate	Low
Chen	2022	USA	To explore patients' perceptions of and preferences for communication surrounding goals of palliative RT and cancer prognosis.	Primary data collection - qualitative	Patients only	Oncology - bone or lung metastases	Secondary care	High	Low
Chou	2017	USA	Describes oncologists' language during discussions of prognosis and treatment goals in clinical interactions with African American patients diagnosed with cancer.	Primary data collection - qualitative	HCP + Patients	African Americans with stage III or IV cancer	Secondary care	High	High
Chow	2018	Canada	To identify the main challenges to the access and delivery of palliative care in patients with advanced congestive heart failure, and to summarize recommendations for clinical practice based on the available literature.	Literature review	HCP + Patients + Carers	Advanced heart failure	N/A	Moderate	Moderate
Christakis	1999	USA	To understand how and why physicians do and do not prognosticate	Literature review	HCP + Patients	Mixed	N/A	Moderate	Moderate

For peer review only

Appendix 5. Details of included articles.

1st Author	Year	Country	Aim	Study design	HCP, Patients or Carers?	Patient population & diagnoses	Setting	Rigour	Relevance
Christakis	1995	USA	To address the questions: what is the role of prognosis in medical thought and practice? Why is prognosis not as explicit a part of medicine as diagnosis and therapy?	Thesis	HCP + Patients + Carers	Mixed	Other	High	High
Chu	2019	UK	Narrative review discussing prognostication in Palliative Care	Literature review	HCP + Patients + Carers	General palliative care	All or mixed settings	Low	High
Chu	2020	UK	To evaluate the recent literature on prognostication for patients with advanced cancer and to suggest ways in which physicians can improve their own clinical practice in formulating and communicating prognostic estimates.	Literature review	HCP + Patients + Carers	Advanced cancer	All or mixed settings	Low	High
Clarke	2018	UK	To investigate how patients and their family members make decisions about their future care as their condition deteriorates, with a particular focus on mealtimes, eating and drinking	Primary data collection - qualitative	HCP + Patients + Carers	Progressive neurological disease	Community	High	Moderate
Cohen	2016	USA	To assess the effectiveness of an educational quality improvement intervention designed to enhance fellows' communication with patients who have advanced kidney disease.	Primary data collection - qualitative + quantitative	HCP only	Advanced kidney disease	Secondary care	Moderate	Moderate
Combes	2021	UK	To develop and refine an intervention to increase advance care planning engagement with older people living with frailty.	Thesis	HCP + Patients	Older people with frailty	Community	High	Low
Connolly	2021	USA	to conduct a qualitative metasynthesis to answer the following research question:What are the post-stroke PC/EOL experiences from the perspectives of patients, their families and healthcare professionals (HCPs)	Literature review	HCP + Patients + Carers	Stroke	All or mixed settings	High	Moderate
Cormican	2022	Ireland	To synthesize qualitative research evidence on the experiences of informal carers caring for a patient with a chronic haematological malignancy	Literature review	Carers only	Chronic haematological malignancy	All or mixed settings	High	Low
Cottrell	2020	UK	To develop and refine an initial theory on engagement in ACP for people with MS and to identify ways to improve its uptake for those who desire it.	Literature review	HCP + Patients + Carers	Multiple Sclerosis	All or mixed settings	High	Low

For peer review only

Appendix 5. Details of included articles.<br>1st Author	Year	Country	Aim	Study design	HCP, Patients or Carers?	Patient population & diagnoses	Setting	Rigour	Relevance
Creutzfeldt	2016	USA	To present current knowledge and recommendations regarding communication tasks and practice approaches for neurologists as they practice primary palliative care, including discussing serious news, managing symptoms, aligning treatment with patient preferences, introducing hospice/terminal care, and using the multiprofessional approach.	Literature review	HCP + Patients + Carers	Neurology	N/A	Low	Moderate
Crowther	2011	UK	To explore needs of people with dementia and their informal carers in the last year of life and surrounding death	Thesis	HCP + Patients + Carers	Dementia	Community	High	Moderate
Dias	2022	Portugal	To explore physicians' barriers in performing advanced care planning in patients with cancer; to propose a guide for discussion of advanced care planning for Brazilian physicians	Thesis	HCP + Patients	Oncology	Secondary care	High	Low
Dickerson	2024	USA	To characterize surgeon communication of pancreatic cancer prognosis using VitalTalk's ADAPT framework	Primary data collection - qualitative	HCP + Patients	Oncology - pancreatic cancer	Secondary care	Moderate	Moderate
Diggle	2022	UK	To present and synthesise the current evidence around bereaved caregivers' experiences of end of life care for people with heart failure	Literature review	Carers only	Heart failure	All or mixed settings	High	Low
Disler	2022	Australia, New Zealand	To explore attitudes and perceived barriers to ACP in self-identified palliative care nurses engaged and interested in care for patients with COPD.	Primary data collection - qualitative + quantitative	HCP only	COPD	All or mixed settings	Moderate	Low
Downer	2022	USA	Narrative review discussing communication about time-limited trials in the intensive care unit	Literature review	N/A	Intensive Care	Secondary care	Low	Moderate
Ekberg	2021	UK	Informs evidence-based communication about illness progression and end of life through a rapid review of studies that directly observe how experienced clinicians manage such discussions.	Literature review	HCP + Patients + Carers	General palliative care	All or mixed settings	Moderate	High

Appendix 5. Details of included articles.<br>1st Author	Year	Country	Aim	Study design	HCP, Patients or Carers?	Patient population & diagnoses	Setting	Rigour	Relevance
Epstein	2021	USA	Explore typical reactions to epistemic and complex uncertainty and offer preliminary proposals for how mindfulness and shared mind might help clinicians and patients navigate more effectively towards clear, health-promoting and values-concordant responses.	Literature review	HCP + Patients + Carers	Serious illness	All or mixed settings	Low	High
Etkind	2016	UK	To examine in detail the concept of uncertainty in illness and its impact on patients and professionals; to appraise current practice and evidence in addressing uncertainty in life-limiting illness	Literature review	HCP + Patients + Carers	Life-limiting illness	All or mixed settings	Moderate	High
Etkind	2025	UK	To co-design clinical practice recommendations for communication of prognostic uncertainty in older people with advanced multimorbidity, based on the lived experience and expertise of patients, carers, and healthcare professionals.	Primary data collection - qualitative	HCP + Patients + Carers	Advanced multimorbidity	Other	High	High
Evans	2009	USA	To understand surrogate decision makers' views regarding whether physicians should discuss prognosis in the face of uncertainty.	Primary data collection - qualitative	Carers only	Intensive Care	Secondary care	High	High
Fallowfield	2002	UK	Narrative review discussing communication of truth vs deceit in palliative care	Literature review	HCP + Patients + Carers	General palliative care	All or mixed settings	Low	Moderate
Fenton	2023	USA	To investigate cancer caregivers' communication experiences and potential impact on patient and caregiver outcomes.	Primary data collection - qualitative	Carers only	Advanced cancer	Secondary care	High	Moderate
Fisher	2012	Australia	Narrative review discussing uncertainty in end-of-life care and shared decision making	Literature review	HCP + Patients + Carers	Intensive Care	Secondary care	Low	High
Fitzsimons	2019	UK and Ireland	To explore caregivers" experience when caring for a loved one with advanced heart failure at the end of life and to identify any unmet psychosocial needs.	Primary data collection - qualitative	Carers only	Heart failure	Secondary care	High	Low
Formagini	2022	USA	To explore key factors that affect the strength of the stigma around palliative care referrals and its association with end of life in cancer care.	Primary data collection - qualitative	HCP + Patients + Carers	Oncology	Secondary care	Moderate	Low

For peer review only

Appendix 5. Details of included articles.

1st Author	Year	Country	Aim	Study design	HCP, Patients or Carers?	Patient population & diagnoses	Setting	Rigour	Relevance
Gadoud	2013	UK	To explore aspects of a palliative care approach for people with advanced HF: recognition of need, transitions in care and impact on patients, family carers and clinicians	Thesis	HCP + Patients + Carers	Heart failure	All or mixed settings	High	Moderate
Gallagher	2015	Brazil, England, Germany, Ireland, Palestine	To understand nurses' EoL decision-making practices in ICUs in different cultural contexts	Primary data collection - qualitative	HCP only	Intensive Care	Secondary care	High	Low
Garland	2013	Canada	To review the existing literature to identify barriers that inhibit end-of-life communication in the heart failure population.	Literature review	HCP + Patients + Carers	Heart failure	All or mixed settings	High	Moderate
Geerse	2019	USA	To characterize the content of serious illness conversations and identify opportunities for improvement	Primary data collection - qualitative	HCP + Patients	Oncology	Secondary care	High	Moderate
Gerlach	2019	Germany	To explore experiences, views and perceptions of haemato-oncologists on the use of the 'Surprise'-Question in the haemato-oncology outpatients clinics of a university hospital in Germany.	Primary data collection - qualitative	HCP only	Haematological malignancy	Secondary care	Moderate	Moderate
Gofton	2018	Canada	To develop a conceptual understanding of the specific characteristics of palliative care in neurology and the challenges of providing palliative care in the setting of neurological illness.	Primary data collection - qualitative	HCP + Patients + Carers	Neurological disease	Secondary care	Moderate	Low
Gonella	2022	Italy	To investigate how end-of-life communication may contribute to palliative-oriented care at the end-of-life in nursing homes according to both families' and nurses' perspective. Secondly, to identify the contextual factors internal to the nursing home that may influence the timing and quality of communication. Thirdly, to confirm the foundations for a first theory of end-of-life communication	Primary data collection - qualitative	HCP + Carers	Palliative care in care homes	Care Home	Moderate	Low
Goodlin	2008	USA	Narrative review applying the current knowledge about discussing prognosis and participatory decision-making to heart failure care.	Literature review	HCP + Patients + Carers	Heart failure	All or mixed settings	Low	High

For peer review only

Appendix 5. Details of included articles.

1st Author	Year	Country	Aim	Study design	HCP, Patients or Carers?	Patient population & diagnoses	Setting	Rigour	Relevance
Goss	2023	USA	To better understand surrogate experiences and needs during the period of active decision-making in SABI (severe acute brain injury), to inform interventions to support SABI patients and families and improve clinician-surrogate communication.	Primary data collection - qualitative	Carers only	Severe acute brain injury	Secondary care	High	High
Gough	2019	UK	To explore patient-centred accounts of the value and timing of prognostic discussions in advanced soft tissue sarcoma	Primary data collection - qualitative	Patients only	Advanced soft tissue sarcoma	Secondary care	Moderate	Moderate
Gramling	2013	USA	To identify and describe major types of prognosis conversations that occur in the natural setting of palliative care consultation.	Primary data collection - qualitative	HCP + Patients + Carers	General palliative care	Secondary care	High	Low
Gramling	2013 a	USA	To describe prognosis communication during PC consultation with seriously ill hospitalized patients.	Primary data collection - qualitative + quantitative	HCP + Patients + Carers	seriously hospitalised patients	Secondary care	Moderate	Moderate
Gray	2021	USA	To describe the current literature on illness and prognostic understanding in patients with haematologic malignancies, to examine the insights gained from literature about illness and prognostic understanding in patients with solid tumors, and to discuss future directions to inform our approach to studying illness and prognostic understanding in haematologic malignancies.	Literature review	HCP + Patients + Carers	Haematological malignancy	All or mixed settings	Moderate	Moderate
Green	2011	UK	To explore the perspectives of health care professionals on the transition of heart failure patients to a palliative care approach.	Primary data collection - qualitative	HCP only	Heart failure	All or mixed settings	Moderate	Moderate
Gutierrez	2010	USA	To explore the communication practices of prognostic information at end of life between and among critical care nurses, physicians, and family members in ICU throughout patients' trajectories, and the social, institutional, professional, and political influences which shape these practices	Thesis	HCP + Patients + Carers	Intensive Care	Secondary care	High	High

For peer review only

Appendix 5. Details of included articles.

1st Author	Year	Country	Aim	Study design	HCP, Patients or Carers?	Patient population & diagnoses	Setting	Rigour	Relevance
Gutierrez	2012	USA	Explore prognostic communication in ICU settings	Primary data collection - qualitative	HCP + Carers	Intensive Care	Secondary care	High	Moderate
Gutierrez	2013	USA	To identify the timing of and influences on critical care physician communication of negative prognoses to family members in an ICU.	Primary data collection - qualitative	HCP + Carers	Intensive Care	Secondary care	High	Moderate
Habib	2019	USA	To characterise reported practices and predictors of prognostic discussions for a cohort of hematologic oncologists.	Primary data collection - quantitative observational	HCP only	Haematological malignancy	Secondary care	High	Moderate
Habib	2021	USA	To characterize whether prognostic uncertainty has increased and if tolerance of uncertainty is associated with prognostic discussion practices in thoracic oncology.	Primary data collection - quantitative observational	HCP only	Lung cancer	Secondary care	Moderate	High
Hagerty	2005	Australia	To clarify the current available knowledge of patient preferences, clinician views and current practice regarding the communication of prognosis.	Literature review	HCP + Patients	Cancer	All or mixed settings	Moderate	High
Halkett	2010	Australia	To understand patients' experiences of High Grade Glioma and identify and describe information and support needs along the disease trajectory.	Primary data collection - qualitative	Patients and carers	High grade glioma	Secondary care	High	Moderate
Hallen	2015	USA	To explore physicians' perceptions of the value of Clinician Prediction Models in EOL care.	Primary data collection - qualitative	HCP only	General medicine	Secondary care	Moderate	High
Han	2016 a	USA	Reflective piece discussing prognostic disclosure and uncertainty	Other	N/A	General palliative care	N/A	Low	Moderate
Han	2016 b	USA	To explore the effects of personalized numeric prognostic information on physicians' intentions to communicate prognosis to cancer patients at the end of life, and to identify factors that moderate these effects.	Primary data collection - quantitative observational	HCP only	End-stage cancer	Primary care	Moderate	Moderate

For peer review only

Appendix 5. Details of included articles.

1st Author	Year	Country	Aim	Study design	HCP, Patients or Carers?	Patient population & diagnoses	Setting	Rigour	Relevance
Hancock	2007	Australia	To conduct a systematic review of truth-telling in discussing prognosis with patients with progressive, advanced life-limiting illnesses and their caregivers.	Literature review	HCP + Patients + Carers	Advanced life-limiting illness	All or mixed settings	High	Moderate
Harrison	2019	Canada	To review how web-based prognosis tools for cancer patients and clinicians describe aleatory (risk estimates) and epistemic (imprecision in risk estimates) uncertainties.	Primary data collection - quantitative observational	N/A	Cancer	N/A	Moderate	Moderate
Harrison	2021	UK	To describe the palliative care needs and experiences of people with mesothelioma and their family carers.	Literature review	HCP + Patients + Carers	Mesothelioma	All or mixed settings	High	Low
Hendershot	2023	USA	To investigate the complexities and interplay between the concepts of prognostic uncertainty and patient preferences as they relate to the delivery of goal-concordant care to patients with severe acute brain injuries (SABI) in the (Neuro-ICU).Neurological Intensive Care Unit	Literature review	HCP + Patients + Carers	Severe acute brain injury	Secondary care	Low	High
Henselmans	2017	Netherlands	To examine how communication about life expectancy is initiated in consultations about palliative chemotherapy, and what prognostic information is presented.	Primary data collection - qualitative	HCP + Patients	Advanced cancer	Secondary care	High	High
Heyland	2009	Canada	To enrich our understanding of the relation between satisfaction with endoflife (EOL) care and discussions regarding the patient's prognosis.	Primary data collection - quantitative observational	Patients and carers	Advanced illness	Secondary care	Moderate	Low
Higginson	2013	UK	To develop and evaluate an interventional tool to improve communication and palliative care, using the ICU as an example of where this is difficult.	Other	HCP + Carers	Intensive Care Medicine	Primary care	High	Moderate
Hjelmfors	2014	Sweden	To describe HF nurses' perspectives on, and daily practice regarding, discussing prognosis and end-of-life care with HF patients in outpatient care. It further aims to explore barriers, facilitators and related factors for discussing these issues.	Primary data collection - quantitative observational	HCP only	Heart failure	All or mixed settings	Moderate	Moderate

For peer review only

Appendix 5. Details of included articles.

1st Author	Year	Country	Aim	Study design	HCP, Patients or Carers?	Patient population & diagnoses	Setting	Rigour	Relevance
Hjelmfors	2018	Sweden	To improve communication about the heart failure trajectory in patients, their families, and health care professionals	Thesis	HCP + Patients + Carers	Heart failure	All or mixed settings	High	Low
Hoff	2011	Sweden	To obtain a deeper understanding of how physicians are dealing with decision making and providing information to patients in situations where pertinent scientific knowledge is lacking or uncertain	Primary data collection - qualitative	HCP only	Oncology (Haematological and Lung)	Secondary care	Moderate	Moderate
Hoff	2014	Sweden	To identify inherent challenges in communicating information about imminent death.	Primary data collection - qualitative	HCP + Patients	Oncology (Haematological and Lung)	Secondary care	Moderate	High
Holloway	2013	USA	Narrative review discussing estimating and communicating prognosis in advanced neurologic disease	Literature review	HCP + Patients + Carers	Neurology	All or mixed settings	Low	High
Hudson	2017	UK	To explore the views and experiences of people who are homeless and those supporting them regarding conversations and approaches to palliative care.	Primary data collection - qualitative	HCP + Patients	General palliative care in patients experiencing homelessness	Community	High	Moderate
Hui	2019	USA	To provide an update on prognostication in patients with advanced cancer, and to discuss future directions for research in this field.	Literature review	HCP + Patients + Carers	Advanced cancer	All or mixed settings	Low	Moderate
Hui	2021	USA	Narrative review discussing the impact of prognostication on patient outcomes.	Literature review	HCP + Patients + Carers	Advanced cancer	All or mixed settings	Low	Moderate
Innes	2009	UK	To examine the research literature, to identify preferences for prognostic awareness in advanced cancer patients, to assess how much and what type of prognostic information cancer patients want in advanced disease and to identify any benefits that patients attribute to prognostic awareness.	Literature review	HCP + Patients + Carers	Advanced cancer	All or mixed settings	High	Moderate
Isaac	2017	USA	Editorial on discussing prognosis in the intensive care unit	Other	N/A	Intensive Care Medicine	Secondary care	Low	Moderate

For peer review only

Appendix 5. Details of included articles.

1st Author	Year	Country	Aim	Study design	HCP, Patients or Carers?	Patient population & diagnoses	Setting	Rigour	Relevance
Islam	2023	UK	To address the question: what are the barriers to and enablers of ethnically diverse patients, family caregivers and health-care professionals engaging in end-of-life care planning?	Primary data collection - qualitative	HCP + Patients + Carers	General palliative care	All or mixed settings	High	High
Jackson	2024	USA	Evidence supporting various communication strategies is presented, followed by a review of formal guidelines, when they exist	Literature review	N/A	Serious illness	All or mixed settings	Low	Moderate
Johnson	2012	USA	A case presentation of acute lung injury (ALI) progressing to acute respiratory distress syndrome (ARDS) requiring tracheal intubation and mechanical ventilation (ETMV) and narrative review discussing uncertainty in critical illness.	Literature review	HCP + Patients + Carers	Intensive Care Medicine	Secondary care	Low	High
Johnson	2015	UK	To explore the process of disclosure, patient experience and preferences for information regarding prognosis or risk of recurrence	Literature review	Patients only	Cancer	All or mixed settings	High	High
Jones	1983	USA	To determine the legal, professional, and ethical responsibilities of registered nurses as they make decisions about disclosure of prognostic information to dying adults	Thesis	HCP + Patients	Dying adults	All or mixed settings	Low	Low
Kantabanlang	2023	UK	To provide an overview of current evidence on the experiences and palliative care needs of patients with hematologic malignancies and their families and the barriers and challenges of integrating palliative care into hematology care in Asia.	Literature review	HCP + Patients + Carers	Haematological malignancy	All or mixed settings	Low	Low
Kapo	2006	USA	Narrative review discussing prognosis in chronic diseases	Literature review	HCP + Patients + Carers	Chronic life-limiting illness	All or mixed settings	Low	Moderate
Kimbell	2014	UK	To broaden our understanding of the experience of living and dying with advanced liver disease, and to examine the utility of a qualitative longitudinal, multi-perspective methodology in end-of-life research.	Thesis	HCP + Patients + Carers	Advanced liver disease	All or mixed settings	High	High
Kimbell	2015	UK	To understand the experiences and support needs of people with advanced liver disease and those of their lay and professional carers to	Primary data collection - qualitative	HCP + Patients + Carers	Advanced liver disease	All or mixed settings	High	Moderate

For peer review only

Appendix 5. Details of included articles.<br>1st Author	Year	Country	Aim	Study design	HCP, Patients or Carers?	Patient population & diagnoses	Setting	Rigour	Relevance
			inform improvements in the supportive and palliative care of this rapidly growing but currently neglected patient group.						
Ko	2020	USA	To explore decision-making experiences, related to utilisation of hospice care programmes from a retrospective perspective, with family caregivers (FCGs) in a rural US–Mexico border region.	Primary data collection - qualitative	Carers only	General palliative care	Community	Moderate	Low
Krawczyk	2016	Canada	To report on the concept of "communicating prognostic uncertainty" which emerged from a mixed methods survey asking family members to rank their satisfaction in seven domains of hospital end-of-life care.	Primary data collection - qualitative	Carers only	General palliative care	All or mixed settings	Moderate	High
Kupeli	2019	UK	To understand the similarities and differences between healthcare professionals' and family carers' perspectives on optimal end-of-life care, gain insights into how the interdependent roles of family carers and HCPs can be optimised, and make recommendations for policy and practice.	Primary data collection - qualitative	Carers only	Advanced dementia	Care Home	Moderate	Moderate
Langmuir	2023	Canada	To review the literature on the experiences of uncertainty for individuals with advanced or metastatic cancer patients who are receiving immunotherapy or TT.	Literature review	Patients only	Advanced cancer	Secondary care	High	Moderate
LeBlanc	2018	USA	Narrative review discussing communicating prognosis in oncology	Literature review	N/A	Advanced cancer	N/A	Low	High
LeClaire	2005	USA	To determine whether the timing of prognostic information delivery by physicians is associated with caregiver satisfaction with communication or decision making in the ICU.	Primary data collection - quantitative observational	Carers only	Intensive Care	Secondary care	Moderate	Low
Lee	2022	Canada	Narrative review discussing uncertainty in surgical palliative care	Literature review	Other	Surgery	All or mixed settings	Low	Moderate
Low	2017	UK	To identify the limitations in palliative care provision in the last year of life for people with liver cirrhosis and potential barriers to and enablers of palliative care.	Primary data collection - qualitative	HCP + Patients	Advanced chronic liver disease	Secondary care	Moderate	Low
Maguire	1988	UK	Opinion article discussing handling uncertainty, collusion and denial when communicating with cancer patients	Other	N/A	Oncology	N/A	Low	Moderate

Appendix 5. Details of included articles.

1st Author	Year	Country	Aim	Study design	HCP, Patients or Carers?	Patient population & diagnoses	Setting	Rigour	Relevance
Mandel	2017	USA	To identify the barriers to serious illness conversations in the dialysis population, review best practices in and specific approaches to conducting serious illness conversations, and offer solutions to overcome barriers as well as practical advice, including specific language and tools, to implement serious illness conversations in the dialysis population.	Literature review	N/A	End-stage renal disease	All or mixed settings	Low	High
Masterson	2018	USA	To draw on the experiences of clinicians to clarify the concept of prognostic understanding and examine the mechanisms that contribute to patient understanding and uptake of prognostic information.	Primary data collection - qualitative	HCP only	Advanced cancer	Secondary care	Moderate	High
Maxwell	2020	USA	To examine hospitalized older adults' and family caregivers' receptivity to general (vs. individualized) prognostic information about frailty, injury, and one-year outcomes; and to determine information needs based on prognostic information.	Primary data collection - qualitative	Patients and carers	Frailty/older adults	Secondary care	Moderate	Low
McClung	2013	USA	To discuss specific clinical recommendations and an analysis of some of the ethical issues involved in the provision of care to elderly patients in the terminal stages of heart failure.	Literature review	HCP + Patients + Carers	Advanced heart failure	All or mixed settings	Moderate	Low
McDonald	2016	UK	Policy document on improving communication with patients in the National Health Service	Policy document/guidance	N/A	General palliative care	N/A	Moderate	Low
McLennon	2013	USA	To describe the frequency and types of ethical dilemmas experienced by oncology nurses caring for advanced cancer patients and to summarise their written comments about prognosis-related communications.	Primary data collection - qualitative	HCP only	Oncology	Secondary care	Moderate	Moderate
Medendorp	2021	Netherlands	To generate an overview of available recommendations on how to communicate uncertainty with patients during clinical encounters.	Literature review	N/A	General medicine	All or mixed settings	Moderate	High
Michael	2022	UK	This chapter covers the importance of cancer communication and strategies to aid practitioners in discussing recurrence, progression, prognostication, and transition to palliative care.	Book chapter	N/A	Advanced progressive disease	All or mixed settings	Low	Moderate

Appendix 5. Details of included articles.

1st Author	Year	Country	Aim	Study design	HCP, Patients or Carers?	Patient population & diagnoses	Setting	Rigour	Relevance
Moglia	2024	Italy	To investigate neurologists' attitudes toward prognostic communication and their knowledge and utilization of the ENCALS survival model in clinical practice.	Primary data collection - quantitative observational	HCP only	Amyotrophic Lateral Sclerosis	Secondary care	Moderate	High
Molzahn	2020	Canada	To describe how people diagnosed with heart failure and their family members describe uncertainty related to impending death.	Primary data collection - qualitative	Patients and carers	Advanced heart failure	Secondary care	High	High
Momen	2011	UK	To reviews the extent to which there is evidence that conversations with clinicians about end-of-life care occur for heart failure patients in practice.	Literature review	HCP + Patients	Heart failure	All or mixed settings	High	Moderate
Mone	2021	UK	To identify if there is concordance between actual prognosis and accurate prognostic awareness in individuals with advanced cancer, and to identify the rationale for any discordance between the two.	Literature review	N/A	Advanced cancer	N/A	High	Moderate
Mount	1980	Canada	Editorial discussing the psychological impact of urological cancer	Other	N/A	Oncology and general palliative care	Secondary care	Low	Moderate
Nadicksbernd	2023	UK	To explore professionals' perspectives of difficult conversations about life-limiting, deteriorating health and end-of-life.	Thesis	HCP only	General palliative care	Community	High	Moderate
Nagelschmidt	2020	Germany	To identify barriers that hinder or influence the discussion of end- of- life issues in the family context.	Literature review	Patients and carers	terminally ill adults	All or mixed settings	Moderate	Low
Narayan	2017	USA	To characterise heart failure patient preferences for, reactions to, and application of individualized SHFM prognostic information along with individualized SHFM (Seattle Heart Failure Model) prognostic information along with their understanding of uncertainty in population-based estimates.	Primary data collection - qualitative	Patients only	Heart failure	Secondary care	High	High
Ngwenya	2021	UK	To identify factors that impact communication and support and recommend ways to improve patients' understanding of living with life-threatening illness.	Primary data collection - qualitative	HCP + Patients + Carers	Lung cancer and COPD	Primary care	Moderate	High

For peer review only

Appendix 5. Details of included articles.

1st Author	Year	Country	Aim	Study design	HCP, Patients or Carers?	Patient population & diagnoses	Setting	Rigour	Relevance
Noble	2017	UK	To explore clinician views on decisional conflict in patients with end-stage kidney disease.	Primary data collection - qualitative	HCP only	End-stage renal disease	Secondary care	Moderate	Moderate
Olsson	2021	Australia	To explore existing guidelines around palliative care to increase current understanding of end-of-life communication processes applicable to the acute care setting.	Literature review	Other	end of life	Secondary care	Moderate	Low
Owusuaa	2021	Netherlands	To explore physicians' views on identifying and disclosing the last phase of life of patients with different illness trajectories.	Primary data collection - qualitative	HCP only	Cancer, COPD	All or mixed settings	Moderate	Moderate
Pardue	2020	USA	To apply Mishel's (1990) model of uncertainty in chronic illness to heart failure patients as a framework for clarifying the role uncertainty plays in end-of-life communication; to determine whether time perspectives play a role in heart failure patients' engagement in end-of-life communication; to elucidate the complex relationships between time perspective and uncertainty in illness and their association with end-of-life communication.	Primary data collection - quantitative observational	Patients only	Heart failure	All or mixed settings	Moderate	Moderate
Parker	2006	Australia	To conduct a systematic review regarding communication of prognosis and EoL issues with adults, and or their caregivers, with an advanced progressive life limiting illness.	Literature review	HCP + Patients + Carers	Advanced illness	All or mixed settings	High	High
Parker	2007	Australia	to review studies regarding prognostic/endof-life communication with adult patients in the advanced stages of a life-limiting illness and their caregivers	Literature review	HCP + Patients + Carers	Advanced illness	All or mixed settings	High	Moderate
Parry	2024	UK	To systematically collate, examine, and summarize what knowledge and insights have been established by Conversation Analysis research in palliative care and in other healthcare settings where there are interactions about the end of the patient's life	Literature review	N/A	General palliative care	N/A	Low	High
Parvez	2015	USA	To consider the concept of 'uncertainty' in clinical practice for end stage renal disease from physician and patient perspectives.	Literature review	HCP + Patients	End stage renal disease	All or mixed settings	Low	High

For peer review only

Appendix 5. Details of included articles.

1st Author	Year	Country	Aim	Study design	HCP, Patients or Carers?	Patient population & diagnoses	Setting	Rigour	Relevance
Payne	2010	UK	To identify patients' and family members' experiences of acute stroke and their preferences for end-of-life care.	Primary data collection - qualitative	Patients and carers	Acute stroke	Secondary care	Moderate	High
Peoples	2020	USA	To identify factors that influence how prognostic uncertainty is viewed by physicians, as it relates to their communications with families.	Primary data collection - qualitative	HCP only	General medicine/surgery	Secondary care	High	High
Perumalswami	2019	USA	To present an overview of the role of prognostic uncertainty in oncologists' emotions, why the landscape of prognostication for cancer is changing, and how certain medical advances that have the potential to induce a "Lazarus effect" for patients near death present daily ethical challenges for oncologists	Literature review	N/A	Oncology	Secondary care	Low	Moderate
Pfeil	2015	Germany	To explore the factors that hinder communication about the terminal nature of advance cancer, and to reconstruct how physicians and nurses in oncology perceive their roles in preparing patients for end-of-life (EOL) decisions.	Primary data collection - qualitative	HCP only	Advanced cancer	Secondary care	Moderate	Moderate
Piovesan	2024	Canada	To evaluate the usefulness, for patients and families, of knowing the prognosis during the last days of life and to identify its utility in planning the activities that are important to them during their remaining days.	Primary data collection - quantitative observational	Patients and carers	Hospice inpatients	Hospice	Moderate	Moderate
Pontin	2011	UK	To explore hospital specialist palliative care professionals' experience of prognostication.	Primary data collection - qualitative	HCP only	General palliative care	Secondary care	Moderate	High
Pooler	2018	Canada	To explore bereaved caregivers' experiences and perceptions of an early integrated palliative approach implemented at a Multidisciplinary Interstitial Lung Disease Clinic.	Primary data collection - qualitative	Carers only	Idiopathic Pulmonary Fibrosis	Secondary care	Moderate	Low
Powers	2011	USA	To illustrate distinctions and intersections of palliative care and end-of-life services through examples from case-centered data of older adults cared for during a four-year ethnographic study of an acute care hospital palliative care consultation service.	Primary data collection - qualitative	HCP + Patients + Carers	General palliative care	Secondary care	Low	Moderate

Appendix 5. Details of included articles.

1st Author	Year	Country	Aim	Study design	HCP, Patients or Carers?	Patient population & diagnoses	Setting	Rigour	Relevance
Pun	2023	Hong Kong	To synthesise empirical findings on the role of family in end- of- life (EOL) communication and to identify the communicative practices that are essential for EOL decision- making in family- oriented cultures.	Literature review	HCP + Patients + Carers	end of life care	All or mixed settings	Moderate	Moderate
Rafferty	2016	USA	To explore spouses' reasons for seeking and avoiding conversations regarding their partners' EOL care preferences, and examine whether certain demographic factors (eg, income, gender, age) more likely contributed to the initiation of EOL conversations.	Primary data collection - qualitative + quantitative	Carers only	End of life care	All or mixed settings	Moderate	Low
Reid	2015	UK	To explore healthcare professionals' views on delivering end-of-life care within an acute hospital trust	Primary data collection - qualitative	HCP only	General palliative care	Secondary care	Moderate	Moderate
Rhee	2013	Australia	To explore the perspectives of general practitioners (GPs) on factors influencing the implementation of ACPs.	Primary data collection - qualitative	HCP only	General practice	Primary care	High	Low
Richards	2013	UK	To explore if and how information about a transition to a palliative care approach was communicated to patients recently discharged from hospital and who fulfilled standardized criteria for palliative care need.	Primary data collection - qualitative	Patients and carers	General palliative care	Secondary care	Moderate	Moderate
Ridley	2013	Australia	Narrative review discussing uncertainty in end-of-life decision making	Literature review	N/A	Intensive care medicine	Secondary care	Low	High
Risco	2022	USA	To discuss the importance of prognostication and developing a prognostic mindset, as well as providing a framework for formulating prognosis and communicating "what to expect."	Literature review	N/A	Neurology	All or mixed settings	Low	High
Robinson	2021	New Zealand	To explore the impact of uncertainty on how death is remembered by bereaved family members of people with heart disease.	Primary data collection - qualitative	Carers only	Heart disease	All or mixed settings	Moderate	High
Rowe	2021	USA	To qualitatively explore the challenges presented by the COVID-19 pandemic from the perspective of palliative care clinicians.	Primary data collection - qualitative	HCP only	General palliative care	All or mixed settings	High	Low

For peer review only

Appendix 5. Details of included articles.<br>1st Author	Year	Country	Aim	Study design	HCP, Patients or Carers?	Patient population & diagnoses	Setting	Rigour	Relevance
Salins	2024	India	To address palliative care needs in the ICU by synthesising palliative care practices provided at end-of-life to critically ill patients and their families before, during, and after ICU admission.	Literature review	HCP + Patients + Carers	Intensive care	Secondary care	High	Moderate
Sanders	2023	Canada	To propose three methodological innovations to advance the basic science of serious illness communication.	Literature review	N/A	Serious illness	All or mixed settings	Low	Moderate
Scholz	2020	Australia	To synthesise clinicians' understandings of communication in end-of-life care.	Literature review	HCP only	Intensive care	All or mixed settings	Moderate	Low
Schuster	2012	USA	To evaluate whether and how physicians discuss prognostic uncertainty with surrogate decision makers of patients who are highly likely, but not certain, to die.	Primary data collection - qualitative + quantitative	HCP + Carers	Intensive care	Secondary care	Moderate	High
Schutz	2018	USA	Editorial discussing communication, personhood and uncertainty in neuropalliative care	Literature review	HCP + Carers	Neuropalliative care	N/A	Low	Low
Sedhom	2024	USA	Narrative review discussing person-centred supportive care in precision oncology	Literature review	HCP + Patients + Carers	Oncology	All or mixed settings	Low	High
Selman	2019	UK	To explore views and experiences of communication, information provision and treatment decision-making among older patients receiving conservative care.	Primary data collection - qualitative	Patients only	Older people with end-stage renal disease	Secondary care	High	Moderate
Sikhosana	2023	Australia	To synthesise the best available evidence on the experience of persons living with a life-limiting illness about expressing their voice during communication with healthcare professionals.	Literature review	Patients only	Life-limiting illness	All or mixed settings	High	Moderate
Simpkin	2019	USA	To provide a narrative review of studies relating to the communication of uncertainty, highlighting current strategies together with challenges and barriers, and outline a framework for future research.	Literature review	HCP + Patients + Carers	General	All or mixed settings	Low	High

For peer review only

Appendix 5. Details of included articles. 1st Author	Year	Country	Aim	Study design	HCP, Patients or Carers?	Patient population & diagnoses	Setting	Rigour	Relevance
Smith	2014		To examine ethical issues in disease prognosis and prognostication in palliative care from a clinical perspective.	Book chapter	N/A	General palliative care	All or mixed settings	Moderate	High
Stanek	2020	USA	(a) provide an analysis and clarification of the concept of goals of care, (b) identify the frequency, distribution, and domains of uncertainty expressed by patients with advanced cancer in their communication with the palliative care team using Han's taxonomy of uncertainty, and (c) describe the magnitude and precision of the association between types of expressed patient uncertainty and patients' self-rated prognosis.	Thesis	Patients only	Advanced cancer	Secondary care	High	High
Stiel	2019	Australia	To explore the experiences of and communication techniques used by palliative care physicians as they consider and discuss nursing home placements for their patients.	Primary data collection - qualitative	HCP only	General palliative care	All or mixed settings	High	Low
Stocker	2014	UK	To explore experiences of giving or receiving a prognosis and managing the transition point from diagnosis to palliative and end of life care for those with heart failure.	Thesis	HCP + Patients + Carers	Heart failure	All or mixed settings	High	High
Stocker	2017	UK	To explore experiences of giving or receiving a prognosis and advanced palliative care planning for those with heart failure.	Primary data collection - qualitative	HCP + Patients + Carers	Heart failure	Secondary care	High	Moderate
Sullivan	2007	USA	To describe whether and when physicians report recognizing and communicating the imminence of death and identify potential barriers and facilitators to recognition and communication about dying in the hospital.	Primary data collection - qualitative	HCP only	General/terminal illness	Secondary care	Moderate	Moderate
Szawarski	2016	UK	Editorial discussing uncertainty in critical illness	Literature review	N/A	Critical illness	N/A	Low	High
Tarbi	2021	USA	To describe the existential dimensions of prognosis communication in naturally-occurring palliative care conversations.	Primary data collection - qualitative + quantitative	HCP + Patients + Carers	Advanced cancer	Secondary care	High	Moderate

For peer review only

Appendix 5. Details of included articles.<br>1st Author	Year	Country	Aim	Study design	HCP, Patients or Carers?	Patient population & diagnoses	Setting	Rigour	Relevance
Tavares	2019	UK	To understand COPD patients' preferences for palliative care discussions; and explore clinicians' opinions and experiences when holding these conversations.	Thesis	HCP + Patients	COPD	All or mixed settings	High	Moderate
Tavares	2020	UK	To understand the preferences of patients with chronic obstructive pulmonary disease for discussions about palliative and advance care planning with clinicians.	Primary data collection - qualitative	Patients only	COPD	All or mixed settings	High	Low
Temel	2016	USA	Narrative review discussing prognostic uncertainty in the modern era of cancer therapeutics	Literature review	N/A	Oncology	All or mixed settings	Low	High
Tetteh	2021	USA	To examine openness and topic avoidance in interpersonal communication about ovarian cancer.	Primary data collection - qualitative	Patients only	Ovarian cancer	All or mixed settings	High	Moderate
Tilburgs	2018	Netherlands	To determine the barriers and facilitators faced by GPs related to ACP with people with dementia.	Literature review	HCP + Patients + Carers	Dementia	Primary care	High	Low
Tolsa	2022	Switzerland	To explore (1) the perspectives and experiences of clinicians regarding the use of predictive scores for LST decision making in severe acute stroke, and (2) clinicians' awareness of their own cognitive biases in this context.	Primary data collection - qualitative	HCP only	Severe acute stroke	Secondary care	High	Moderate
Tosh	2018	UK	Reflective article discussing conversations about death	Other	N/A	General/end-of-life	N/A	Low	Moderate
Trankle	2020	Australia	To complement our quantitative survey data by providing a greater understanding of the experiences and views of clinicians when engaging in EoL discussions.	Primary data collection - qualitative + quantitative	HCP only	General palliative care	All or mixed settings	High	Moderate
Tuck	2015	USA	To determine preferences of patients with Parkinson's Disease for timing and initiation of discussions regarding treatment, prognosis, advanced care planning, and end-of-life care options such as hospice.	Primary data collection - qualitative + quantitative	Patients only	Parkinson's Disease	Secondary care	High	Low

For peer review only

Appendix 5. Details of included articles. 1st Author	Year	Country	Aim	Study design	HCP, Patients or Carers?	Patient population & diagnoses	Setting	Rigour	Relevance
Udo	2018	Sweden	To explore palliative home care physicians' experiences regarding end-of-life breakpoint communication	Primary data collection - qualitative	HCP only	General palliative care	Community	High	Moderate
van der Velden	2020	Netherlands	To offer up-to-date, evidence-based guidance on prognostic communication in palliative oncology.	Literature review	Patients only	Oncology	All or mixed settings	Moderate	Moderate
van der Velden	2021	Netherlands	To explore (1) how advanced cancer patients phrase their questions and ideas about prognosis; (2) how consulting oncologists discuss prognosis with advanced cancer patients; and (3) how advanced cancer patients and consulting oncologists discuss prognostic information previously provided by the referring oncologist.	Primary data collection - qualitative	HCP + Patients	Advanced cancer	Secondary care	High	High
van der Velden	2023	Netherlands	(1) describe the extent and direction of prognostic discordance, patients' prognostic information preferences in cases of prognostic discordance, and physicians' awareness of prognostic discordance; and (2) examine which patient, physician, and caregiver factors predict prognostic discordance.	Primary data collection - quantitative observational	HCP + Patients + Carers	Advanced cancer	Secondary care	High	Moderate
van der Velden	2024	Netherlands	To investigate the effects of prognostic communication strategies (prognostic disclosure vs. communication of unpredictability vs. non-disclosure; standard vs.standard and best-case vs.standard, best- and worst-case survival scenarios; numerical vs.word-based estimates) on prognostic perceptions, treatment decision-making and end-of-life anticipation in advanced cancer.	Primary data collection - quantitative observational	Other	Advanced cancer	All or mixed settings	Moderate	Moderate
van der Velden	2024 b	Netherlands	To investigate effects of prognostic communication strategies on emotions, coping, and appreciation of consultations in advanced cancer.	Primary data collection - quantitative observational	Other	Advanced cancer	All or mixed settings	Moderate	Moderate
van Eenennaam	2020	Netherlands	To develop a communication guide to support neurologists and rehabilitation physicians in discussing personalized prognosis, tailored to the individual needs and preferences of people with ALS and their families.	Policy document/guidance	HCP + Patients + Carers	Amyotrophic Lateral Sclerosis	All or mixed settings	Moderate	High

Appendix 5. Details of included articles.

1st Author	Year	Country	Aim	Study design	HCP, Patients or Carers?	Patient population & diagnoses	Setting	Rigour	Relevance
Vlckova	2020	Czech Republic	To synthesise the available literature on factors associated with accurate prognostic awareness in patients with cancer.	Literature review	HCP + Patients	Oncology	All or mixed settings	High	Moderate
Walczak	2013	Australia, USA	To explore patients' perspectives across two cultures (Australia and USA) regarding communication about prognosis and end-of-life care issues and to consider the ways in which these discussions can be optimised.	Primary data collection - qualitative	Patients only	Oncology	Secondary care	High	High
Walczak	2016	Australia	To identify and synthesise evidence for interventions targeting end-of-life communication.	Literature review	HCP + Patients + Carers	End-of-life care	All or mixed settings	High	Low
Waldrop	2012	USA	Guided by concepts from the Sense of Coherence Theory, which illuminates factors that contribute to adaptation in stressful situations, the objective of this study was to explore and describe family caregivers' accounts of the nature and timing of communication they had with a loved one's health care provider(s) during the advanced stages of cancer and before hospice enrollment.	Primary data collection - qualitative	Carers only	Advanced cancer	Hospice	High	Moderate
Wijsenbeek	2022	Netherlands	Consider the difficulties inherent in discussions with patients with IPF and their loved ones, and how clinicians might communicate with patients more effectively, from breaking the news about the diagnosis to providing support throughout the course of the disease.	Literature review	HCP + Patients + Carers	Idiopathic Pulmonary Fibrosis	All or mixed settings	Low	High
Wittenberg	2009	USA	Narrative review discussing estimating prognosis in end-stage renal disease	Literature review	HCP + Patients + Carers	End-stage renal disease	Secondary care	Low	Moderate
Wittenberg-Lyles	2008	USA	To understand the use and effectiveness of current communication protocols in terminal prognosis disclosures.	Primary data collection - qualitative	HCP + Patients + Carers	General palliative care	Secondary care	Low	Low
Wu	2024	China	To synthesise conversation analytic studies examining communication between healthcare professionals (HCPs) and patients/companions in the setting of palliative care.	Literature review	HCP + Patients + Carers	General palliative care	All or mixed settings	Moderate	High

For peer review only - http://bmjopen.bmj.com/site/about/guidelines.xhtml

Appendix 5. Details of included articles.<br>1st Author	Year	Country	Aim	Study design	HCP, Patients or Carers?	Patient population & diagnoses	Setting	Rigour	Relevance
Xie	2023	Canada	Narrative review discussing Artificial Intelligence decision support tools for end-of-life care planning conversations	Literature review	HCP + Patients + Carers	End-of-life care	All or mixed settings	Moderate	Low
Young	2024	New Zealand	To synthesise literature on patients in the last months of life due to a cancer diagnosis who have declined a referral to end-of-life care.	Literature review	Patients and carers	Cancer	All or mixed settings	High	High
Ziegler	2015	Australia	Reflective article discussing risk and prognosis	Other	N/A		N/A	Low	Moderate

For peer review only

Appendix 6. Table of CMO constructs

	Context	Mechanism	Outcome
1	well trained, experienced clinician	Assess and establish patient understanding of condition	Good communication: individualised, timely, **sensitive**, respectful, consistent
2	well trained, experienced clinician	explicit/open communication of prognostic uncertainty	shared understanding of situation, sense of meaning, acceptance, facilitates care planning
3	clinician tolerates uncertainty	explicit/open communication of prognostic uncertainty	shared understanding of situation, sense of meaning, acceptance, facilitates care planning
4	patient/family/carer prompt prognostic discussion	Clinician engages proactively with uncertainty & their reactions to it	explicit/open communication of prognostic uncertainty
5	patient too unwell to communicate	honest/open communication of prognostic uncertainty with family	family satisfaction with end of life care
6	uncertain future illness trajectory	communication of a range of possible scenarios/repeated discussions	patient/family can prepare
7	patient or family unwilling to discuss uncertainty, prefer not to know	conversation not initiated	lack of understanding may lead to denial, distress, and even unresolved bereavement narratives for family, but can also enable patient to have hope and 'live in the moment'
8	strong care relationship, e.g. from relational continuity	collaboration/ working together to enable explicit/open/confident communication of prognostic uncertainty	shared understanding of situation, sense of meaning, acceptance, facilitates care planning
9	empathic care relationship	being available and appearing caring	patient and family feel supported despite uncertainty
10	differences in patient vs relative communication needs	degree to which communication is based on assessment of individual needs/preferences	differing communication needs are met
11	empathic care relationship	collaborative approach to understanding prognostic uncertainty	shared understanding of situation, acceptance, facilitates care planning

For peer review only

	Context	Mechanism	Outcome
12	poor previous communication	Acknowledgement of previous error, open acknowledgement of prognostic uncertainty	builds trust, avoids further conflict
13	complex illness situations	case conference with family	helps to explore and manage uncertainty, leaves room for hope, increases trust, supports decision making
14	treatment decision making in context of prognostic uncertainty	using the concept of 'practical certainty' i.e. 'we are as sure as we can be' during communication with patient and/or family	supports decision making whilst acknowledging inherent uncertainty
15	perceived expertise/authority of clinician delivering prognostic information	value that patient/family place on prognostic information	degree to which uncertainty is accepted/integrated
16	Uncertain prognosis in cancer care	having prognostic discussion with treating oncologist with whom patient has a prior relationship	greater prognostic awareness, hearing prognostic uncertainty from a senior clinician is perceived as more acceptable
17	baseline level of clinician expertise	clinician training/use of prognostic models	Clinician engages proactively with uncertainty & their reactions to it
18	knowledge of best practice but absence of training/experience/ strategies	clinicians anxious about holding conversations and avoid them	implicit/tacit/no communication of prognostic uncertainty
19	Acute illness with uncertain recovery, e.g. severe stroke	use prognostic tools as an aid + clinicians to recognise own biases regarding communicating prognostic uncertainty	improves decision making + clinician-patient/family relationship (especially where families prefer explicit communication of uncertainties)
20	inexperienced, poorly trained clinician	conversation not initiated with patient/family	family/informal caregivers may feel pressure to hold or communicate uncertainty themselves, leading to distress, disempowerment and regret
21	weak care relationship	implicit/tacit/no communication of prognostic uncertainty	lack of understanding, loss of trust, unresolved bereavement narratives

For peer review only

	Context	Mechanism	Outcome
22	patient or relative asks about prognosis	uncertainty may or may not be mentioned depending on clinician tolerance/experience/judgement	Variation in degree of understanding of prognostic uncertainty
23	emotional distress from uncertainty	moving from discussion of uncertainty to end on something where there is certainty	orients towards action and certainty, supports planning
24	Desire to shield patients	vague communication; Uncertainty avoided or un-acknowledged by clinicians	Patient and family do not understand prognostic uncertainty, may lead to greater distress
25	clinicians not confident of prognosis	do not engage in discussions about it	patients unaware what to expect, could cause anxiety or distress
26	Timing of discussion - delayed until near death	Clinician delays discussion until near to patient death to increase the certainty of prognostic information and avoid uncertainty	Family and patient informed late of prognosis/minimal time to process and plan due to clinician low tolerance of uncertainty
27	Prognostic uncertainty as barrier to discussion, often because of poor tolerance by clinician	Discussion avoided by healthcare professionals	Barrier to timely and appropriate ACP engagement
28	Uncertainty avoided or un-acknowledged by clinicians	Family left with responsibility to communicate	Family distress and potential poor health outcomes
29	clinician does not tolerate uncertainty	clinician delays conversation until prognostic uncertainty resolved	late communication of prognosis or prognostic uncertainty, low patient understanding, delayed decisions, however may be appropriate if information may be available soon
30	deteriorating patient	use of proxy constructs e.g. offering unscheduled hospital appointment to communicate short prognosis	lack of clarity can lead to misunderstandings, but may also foster hope
31	uncertainty presented as certainty by clinicians- positive framing	expected outcome does not occur	clinician feels distressed

For peer review only

	Context	Mechanism	Outcome
32	use of jargon/poor communication training etc	lack of clarity/prognostic uncertainty not communicated in a way that patients/families can understand	patient and family do not understand prognostic uncertainty
33	death is expected/in terminal phase	clinician introduces optimistic uncertainty to avoid giving bad news	false hope
34	advance care planning (ACP) discussion	Clinician concern that prognostic uncertainty means decision making is inappropriate	uncertainty is a barrier to ACP if using the 'decision making' model, but not if outlining future possibilities.
35	particularly unpredictable illness situation	challenging for clinicians to manage expectations in a changing situation	higher risk that patient and family do not understand uncertainty
36	finely balanced decision re aggressiveness of future care (e.g. in heart failure)	tacit/implicit/no communication of prognostic uncertainty	lack of understanding leading to aggressive care not in line with preferences.
37	different illnesses have different levels of uncertainty and reactions to it	tailor prognostic communication to nature of illness & trajectory	better prognostic awareness in illnesses that have more unpredictable trajectories
38	clinician does not tolerate uncertainty	uncertainty presented as certainty – positive or negative framing	false hope feelings of distress, hopelessness, abandonment
39	differences in patient vs relative communication needs	standardised communication	needs not met
40	no recognition of prognostic uncertainty	Communication frameworks and tools including AI nudge re potentially poor prognosis	increased likelihood of communication of prognosis when these tools are used
41	Unclear prognostic information and disclosure of uncertainty without explaining future possibilities	Patient perceives positive prognosis	Patient pursues unnecessarily aggressive treatments
42	ACP discussions	managing prognostic uncertainty by incorporating discussion of 'typical' illness trajectories	supports care planning in context of prognostic uncertainty
43	lack of time/resources, lack of access to clinicians	weak care relationship	implicit/tacit/no communication of prognostic uncertainty

For peer review only

	Context	Mechanism	Outcome
44	poor continuity of care	conflicting information from different providers	uncertainty (and associated distress) magnified, further discussions may be avoided for fear of introducing more conflicting information
45	variation in communication practices dependent on care environment/ culture (e.g. hospice vs hospital etc)	ability to make time for these discussions	explicit/open communication of prognostic uncertainty
46	socio-ethico-political norms and values	ability/willingness of patient/family to engage with prognostic information	level of understanding & ability to plan based on prognostic knowledge
47	patient/family sociocultural background	Communication not tailored to language/cultural preferences	patient and family do not understand prognostic uncertainty
48	cultural barriers to discussing uncertainty e.g. misconceptions that palliative care and dying are synonymous	clinicians less likely to raise uncertainty. focus on disease management rather than wider concerns	lack of communication/discussions delayed until uncertainty resolved
49	culture of avoiding explicit discussion of prognosis or uncertainty (high context communication)	family believe informing patient may harm their wellbeing	a) patient may have poor understanding of prognostic information, potentially causing anxiety and further uncertainty of situation b) cultural preferences are met
50	Patient/family spiritual/sociocultural background and views	Patient blocks or avoids discussion of prognosis and uncertainty "keeping it positive"	Discussion is delayed until patient is nearer end of life/ prognosis is more certain and short, resulting in more limited scope for care planning
51	Patient/family spiritual/sociocultural background and views	Family requests that patient is not given prognostic information within certain cultural contexts	Patient has no understanding or knowledge of prognosis - family may have been told separately
52	patient who lacks capacity to contribute to discussions e.g. advanced dementia	Degree of family awareness of patients wishes	Family awareness of patient wishes affects how easy it is to discuss prognosis or end of life care, which may affect understanding of uncertain illness trajectory

For peer review only

References for all included articles

1. Alwidyan T, McCorry NK, Parsons C. Healthcare professionals' perspectives of deprescribing in older patients at the end of life in hospice care: a qualitative study using the Theoretical Domains Framework. Int J Pharm Pract. 2023;31(3):305-13.
2. Anderson RJ. Communication with the Relatives and Friends of Imminently Dying Patients in a Hospice. England: University of London, University College London (United Kingdom); 2020.
3. Anderson RJ, Stone PC, Low JTS, Bloch S. Managing uncertainty and references to time in prognostic conversations with family members at the end of life: A conversation analytic study. Palliat Med. 2020;34(7):896-905.
4. Anderson RJ, Stone PC, Low JTS, Bloch S. Transitioning out of prognostic talk in discussions with families of hospice patients at the end of life: A conversation analytic study. Patient Educ Couns. 2021;104(5):1075-85.
5. Anstey S. Clinical thinking, knowledge and the referral practices of consultant physicians to specialist palliative care services: an interpretative phenomenological analysis. Wales: Cardiff University (United Kingdom); 2012.
6. Bailey SJ, Cogle K. Talking about dying: How to begin honest conversations about what lies ahead: Royal College of Physicians; 2018.
7. Barclay S, Momen N, Case-Upton S, Kuhn I, Smith E. End-of-life care conversations with heart failure patients: a systematic literature review and narrative synthesis. Br J Gen Pract. 2011;61(582):e49-62.
8. Baum LVM, Friedman D. The Uncertain Science of Predicting Death. JAMA Netw Open. 2020;3(4):e201736.
9. Bayuo J, Bristowe K, Harding R, Agbeko AE, Wong FKY, Agyei FB, et al. "Hanging in a balance": A qualitative study exploring clinicians' experiences of providing care at the end of life in the burn unit. Palliat Med. 2021;35(2):417-25.
10. Becker C, Beck K, Vincent A, Hunziker S. Communication challenges in end-of-life decisions. Swiss Med Wkly. 2020;150:w20351.
11. Bgs. End of Life Care in Frailty: Identification and prognostication. CLINICAL GUIDELINES. 2020.
12. Bloomer MJ, Botti M, Runacres F, Poon P, Barnfield J, Hutchinson AM. Communicating end-of-life care goals and decision-making among a multidisciplinary geriatric inpatient rehabilitation team: A qualitative descriptive study. Palliat Med. 2018;32(10):1615-23.
13. Bloomer MJ, Botti M, Runacres F, Poon P, Barnfield J, Hutchinson AM. End-of-life care for older people in subacute care: A retrospective clinical audit. Collegian. 2019;26(1):22-7.
14. Borbasi JAL, Tong A, Ritchie A, Poulos CJ, Clayton JM. "A good death but there was all this tension around"- perspectives of residential managers on the experience of delivering end of life care for people living with dementia. BMC Geriatr. 2021;21(1):306.
15. Bristowe K, Carey I, Hopper A, Shouls S, Prentice W, Caulkin R, et al. Patient and carer experiences of clinical uncertainty and deterioration, in the face of limited reversibility: A comparative observational study of the AMBER care bundle. Palliat Med. 2015;29(9):797-807.
16. Broom A, Kirby E, Good P, Wootton J, Adams J. The troubles of telling: managing communication about the end of life. Qual Health Res. 2014;24(2):151-62.

For peer review only

17. Bruun A, White N, Oostendorp L, Stone P, Bloch S. Time estimates in prognostic discussions: A conversation analytic study of hospice multidisciplinary team meetings. Palliat Med. 2024;38(5):593-601.
18. Bruun A, White N, Oostendorp L, Stone P, Bloch S. Prognostication As an Interactionally Delicate Matter: A Conversation Analytic Study of Hospice Multidisciplinary Team Meetings. Health Commun. 2025;40(6):956-64.
19. Burgers VWG, van den Bent MJ, Darlington AE, Gualtherie van Weezel AE, Compter A, Tromp JM, et al. A qualitative study on the challenges health care professionals face when caring for adolescents and young adults with an uncertain and/or poor cancer prognosis. ESMO Open. 2022;7(3):100476.
20. Cagle JG, McClymont KM, Thai JN, Smith AK. "If You Don't Know, All of a Sudden, They're Gone": Caregiver Perspectives About Prognostic Communication for Disabled Elderly Adults. J Am Geriatr Soc. 2016;64(6):1299-306.
21. Carlini J, Bahudin D, Michaleff ZA, Plunkett E, She EN, Clark J, et al. Discordance and concordance on perception of quality care at end of life between older patients, caregivers and clinicians: a scoping review. Eur Geriatr Med. 2022;13(1):87-99.
22. Cauley CE, Block SD, Koritsanszky LA, Gass JD, Frydman JL, Nurudeen SM, et al. Surgeons' Perspectives on Avoiding Nonbeneficial Treatments in Seriously Ill Older Patients with Surgical Emergencies: A Qualitative Study. J Palliat Med. 2016;19(5):529-37.
23. Cavanagh CE, Rosman L, Spatz ES, Fried T, Gandhi PU, Soucier RJ, et al. Dying to know: prognosis communication in heart failure. ESC Heart Fail. 2020;7(6):3452-63.
24. Chan WC. Being aware of the prognosis: how does it relate to palliative care patients' anxiety and communication difficulty with family members in the Hong Kong Chinese context? J Palliat Med. 2011;14(9):997-1003.
25. Chang A, Datta-Barua I, McLaughlin B, Daly B. A survey of prognosis discussions held by health-care providers who request palliative care consultation. Palliat Med. 2014;28(4):312-7.
26. Chen JJ, Gamble K, Graham-Wisener L, McGlade K, Doherty J, Donnelly P, et al. GP perceptions of the adequacy of community-based care for patients with advanced heart failure in a UK region (NI): a qualitative study. Open Heart. 2018;5(1):e000734.
27. Chen JJ, Roldan CS, Nichipor AN, Balboni TA, Krishnan MS, Revette AC, et al. Prognostic Understanding and Goals of Palliative Radiotherapy: A Qualitative Study. J Pain Symptom Manage. 2022;64(6):567-76.
28. Chou WS, Hamel LM, Thai CL, Debono D, Chapman RA, Albrecht TL, et al. Discussing prognosis and treatment goals with patients with advanced cancer: A qualitative analysis of oncologists' language. Health Expect. 2017;20(5):1073-80.
29. Chow J, Senderovich H. It's Time to Talk: Challenges in Providing Integrated Palliative Care in Advanced Congestive Heart Failure. A Narrative Review. Curr Cardiol Rev. 2018;14(2):128-37.
30. Christakis NA. Prognostication and bioethics. Daedalus. 1999;128(4):197-214.
31. Christakis NA, Fox RC. Prognostication and death in medical thought and practice. United States -- Pennsylvania: University of Pennsylvania; 1995.
32. Chu C, Anderson R, White N, Stone P. Prognosticating for Adult Patients With Advanced Incurable Cancer: a Needed Oncologist Skill. Curr Treat Options Oncol. 2020;21(1):5.

For peer review only

33. Chu C, White N, Stone P. Prognostication in palliative care. Clin Med (Lond). 2019;19(4):306-10.
34. Clarke G, Fistein E, Holland A, Tobin J, Barclay S, Barclay S. Planning for an uncertain future in progressive neurological disease: a qualitative study of patient and family decision-making with a focus on eating and drinking. BMC Neurol. 2018;18(1):115.
35. Cohen RA, Jackson VA, Norwich D, Schell JO, Schaefer K, Ship AN, et al. A Nephrology Fellows' Communication Skills Course: An Educational Quality Improvement Report. Am J Kidney Dis. 2016;68(2):203-11.
36. Combes S. Conversations on Living and Dying: Facilitating Advance Care Planning with Older People Living with Frailty. Developing and Refining an Intervention. England: University of London, King's College (United Kingdom); 2021.
37. Connolly T, Coats H, DeSanto K, Jones J. The experience of uncertainty for patients, families and healthcare providers in post-stroke palliative and end-of-life care: a qualitative meta-synthesis. Age Ageing. 2021;50(2):534-45.
38. Cormican O, Dowling M. Providing Care to People Living with a Chronic Hematological Malignancy: A Qualitative Evidence Synthesis of Informal Carers' Experiences. Semin Oncol Nurs. 2022;38(6):151338.
39. Cottrell L, Economos G, Evans C, Silber E, Burman R, Nicholas R, et al. A realist review of advance care planning for people with multiple sclerosis and their families. PLoS One. 2020;15(10):e0240815.
40. Creutzfeldt CJ, Robinson MT, Holloway RG. Neurologists as primary palliative care providers: Communication and practice approaches. Neurol Clin Pract. 2016;6(1):40-8.
41. Crowther JL. Dementia: What Care Do Patients and Informal Carers Need in the Last Year of Life and Time Surrounding Death? An Exploratory Qualitative Study. England: The University of Liverpool (United Kingdom); 2011.
42. Dias LM, Barra W, Rego F. Advance Care Planning in Oncology: Challenges in Clinical Practice. Portugal: Universidade do Porto (Portugal); 2022.
43. Dickerson LK, Lipson TA, Chauhan SSB, Allen GI, Young B, Park JO, et al. Evaluating surgeon communication of pancreatic cancer prognosis using the VitalTalk ADAPT framework. J Surg Oncol. 2024;130(3):476-84.
44. Diggle MFJ, Schutz S, Butcher D. Bereaved Caregivers' Experiences of End of Life Care For People With Advanced Heart Failure: A Narrative Synthesis. Omega (Westport). 2025;90(4):1581-608.
45. Disler R, Pascoe A, Luckett T, Donesky D, Irving L, Currow DC, et al. Barriers to Palliative Care Referral and Advance Care Planning (ACP) for Patients With COPD: A Cross-Sectional Survey of Palliative Care Nurses. Am J Hosp Palliat Care. 2022;39(2):169-77.
46. Downer K, Gustin J, Lincoln T, Goodman L, Barnett MD. Communicating About Time-Limited Trials. Chest. 2022;161(1):202-7.
47. Ekberg S, Parry R, Land V, Ekberg K, Pino M, Antaki C, et al. Communicating with patients and families about illness progression and end of life: a review of studies using direct observation of clinical practice. BMC Palliat Care. 2021;20(1):186.
48. Epstein RM. Facing epistemic and complex uncertainty in serious illness: The role of mindfulness and shared mind. Patient Educ Couns. 2021;104(11):2635-42.
49. Etkind SN, Fennessy R, Wang J, Simpson RM, O'Neill B, Barclay SIG, et al. Communicating prognostic uncertainties in advanced multimorbidity: a multimethod qualitative study to co-design practice recommendations. Eur Geriatr Med. 2025.

50. Etkind SN, Koffman J. Approaches to managing uncertainty in people with life-limiting conditions: role of communication and palliative care. Postgrad Med J. 2016;92(1089):412-7.
51. Evans LR, Boyd EA, Malvar G, Apatira L, Luce JM, Lo B, et al. Surrogate decision-makers' perspectives on discussing prognosis in the face of uncertainty. Am J Respir Crit Care Med. 2009;179(1):48-53.
52. Fallowfield LJ, Jenkins VA, Beveridge HA. Truth may hurt but deceit hurts more: communication in palliative care. Palliat Med. 2002;16(4):297-303.
53. Fenton A, Fletcher KM, Kizildag D, Borstelmann NA, Kessler D, Cronin C, et al. Cancer Caregivers' Prognostic and End-of-Life Communication Needs and Experiences and their Impact. J Pain Symptom Manage. 2023;65(1):16-25.
54. Fisher M, Ridley S. Uncertainty in end-of-life care and shared decision making. Critical care and resuscitation : journal of the Australasian Academy of Critical Care Medicine. 2012;14(1):81-7.
55. Fitzsimons D, Doherty LC, Murphy M, Dixon L, Donnelly P, McDonald K, et al. Inadequate Communication Exacerbates the Support Needs of Current and Bereaved Caregivers in Advanced Heart Failure and Impedes Shared Decision-making. J Cardiovasc Nurs. 2019;34(1):11-9.
56. Formagini T, Poague C, O'Neal A, Brooks JV. "When I Heard the Word Palliative": Obscuring and Clarifying Factors Affecting the Stigma Around Palliative Care Referral in Oncology. JCO Oncol Pract. 2022;18(1):e72-e9.
57. Gadoud AC. A palliative care approach for people with advanced heart failure: recognition of need, transitions in care and impact on patients, family carers and clinicians. England: University of Hull (United Kingdom); 2013.
58. Garland EL, Bruce A, Stajduhar K. Exposing barriers to end-of-life communication in heart failure: an integrative review. Canadian journal of cardiovascular nursing = Journal canadien en soins infirmiers cardio-vasculaires. 2013;23(1):12-8.
59. Geerse OP, Lamas DJ, Sanders JJ, Paladino J, Kavanagh J, Henrich NJ, et al. A Qualitative Study of Serious Illness Conversations in Patients with Advanced Cancer. J Palliat Med. 2019;22(7):773-81.
60. Gerlach C, Goebel S, Weber S, Weber M, Sleeman KE. Space for intuition - the 'Surprise'-Question in haemato-oncology: Qualitative analysis of experiences and perceptions of haemato-oncologists. Palliat Med. 2019;33(5):531-40.
61. Gofton TE, Chum M, Schulz V, Gofton BT, Sarpal A, Watling C. Challenges facing palliative neurology practice: A qualitative analysis. J Neurol Sci. 2018;385:225-31.
62. Gonella S, Basso I, Dimonte V, Giulio PD. The role of end-of-life communication in contributing to palliative-oriented care at the end-of-life in nursing home. Int J Palliat Nurs. 2022;28(1):16-26.
63. Goodlin SJ, Quill TE, Arnold RM. Communication and decision-making about prognosis in heart failure care. J Card Fail. 2008;14(2):106-13.
64. Goss AL, Voumard RR, Engelberg RA, Curtis JR, Creutzfeldt CJ. Do They Have a Choice? Surrogate Decision-Making After Severe Acute Brain Injury. Crit Care Med. 2023;51(7):924-35.
65. Gough N, Ross JR, Riley J, Judson I, Koffman J. 'When something is this rare ... how do you know bad really is bad...?'-views on prognostic discussions from patients with advanced soft tissue sarcoma. BMJ Support Palliat Care. 2019;9(1):100-7.

For peer review only

66. Gramling R, Norton S, Ladwig S, Winters P, Metzger M, Quill T, et al. Latent classes of prognosis conversations in palliative care: a mixed-methods study. J Palliat Med. 2013;16(6):653-60.
67. Gramling R, Norton SA, Ladwig S, Metzger M, DeLuca J, Gramling D, et al. Direct observation of prognosis communication in palliative care: a descriptive study. J Pain Symptom Manage. 2013;45(2):202-12.
68. Gray TF, Temel JS, El-Jawahri A. Illness and prognostic understanding in patients with hematologic malignancies. Blood Rev. 2021;45:100692.
69. Green E, Gardiner C, Gott M, Ingleton C. Exploring the extent of communication surrounding transitions to palliative care in heart failure: the perspectives of health care professionals. Journal of palliative care. 2011;27(2):107-16.
70. Gutierrez KM. Communication of prognostic information in an ICU at end of life: practices among and between nurses, physicians and family members: University of Minnesota; 2010.
71. Gutierrez KM. Prognostic communication of critical care nurses and physicians at end of life. Dimens Crit Care Nurs. 2012;31(3):170-82.
72. Gutierrez KM. Prognostic categories and timing of negative prognostic communication from critical care physicians to family members at end-of-life in an intensive care unit. Nurs Inq. 2013;20(3):232-44.
73. Habib AR, Chen R, Magnavita ES, Jaung T, Awad MM, Odejide O, et al. Prevalence and Tolerance of Prognostic Uncertainty Among Thoracic Oncologists. Oncologist. 2021;26(8):e1480-e2.
74. Habib AR, Cronin AM, Earle CC, Tulsky JA, Mack JW, Abel GA, et al. How Do Blood Cancer Doctors Discuss Prognosis? Findings from a National Survey of Hematologic Oncologists. J Palliat Med. 2019;22(6):677-84.
75. Hagerty RG, Butow PN, Ellis PM, Dimitry S, Tattersall MH. Communicating prognosis in cancer care: a systematic review of the literature. Ann Oncol. 2005;16(7):1005-53.
76. Halkett GK, Lobb EA, Oldham L, Nowak AK. The information and support needs of patients diagnosed with High Grade Glioma. Patient Educ Couns. 2010;79(1):112-9.
77. Hallen SA, Hootsmans NA, Blaisdell L, Gutheil CM, Han PK. Physicians' perceptions of the value of prognostic models: the benefits and risks of prognostic confidence. Health Expect. 2015;18(6):2266-77.
78. Han PK. The Need for Uncertainty: A Case for Prognostic Silence. Perspect Biol Med. 2016;59(4):567-75.
79. Han PK, Dieckmann NF, Holt C, Gutheil C, Peters E. Factors Affecting Physicians' Intentions to Communicate Personalized Prognostic Information to Cancer Patients at the End of Life: An Experimental Vignette Study. Med Decis Making. 2016;36(6):703-13.
80. Hancock K, Clayton JM, Parker SM, Wal der S, Butow PN, Carrick S, et al. Truth-telling in discussing prognosis in advanced life-limiting illnesses: a systematic review. Palliat Med. 2007;21(6):507-17.
81. Harrison M, Gardiner C, Taylor B, Ejegi-Memeh S, Darlison L. Understanding the palliative care needs and experiences of people with mesothelioma and their family carers: An integrative systematic review. Palliat Med. 2021;35(6):1039-51.
82. Harrison M, Han PKJ, Rabin B, Bell M, Kay H, Spooner L, et al. Communicating uncertainty in cancer prognosis: a review of web-based prognostic tools. Patient education and counseling. 2019;102(5):842-9.

For peer review only

83. Hendershot KA, Elias MN, Taylor BL, Wahlster S, Creutzfeldt CJ. An Update on Palliative Care in Neurocritical Care: Providing Goal-Concordant Care in the Face of Prognostic Uncertainty. Curr Treat Options Neurol. 2023;25(12):517-29.
84. Henselmans I, Smets EMA, Han PKJ, de Haes H, Laarhoven H. How long do I have? Observational study on communication about life expectancy with advanced cancer patients. Patient Educ Couns. 2017;100(10):1820-7.
85. Heyland DK, Allan DE, Rocker G, Dodek P, Pichora D, Gafni A. Discussing prognosis with patients and their families near the end of life: impact on satisfaction with end-of-life care. Open Med. 2009;3(2):e101-10.
86. Higginson IJ, Koffman J, Hopkins P, Prentice W, Burman R, Leonard S, et al. Development and evaluation of the feasibility and effects on staff, patients, and families of a new tool, the Psychosocial Assessment and Communication Evaluation (PACE), to improve communication and palliative care in intensive care and during clinical uncertainty. BMC medicine. 2013;11:1-14.
87. Hjelmfors L, Stromberg A, Friedrichsen M, Martensson J, Jaarsma T. Communicating prognosis and end-of-life care to heart failure patients: a survey of heart failure nurses' perspectives. Eur J Cardiovasc Nurs. 2014;13(2):152-61.
88. Hjelmfors L, Strömberg A, Friedrichsen M, Mårtensson J, Jaarsma T. Communication About the Heart Failure Trajectory in Patients, Their Families and Health Care Professionals. Sweden: Linkopings Universitet (Sweden); 2018.
89. Hoff L, Hermeren G. Between uncertainty and certainty. The Journal of clinical ethics. 2011;22(2):139-50.
90. Hoff L, Hermeren G. Identifying challenges to communicating with patients about their imminent death. The Journal of clinical ethics. 2014;25(4):296-306.
91. Holloway RG, Gramling R, Kelly AG. Estimating and communicating prognosis in advanced neurologic disease. Neurology. 2013;80(8):764-72.
92. Hudson BF, Shulman C, Low J, Hewett N, Daley J, Davis S, et al. Challenges to discussing palliative care with people experiencing homelessness: a qualitative study. BMJ Open. 2017;7(11):e017502.
93. Hui D, Mo L, Paiva CE. The Importance of Prognostication: Impact of Prognostic Predictions, Disclosures, Awareness, and Acceptance on Patient Outcomes. Curr Treat Options Oncol. 2021;22(2):12.
94. Hui D, Paiva CE, Del Fabbro EG, Steer C, Naberhuis J, van de Wetering M, et al. Prognostication in advanced cancer: update and directions for future research. Support Care Cancer. 2019;27(6):1973-84.
95. Innes S, Payne S. Advanced cancer patients' prognostic information preferences: a review. Palliat Med. 2009;23(1):29-39.
96. International Nurses' End-of-Life Decision-Making in Intensive Care Research G, Gallagher A, Bousso RS, McCarthy J, Kohlen H, Andrews T, et al. Negotiated reorienting: a grounded theory of nurses' end-of-life decision-making in the intensive care unit. Int J Nurs Stud. 2015;52(4):794-803.
97. Isaac M, Curtis JR. How to respond to an ICU patient asking if she/he is going to die. Intensive Care Med. 2017;43(2):220-2.
98. Islam Z, Pollock K, Patterson A, Hanjari M, Wallace L, Mururajani I, et al. Thinking ahead about medical treatments in advanced illness: a qualitative study of barriers and enablers in end-of-life care planning with patients and families from ethnically diverse backgrounds. Health Soc Care Deliv Res. 2023;11(7):1-135.
99. Jackson VA, Emanuel L. Navigating and Communicating about Serious Illness and End of Life. N Engl J Med. 2024;390(1):63-9.

For peer review only

100. Johnson M, Tod AM, Brummell S, Collins K. Prognostic communication in cancer: A critical interpretive synthesis of the literature. Eur J Oncol Nurs. 2015;19(5):554-67.
101. Johnson RF, Jr., Gustin J. Acute lung injury and acute respiratory distress syndrome requiring tracheal intubation and mechanical ventilation in the intensive care unit: impact on managing uncertainty for patient-centered communication. Am J Hosp Palliat Care. 2013;30(6):569-75.
102. Jones MB. A case analysis of the legal, professional, and ethical responsibilities of registered nurses for disclosure of prognostic information to dying adults (philosophy). United States -- Alabama: The University of Alabama at Birmingham; 1983.
103. Kantabanlang Y, Lin CP, Nilmanat K, Guo P. Palliative care needs of patients with hematologic malignancies and family caregivers and challenges of palliative care provision in Asia: A review of evidence. Semin Hematol. 2023;60(4):220-5.
104. Kapo JM, Casarett D. Review: Prognosis in chronic diseases. Annals of Long-Term Care. 2006;14(2):18-23.
105. Kimbell B. Living, dying and caring in advanced liver disease: the challenge of uncertainty. Scotland: The University of Edinburgh (United Kingdom); 2014.
106. Kimbell B, Boyd K, Kendall M, Iredale J, Murray SA. Managing uncertainty in advanced liver disease: a qualitative, multiperspective, serial interview study. BMJ Open. 2015;5(11):e009241.
107. Ko E, Fuentes D, Singh-Carlson S, Nedjat-Haiem F. Challenges and facilitators of hospice decision-making: a retrospective review of family caregivers of home hospice patients in a rural US-Mexico border region-a qualitative study. BMJ Open. 2020;10(7):e035634.
108. Krawczyk M, Gallagher R. Communicating prognostic uncertainty in potential end-of-life contexts: experiences of family members. BMC Palliat Care. 2016;15:59.
109. Kupeli N, Sampson EL, Leavey G, Harrington J, Davis S, Candy B, et al. Context, mechanisms and outcomes in end-of-life care for people with advanced dementia: family carers perspective. BMC Palliat Care. 2019;18(1):87.
110. Langmuir T, Chu A, Sehabi G, Giguere L, Lamarche J, Boudjatat W, et al. A new landscape in illness uncertainty: A systematic review and thematic synthesis of the experience of uncertainty in patients with advanced cancer receiving immunotherapy or targeted therapy. Psychooncology. 2023;32(3):356-67.
111. LeBlanc TW, Temel JS, Helft PR. "How Much Time Do I Have?": Communicating Prognosis in the Era of Exceptional Responders. Am Soc Clin Oncol Educ Book. 2018;38:787-94.
112. LeClaire MM, Oakes JM, Weinert CR. Communication of prognostic information for critically ill patients. Chest. 2005;128(3):1728-35.
113. Lee CW, Vitous CA, Silveira MJ, Forman J, Dossett LA, Mody L, et al. Delays in Palliative Care Referral Among Surgical Patients: Perspectives of Surgical Residents Across the State of Michigan. J Pain Symptom Manage. 2019;57(6):1080-8 e1.
114. Low J, Davis S, Vickerstaff V, Greenslade L, Hopkins K, Langford A, et al. Advanced chronic liver disease in the last year of life: a mixed methods study to understand how care in a specialist liver unit could be improved. BMJ Open. 2017;7(8):e016887.
115. Maguire P, Faulkner A. Communicate with cancer patients: 2. Handling uncertainty, collusion, and denial. BMJ. 1988;297(6654):972-4.

For peer review only

116. Mandel EI, Bernacki RE, Block SD. Serious Illness Conversations in ESRD. Clin J Am Soc Nephrol. 2017;12(5):854-63.
117. Marie C. Difficult conversations with dying people and their families: Marie Curie; 2014.
118. Marie C. What's stopping doctors having that conversation about death? Marie Curie. 2019.
119. Marie C. Tips for talking to your patients about a prognosis. Marie Curie. 2020.
120. Masterson MP, Applebaum AJ, Buda K, Reisch S, Rosenfeld B. Don't Shoot the Messenger: Experiences of Delivering Prognostic Information in the Context of Advanced Cancer. Am J Hosp Palliat Care. 2018;35(12):1526-31.
121. Maxwell CA, Mixon AS, Conner E, Phillippi JC. Receptivity of Hospitalized Older Adults and Family Caregivers to Prognostic Information about Aging, Injury, and Frailty: A Qualitative Study. Int J Nurs Stud. 2020;109:103602.
122. McClung JA. End-of-life care in the treatment of advanced heart failure in the elderly. Cardiol Rev. 2013;21(1):9-15.
123. McDonald A, Sherlock J. A long and winding road: Improving communication with patients in the NHS: Marie Curie; 2016.
124. McLennon SM, Uhrich M, Lasiter S, Chamness AR, Helft PR. Oncology nurses' narratives about ethical dilemmas and prognosis-related communication in advanced cancer patients. Cancer Nurs. 2013;36(2):114-21.
125. Medendorp NM, Stiggelbout AM, Aalfs CM, Han PKJ, Smets EMA, Hillen MA. A scoping review of practice recommendations for clinicians' communication of uncertainty. Health Expect. 2021;24(4):1025-43.
126. Michael N, Shah R. Communication about Advanced Progressive Disease , Prognosis, and Advance Care Plans. In: Kissane DWMW, William B, editors. Psycho-Oncology in Palliative and End of Life Care. New York: Oxford Academic; 2022. p. 1-C.P207.
127. Moglia C, Palumbo F, Botto R, Iazzolino B, Ticozzi N, Calvo A, et al. Prognostic communication in amyotrophic lateral sclerosis: findings from a Nationwide Italian survey. Neurol Sci. 2024;45(12):5787-94.
128. Molzahn AE, Sheilds L, Bruce A, Schick-Makaroff K, Antonio M, Clark AM. Life and priorities before death: A narrative inquiry of uncertainty and end of life in people with heart failure and their family members. Eur J Cardiovasc Nurs. 2020;19(7):629-37.
129. Momen NC, Barclay SI. Addressing 'the elephant on the table': barriers to end of life care conversations in heart failure - a literature review and narrative synthesis. Curr Opin Support Palliat Care. 2011;5(4):312-6.
130. Mone S, Kerr H. Prognostic awareness in advanced cancer: an integrative literature review. BMJ Support Palliat Care. 2021;11(1):53-8.
131. Mount BM. Psychological Impact of Urologic Cancer. Cancer. 1980;45 Suppl 7:1985-92.
132. Nadicksbernd J. Why Do Community Healthcare Professionals Find Some Conversations Difficult? England: University of London, Royal Holloway College (United Kingdom); 2023.
133. Nagelschmidt K, Leppin N, Seifart C, Rief W, von Blanckenburg P. Systematic mixed-method review of barriers to end-of-life communication in the family context. BMJ Support Palliat Care. 2021;11(3):253-63.

For peer review only

134. Narayan M, Jones J, Portalupi LB, McIlvennan CK, Matlock DD, Allen LA. Patient Perspectives on Communication of Individualized Survival Estimates in Heart Failure. J Card Fail. 2017;23(4):272-7.
135. Ngwenya N, Crang C, Farquhar M, Rintoul RC, Mahadeva R, Calvert LD, et al. Communicating uncertainty: contrasting the communication experiences of patients with advanced COPD and incurable lung cancer. Fam Pract. 2021;38(5):637-43.
136. Nice. Care of dying adults in the last days of life. NICE. 2015.
137. Nice. Chronic heart failure in adults: diagnosis and management. NICE. 2018.
138. Noble H, Brazil K, Burns A, Hallahan S, Normand C, Roderick P, et al. Clinician views of patient decisional conflict when deciding between dialysis and conservative management: Qualitative findings from the PAlliative Care in chronic Kidney diSease (PACKS) study. Palliat Med. 2017;31(10):921-31.
139. Olsson MM, Windsor C, Chambers S, Green TL. A Scoping Review of End-of-Life Communication in International Palliative Care Guidelines for Acute Care Settings. J Pain Symptom Manage. 2021;62(2):425-37 e2.
140. Owusuaa C, van Beelen I, van der Heide A, van der Rijt CCD. Physicians' views on the usefulness and feasibility of identifying and disclosing patients' last phase of life: a focus group study. BMJ Support Palliat Care. 2021;14(e2).
141. Pardue CM. Uncertainty in the context of end-of-life communication in heart failure. Dissertation Abstracts International: Section B: The Sciences and Engineering. 2021;82(8):No-Specified.
142. Parker S, Clayton J, Hancock K, Walder S, Butow P, Carrick S, et al. Communicating Prognosis and Issues Surrounding End of Life (EOL) in adults in the advanced stages of a life-limiting illness: A Systematic Review. 2006.
143. Parker SM, Clayton JM, Hancock K, Walder S, Butow PN, Carrick S, et al. A systematic review of prognostic/end-of-life communication with adults in the advanced stages of a life-limiting illness: patient/caregiver preferences for the content, style, and timing of information. J Pain Symptom Manage. 2007;34(1):81-93.
144. Parry R. Communication in Palliative Care and About End of Life: A State-of-the-Art Literature Review of Conversation-Analytic Research in Healthcare. Research on Language and Social Interaction. 2024;57(1):127-48.
145. Parvez S, Abdel-Kader K, Song MK, Unruh M. Conveying uncertainty in prognosis to patients with ESRD. Blood Purif. 2015;39(1-3):58-64.
146. Payne S, Burton C, Addington-Hall J, Jones A. End-of-life issues in acute stroke care: a qualitative study of the experiences and preferences of patients and families. Palliat Med. 2010;24(2):146-53.
147. Peoples HA, Boone B, Blumenthal-Barby JS, Bruce CR. How Clinician-Family Interactions Potentially Impact Clinicians' Conceptualization and Discussions Regarding Prognostic Uncertainties. J Palliat Care. 2020;35(1):29-33.
148. Perumalswami CR, Jagsi R, Goold SD. Predicting a "Lazarus Effect" in Patients With Advanced Cancer Near the End of Life: Prognostic Uncertainty, Oncologists' Emotions, and Ethical Questions. Am J Bioeth. 2019;19(12):57-60.
149. Pfeil TA, Laryionava K, Reiter-Theil S, Hiddemann W, Winkler EC. What keeps oncologists from addressing palliative care early on with incurable cancer patients? An active stance seems key. Oncologist. 2015;20(1):56-61.
150. Piovesan M, Orr P, Tevyaw S, Roussos E, Cherid C, Bouchard S. A Prospective Study with Patients and Families on the Usefulness of Accurate

For peer review only

Prognosis for Palliative Care Patients. J Soc Work End Life Palliat Care. 2024;20(2):133-46.
151. Pontin D, Jordan N. Issues in prognostication for hospital specialist palliative care doctors and nurses: a qualitative inquiry. Palliat Med. 2013;27(2):165-71.
152. Pooler C, Richman-Eisenstat J, Kalluri M. Early integrated palliative approach for idiopathic pulmonary fibrosis: A narrative study of bereaved caregivers' experiences. Palliat Med. 2018;32(9):1455-64.
153. Powers BA, Norton SA, Schmitt MH, Quill TE, Metzger M. Meaning and practice of palliative care for hospitalized older adults with life limiting illnesses. J Aging Res. 2011;2011:406164.
154. Pun J, Chow JCH, Fok L, Cheung KM. Role of patients' family members in end-of-life communication: an integrative review. BMJ Open. 2023;13(2):e067304.
155. Rafferty KA, Cramer EM, Priddis D. Managing end-of-life uncertainty: applying problematic integration theory to spousal communication about death and dying. American Journal of Hospice and Palliative Medicine®. 2016;33(1):69-76.
156. Reid C, Gibbins J, Bloor S, Burcombe M, McCoubrie R, Forbes K. Healthcare professionals' perspectives on delivering end-of-life care within acute hospital trusts: a qualitative study. BMJ Support Palliat Care. 2015;5(5):490-5.
157. Rhee JJ, Zwar NA, Kemp LA. Why are advance care planning decisions not implemented? Insights from interviews with Australian general practitioners. J Palliat Med. 2013;16(10):1197-204.
158. Richards N, Ingleton C, Gardiner C, Gott M. Awareness contexts revisited: indeterminacy in initiating discussions at the end-of-life. J Adv Nurs. 2013;69(12):2654-64.
159. Ridley S, Fisher M. Uncertainty in end-of-life care. Curr Opin Crit Care. 2013;19(6):642-7.
160. Risco JR, Kelly AG, Holloway RG. Prognostication in neurology. Handb Clin Neurol. 2022;190:175-93.
161. Robinson J, Pilbeam C, Goodwin H, Raphael D, Waterworth S, Gott M. The impact of uncertainty on bereaved family's experiences of care at the end of life: a thematic analysis of free text survey data. BMC Palliat Care. 2021;20(1):60.
162. Rowe JG, Potts M, McGhie R, Dinh A, Engel I, England K, et al. Palliative Care Practice During the COVID-19 Pandemic: A Descriptive Qualitative Study of Palliative Care Clinicians. J Pain Symptom Manage. 2021;62(6):1111-6.
163. Salins N, Dhyani VS, Mathew M, Prasad A, Rao AP, Damani A, et al. Assessing palliative care practices in intensive care units and interpreting them using the lens of appropriate care concepts. An umbrella review. Intensive Care Med. 2024;50(9):1438-58.
164. Sanders JJ, Blanch-Hartigan D, Ericson J, Tarbi E, Rizzo D, Gramling R, et al. Methodological innovations to strengthen evidence-based serious illness communication. Patient Educ Couns. 2023;114:107790.
165. Scholz B, Goncharov L, Emmerich N, Lu VN, Chapman M, Clark SJ, et al. Clinicians' accounts of communication with patients in end-of-life care contexts: A systematic review. Patient Educ Couns. 2020;103(10):1913-21.
166. Schuster RA, Hong SY, Arnold RM, White DB. Do physicians disclose uncertainty when discussing prognosis in grave critical illness? Narrat Inq Bioeth. 2012;2(2):125-35.
167. Schutz REC, Creutzfeldt CJ. Three big things in neuropalliative care: Communication, personhood and uncertainty. J Neurol Sci. 2018;385:232-3.

For peer review only

168. Sedhom R, Bates-Pappas GE, Feldman J, Elk R, Gupta A, Fisch MJ, et al. Tumor Is Not the Only Target: Ensuring Equitable Person-Centered Supportive Care in the Era of Precision Medicine. Am Soc Clin Oncol Educ Book. 2024;44(3):e434026.
169. Selman LE, Bristowe K, Higginson IJ, Murtagh FEM. The views and experiences of older people with conservatively managed renal failure: a qualitative study of communication, information and decision-making. BMC Nephrol. 2019;20(1):38.
170. Sikhosana N, Whitehead D, Moxham L, Karacsony S, Namasivayam P, Fernandez R. Voice of persons with a life-limiting illness in conversation with healthcare professionals: systematic review and meta-synthesis. BMJ Support Palliat Care. 2023.
171. Simpkin AL, Armstrong KA. Communicating Uncertainty: a Narrative Review and Framework for Future Research. J Gen Intern Med. 2019;34(11):2586-91.
172. Smith AK, Glare P. Ethical issues in prognosis and prognostication. The Oxford handbook of ethics at the end of life. 2014:170-89.
173. Stanek MS, Norton SA. Expressions of Uncertainty from Patients with Advanced Cancer when Communicating with the Palliative Care Team. United States -- New York: University of Rochester; 2020.
174. Stiel H, Nagarajan SV, Forster BC, Clayton JM. Palliative care physicians' perspectives on transferring patients to nursing homes and communication strategies to facilitate this transition: A qualitative study. Palliat Med. 2019;33(3):323-31.
175. Stocker R. Experiences and perceptions of the role of palliative and end of life care in heart failure: a modified grounded theory study. England: University of Durham (United Kingdom); 2014.
176. Stocker R, Close H, Hancock H, Hungin APS. Should heart failure be regarded as a terminal illness requiring palliative care? A study of heart failure patients', carers' and clinicians' understanding of heart failure prognosis and its management. BMJ Support Palliat Care. 2017;7(4):464-9.
177. Sullivan AM, Lakoma MD, Matsuyama RK, Rosenblatt L, Arnold RM, Block SD. Diagnosing and discussing imminent death in the hospital: a secondary analysis of physician interviews. J Palliat Med. 2007;10(4):882-93.
178. Szawarski P. Classic cases revisited: Oscar the cat and predicting death. J Intensive Care Soc. 2016;17(4):341-5.
179. Tarbi EC, Gramling R, Bradway C, Broden EG, Meghani SH. "I Had a Lot More Planned": The Existential Dimensions of Prognosis Communication with Adults with Advanced Cancer. J Palliat Med. 2021;24(10):1443-54.
180. Tavares N, Hunt KJ, Jarrett N, Wilkinson TM. The preferences of patients with chronic obstructive pulmonary disease are to discuss palliative care plans with familiar respiratory clinicians, but to delay conversations until their condition deteriorates: A study guided by interpretative phenomenological analysis. Palliat Med. 2020;34(10):1361-73.
181. Tavares NC. Palliative Care Communication in Copd : Patients' Preferences and Clinicians' Judgements. England: University of Southampton (United Kingdom); 2019.
182. Temel JS, Shaw AT, Greer JA. Challenge of Prognostic Uncertainty in the Modern Era of Cancer Therapeutics. J Clin Oncol. 2016;34(30):3605-8.

For peer review only

183. Tetteh DA, Akhther N. Openness and topic avoidance in interpersonal communication about ovarian cancer: An uncertainty management perspective. Qual Res Med Healthc. 2021;5(3):9376.
184. Tilburgs B, Vernooij-Dassen M, Koopmans R, van Gennip H, Engels Y, Perry M. Barriers and facilitators for GPs in dementia advance care planning: A systematic integrative review. PLoS One. 2018;13(6):e0198535.
185. Tolsa L, Jones L, Michel P, Borasio GD, Jox RJ, Rutz Voumard R. 'We Have Guidelines, but We Can Also Be Artists': Neurologists Discuss Prognostic Uncertainty, Cognitive Biases, and Scoring Tools. Brain Sci. 2022;12(11):1591.
186. Trankle SA, Shanmugam S, Lewis E, Nicholson M, Hillman K, Cardona M. Are We Making Progress on Communication with People Who Are Near the End of Life in the Australian Health System? A Thematic Analysis. Health Commun. 2020;35(2):158-67.
187. Tuck KK, Brod L, Nutt J, Fromme EK. Preferences of patients with Parkinson's disease for communication about advanced care planning. Am J Hosp Palliat Care. 2015;32(1):68-77.
188. Udo C, Lovgren M, Lundquist G, Axelsson B. Palliative care physicians' experiences of end-of-life communication: A focus group study. Eur J Cancer Care (Engl). 2018;27(1).
189. van der Velden NCA, Han PKJ, van Laarhoven HWM, de Vos F, Hendriks LEL, Burgers SA, et al. Prevalence and Predictors of Physician-Patient Discordance in Prognostic Perceptions in Advanced Cancer. Oncologist. 2023;28(8):e653-e68.
190. van der Velden NCA, Meijers MC, Han PKJ, van Laarhoven HWM, Smets EMA, Henselmans I. The Effect of Prognostic Communication on Patient Outcomes in Palliative Cancer Care: a Systematic Review. Curr Treat Options Oncol. 2020;21(5):40.
191. van der Velden NCA, Smets EMA, van Vliet LM, Brom L, van Laarhoven HWM, Henselmans I. Effects of Prognostic Communication Strategies on Prognostic Perceptions, Treatment Decisions and End-Of-Life Anticipation in Advanced Cancer: An Experimental Study among Analogue Patients. J Pain Symptom Manage. 2024;67(6):478-89 e13.
192. van der Velden NCA, Smets EMA, van Vliet LM, Brom L, van Laarhoven HWM, Henselmans I. Effects of prognostic communication strategies on emotions, coping, and appreciation of consultations: An experimental study in advanced cancer. Palliat Support Care. 2024:1-13.
193. van der Velden NCA, van der Kleij MBA, Lehmann V, Smets EMA, Stouthard JML, Henselmans I, et al. Communication about Prognosis during Patient-Initiated Second Opinion Consultations in Advanced Cancer Care: An Observational Qualitative Analysis. Int J Environ Res Public Health. 2021;18(11).
194. van Eenennaam RM, Kruithof WJ, van Es MA, Kruitwagen-van Reenen ET, Westeneng HJ, Visser-Meily JMA, et al. Discussing personalized prognosis in amyotrophic lateral sclerosis: development of a communication guide. BMC Neurol. 2020;20(1):446.
195. Vlckova K, Tuckova A, Polakova K, Loucka M. Factors associated with prognostic awareness in patients with cancer: A systematic review. Psychooncology. 2020;29(6):990-1003.
196. Walczak A, Butow PN, Bu S, Clayton JM. A systematic review of evidence for end-of-life communication interventions: Who do they target, how are they structured and do they work? Patient Educ Couns. 2016;99(1):3-16.

For peer review only

197. Walczak A, Butow PN, Davidson PM, Bellemore FA, Tattersall MH, Clayton JM, et al. Patient perspectives regarding communication about prognosis and end-of-life issues: how can it be optimised? Patient Educ Couns. 2013;90(3):307-14.
198. Waldrop DP, Meeker MA, Kerr C, Skretny J, Tangeman J, Milch R. The nature and timing of family-provider communication in late-stage cancer: a qualitative study of caregivers' experiences. J Pain Symptom Manage. 2012;43(2):182-94.
199. Wijsenbeek MS, Bonella F, Orsatti L, Russell AM, Valenzuela C, Wuyts WA, et al. Communicating with patients with idiopathic pulmonary fibrosis: can we do it better? ERJ Open Res. 2022;8(1):00422-2021.
200. Wittenberg SM, Cohen LM. Estimating prognosis in end-stage renal disease. Progress in Palliative Care. 2013;17(4):165-9.
201. Wittenberg-Lyles EM, Goldsmith J, Sanchez-Reilly S, Ragan SL. Communicating a terminal prognosis in a palliative care setting: deficiencies in current communication training protocols. Soc Sci Med. 2008;66(11):2356-65.
202. Wu Y, Zhang X. Examining Conversation Analysis in Palliative Care: A Systematic Review. Health Commun. 2024;39(13):3072-83.
203. Xie W, Butcher R. Artificial Intelligence Decision Support Tools for End-of-Life Care Planning Conversations: CADTH Horizon Scan. 2023.
204. Young J, Lyons A, Egan R, Dew K. Embodied decisions unfolding over time: a meta-ethnography systematic review of people with cancer's reasons for delaying or declining end-of-life care. BMC Palliat Care. 2024;23(1):45.
205. Ziegler DS. Never say never. J Clin Oncol. 2015;33(5):518-20.

For peer review only
