# Supplementary material for: Improving communication of prognostic uncertainty in advanced illness: a realist review
Source: BMJ Open. 2026 Jul 1;16(7):e115385. doi: 10.1136/bmjopen-2025-115385 (PMC13663710; doi:10.1136/bmjopen-2025-115385)
Supplement: Supplementary file 9 [file bmjopen-2025-115385.reviewer_comments.pdf]

PEER REVIEW HISTORY

BMJ Open publishes all reviews undertaken for accepted manuscripts. Reviewers are asked to complete a checklist review form and are provided with free text boxes to elaborate on their assessment. These free text comments are reproduced below.

ARTICLE DETAILS

Title (Provisional)

Improving Communication of Prognostic Uncertainty in Advanced Illness: A Realist Review

Authors

Etkind, Simon; Weetman, Katharine; Hyams, Katy; Acharya, Tanvi; Shokraneh, Farhad; Macartney, John I

VERSION 1 - REVIEW

**Reviewer**	**1**
**Name**	**Peeler, Anna**
**Affiliation**	**Cicely Saunders Institute of Palliative Care, Policy, and Rehabilitation, King's College London**
**Date**	**11-Mar-2026**
**COI**	**None**

Thank you very much for the opportunity to review this manuscript. This realist review presents a timely synthesis of a highly complex and clinically relevant topic: how prognostic uncertainty is communicated in advanced illness, why communication succeeds or fails, and for whom. The topic has clear international relevance, aligns with BMJ Open's remit, and responds to a major evidence gap. The breadth of data (205 studies), methodological transparency, and depth of interpretation are commendable. With targeted edits, particularly regarding methodological clarity, conceptual framing, and presentation, I would recommend this manuscript for publication. Below, I provide section-by-section comments and suggestions for strengthening the manuscript.

Abstract

- Clearly summarises the aims, methods, and major findings, and effectively conveys the breadth of the evidence included
- Stakeholder involvement is a key component of realist reviews, but this is not mentioned at all in the abstract. Adding in details about your stakeholder group would strengthen the methods here.

Introduction / Background

- Frames prognostic uncertainty well as a critical challenge in advanced illness and situates the need for improved communication within demographic, clinical, and ethical contexts
- The rationale for using realist synthesis is well justified and aligns with the complexity of the topic, though explicitly emphasising how previous prognostic communication reviews do not unpack mechanisms of uncertainty would sharpen the argument

Methods

- The search strategy is comprehensive and well documented, including grey literature and cross-disciplinary sources
- Best practice dictates that at least two reviewers should examine each search results in either the title/abstract screening or to the full text review. It might be too late to change this, but it could be acknowledged in the limitations.
- The description of coding procedures, team triangulation, and iterative CMOC refinement is robust, but the explanation of how disagreements were resolved could be made more explicit
- There is very little description of how the initial programme theory was developed. Describing which and how many paper informed the theory, who the patient and clinician expert contributors were and how they were selected, and how the theory changed with their guidance would be helpful (this might be in Appendix 2 as it is mentioned, but I can't seem to find this in the submission, or it's not labelled). Additionally, providing a brief explanation of how the team determined that the theory was sufficiently refined (e.g., conceptual saturation or stability) would enhance methodological clarity.
- A clearer description of how the stakeholder group was selected would be helpful. Including their demographics/clinical experience in the results would improve transparency.

Results

- The description of how contextual factors (e.g., preferences, clinician experience, sociocultural norms) interact with mechanisms (explicitness, timing, avoidance) is rich and insightful
- The findings around hope, trust, and emotional outcomes are especially compelling, though the variability of evidence (e.g., mixed findings on distress) could be more explicitly acknowledged at each relevant point.

Discussion

- The discussion provides thoughtful interpretive commentary and effectively connects the programme theory to broader literature on prognostic communication and illness trajectories; nevertheless, a few statements suggest causal relationships where realist interpretation would favour generative tendencies.
- Some paragraphs closely repeat content already presented in the Results section. Avoid repetition would sharpen the narrative and highlight the manuscript's original contributions.
- Given that training, confidence, and uncertainty tolerance repeatedly emerged as key mechanisms, expanding the discussion on implications for clinician training (e.g., skills development, reflective practice) would add value.
- The attention to sociocultural dimensions is well handled, though briefly acknowledging the limited empirical evidence in this area, and the risks of stereotyping, would add more nuance.
- The manuscript would benefit from more explicit articulation of how this realist synthesis advances the field beyond previous reviews: namely, by unveiling the underlying mechanisms shaping prognostic uncertainty communication.
- The strengths and limitations section presents a candid and balanced assessment, clearly acknowledging the breadth of included evidence and the role of stakeholders in shaping programme theory
- The geographic concentration of included studies is appropriately noted, but highlighting the absence of perspectives from LMICs or low-resource settings (as this is where the majority of people with palliative care need live) would further strengthen this reflection.

**Reviewer**	**2**
**Name**	**Fukahori, Hiroki**
**Affiliation**	**Keio University**
**Date**	**06-Apr-2026**
**COI**	**None**

Reviewer's Comments

Overall Evaluation

This paper synthesizes evidence from 205 sources using a realist review methodology. The scope of the work is commendable, and it provides valuable insights for both academia and clinical practice. However, I found certain sections difficult to follow, particularly regarding the transparency of the analysis process and the connection between the 52 CMOCs and the final Programme Theory. Detailed comments are provided below to assist the authors in

strengthening the manuscript. As I am still refining my expertise in realist methodology, I welcome any clarification from the authors if my comments reflect a misunderstanding of your specific approach.

Abstract

Geographical Bias:

Given that the 205 sources are predominantly from 11 countries (Europe, North America, and Oceania), it would be helpful to briefly mention this geographic focus in the Abstract for the benefit of international readers.

Consistency with Results:

The description of the results in the Abstract deviates slightly from the Final Programme Theory (Figure 2). For instance, "communication models and tools" are not explicitly highlighted in the figure. It would be clearer if the Abstract and figures were more explicitly aligned.

Specificity of Findings:

The statement that effective communication is characterized by "openness, empathy, and tailoring" feels somewhat generic for a realist review. It might be useful to at least signal that "openness" may not be universally effective across all cultural or national contexts.

Novelty:

In the conclusion of the Abstract, it would be beneficial to state more explicitly what new understanding this review adds compared to existing literature.

Overreach:

The claim that this approach "may enhance trust, preparedness, and shared care planning" seems a bit premature, as the specific approach has not yet been developed or tested. A more cautious tone might be appropriate here.

Background

Terminology: (P5, Line 30)

The scope of "evidence" is currently ambiguous. Please define what types of evidence were included.

Stakeholder Details:

Please provide more detail regarding the stakeholders, patients, and clinical experts involved (e.g., demographics, professional background, and the total number of participants).

Development of the Initial Programme Theory (IPT)

Process Transparency:

The description of how the IPT was developed is insufficient. Please clarify the process: How many papers informed the IPT? How was the theory justified and refined?

Methodological Rigor:

There is a lack of methodological citations regarding the IPT development. Please cite relevant realist methodology and detail the specific steps taken during the discussion phase to formalize the theory.

Search Strategy

Librarian Involvement:

It should be clarified whether a professional librarian or information specialist was involved in designing the search strategy.

Language Bias:

While the authors state that no language limits were applied, the databases used often default to English-language publications. Please address whether this resulted in a practical exclusion of non-English literature.

Screening and Data Extraction

Justification of Percentages:

Please provide citations or methodological justifications for why only 10% of titles/abstracts and 30% of full texts were double-screened. Similarly, justify the 10% double-check for data extraction.

Quality Appraisal:

Clarify if the quality appraisal (within the extraction form) was performed by a single reviewer or if it was also subject to a double-check.

Coding Transparency:

The coding process requires a more detailed explanation grounded in realist literature. Without a transparent audit trail, the transition from raw data to the final theory appears somewhat subjective.

Synthesis

Process Specification:

How was Appendix 6 (Table 2) derived? Please clarify how the IPT was utilized during the coding and synthesis stages.

Stakeholder Engagement:

Provide specific details on stakeholder involvement during synthesis (e.g., number of meetings, roles of participants, and how their feedback specifically shaped the CMOCs).

Results

Quality vs. Theory Building:

The results suggest that only a subset of papers (82 and 63, respectively) were rated high in rigor or relevance. Please clarify—either in the Methods or Results—how papers with "low" or "moderate" relevance were treated during theory development.

CMOC Quantity:

It is somewhat surprising that only 52 CMOCs were extracted from a substantial pool of 205 sources.

Figure first:

I suggest placing Figure 2 at the beginning of the CMOC section to improve readability.

Conceptual Alignment:

The authors have organized the 52 CMOCs into eight thematic areas, which is a practical way to manage such a large volume of data. However, from a reader's perspective, the logical connection between these individual CMOCs and the integrated Final Programme Theory (Figure 2) is somewhat difficult to follow. Additionally, it remains unclear "how" the communication of prognostic uncertainty works, "for whom," and "in what context." It would be helpful if the authors could provide a clearer rationale or a brief narrative explanation in the text to bridge these 52 configurations with the overarching themes and brief explanation of mechanism of how the communication works. This would significantly enhance the transparency and theoretical clarity of the synthesis.

Visual Clarity of the Theory:

The simplified figure (Figure 2) makes it difficult to see how the 52 individual CMOCs map onto the overall theory. To improve clarity, the authors might consider incorporating the more detailed figure from the Appendix into the main text, or perhaps using it to replace Figure 2.

Discussion

Linearity:

The statement that "the degree of explicitness affects shared understanding... impacting distress and acceptance" suggests a linear relationship that the complex realist data may not fully support. I recommend a more nuanced explanation that reflects the non-linear nature of these interactions.

Cultural Bias:

While stakeholder involvement is a strength, if the stakeholders were limited to specific Western cultures, the resulting theory may be culturally biased. This is particularly critical for communication and end-of-life care, which are deeply influenced by cultural norms and healthcare systems.

Limitations:

The authors correctly identify the geographic bias (USA, Europe, Australia) in the limitations. As noted earlier, this should be signaled earlier in the paper to assist international readers.

Meaning & Implications

Tempered Claims:

Given that the theory was developed partly from "low" or "moderate" quality evidence, the recommendations for healthcare professionals should be more cautious. I suggest emphasizing the need for empirical validation of this theory—including the development of educational and intervention programs—before its widespread integration into education or clinical practice.

I hope these comments are helpful for the further development of your manuscript.

VERSION 1 - AUTHOR RESPONSE

	Reviewer 1 Comment	Author Response	Location of Changes
1	Thank you very much for the opportunity to review this manuscript. This realist review presents a timely synthesis of a highly complex and clinically relevant topic: how prognostic uncertainty is communicated in advanced illness, why communication succeeds or fails, and for whom. The topic has clear international relevance, aligns with BMJ Open's remit, and responds to a major evidence gap. The breadth of data (205 studies), methodological transparency, and depth of interpretation are commendable. With targeted edits, particularly regarding methodological clarity, conceptual framing, and presentation, I would recommend this manuscript for publication. Below, I provide section-by-section comments and suggestions for strengthening the manuscript.	Thank you for this positive comment	
2	Abstract<br>• Clearly summarises the aims, methods, and major findings, and effectively conveys the breadth of the evidence included<br>• Stakeholder involvement is a key component of realist reviews, but this is not mentioned at all in the abstract. Adding in details about your stakeholder group would strengthen the methods here.	Thank you<br>We have added details of the stakeholder group here to note that it included clinical and lay advisors. There is not space for extensive information in the abstract but we have added more detail in the main paper	

3	Introduction / Background<br>• Frames prognostic uncertainty well as a critical challenge in advanced illness and situates the need for improved communication within demographic, clinical, and ethical contexts<br>• The rationale for using realist synthesis is well justified and aligns with the complexity of the topic, though explicitly emphasising how previous prognostic communication reviews do not unpack mechanisms of uncertainty would sharpen the argument	Thank you<br>Thank you, we agree and have added the phrase "and existing research has not explicitly focused on the mechanisms of communication" to the introduction	Introduction paragraph 4
4	Methods<br>• The search strategy is comprehensive and well documented, including grey literature and cross-disciplinary sources	Thank you	
5	Best practice dictates that at least two reviewers should examine each search results in either the title/abstract screening or to the full text review. It might be too late to change this, but it could be acknowledged in the limitations.	Full double screening was not possible given the resources available for the project and the size of the literature to be synthesised. We have acknowledged this in the limitations	Discussion strengths/ limitations
6	The description of coding procedures, team triangulation, and iterative CMOC refinement is robust, but the explanation of how disagreements were resolved could be made more explicit	Thank you, we have added detail	Methods, screening & data extraction
7	There is very little description of how the initial programme theory was developed. Describing which and how many paper informed the theory, who the patient and clinician expert contributors were and how they were selected, and how the theory changed with their guidance would be helpful (this might be in Appendix 2 as it is mentioned, but I can't seem to find this in the submission, or it's not labelled). Additionally, providing a brief explanation of how the team determined that the theory was sufficiently refined (e.g., conceptual saturation or stability) would enhance methodological clarity.	Thank you, we have added relevant detail to the methods, including number of papers, details of advisory group. We have also expanded on appendix 2 to add more detail (apologies if this was not visible to the reviewer in the initial submission).	Methods and appendix 2

8	A clearer description of how the stakeholder group was selected would be helpful. Including their demographics/clinical experience in the results would improve transparency.	Whilst we do not have consent to publish individual demographics, we have added details of the advisory group and how they were selected<br><br>"The review was supported by an advisory group of two clinicians (one GP, one palliative care consultant, and two lay advisors with relevant lived experience. The group comprised advisors from a range of geographical areas and cultural backgrounds."	Methods, design
9	Results<br>• The description of how contextual factors (e.g., preferences, clinician experience, sociocultural norms) interact with mechanisms (explicitness, timing, avoidance) is rich and insightful	Thank you	
10	The findings around hope, trust, and emotional outcomes are especially compelling, though the variability of evidence (e.g., mixed findings on distress) could be more explicitly acknowledged at each relevant point.	We agree, hope was found to have a nuanced relationship with uncertainty, including as an inhibitor to discussion, a result of communication of uncertainty, and a counterpoint to distress. The relevant section already acknowledges the variability of evidence and we have made some changes to make this even more explicit.	Results
11	Discussion<br>• The discussion provides thoughtful interpretive commentary and effectively connects the programme theory to broader literature on prognostic communication and illness trajectories; nevertheless, a few statements suggest causal relationships where realist interpretation would favour generative tendencies.	We agree this research cannot determine causation. We have reviewed wording in the discussion and made some adjustments to avoid giving this impression e.g. changed "the degree of explicitness affects shared understanding" to<br>"the degree of explicitness appears to influence shared understanding"	Discussion
12	Some paragraphs closely repeat content already presented in the Results section. Avoid repetition would sharpen the narrative and highlight the manuscript's original contributions.	We have amended the discussion, particularly the comparison with existing literature, to more explicitly bring out what this review adds	Discussion, comparison with existing literature
13	Given that training, confidence, and uncertainty tolerance repeatedly emerged as key mechanisms, expanding the discussion on implications for clinician training (e.g., skills	We have rephrased the 'meaning and implications section to provide a more	Discussion, meaning and implications

	development, reflective practice) would add value.	direct and specific discussion of training	
14	The attention to sociocultural dimensions is well handled, though briefly acknowledging the limited empirical evidence in this area, and the risks of stereotyping, would add more nuance.	We feel this has already been addressed in the 'comparison with existing literature' section of the discussion, where the text specifically acknowledges limited empirical evidence and the importance of avoiding assumptions/generalisations based on cultural background (i.e. stereotyping)	
15	The manuscript would benefit from more explicit articulation of how this realist synthesis advances the field beyond previous reviews: namely, by unveiling the underlying mechanisms shaping prognostic uncertainty communication.	We have adjusted the wording and added information to the strengths/limitations section	Strengths/limitations and discussion
16	The strengths and limitations section presents a candid and balanced assessment, clearly acknowledging the breadth of included evidence and the role of stakeholders in shaping programme theory	Thank you	
17	The geographic concentration of included studies is appropriately noted, but highlighting the absence of perspectives from LMICs or low-resource settings (as this is where the majority of people with palliative care need live) would further strengthen this reflection.	We have added this detail about the limits of the evidence base	

	**Reviewer 2 comment**	**Author response**	**Location of Changes**
1	This paper synthesizes evidence from 205 sources using a realist review methodology. The scope of the work is commendable, and it provides valuable insights for both academia and clinical practice. However, I found certain sections difficult to follow, particularly regarding the transparency of the analysis process and the connection between the 52 CMOCs and the final Programme Theory. Detailed comments are provided below to assist the authors in strengthening the manuscript. As I am still refining my expertise in realist methodology, I welcome any clarification from the authors if my	Thank you for this assessment, detailed responses below	

	comments reflect a misunderstanding of your specific approach.		
2	Geographical Bias:<br>Given that the 205 sources are predominantly from 11 countries (Europe, North America, and Oceania), it would be helpful to briefly mention this geographic focus in the Abstract for the benefit of international readers.	We have acknowledged this in the abstract	
3	Consistency with Results:<br>The description of the results in the Abstract deviates slightly from the Final Programme Theory (Figure 2). For instance, "communication models and tools" are not explicitly highlighted in the figure. It would be clearer if the Abstract and figures were more explicitly aligned.	Thank you, we have corrected "tools" to "interventions" in the abstract and made minor amendments to the abstract to align terminology	Abstract
4	Specificity of Findings:<br>The statement that effective communication is characterized by "openness, empathy, and tailoring" feels somewhat generic for a realist review. It might be useful to at least signal that "openness" may not be universally effective across all cultural or national contexts.	We have amended to "There was no single model of effective communication as this varied based on individual characteristics and preferences, but openness was usually viewed positively"	Abstract
5	Novelty:<br>In the conclusion of the Abstract, it would be beneficial to state more explicitly what new understanding this review adds compared to existing literature.	Thank you, we have added that this review synthesises information across illness contexts to highlight common strands of effective communication. We have added an example of insights regarding mechanisms.	Abstract
6	Overreach:<br>The claim that this approach "may enhance trust, preparedness, and shared care planning" seems a bit premature, as the specific approach has not yet been developed or tested. A more cautious tone might be appropriate here.	We are not advocating for a single specific approach, but rather developing a theory that may inform effective approaches. We have removed this sentence and made edits throughout the abstract for clarity and word count	Abstract
7	Background<br>Terminology: (P5, Line 30)<br>The scope of "evidence" is currently ambiguous. Please define what types of evidence were included.	In line with realist methodology and previous realist reviews, we included a diverse range and types of evidence of relevance to the programme theory. We have clarified this in the methods with citations "we included published peer reviewed papers, and grey literature such as reports, theses, and guidelines."	Methods, inclusion/ exclusion criteria

8	Stakeholder Details:<br>Please provide more detail regarding the stakeholders, patients, and clinical experts involved (e.g., demographics, professional background, and the total number of participants).	Whilst we do not have consent to publish individual demographics, we have added a new section with details of the advisory group and how they were selected. See also response to reviewer 1 comment 8	Methods “User involvement and stakeholder advisory group” section
9	Development of the Initial Programme Theory (IPT)<br>Process Transparency:<br>The description of how the IPT was developed is insufficient. Please clarify the process: How many papers informed the IPT? How was the theory justified and refined?	Details added in methods and appendix 2, see also response to reviewer 1 comment 7	
10	Methodological Rigor:<br>There is a lack of methodological citations regarding the IPT development. Please cite relevant realist methodology and detail the specific steps taken during the discussion phase to formalize the theory.	Thank you, we have added a more detailed description of the development of the IPT including citations<br>Coleman S, et al. Searching for Programme theories for a realist evaluation: a case study comparing an academic database search and a simple Google search. BMC medical research methodology. 2020;20(1):217.<br><br>Weetman K et al. Improving best practice for patients receiving hospital discharge letters: a realist review protocol. BMJ Open. 2017;7(11):e018353.<br><br>Pawson R et al Realist synthesis-an introduction. ESRC res methods program. 2004;2:55.<br><br>Pawson R et al. Realist review - a new method of systematic review designed for complex policy interventions. Journal of Health Services Research & Policy. 2005;10(1_suppl):21-34.	
11	Search Strategy<br>Librarian Involvement:<br>It should be clarified whether a professional librarian or information specialist was involved in designing the search strategy.	One of the authors is an information specialist and led on search design. We have stated this in the “searches” section	Methods
12	Language Bias:<br>While the authors state that no language limits were applied, the databases used often default to English-language publications. Please address	Yes this is a good point, thanks for raising. Whilst some foreign language papers are included in the databases searched, the majority will be in English. We were not able to	Strengths and limitations

	whether this resulted in a practical exclusion of non-English literature.	specifically search foreign language repositories in this project and have acknowledged this limitation in a new strengths and limitations bullet points section requested by the editors	section (after abstract)
13	Screening and Data Extraction<br>Justification of Percentages:<br>Please provide citations or methodological justifications for why only 10% of titles/abstracts and 30% of full texts were double-screened. Similarly, justify the 10% double-check for data extraction.	The 10% double-screen minimum for title/abstract, main text, and data extraction was informed by previous realist reviews (e.g. Lawson et al., 2025; Weetman et al., 2019;)<br>We went above this minimum for full text screening to allow time to align interpretation of inclusion criteria, after 30% double screening we were confident reviewer decision making was aligned. We have added clarification and cited relevant literature in this part of the methods	Methods, screening
14	Quality Appraisal:<br>Clarify if the quality appraisal (within the extraction form) was performed by a single reviewer or if it was also subject to a double-check.	Thank you, 25% of the rigour assessments and 50% of relevance assessments were cross checked by a second researcher and we have added this detail	Methods, rigour and relevance
15	Coding Transparency:<br>The coding process requires a more detailed explanation grounded in realist literature. Without a transparent audit trail, the transition from raw data to the final theory appears somewhat subjective.	We have added detail of the coding process to the methods	Methods, synthesis
16	Synthesis<br>Process Specification:<br>How was Appendix 6 (Table 2) derived? Please clarify how the IPT was utilized during the coding and synthesis stages.	We have added a description of how the CMO table was derived in the 'synthesis' section of the methods	Methods, synthesis
17	Stakeholder Engagement:<br>Provide specific details on stakeholder involvement during synthesis (e.g., number of meetings, roles of participants, and how their feedback specifically shaped the CMOCs).	Detail added to methods	Methods, 'User involvement' and 'synthesis'
18	Results<br>Quality vs. Theory Building:<br>The results suggest that only a subset of papers (82 and 63, respectively) were rated high in rigor	Although rigour and quality were appraised, documents were not excluded solely based on rigour as Pawson et al. (2004; 2005; 2006)	Methods,, rigour and relevance assessments

	or relevance. Please clarify—either in the Methods or Results—how papers with "low" or "moderate" relevance were treated during theory development.	suggest lower quality evidence may still have valid contributions of relevance to theory development, with appropriate contextualisation.<br>We have added detail of how these papers were treated to the methods	
19	CMOC Quantity:<br>It is somewhat surprising that only 52 CMOCs were extracted from a substantial pool of 205 sources	We followed the process described to identify CMOCs. From an initial list of 56 CMOCs, we combined a small number of very similar formulations to give the final list of 52.	
20	Figure first:<br>I suggest placing Figure 2 at the beginning of the CMOC section to improve readability	Figure 2 moved as requested	
21	Conceptual Alignment:<br>The authors have organized the 52 CMOCs into eight thematic areas, which is a practical way to manage such a large volume of data. However, from a reader's perspective, the logical connection between these individual CMOCs and the integrated Final Programme Theory (Figure 2) is somewhat difficult to follow. Additionally, it remains unclear "how" the communication of prognostic uncertainty works, "for whom," and "in what context." It would be helpful if the authors could provide a clearer rationale or a brief narrative explanation in the text to bridge these 52 configurations with the overarching themes and brief explanation of mechanism of how the communication works. This would significantly enhance the transparency and theoretical clarity of the synthesis.	We have added a more detailed description in the methods of how the CMOCs were integrated into thematic areas to build the final theory. If preferable, this could be moved to a supplementary file.	Methods, synthesis
22	Visual Clarity of the Theory:<br>The simplified figure (Figure 2) makes it difficult to see how the 52 individual CMOCs map onto the overall theory. To improve clarity, the authors might consider incorporating the more detailed figure from the Appendix into the main text, or perhaps using it to replace Figure 2.	We are open to using the more detailed figure in the main manuscript. Our judgement had been that a simpler summary figure might sit better in the main text, to provide a clearer overall view of the theory. However we agree the more detailed figure allows for more thorough appraisal of the theory.<br>If there is space we would suggest including the simpler figure (figure 2) at	Results, figure 2 & 3.

		the start of the results section, and including the detailed figure as figure 3 at the end of the results section. We have done this in the revised manuscript	
23	Discussion. Linearity:<br>The statement that "the degree of explicitness affects shared understanding... impacting distress and acceptance" suggests a linear relationship that the complex realist data may not fully support. I recommend a more nuanced explanation that reflects the non-linear nature of these interactions.	Thank you we have rephrased using the word 'appears to influence' which better reflects the complexity of this non-linear relationship	Discussion, first paragraph
24	Cultural Bias:<br>While stakeholder involvement is a strength, if the stakeholders were limited to specific Western cultures, the resulting theory may be culturally biased. This is particularly critical for communication and end-of-life care, which are deeply influenced by cultural norms and healthcare systems.	Stakeholders were from a range of cultural groups, but the resources of this project did not allow a sufficiently large stakeholder group to incorporate a representative sample of perspectives across cultures or internationally. This is somewhat ameliorated by the extent of the evidence synthesised, however the previously noted geographical spread of the literature means that it is not reasonable to say this theory is universally applicable. We are rather arguing that this theory represents a synthesis of the existing evidence, supported by stakeholder views. We have identified areas where further research would be helpful. This has been stated in the discussion the 'future research section'	Discussion and 'unanswered questions and future research'
25	Limitations:<br>The authors correctly identify the geographic bias (USA, Europe, Australia) in the limitations. As noted earlier, this should be signaled earlier in the paper to assist international readers	Added in abstract	abstract
26	Meaning & Implications<br>Tempered Claims:<br>Given that the theory was developed partly from "low" or "moderate" quality evidence, the recommendations for healthcare professionals should be more cautious. I suggest emphasizing	We agree this is worth noting, however the size of the literature synthesised does mean that in many areas we felt there was sufficient evidence to make the relevant recommendations. We have added a qualifying note to the	Discussion, meaning and implications

	the need for empirical validation of this theory—including the development of educational and intervention programs—before its widespread integration into education or clinical practice.	effect that further empirical validation may be useful to inform implementation in specific contexts.	

VERSION 2 - REVIEW

**Reviewer**	**2**
**Name**	**Fukahori, Hiroki**
**Affiliation**	**Keio University**
**Date**	**01-Jun-2026**
**COI**	

The authors have effectively revised their manuscript in accordance with the reviewers' comments. While the original framework was already clear, it has now been further refined, and the methodology is described with much greater clarity. Furthermore, the inclusion of the need to accumulate evidence in low- and middle-income countries adds significant value. Overall, the manuscript is now much more accessible to both international readers and researchers new to the realist approach.
